# Design, Synthesis, and Biological Evaluation of Two Series of Novel A-Ring Fused Steroidal Pyrazines as Potential Anticancer Agents

**DOI:** 10.3390/ijms21051665

**Published:** 2020-02-28

**Authors:** Shijun Wang, Xiaorong Yuan, Hao Qian, Na Li, Junru Wang

**Affiliations:** 1 College of Chemistry & Pharmacy, Northwest A&F University, 22 Xinong Road, Yangling 712100, China; 444795415@nwafu.edu.cn (S.W.);; 2 College of Food Science and Technology, Northwest University, Xi’an 710127, China

**Keywords:** synthesis, fused steroids, pyrazines, anticancer activity, mechanism, docking

## Abstract

Background: Increasingly, different heterocyclic systems have been introduced into the steroid nucleus to significantly enhance the antitumor activities of steroid molecules. However, in this study, few literature precedents describing the pyrazine heterocyclic-condensed modification to an A-ring of steroid monomers were found, although the pyrazine group is thought to be essential for the potent anticancer activity of clinically relevant drugs and natural steroid dimers. Methods and Results: Two series of novel A-ring fused steroidal pyrazines were designed and efficiently synthesized from commercially available progesterone via key α-ketoenol intermediates. Through a cell counting kit-8 cytotoxic assay of 36 derivatives for three tumor cells, 14 compounds displayed significant antiproliferative activity compared to 5-fluorouracil, especially for human prostatic tumor cells (PC-3) in vitro. Further mechanistic studies indicated that the most active compound, **12n** (IC_50_, 0.93 μM; SI, 28.71), could induce the cell apoptosis of PC-3 cells in a dose-dependent manner and cause cell cycle arrest in the G2/M phase. The molecular docking study suggested that compound **12n** fitted the active sites of cytochrome P450 17A1 (6CIZ) well. Conclusions: **12n** might serve as a promising lead compound for the development of novel anticancer drugs. This facile ring-closing strategy may provide a novel and promising avenue for the cycloaddition reaction of the steroidal skeleton through *α*-ketoenol intermediates.

## 1. Introduction

Despite important advances in anticancer therapy, cancer is the second leading cause of death worldwide for humans, following cardiovascular diseases. Currently, chemotherapy has become a routine in most cancer therapy, but it is limited by the emergence of resistant cancer cells. Therefore, developing safer and more selective anticancer drugs is still an unequivocal need. Steroidal derivatives have attracted great attention due to their rigid sterane skeleton with a range of functionalization, the ability to interact with various biological targets and pathways [1], a broad spectrum of bioactivity, the specific ability to penetrate cell membranes, and so on [2,3,4]. The steroidal derivative named abiraterone has been successfully used as a commercial anti-prostate cancer drug.

**Figure 1:**
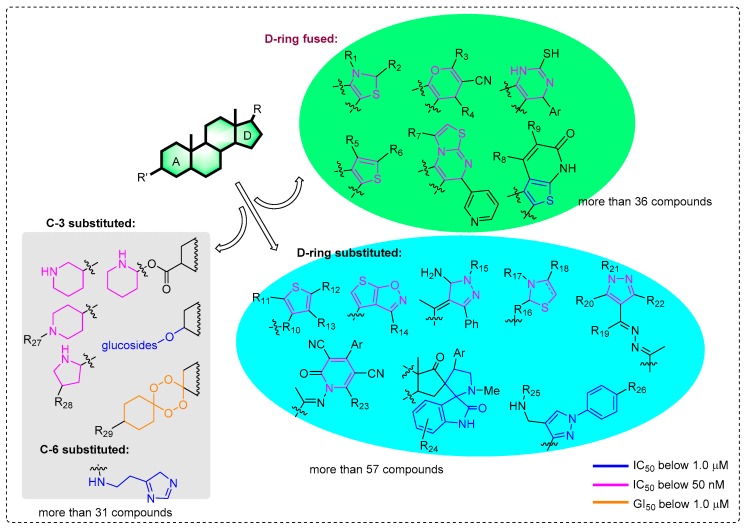
Current potential anticancer steroid stuctures with the IC50/GI50 values below 1.0 μM.

Previous modifications of steroids have mainly focused on the functionalization of the C-3/6/20 position of the steroid nucleus for screening anticancer molecules (Figure 1). More than 124 compounds have significant cytotoxic effects, with IC_50_/GI_50_ values below 1 μM [5,6,7,8,9,10,11,12,13,14,15,16,17,18]. Among them, more than 52 compounds even have values below 50 nM [5,8,12,13,14,15,16,17,18]. In particular, the introduction of different heterocyclic systems into ring D, including pyri(mi)dine [15,16], thiophene, thiazole [12,13], and pyrazole [9,10,13], can significantly enhance the antitumor activities of steroid molecules, even up to nanomolar levels. This is different from the modification of the A ring, which uses the extremely potential bufalin as a starting substrate. As shown in Figure 1, there are 36 D-ring fused steroids and 57 substituted compounds linked by a side-chain which IC_50_ values that can be below 1 *μ*M. However, the anticancer activity of A-ring fused steroids has scarcely been reported, expect for in 2017 (moderate antiproliferative activity) [19]. Meanwhile, few literature precedents describing the pyrazine ring-condensed modification to an A-ring of steroid monomers were also found in this study. Furthermore, the mechanism of antitumor activity for these derivatives has not been deeply investigated, especially for compounds with nanomolar IC_50_ values.

**Figure 2:**
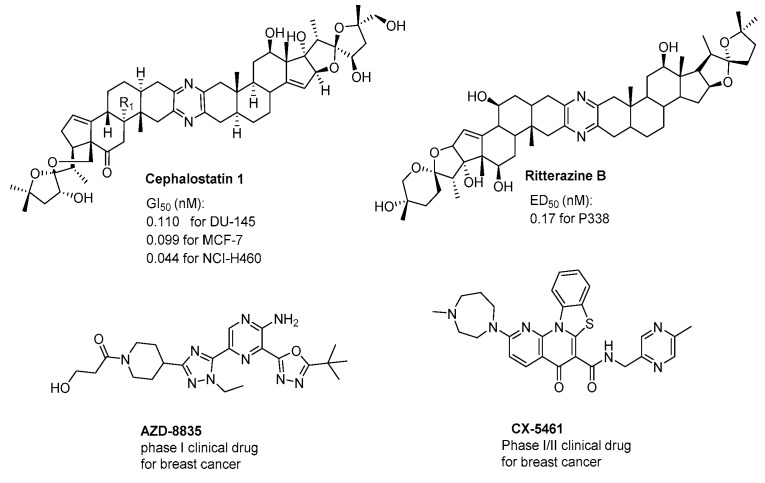
The structures of anticancer agents containing a pyrazine moiety.

In addition, the pyrazines and their derivatives are known to be very important structural motifs in various natural products, such as cephalostatins and ritterazines (Figure 2) [20,21,22], and possess extremely potent anticancer activity (sub-nanomolar levels) [23,24]. Furthermore, the pyrazine ring is also thought to be essential for their strong antitumor activity exhibited in pharmacologically active synthetic compounds (Figure 2) AZD8835 [25] and clinically relevant drugs CX5641 [26].

In view of the pharmacological importance of the steroid skeleton and pyrazine functional group in developing new biologically active steroids, we herein represent the synthesis of two series of novel steroidal A-ring fused pyrazines and their antiproliferative activity against human cancer cell lines (MCF-7, prostatic tumor cells (PC-3), and HepG2) and a human normal liver cell (THLE-2) in vitro. Our further goal was to explore their effects on the cell cycle and possible molecular mechanisms of inducing apoptosis.

## 2. Results

### 2.1. Chemistry

**Scheme 1:**
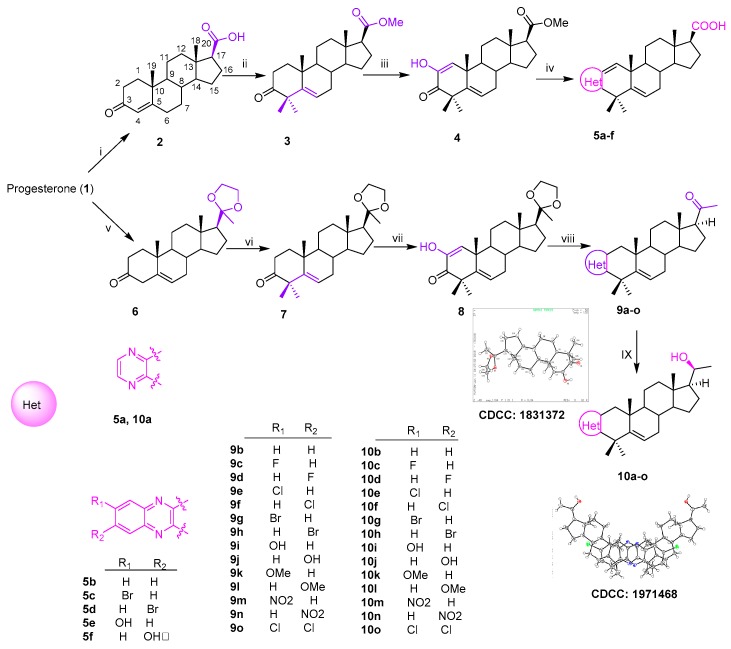
Synthesis of A-ring fused steroidal benzopyrazines.

**Scheme 2:**
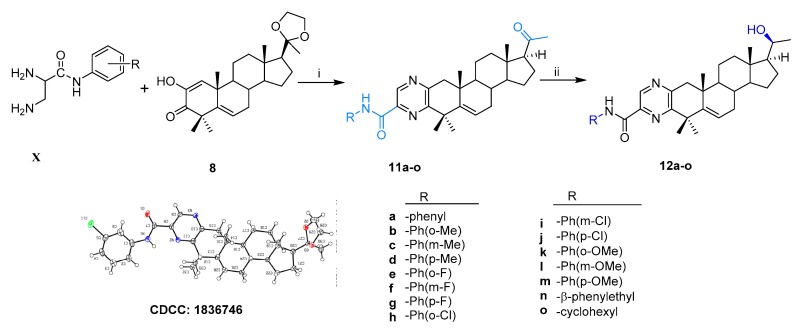
Synthesis of steroidal A-ring fused pyrazinamides. Reagents and conditions: i. (**a**) amine, EtOH, *ρ*-TSA, reflux, 85–90%; (**b**) 1M HCl, 95–99%; ii. NaBH4, dry MeOH/THF (*v*/*v*, 2:1), 0 °C, 85–90%.

Thirty-six novel A-ring fused steroidal pyrazines, belonging to two different series, were designed and efficiently synthesized through four steps, with progesterone **1** as starting material. Among them, twenty-one target derivatives (**5a–5f**, **10a–10o)**, mainly bearing a benzopyrazines (quinoxalines) group, were prepared by the cyclocondensation of key intermediates containing an *α*-ketoenols group (2-hydroxy-3-oxo-△^1,5^ analog) with 1,2-diamines and substituted *o*-phenylenediamines (OPDs) in excellent yields (Scheme 1). The second series of steroidal A-ring fused pyrazines (**12a–12o**), bearing a pyrazinamides moiety, were synthesized through a ring-closing reaction of intermediate **8** and 2,3-diaminopropanamides in similar conditions (Scheme 2).

All the structures of the newly synthesized compounds were identified by NMR and HRMS spectra. The absolute configurations of *α*-ketoenol intermediates **8** and **10g** and the precursor of **11i** were confirmed by the single crystal diffraction method using CuK*α* radiation and deposited with the Cambridge Crystallographic Data Centre (CCDC numbers: 1831372, 1971468 and 1836746, respectively; Scheme 1). The single-molecule structure of **10g** was re-drawn by ShelXle software because of one unit containing two molecules for **10g** (see section 1 of Supplementary Materials).

Reagents and conditions: i. NaOH, Br2, 76%; ii. (a) MeI, K2CO3, acetone, 40 °C, 5 h, 90%; (b) t-BuOK, t-BuOH, MeI, 60%; iii. t-BuOK, dry-THF, O2, 1 h, 69%; iv. (a) EtOH, reflux, overnight; (b) KOH, H2O, EG, reflux, 30%–67%; v. EG, (EtO)4Si, ρ-TsOH (cat.), r.t., 5 h, 95%; vi. t-BuOH, t-BuOK, 1 h, then, MeI, 50%; vii. t-BuOK, t-BuOH, THF, O2, 89%; viii. (a) amine, EtOH, ρ-TSA, reflux; (b) 1M HCl, 77%–90%; (c) NaBH4, dry MeOH/THF (v/v, 2:1), 0 °C, 80%–90%.

#### 2.1.1. A-Ring Fused Steroidal Benzopyrazines **5a–5f** and **10a–10o** (Series 1)

The carboxylic acid **2** was obtained through the reaction of progesterone with bromine and sodium hydroxide [27]. The compound **2** was subsequently transformed to its corresponding methyl ester using methyl iodide [28] in acetone, and then reacted with MeI and *t*-BuOK in dry THF [29], which generated the 4,4-dimethyl compound **3** (Scheme 1). The resulting residue (**3**) was treated with an excess of *t*-BuOK in the *t*-BuOH/THF system (20:1, *v*/*v*) under an oxygen condition [30] to afford the key *α*-ketoenol intermediate compound **4** (Scheme 1). Target derivatives **5a–5f** were achieved over two steps in excellent yields by combining 1,2-diamines or substituted OPDs with key intermediate product (**4**) in refluxing ethanol and then deprotecting though directly adding KOH (Scheme 1).

The compound **10a–10o** was eventually prepared from key intermediate product (**8**) over three simple steps, as shown in Scheme Progesterone was initially selectively protected with the ethylene ketal group [31] to afford compound The subsequent reaction of **6** with *t*-BuOK and MeI took place in *t*-BuOH [32] and led to the 4,4-dimethyl compound The 4,4-dimethyl moiety was beneficial to the following C2 oxidization reaction, to afford specific 2,3-diketone product **8** under the same condition as the formation of compound **4**, the configuration of which was confirmed by X-ray data (Scheme 1, CCDC: 1831372).

The compound **8** was treated with 1,2-diamines and substituted OPDs to get target compounds **9a–9b** and **9g–9j,** which were then converted into the corresponding target derivatives **10a–10b** and **10g–10j** through the removal of 20-ethylene ketal protection and reduction. This scaffold of **10g** was confirmed by X-ray single crystal diffraction (CCDC: 1971468). The antitumor activities of these six compounds containing 20-OH were not obviously different from those of **5a–5f** containing 20-COOH. Therefore, the remaining compounds of A-ring fused steroidal pyrazines **10c–10f** and **10k–10o** were obtained under the same condition. All spectral and analytical data were in agreement with the assigned structures (see section 2 of Supplementary Materials).

#### 2.1.2. A-Ring Fused Steroidal Pyrazinamides **12a–12o** (Series 2)

According to the same condition as above, the treatment of compound **8** with different substituted 2,3-diaminopropanamides (**X**) resulted in the formation of **11a–11o**, which then changed to the corresponding target products **12a–12o** (Scheme 2). The configuration of **11i** bearing the ethylene ketal group was confirmed by X-ray single crystal diffraction (CCDC, 1836746). All the structures of the newly synthesized compounds were confirmed by ^1^H NMR, ^13^C NMR, and HRMS spectra (see section 2 of Supplementary Materials).

### 2.2. Antiproliferative Activity

All the thirty-six target derivatives (**5a–5f, 10a–10o**, and **12a–12o**) were screened for antiproliferative activities against three human cancer cell lines (MCF-7, PC-3, and HepG2) and cytotoxicity on a normal human cell (THLE-2) at the concentration of 20 μM by using the CCK8 assay. The preliminary bioassay results are depicted in Table 1 For PC-3 cell lines, fourteen compounds exhibited equal or superior activity against the PC-3 cell line compared with the commercial drug 5-fluorouracil (5-Fu). Many compounds, such as **5d**, **10d–f, 10j, 10l, 12e–f, 12h–k**, and **12m**, showed moderate antiproliferative activities (30% < inhibition ratios < 60%); **12g** and **12n** displayed remarkable activities (inhibition ratios > 60%) against PC-3 cells. However, for MCF-7 cells, many compounds, except **10l, 12g,** and **12n**, did not display obvious antitumor activities (inhibition ratios < 30%). In addition, for HepG2 cell lines, these compounds, except **10b, 10f, 10l**, and **12n**, did not display excellent antiproliferative activities (inhibition ratios < 30%). Meanwhile, most of the compounds exhibited low cytotoxicity against the THLE-2 cell line. In particular, the IC_50_ values of the compounds **12g** and **12n** were 6.88 and 0.93 μM against the PC-3 cell line, respectively, and the latter possessed a selectivity index (SI) value of up to 28.71 (Table 2).

These compounds containing a halogen atom exhibited better antitumor activity, and the products containing a substitute which was closer to the 4,4-dimethyl moiety displayed better inhibitory activity for **5b–5f** and **10b–10n**. Moreover, variation of the substituted position of the phenyl ring slightly affected the activities, with *para* ˃ *ortho* ˃ *meta* for compounds **12b–12n**.

The differences in the conformation of these compounds, such as the structural rigidity or the molecular volumes, were displayed by using the Molinspiration tool (Table 3) [33]. In the first series of derivatives **10c–10o**, the halogen substitute could apparently enhance the transport properties (lower topologic polar surface area (TPSA)) of compounds. In the second series of derivatives **12a–10o**, the introduction of the F atom could maintain the compounds’ solubility (lower miLogP and molecular volumes). These calculated molecular properties may provide some references to compounds’ lead-like properties in prodrugs.

### 2.3. Morphological Detection of Cell Apoptosis

**Figure 3:**
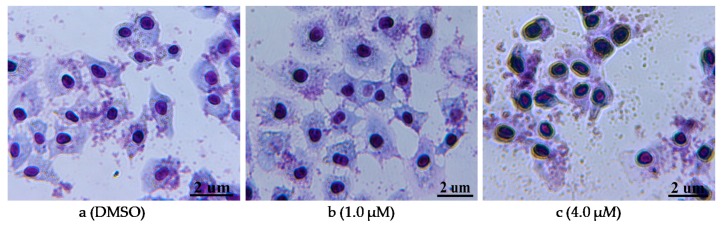
Morphological changes of PC-3 cells through Giemsa staining. (**a**) DMSO used as the negative control; (**b**,**c**) dealt with compound **12n** for 48 h at the concentration of 1.0 and 4.0 μM, respectively.

The compound **12n**, which possessed the strongest activity, was selected to further assess its effects on morphological changes of PC-3 cells. After being treated with **12n** at different concentrations (1.0 and 4.0 μM), the cells were stained with Giemsa stain. As shown in Figure 3a, the cells in the control (0.1% MSO) showed a regular appearance and were surrounded by abundant cytoplasm. In comparison, along with the increased concentration of **12n**, the cytoplasm became deformed and clearly distinguished, and the color of the cell nucleus became deeper so that even the nuclear condensation was exhibited (Figure 3b,c). Other morphological changes, such as the loss of volume and deformation of a regular appearance, were also found. Generally speaking, these results indicated that **12n** could cause the morphological change of PC-3 cells in a dose-dependent manner.

### 2.4. Cell Apoptosis Analysis

**Figure 4:**
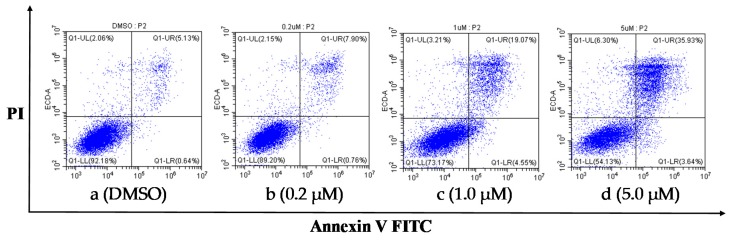
Compound **12n** induced apoptosis in PC-3 cells after 48 h through treatment with **12n** at different concentrations (0.2, 1.0, and 5.0 μM).

To clarify the effect of steroidal pyrazines on the induction of apoptosis in PC-3 cells, a flow cytometry assay with Annexin-V-FITC/PI double staining was implemented. As shown in Figure 4, the apoptotic ratios (including the early and late apoptotic ratios) of PC-3 cells treated with compound **12n** increased gradually in a concentration-dependent manner (Figure 4). Moreover, the apoptosis ratios of compound **12n** at different concentration gradients were found to be 8.66% (0.2 μM), 23.62% (1.0 μM), and 39.57% (5.0 μM), respectively, which were apparently higher than that of the blank control group (5.77%) (Table 4). These results indicated that **12n** induced the apoptosis of PC-3 cells in a dose-dependent manner.

### 2.5. Cell Cycle Assay

**Figure 5:**
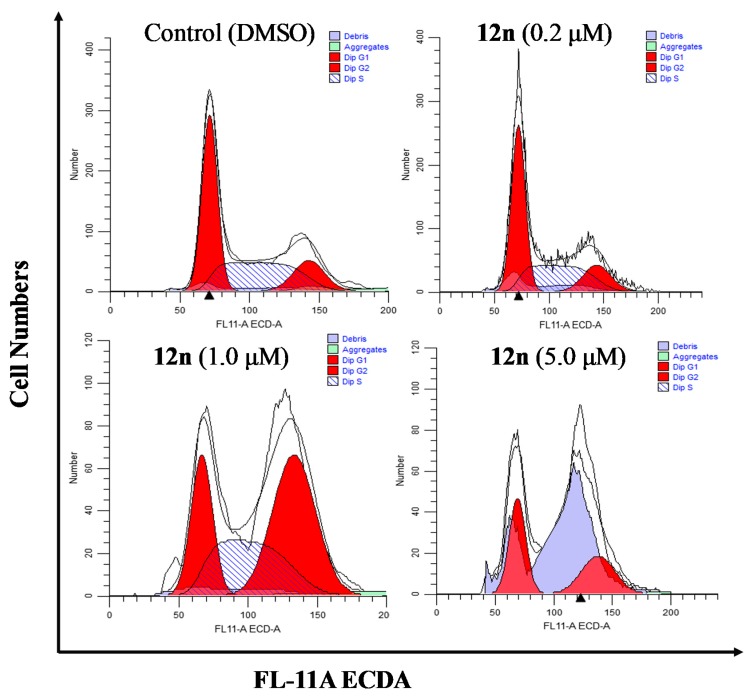
Effects of compound **12n** on the cell cycle distribution of PC-3 cells after treatment for 48 h.

To further clarify the effects of A-ring fused steroidal pyrazines on cell growth, the cell cycle distribution of PC-3 cells was investigated through treatment with compound **12n** at concentration gradients for 48 h (Figure 5 and Table 5). Treatment with **12n** at various concentrations of 0.2, 1.0, and 5.0 μM resulted in a significant accumulation of 16.09%, 46.19%, and 44.27% of cells in the G2/M phase (control group, 15.69%). When the concentration of **12n** was 8 μM, the percentage of cells in the G1 phase increased to 49.62% and G2/M phase increased to 44.27%, whilst exhibiting a substantial decrease of the S phase. These results suggested that derivative **12n** could cause obvious G2/M phase arrest in a dose-dependent manner.

### 2.6. The Molecular Docking

Potential molecular targets for the most active compound, **12n**, were screened by rapid molecular docking against more than ten proteins using the MOE software (Table **6**). The cytochromes P450 17A1/19A1 (CYP17A1/CYP17A1) have a strong correlation with cell apoptosis, because most of them have an Abiraterone-specific active site. As can be seen in Table 6, the CYP17A1 proteins (except 3S7S) have stronger binding abilities than the reference drug Abiraterone acetate (−8.91 kcal/mol). Furthermore, the binding ability of 6CIZ proteins with **12n** was the strongest (−11.72 kcal/mol). This protein could be used to test its binding abilities with various steroidal pyrazines.

As can be seen (Table 7), 36 steroidal pyrazines were further checked by comparisons with Abiraterone acetate used as a commercial anticancer drug. Successful docking indicated that most of the designed compounds had research significance, especially compounds **12a–12n**, which had virtual ligand screening (VLS) score values lower than −10.0 kcal/mol (Abiraterone acetate, −8.91). In addition, among the compounds, **12n** exhibited the lowest score value (−11.87). Steroidal pyrazinamides had lower scores than benzopyrazine derivatives **10a–o**, suggesting better anticancer activity. Substituents close to 4,4-dimethyl had lower scores than those located further away from 4,4-dimethyl. The tendency of docking scores was consistent with that of antiproliferative activities of compounds (Table 1).

**Figure 6:**
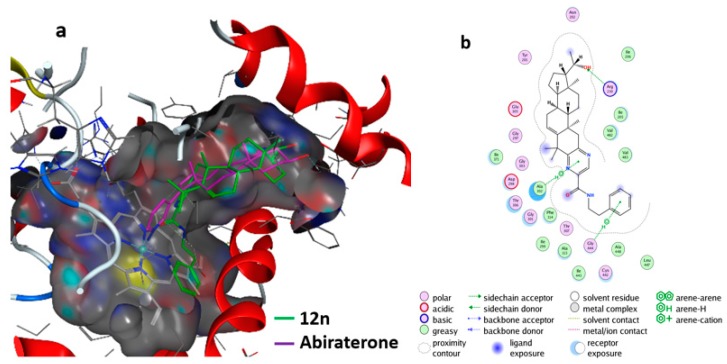
Interaction of the compound **12n** with the CYP17A1 (6CIZ) receptor: (**a**) docking of compound **12n** (green) with 6CIZ and Abiraterone (purple) was used as the reference; (**b**) interaction of compound **12n** with 6CIZ.

Furthermore, the molecular interactions of the most active compound, **19**, with 6CIZ were displayed in detail by using the MOE software. The hypothetical 3D binding modes of compound **12n** and the reference ligands with 6CIZ are presented in Figure 6a. Compound **12n** was predicted to adopt a similar binding space and orientation as Abiraterone. A similar analysis suggests that **12n** could interact strongly with CYP17A1 family proteins (6CIZ). In addition, the two-dimensional (2D) interaction diagram for compound 12n with 6CIZ is shown in Figure 6b. The hydrogen bond in this complex is the most notable: the pyrizine ring, *β*-phenethylamine ring, and 20-OH group could form a hydrogen bond with Ala 302, Gly 444, and Arg of 6CIZ, respectively.

The molecule of compound **12n** could also hydrophobically interact with amino acid residues Ala302, Phe114, Val483, and Val482 (Figure 6b). The pyrizine group played the key role in determining the inhibitory properties of compound **12**, because it not only resulted in the formation of the H-bond with Ala302, but also interacted with Ala302 through hydrophobic and van der Waals contacts [34]. The most active compound, **12n**, fitted the active sites of CYP17A1 (6CIZ) well. These docking results further proved the bioactivity and pharmacological potential of compound **12n** against PC-3 cells. These ligand-based screening results could be used as a guide for the deep optimization of pre-drugs.

### 2.7. Preliminary Structure–Activity Relationship

Generally, combining the CCK-8 assay and molecular docking screening results, a preliminary structure–activity relationship could be obtained. The halogen substituents were beneficial to improving the antitumor activity. For benzopyrazine derivatives **10a–o**, substituents close to 4,4-dimethyl were better than those located further away from 4,4-dimethyl. The modification using pyrazinamides was more conducive to anticancer activity than that using benzopyrazines.

## 3. Discussion

The formation of α-ketoenols (2-hydroxy-3-oxo-△^1,5^ analogs) was the step bottleneck in the whole synthetic strategy. The desired A-ring fused steroidal pyrazines could be obtained from compound **3** or **7** in quite low yields, without the α-ketoenol intermediates (**4** or **8**), even in harsh reaction conditions (sulfur and refluxing morpholine) [30]. However, the corresponding *α*-ketoenols might be prepared in complex reaction conditions, according to previous reports [35,36], such as in dry toluene at −25 °C for 1.5–4 h with *t*-BuOK (1.5 equiv.) and (3 equiv.) 18-crown-6 under dry O_2_, and easily accompanied by a byproduct through oxygenation at CFinally, the introduction of 4,4-dimethyl could not only maintain the activity of target compounds, but also be favorable for the efficient formation of α-ketoenols at room temperature. In addition, the 4,4-dimethyl group was widespread in sterols derived from the natural lanostane or cycloartane skeletons and was also included in triterpenoids which showed broad spectrum antitumor activity [37].

Through modifications of the steroidal D-ring and its C-20 side-chain, more than 35 steroid monomers with nanomolar IC_50_ values below 50 nM were synthesized by Banday’s group in 2010 [8] and by Mohareb’s group in 2012, 2013, and 2014 [12,13,14,15,16]. Additionally, about nine steroid monomers were reported by Ma’s group [17] that had less than 50 nM levels through 3-nitrogen-containing esterificated at the C-3 position based on special bufalin, which originally had excellent antitumor activity. However, the mechanism of their antitumor activity for steroid monomer derivatives still has not been deeply investigated, especially for compounds with nanomolar IC_50_ values. Only a few preliminary mechanisms were reported by Shi et al. and Liu et al. for potential synthetic derivatives with IC_50_ values below 1 μM. An androsten-17-(1’,3’,4’)-pyrazole (IC_50_ 0.53 μM) could cause Hela cell line apoptosis and arrest the cell cycle in the S phase [10]. Another steroidal spiro-oxindole (IC_50_ 0.71 μM) arrested the cell cycle in the G2/M phase [6]. Furthermore, for ring-condensed derivatives, the steroidal ring A/D-fused arylpyrazoles displayed moderate anticancer activity (4.0–5.0 μM) that might cause cell cycle arrest in the G2/M phase for T47D cells and MDA-MB-231cells, respectively [19]. In this work, compound **12n** could also cause cell cycle arrest in the G2/M phase.

Generally, there are currently two major apoptotic pathways used by cytotoxic drugs: one that begins with the ligation of death receptors and subsequently activates the initiator caspase-8, and another that involves the “activation” of the mitochondria pathway. For natural steroid monomers like *α*-solanine and *α*-chaconine, several studies have found that they could suppress the migration and invasion of cancer cells through the inhibition of PI3K/Akt and MAPK signaling pathways and also suppress metastasis by declining the NF-κB activity [11]. However, for steroid dimers, the ED_50_ /IC_50_ values could be below 1 μM for 20 natural cephalostatins, 19 ritterazines, and only 1 [1,2,4] triazolo [1,5-α] pyrimidine-based phenyl-linked steroid dimer [38,39]. These mechanical studies of cephalostatin 1 and the synthetic steroid dimer suggested that they could induce apoptosis, probably through the mitochondrial pathway, which was a distinctive feature of dimeric steroids and different from that of steroid monomers [23,39]. In summary, the anticancer mechanism still requires further research for satisfying the development of novel anticancer drugs, especially for synthetic fused derivatives with IC_50_ values below 1 *μ*M.

## 4. Materials and Methods

### 4.1. General

All chemicals and solvents were purchased from commercial sources (Chron Chemical Co., Ltd., Chengdu, China; Aladdin Industrial Co., Ltd., Shanghai, China) and used as received, except for THF and *t*-BuOH, which were distillated when needed. The ^1^H-NMR and ^13^C-NMR spectra were investigated using an Avance spectrometer (Bruker, Billerica, MA, USA) at 500 and 125 MHz. Chemical shifts were measured relative to the residual solvent peaks of CDCl_3_ and DMSO-*d*_6_, with tetramethylsilane (TMS) as the internal standard (Aladdin, Shanghai, China). High-resolution mass spectroscopy was undertaken using an AB SCIEX Triple TOF 5600^+^ spectrometer (AB SCIEX, Boston, MA, USA). Melting points were measured through capillary melting apparatus and uncorrected (SGW X-4, Shanghai, China). The column chromatography and thin-layer chromatography (TLC) were implemented on a silica gel 100–200 mesh and silica GF_254_ with ultraviolet detection purchased from Qingdao Marine Chemical Company (China). Four cell lines (MCF-7, PC-3, HepG2, and THLE-2) were obtained from the ATCC (Manassas, VA, USA) and were cultured using Ham’s F-12K (Wisent, Nanjing, China), DMEM medium (Hyclone Laboratories Inc., Logan, Utah, USA) and 10% FBS (Gibco&Invitrogen, Carlsbad, CA, USA). The optical density (OD) values were recorded using a multimode plate reader (PerkinElmer VICTOR Nivo, Turku, Finland). The biological samples were processed and analyzed by flow cytometry (CytoFLEX, California, United States). Molecular docking experiments were implemented using the MOE software (CCG, Quebec, Canada).

#### 4.1.1. Synthesis of 4,4-dimethyl-3-oxo-pregnen-5-ene-17-carboxylate (3)

The carboxylic acid compound **2** could be easily obtained and recrystallized from acetone, according to a previously described method [27]. The esterification reaction of **2** was also carried out as previously described [28]. The 4,4-dimethylsteroid **3** could be prepared following the reported method [29]. The ^1^H and ^13^C NMR spectra of compounds **2** and **3** are available in the section 2.2 of Supplementary Materials.

#### 4.1.2. Synthesis of 4,4-dimethyl-2-hydroxy-3-oxo-pregnen-1,5-diene-17β-carboxylate (4)

To a solution of **3** (7.2 g, 20.0 mmol) in *t*-BuOH (200 mL) and THF (5.0 mL), *t*-BuOK (4.50 g, 40.00 mmol) was added, and the mixture was stirred at 40 °C under O_2_ for 40 min. After the THF was removed, the residue was replenished with brine solution (80 mL). The mixture was extracted with EtOA, and the organic layer was washed with saturated NaCl solution, dried over Na_2_SO_4_, and concentrated. Then, the crude solid was purified through silica gel column chromatography (PE/EA, 10/1, v/v), to afford the *α*-ketoenol **4** (5.2 g, 69%) as a white solid [30]. m.p. 152−154 °C; ^1^H NMR (500 MHz, DMSO-*d*_6_) δ 8.27 (d, *J* = 8.8 Hz, 1H, OH), 6.00 (d, *J* = 8.8 Hz, 1H, H1), 5.57 (t, *J* = 3.5 Hz, 1H, H6), 3.60 (s, 3H, COOCH_3_), 2.37 (t, *J* = 9.3 Hz, 1H), 2.23 – 2.07 (m, 2H), 1.99 (td, *J* = 13.2, 6.6 Hz, 3H), 1.75 – 1.60 (m, 6H,), 1.29 (s, 3H), 1.24 (s, 6H, 2 × CH_3_), 1.21 (s, 2H), 0.63 (d, *J* = 4.0 Hz, 3H, CH_3_); ^13^C NMR (126 MHz, DMSO) δ 198.05, 173.50, 144.06, 143.89, 122.71, 120.78, 55.61, 54.13, 51.05, 48.10, 47.55, 43.33, 37.80, 37.41, 31.05, 30.79, 30.65, 24.40, 23.79, 23.27, 23.22, 20.37, 13.23; HRMS (ESI) *m/z* calcd for C_23_H_32_O_4_ [M + H]^+^ 373.2373, found 373.2357.

#### 4.1.3. General Procedure for the Preparation of Target Compounds **5a–5f**

To a solution of **4** (372.2 mg, 1.0 mmol) in anhydrous EtOH (20 mL), anhydrous magnesium sulfate (0.5 g, 4 eq.) and ethylene diamine (2 eq.) were added. The mixture was heated to reflux for 8 h and then filtered, and the filtrate was concentrated to provide the crude white solid. The residue was purified by flash column chromatography on silica gel (petroleum ether/EtOAc 3:1, *v*/*v*) to afford the crude product, which was used for the next reaction, without further purification. Immediately, the crude product was dissolved in ethylene glycol (10.0 mL), an aqueous solution of KOH (1.0 mL, 40% *m*/*v*) was added, and the resulting suspension was heated at 130 °C under reflux for 20 min until a clear red solution was obtained [29]. After being cooled down to r.t., saturated brine (20 mL) and HCl 10% (10 mL) were added to adjust the pH to 3, and the precipitate was extracted with EtOAc. The organic layer was washed with saturated brine, dried over anhydrous Na_2_SO_4_, and evaporated in vacuo. The crude product was purified by column chromatography on silica (petroleum ether/EtOAc gradient, 3:1 to 1:1), to yield the desired A-ring fused steroidal pyrazine **5a**. White solid; yield: 67%; m.p. 270–292 °C. ^1^H NMR (500 MHz, DMSO-*d*_6_) δ 11.93 (s, 1H, COOH), 8.48 (d, *J* = 2.4 Hz, 1H, pyrazine), 8.34 (d, *J* = 2.6 Hz, 1H, pyrazine), 5.81 (dt, *J* = 7.1, 3.4 Hz, 1H, H6), 2.97 (d, *J* = 15.6 Hz, 1H, H1), 2.64 (d, *J* = 15.6 Hz, 1H, H1), 2.43 (d, *J* = 14.9 Hz, 1H), 2.30 (t, *J* = 9.1 Hz, 1H), 2.15 (dt, *J* = 17.3, 5.2 Hz, 1H), 2.09 – 1.93 (m, 2H), 1.77 – 1.63 (m, 4H), 1.54 (s, 3H, CH_3_), 1.52 (d, *J* = 3.2 Hz, 1H), 1.27 (s, 3H, CH_3_), 1.25 – 1.17 (m, 4H), 0.70 (s, 3H, CH_3_), 0.65 (s, 3H, CH_3_); ^13^C NMR (126 MHz, DMSO) δ 174.65, 158.32, 150.32, 148.64, 142.61, 141.48, 120.09, 55.56, 54.46, 48.20, 43.13, 43.00, 40.86, 37.63, 37.19, 33.34, 31.56, 31.33, 31.08, 24.03, 23.26, 20.48, 20.20, 13.HRMS (ESI) *m/z* calcd for C_24_H_32_N_2_O_2_ [M + H]^+^ 381.2536, found 381.2538.

Target derivatives **5b–5f** were achieved using the same protocols by combining *o*-phenylenediamine (OPD) or substituted OPD with key intermediate product (**4)**, but without anhydrous MgSO_4_, in refluxing ethanol, and then deprotecting through adding KOH, with 50%–70% yields for two steps. Those novel derivatives were identified by ^1^H NMR, ^13^C NMR, and HRMS(ESI) spectra (see section 2.3 and 4 in Supplementary Materials).

#### 4.1.4. Synthesis of 4,4-dimethyl-2-hydroxy-3-oxo-pregnen-1,5-diene-20-ethylene ketal (8)

Progesterone 20-ethylene ketal (**6**) could be prepared according to the reported methods [31]. Then 4,4-dimethyl-3-oxo-pregnen-1,5-diene-20-ethylene ketal (**7**) was synthesized following the reported methods [32], and without HCl aqueous solution to adjust the pH, in case of losing the 20-ethylene ketal. These novel compounds **6** and **7** were identified by ^1^H NMR, ^13^C NMR, and HRMS(ESI) spectra (see section 2.4, 2.5 and 4 in Supplementary Materials).

The *α*-ketoenol product **8** was achieved using the above procedure of compound The configuration of compound **8** was confirmed by X-ray data (Scheme 1, CCDC: 1831372). White solid; yield: 65%; m.p. 148−150°C; ^1^H NMR (500 MHz, DMSO-*d*_6_) δ 8.32 (s, 1H, OH), 6.03 (s, 1H, H1), 5.59 (d, *J* = 4.6 Hz, 1H, H6), 3.97 – 3.87 (m, 2H, -OCH_2_CH_2_O-), 3.82 (dt, *J* = 17.7, 7.0 Hz, 2H, -OCH_2_CH_2_O-), 2.16 – 2.05 (m, 2H, H7), 1.77 – 1.50 (m, 8H), 1.32 (s, 3H, CH_3_), 1.27 (s, 3H, CH_3_), 1.23 (s, 6H, 2 × CH_3_), 1.20 – 1.02 (m, 4H), 0.78 (s, 3H, CH_3_); ^13^C NMR (125 MHz, CDCl_3_) δ 199.40, 143.84, 142.83, 123.11, 122.10, 111.86, 65.19, 63.22, 58.00, 57.06, 48.23, 47.25, 41.75, 39.19, 38.47, 32.08, 31.43, 30.55, 24.59, 23.97, 23.62, 23.20, 23.01, 20.77, 13.01; HRMS (ESI) *m/z*: calcd for C_25_H_36_O_4_ [M + H]^+^ 401.2686, found 401.2678.

#### 4.1.5. General Procedure for the Conversion of **8** to **9a–9o** and **10a–10o**

To a solution of **8** (400 mg, 1.0 mmol,) in dry EtOH (10 mL), anhydrous magnesium sulfate (0.5 g, 4 eq.) and ethylene diamine (2 eq.) were added, and the mixture was heated to reflux for 8 h. The mixture was filtered and the filtrate was concentrated to give a white mixture. The residue was purified by flash chromatography on silica gel (petroleum ether/EtOAc 2:1, *v*/*v*) to afford a white solid, which was used for the next reaction, without further purification. The crude product was then dissolved in MeOH/DCM (5 mL, 4:1, *v*/*v*), three drops of 3 M HCl were added, and the clear solution was stirred for 10 min at r.t. After being adjusted to pH = 7 by NaHCO3, the solvents were removed and the residue was purified by column chromatography (PE:EA = 3:1), to afford **9a**. Furthermore, to a solution of **9a** in dry MeOH/THF (10 mL, 2:1, *v*/*v*), NaBH_4_ (80 mg) at 0 °C was added portion-wise, and the resulting mixture was stirred at 0 °C for 15 min. The reaction was quenched (saturated aqueous NH_4_Cl, 10 mL), the solvent was evaporated, and the residue was dissolved in water and EtOAc (40 mL, 1:1, *v*/*v*). The organic layer was extracted with EtOAc (30 mL × 3), dried over anhydrous Na_2_SO_4_, and evaporated in vacuo. The crude product was purified by silica column chromatography (DCM/MT = 20:1), to afford compound **10a**, white solid, yield: 67%; m.p. 150–152 °C; ^1^H NMR (500 MHz, Chloroform-*d*) δ 8.42 (d, *J* = 2.7 Hz, 1H, pyrazine), 8.27 (d, *J* = 2.6 Hz, 1H, pyrazine), 5.79 (dd, *J* = 5.3, 2.5 Hz, 1H, H6), 3.75 (dt, *J* = 12.0, 5.8 Hz, 1H, H20), 3.10 (d, *J* = 15.7 Hz, 1H, H1), 2.64 (d, *J* = 15.6 Hz, 1H, H1), 2.29 – 2.11 (m, 2H), 1.79 – 1.66 (m, 4H), 1.61 (s, 3H, CH_3_), 1.53 (td, *J* = 13.1, 3.9 Hz, 2H), 1.38 (d, *J* = 9.0 Hz, 1H), 1.33 (s, 3H, CH_3_), 1.25 (p, *J* = 4.7 Hz, 3H), 1.21 (d, *J* = 8.5 Hz, 2H), 1.16 (d, *J* = 6.1 Hz, 3H, CH_3_), 0.80 (s, 6H, 2 × CH_3_); ^13^C NMR (125 MHz, CDCl_3_) δ 159.76, 151.36, 149.14, 142.65, 141.31, 120.77, 70.64, 58.55, 56.48, 49.06, 43.99, 42.46, 41.41, 39.99, 37.86, 33.69, 32.11, 31.86, 31.44, 25.84, 24.63, 23.88, 21.12, 20.56, 12.58; HRMS (ESI) *m/z* calcd for C_25_H_36_N_2_O [M + H]^+^ 381.2900, found 381.2885.

Compounds **9b–9o** and **10b–10o** were synthesized similarly using the above procedure, but without MgSOCollectively, the compound **8** was treated with *o*-phenylenediamine (OPD, 1.5 eq.) and substituted OPD (1.5 eq.) to get the A-ring fused steroidal pyrazines **9b–9o** (24–71% yields for two steps), which were then converted into the corresponding target derivatives **10b-o** through the removal of ketal protection and reduction (70–89%). Furthermore, this scaffold **10g** was confirmed by X-ray single crystal diffraction (CCDC: 1971468). Those novel compounds were identified by ^1^H NMR, ^13^C NMR, and HRMS(ESI) spectra (see section 2.6 and 4 Supplementary Materials).

#### 4.1.6. General Procedure for the Conversion of **8** to **11a–11o** and **12a–12o**

The 2,3-diaminopropanamides (**X**) could be prepared by using the standard coupling reagent (DIPEA/HBTU), according to published literature [40] and were then used directly to trigger A-ring condensation with *α*-ketoenols intermediate Furthermore, the ring-closing reaction was carried out in the same condition as that of compound **10b**, and resulted in the formation of **11a–11o** (31–64% for two steps), which then changed to the corresponding target products **12a–12o** in 75–96% yields (Scheme 2). This scaffold of **11i** containing the ethylene ketal group was identified by X-ray single crystal diffraction (CCDC, 1836746, see Scheme 2). Those novel compounds were identified by ^1^H NMR, ^13^C NMR, and HRMS(ESI) spectra (see section 2.7 and 4 in Supplementary Materials).

### 4.2. The X-ray Structure of Three Representative Compounds

X-ray-quality crystal of compounds **8** and **10g** was obtained from pure acetone after 3 days and gained from DCM/MT (1:1) after 5 days. X-ray-quality crystal of the compound **11i** containing the ethylene ketal protecting group was obtained from cyclohexane/acetone (3:1) after 3 days. A suitable crystal was selected on a SuperNova, Dual, Cu at zero, AtlasS2 diffractometer (Rigaku Corporation, Tokyo, Japan). The crystal was kept at 100.00(10) K during data collection. The atomic coordinates have been deposited at the Cambridge Crystallographic Data Center (CCDC).

### 4.3. In Vitro Cytotoxicity

Thirty-six novel steroidal A-ring fused pyrazine hybrids were evaluated by Cell Counting Kit-8 (CCK-8) assays [41], to evaluate their in vitro antiproliferative activities on three human cancer cell lines and one normal cell line: MCF-7 (breast carcinoma), PC-3 (prostate carcinoma), HepG2 (liver hepatocellular carcinoma), and THLE-2 (normal lung cell), with 5-fluorouracil as the positive control.

Cells (3 × 10^3^ cells per well) seeded into 96-well plates (Costar, Shanghai, China) were incubated with Ham’s F-12K containing various concentrations of steroidal pyrazines (**5a**–**5f, 10a**–**10o**, and **12a**–**12o**) for 48 h at 37 °C and 5% COThen, 10 μL of CCK-8 reagent (Beyotime, Shanghai, China) dissolved in PBS (Phosphate buffered saline) was added per well, and continuously incubated at 37 °C for 1 h. Finally, the absorbance of samples taken from each well was measured on a microplate reader at 450 nm. The antiproliferative activities were expressed as inhibitory activity and IC_50_ values calculated using GraphPad Software 5.0 (GraphPad Software, San Diego, CA, USA), which were the mean values of three independent tests and are shown in Table 1 and Table 2, respectively. The formula used for cell inhibitory rates was (1 - sample absorption/vehicle absorption) × 100%.

### 4.4. Calculation of the Molecular Properties of All Derivatives

The molecular properties were calculated using the Molinspiration tool (http://www.molinspiration.com) [33].

### 4.5. Giemsa Staining for the Detection of Morphological Features of Apoptosis

Giemsa stain was used to assess the morphological changes of PC-3 cells, according to the standard Giemsa procedure established by the supplier (Solarbio, Beijng, China). Briefly, PC-3 cells were plated at 3 × 10^3^ cells/well into a 96-well plates. PC-3 cells after treatment with **12n** for 48 h were fixed with methanol (75%) for 10 min at room temperature. Then, the cells were stained with Giemsa (diluted with phosphate buffer), observed, and photographed under the microscope at 200 × (Olympus Corporation, Tokyo, Japan ) [42]. PC-3 cell lines that did not deal with the compound 12n were used as the control.

### 4.6. Cell Apoptosis Assay

The cell apoptosis was evaluated with Annexin V-FITC/PI dual staining (Beyotime, Shanghai, China), as previously described [43]. PC-3 cells (1 × 10^6^ cells per well) seeded into 6-well plates (Costar, Shanghai, China) were incubated with Ham’s F-12K containing various concentrations (0.2, 1.0, and 5.0 μM) of compound **12n** for 48 h. The biological samples were analyzed using flow cytometry (CytoFLEX, CA, USA).

### 4.7. Cell Cycle Assay

The cell cycle was examined by PI single staining (Beyotime, Shanghai, China), as previously described [43]. PC-3 cells (1 × 10^6^ cells per well) seeded into 6-well plates were incubated with Ham’s F-12K containing various concentrations (0.2, 1.0, and 5.0 μM) of **12n** for 48 h. The samples were analyzed using flow cytometry for DNA content (CytoFLEX, California, United States).

### 4.8. Molecular Docking Study

The X-ray structures and data of protein targets were obtained from the protein data bank (PDB) (www.rcsb.org): CYP17A1 (17a-hydroxylase/C17,20-lyase, PDB ID: 6CIZ, 6CHI, 6CIR, 5UYS, 4NKZ, 4NKX, 4NKV, 4NKW, and 3RUK) and CYP19A1 (aromatase, PDB ID: 3S7S).

All designed compound structures were energy-minimized using the ChemdBio software and saved in an sdf format. Molecular docking was implemented with the default settings using the MOE software, referring to the published reports [34,44,45]. The numbers of placement poses were 20 for flash docking and 300 for molecular interaction.

## 5. Conclusions

In summary, 36 novel steroidal A-ring fused pyrazines, belonging to two different series, were efficiently prepared with key *α*-ketoenol intermediates. This facile ring-closing strategy may provide a novel and promising avenue for the cycloaddition reaction of the steroidal skeleton. The most active compound, **12n**, had a lower IC_50_ value (0.93 μM) and a higher SI value (28.71) compared to human prostatic cells (PC-3). Furthermore, **12n** could induce apoptosis of PC-3 cells and cause cell cycle arrest in the G2/M phase. The molecular docking study suggested that **12n** could be used as an inhibitor of CYP17A1 (6CIZ). Collectively, **12n** may serve as a new candidate for anticancer drug discovery. In addition, the anticancer mechanism still requires further research for satisfying the development of novel anticancer drugs, especially for synthetic fused steroidal derivatives.

## Figures and Tables

**Table 1:** Inhibitory activity of the derivatives of **5a–f**, **10a–o**, and **12a–o** in terms of the growth of three human cancer cell lines and one human normal cell line.

Structures	No.	Substituents	Inhibitory Ratio (Mean% ± SD%)^a^			
			MCF-7	PC-3	HepG2	THLE-2
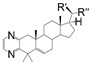	**5a**	R’ = O, R’’ = OH	3.09 ± 1.05	25.13 ± 2.64	17.9 ± 1.16	NA^b^
	**10a**	R’ = OH, R’’ = Me	4.99 ± 3.09	**NA**	6.2 ± 5.25	NA
	**5b**	(R_1_ = R_2_ = H)	NA	12.54 ± 12.90	7.24 ± 5.83	12.64 ± 1.22
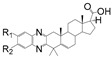	**5c**	(R_1_ = Br, R_2_ = H)	6.86 ± 7.46	20.73 ± 12.48	NA	12.53 ± 0.90
	**5d**	(R_1_ = H, R_2_ = Br	12.44 ± 0.30	**40.20 ± 0.64**	NA	2.39 ± 0.65
	**5e**	(R_1_= OH, R_2_ = H)	6.57 ± 0.09	NA	NA	NA
	**5f**	(R_1_= H, R_2_ = OH)	18.38 ± 13.06	NA	24.89 ± 2.30	NA
	**10b**	(R_1_ = R_2_ = H)	9.95 ± 0.55	12.54 ± 12.90	7.24 ± 5.83	4.35 ± 2.27
	**10c**	(R_1_ = F, R_2_ = H)	3.85 ± 1.97	24.87 ± 2.12	NA	NA
	**10d**	(R_1_ = H, R_2_ = F)	21.17 ± 9.12	**52.78 ± 0.86**	22.39 ± 4.89	NA
	**10e**	(R_1_ = Cl, R_2_ = H)	NA	**44.77 ± 3.26**	25.64 ± 2.51	NA
	**10f**	(R_1_ = H, R_2_ = Cl)	23.87 ± 1.87	**59.59 ± 1.29**	**32.57 ± 0.41**	NA
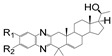	**10g**	(R_1_ = Br, R_2_ = H)	NA	NA	11.67 ± 2.56	NA
	**10h**	(R_1 =_ H, R_2_ = Br)	21.25 ± 3.73	21.72 ± 1.27	13.08 ± 0.68	2.39 ± 0.07
	**10i**	(R_1_ = OH, R_2_ = H)	17.92 ± 6.61	14.16 ± 0.87	NA	NA
	**10j**	(R_1_ = H, R_2_ = OH)	18.6 ± 0.92	**33.07 ± 0.34**	NA	NA
	**10k**	(R_1_ = OMe, R_2_ = H)	11.24 ± 0.49	NA	29.32 ± 5.38	3.54 ± 2.16
	**10l**	(R_1_ = H, R_2_ = OMe)	**41.63 ± 1.28**	30.91 ± 1.74	**33.30 ± 1.13**	22.2 ± 2.72
	**10m**	(R_1_ = NO_2_, R_2_ = H)	14.71 ± 0.25	15.98 ± 0.57	18.19 ± 7.46	NA
	**10n**	(R_1_ = H, R_2_ = NO_2_)	19.98 ± 2.56	16.59 ± 3.72	18.72 ± 2.69	NA
	**10o**	(R_1_ = R_2_ = Cl)	NA	NA	NA	ND^c^
	**12a**	(R = phenyl)	NA	24.19 ± 0.94	12.54 ± 12.90	NA
	**12b**	(R = *o*-Me-Phenyl)	8.12 ± 3.17	20.79 ± 7.04	15.23 ± 5.61	5.15 ± 1.79
	**12c**	(R = *m*-Me phenyl)	3.57 ± 1.69	19.93 ± 0.43	7.02 ± 3.10	NA
	**12d**	(R = *p*-Me phenyl)	24.96 ± 3.43	22.60 ± 2.25	16.29 ± 3.72	18.25 ± 6.76
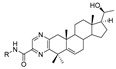	**12e**	(R = *o*-F phenyl)	3.28 ± 1.90	**47.01 ± 3.47**	17.61 ± 3.19	14.01 ± 1.47
	**12f**	(R = *m*-F phenyl)	0.80 ± 3.31	**33.21 ± 4.68**	12.79 ± 4.26	NA
	**12g**	(R = *p*-F phenyl)	33.38 ± 5.35	**66.76 ± 1.08**	NA	17.63 ± 1.39
	**12h**	(R = *o*-Cl phenyl)	NA	**41.70 ± 4.79**	21.95 ± 2.05	NA
	**12i**	(R = *m*-Cl phenyl)	20.22 ± 0.52	**35.91 ± 2.97**	15.94 ± 2.93	NA
	**12j**	(R = *p*-Cl phenyl)	24.88 ± 4.84	**51.10 ± 1.02**	23.05 ± 1.27	NA
	**12k**	(R = *o*-OMe phenyl)	17.66 ± 1.01	**32.85 ± 1.19**	12.89 ± 0.43	4.71 ± 1.25
	**12l**	(R = *m*-OMe phenyl	13.83 ± 2.32	26.88 ± 1.71	12.02 ± 1.67	NA
	**12m**	(R = *p*-OMe phenyl)	19.99 ± 2.33	**48.58 ± 0.08**	20.27 ± 3.60	5.68 ± 1.90
	**12n**	(R = *β*- phenethyl)	**40.78 ± 1.72**	**74.91 ± 1.22**	**55.37 ± 1.00**	19.31 ± 1.81
	**12o**	(R = cyclohexyl)	15.08 ± 1.25	NA	24.05 ± 2.34	NA
**5-FU^d^**			47.90 ± 1.67	34.98 ± 1.46	47.72 ± 1.30	51.78 ± 0.69

The bold numbers represent the better activities of the compounds; cell lines: MCF-7 (breast), PC-3 (prostatic), HepG2 (hepatoma), and THLE-2 (normal human cells). ^a^ Results are expressed as a percentage of inhibition ± standard error, tested after treatment with compounds at the concentration of 20 μM for 48 h; all data are the mean values of three independent experiments; ^b^ NA, no activity; ^c^ ND, not determined; ^d^ 5-FU, 5-fluorouracil, which was used as a positive control.

**Table 2:** IC_50_ of the derivatives **12g** and **12n** with PC-3 and THLE-2 cell lines.

Compounds	PC-3 (IC_50_, μM)^a^	THLE-2 (IC_50_, μM)	SI^b^
**12g**	6.88 ± 0.04	14.69	2.14
**12n**	0.93 ± 0.02	26.70	28.71
**5-FU**	22.35 ± 0.41	12.86	0.58

^a^ IC_50_ value was obtained on the basis of triplicate assay results. ^b^ SI: Selectivity index = (IC_50_ of THLE-2)/(IC_50_ of /PC-3).

**Table 3:** Calculated molecular properties of all the derivatives.

NO.	miLogP^a^	TPSA^b^	nON^c^	nOHNH^d^	Vol^e^	NO.	miLogP	TPSA	nON	nOHNH	Vol
**5a**	5.25	63.08	4	1	370.66	**12b**	7.27	75.11	5	2	504.42
**10a**	5.74	46.01	3	1	385.06	**12c**	7.29	75.11	5	2	504.42
**5b**	6.75	63.08	4	1	414.65	**12d**	7.31	75.11	5	2	504.42
**5c, 5d^f^**	7.54	63.08	4	1	432.54	**12e**	6.98	75.11	5	2	492.79
**5e, 5f**	6.25	83.31	5	2	422.67	**12f**	7.01	75.11	5	2	492.79
**10b**	7.24	46.01	3	1	429.06	**12g**	7.03	75.11	5	2	492.79
**10c,10d**	7.38	46.01	3	1	433.99	**12h**	7.50	75.11	5	2	501.39
**10e, 10f**	7.89	46.01	3	1	422.59	**12i**	7.52	75.11	5	2	501.39
**10g, 10h**	8.02	46.01	3	1	446.94	**12j**	7.54	75.11	5	2	501.39
**10i, 10j**	6.74	66.24	4	2	437.07	**12k**	6.88	84.34	6	2	513.40
**10k, 10l**	7.27	55.25	4	1	454.60	**12l**	6.90	84.34	6	2	513.40
**10m, 10n**	7.17	91.84	6	1	452.39	**12m**	6.92	84.34	6	2	513.40
**10o**	8.40	46.01	3	1	456.13	**12n**	6.97	75.11	5	2	521.46
**12a**	6.87	75.11	5	2	487.86	**12o**	7.07	75.11	5	2	506.44

a, Octanol-water partition coefficient (LogP). b, Topologic polar surface area (TPSA) [Å^2^]. c, Sum of O and N H-bond acceptors (nON). d, Sum of OH and NH H-bond donors (nOHNH). e, Molecular volume (Vol.) [Å^3^]. f, The isomers such as **5c** and **5d** had the same calculated molecular properties.

**Table 4:** The percentage of cell apoptosis of PC3 cells after 48 h through treatment with **12n** at different concentrations (0.2, 1.0, and 5.0 μM).

Compounds	Concentration (*μ*M)	Percentage of Cell Apoptosis (%)			
		Normal Living Cell	Early Apoptosis	Late Apoptosis	Necrosis
**12n**	0.2	89.20	0.76	7.90	2.15
	1.0	73.17	4.55	19.07	3.21
	5.0	54.13	3.64	35.93	6.30
Control	1% DMSO	92.18	0.64	5.13	2.06

**Table 5:** Distribution of the cell cycle after treatment with **12n** in PC-3 cells.

Compounds	Concentration (μM)	Percentage of Cell Cycles (%)		
		G1	S	G2/M
**12n**	0.2	46.65	37.26	16.09
	1.0	23.03	30.78	46.19
	5.0	55.73	0.00	44.27
Control	**1%** DMSO	49.62	34.69	15.69

**Table 6:** Possible cancer-associated protein targets for compound **12n** screened by fast docking against the protein data bank (PDB) database.

PDB	Scores ^a^	PDB	Scores	PDB	Scores
6CIZ ^b^	−11.72	3S7S ^c^	−10.49	4NKX	−10.20
4NKZ	−11.02	4NKV	−10.36	3RUK	−10.08
5UYS	−10.81	4NKW	−10.32		
6CIR	−10.52	6CHI	−10.30		

^a^ Unit is kcal/mol; ^b^ the crystal structure of CYP17A1 has an Abiraterone-specific active site; ^c^ the crystal structure of CYP19A1 has an androstenedione-specific active site.

**Table 7:** Binding affinity scores of the compounds **5a−f, 10a−o**, and **12a−o** with 6CIZ.

No.	Substituents	Scores	No.	Substituents	Scores
**5a**	R’ = O, R’’ = OH	−8.65	**10n**	(R_1_ = H, R_2_ = NO_2_)	−10.66
**5b**	(R_1_ = R_2_ = H)	−9.60	**10o**	(R_1_ = R_2_ = Cl)	−10.44
**5c**	(R_1_ = Br, R_2_ = H)	−9.60	**12a**	(R = phenyl)	−11.03
**15d**	(R_1_ = H, R_2_ = Br	−9.75	**12b**	(R = *o*-Me-Phenyl)	−10.73
**5e**	(R_1_ = OH, R_2_ = H)	−9.63	**12c**	(R = *m*-Me phenyl)	−11.29
**5f**	(R_1_ = H, R_2_ = OH)	−9.64	**12d**	(R = *p*-Me phenyl)	−11.21
**10a**	R’ = OH, R’’ = Me	−8.96	**12e**	(R = *o*-F phenyl)	−10.56
**10b**	(R_1_ = R_2_ = H)	−9.68	**12f**	(R = *m*-F phenyl)	−10.71
**10c**	(R_1_ = F, R_2_ = H)	−9.80	**12g**	(R = *p*-F phenyl)	−11.16
**10d**	(R_1_ = H, R_2_ = F)	−9.81	**12h**	(R = *o*-Cl phenyl)	−10.99
**10e**	(R_1_ = Cl, R_2_ = H)	−10.06	**12i**	(R = *m*-Cl phenyl)	−10.80
**10f**	(R_1_ = H, R_2_ = Cl)	−10.17	**12j**	(R = *p*-Cl phenyl)	−11.00
**10g**	(R_1_ = Br, R_2_ = H)	−10.07	**12k**	(R = *o*-OMe phenyl)	−11.29
**10h**	(R_1 =_ H, R_2_ = Br)	−10.14	**12l**	(R = *m*-OMe phenyl	−11.49
**10i**	(R_1_ = OH, R_2_ = H)	−9.97	**12m**	(R = *p*-OMe phenyl)	−11.32
**10j**	(R_1_ = H, R_2_ = OH)	−10.01	**12n**	(R = *β*- phenethyl)	−11.87
**10k**	(R_1_ = OMe, R_2_ = H)	−8.76	**12o**	(R = cyclohexyl)	−11.48
**10l**	(R_1_ = H, R_2_ = OMe)	−9.15	Abiraterone acetate*		−8.91
**10m**	(R_1_ = NO_2_, R_2_ = H)	−10.25			

* Abiraterone acetate was used as the control.

## Supplementary Materials

Supplementary materials can be found at https://www.mdpi.com/1422-0067/21/5/1665/s1. The following are available online: The NMR spectra of all compounds and the HRMS spectra of representative compounds.

## Abbreviations

OPDs	*o*-Phenylenediamines
CCDC	Cambridge crystallographic data centre
EG	Ethylene glycol
(EtO)4Si	Tetraethyl orthosilicate
CCK8	Cell counting kit-8
MOE	Molecular operating environment
CYP17A1	Cytochrome P450 17A1
miLogP	LogP calculated by Molinspiration tool
TPSA	Topologic polar surface area
