# Supplementary material for: Design, Synthesis, and Biological Evaluation of Two Series of Novel A-Ring Fused Steroidal Pyrazines as Potential Anticancer Agents
Source: Int J Mol Sci. 2020 Feb 28;21(5):1665. doi: 10.3390/ijms21051665 (PMC7084598; doi:10.3390/ijms21051665)
Supplement: Supplementary file 1 [file ijms-21-01665-s001.pdf]

Supplementary Materials

Design, synthesis and biological evaluation of two series of novel A-ring fused steroidal pyrazines as potential anticancer agents

Shijun Wang [1], Xiaorong Yuan [1], Hao Qian [1], Na Li [2,*] and Junru Wang [1,*]

1 College of Chemistry & Pharmacy, Northwest A&F University, 22 Xinong Road, Yangling 712100, Shannxi, China; 444795415@nwafu.edu.cn (S.-J.W.); a1045244699@126.com (X.-R.Y.); a1809762418@126.com (H.Q.)
2 College of Food Science and Technology, Northwest University, Xi'an 710127, Shannxi, China
* Correspondence: nwuln@nwu.edu.cn (N.L); wangjunru@nwsuaf.edu.cn (J.-R.W.) Tel./fax: +86-29-8709-2662

Received: 30 January 2020; Accepted: 26 February 2020; Published: date

Contents

1. X-ray Structure of Compound **10g** S1
2. The Identification of Compound **2-3, 5b-5f, 6, 9b-9o, 10b-10o, 11a-11o** and **12a-12o** ... S2
   2.1 *Compound **2*** ... S2
   2.2 *Compound **3*** ... S2
   2.3 *Compound **5b-5f**.* ... S2
   2.4 *Compound **6*** ... S3
   2.5 *Compound **7*** ... S3
   2.6 *Compound **9b-9o** and **10b-10o*** ... S4
   2.7 *Compound **11a-11o** and **12a-12o*** ... S8
3. The NMR Spectra of Compounds **2-12** ... S14
   3.1. The ^{1}H NMR and ^{13}C NMR Spectra of Compound **2-4** ... S14
   3.2. The ^{1}H NMR and ^{13}C NMR Spectra of Compound **5a-5f** ... S16
   3.3. The ^{1}H NMR and ^{13}C NMR Spectra of Compound **6-8** ... S23
   *3.4. The ^{1}H and ^{13}C NMR Spectra of Compound **9a-9o** and the ^{1}H NMR Spectra of Compound **10a-10o*** ... S26
   *3.5. The ^{1}H and ^{13}C NMR Spectra of Compound **11a-11o** and the ^{1}H NMR Spectra of Compound **12a-12o*** . S47
4. The HRMS(ESI+) Spectra of Some Representative Compounds ... S70

1. X-ray Structure of Compound **10g**

X-ray quality crystal of compound **10g** was acquiredd from the DCM:MT = 1:1 solution after 3 days. A white crystal was selected on a SuperNova, Dual, Cu at zero, AtlasS2 diffractometer, The crystal was kept at 100.00(10) K during data collection. The atomic coordinates have been deposited at the Cambridge Crystallographic Data Center (CCDC) with CCDC number 1971468. The absolute configuration of **10g** was identifieded by X-rays using CuKα radiation (λ=1.54184 Å), and was reliable with the Flack parameter down to 0.013(6). Generally, there were two independent molecules in a crystal unit for **10g**, and the structure of a single

molecule was re-solved and generated by ShelXle software.

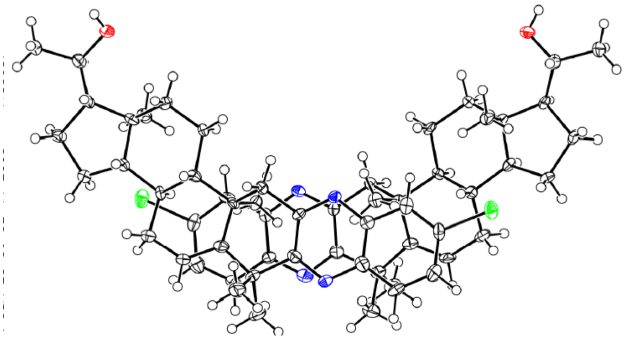

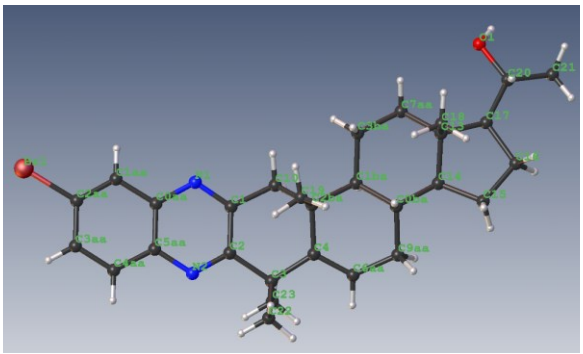

2. The Identification of Compound **2-3, 5b-5f, 6, 9b-9o, 10b-10o, 11a-11o** and **12a-12o**

2.1 *Compound* ***2***

Compound **2:** The carboxylic acid compound could be easily obtained according to reported literature[51] and recrystallized by acetone to provide white solid **2** (76%). m.p. 244–250°C; ^{1}H NMR (500 MHz, DMSO-*d*$_6$) δ 11.90 (s, 1H, COOH), 5.62 (s, 1H, H4), 2.45 – 2.34 (m, 2H, H2), 2.31 – 2.19 (m, 2H, H6), 2.19 – 2.11 (m, 1H, H17), 2.01 – 1.94 (m, 3H), 1.79 (d, *J* = 12.6 Hz, 1H), 1.74 – 1.68 (m, 1H), 1.62 (td, *J* = 14.4, 14.0, 7.8 Hz, 2H), 1.58 – 1.47 (m, 2H), 1.36 (tt, *J* = 13.2, 6.5 Hz, 1H), 1.29 – 1.18 (m, 2H), 1.14 (s, 3H, CH_3), 1.08 (td, *J* = 11.5, 7.0 Hz, 1H), 0.95 (tdd, *J* = 15.0, 12.6, 4.1 Hz, 2H), 0.67 (s, 3H, CH_3); ^{13}C NMR (126 MHz, DMSO) δ 197.88, 174.59, 170.76, 123.17, 54.66, 54.44, 53.16, 43.00, 38.17, 37.58, 35.15, 35.03, 33.58, 31.96, 31.63, 23.99, 23.20, 20.42, 16.86, 13.10; HRMS (ESI) *m/z* calcd for $C_{20}H_{28}O_3$ $[M + H]^+$, 317.2111, found 317.2109.

2.2 *Compound* ***3***

White solid; yield: 65%; m.p. 124–126°C; ^{1}H NMR (500 MHz, Chloroform-*d*) δ 5.54 (dd, *J* = 5.3, 2.5 Hz, 1H, H6), 3.66 (s, 3H, $COOCH_3$), 2.55 (ddd, *J* = 18.8, 8.4, 1.7 Hz, 1H, H2), 2.45 (ddd, *J* = 19.0, 11.3, 8.1 Hz, 1H,H2), 2.34 (t, *J* = 9.4 Hz, 1H), 2.12 (ddd, *J* = 14.8, 8.0, 3.8 Hz, 2H), 2.07 – 1.95 (m, 2H), 1.81 (dtd, *J* = 13.7, 9.6, 6.5 Hz, 1H), 1.74 – 1.58 (m, 4H), 1.50 (qd, *J* = 10.7, 5.4 Hz, 1H), 1.41 – 1.34 (m, 1H), 1.33 – 1.25 (m, 2H), 1.22 (s, 6H, 2 × CH_3), 1.17 – 1.02 (m, 2H), 0.84 (s, 3H, CH_3), 0.66 (s, 3H, CH_3); ^{13}C NMR (126 MHz, $CDCl_3$) δ 216.60, 174.55, 150.06, 119.79, 56.31, 55.28, 51.35, 49.04, 48.78, 44.09, 38.30, 37.25, 33.79, 32.26, 31.78, 31.48, 30.30, 27.40, 24.59, 23.82, 21.26, 19.46, 13.50; HRMS (ESI) *m/z* calcd for $C_{23}H_{34}O_3$ $[M + H]^+$ 359.2581, found 359.2580.

2.3 *Compound* ***5b-5f****.*

5b: White solid; yield: 65%; m.p. 295-297°C; ^{1}H NMR (500 MHz, DMSO-*d*$_6$) δ 11.97 (s, 1H, COOH), 8.02 (ddd, *J* = 15.8, 6.3, 3.5 Hz, 2H, C_6H_4), 7.77 (dt, *J* = 6.6, 3.7 Hz, 2H, C_6H_4), 5.99 – 5.72 (m, 1H, H6), 3.21 (dd, *J* = 15.6, 6.5 Hz, 1H), 2.89 (d, *J* = 15.4 Hz, 1H), 2.34 (t, *J* = 9.2 Hz, 1H), 2.20 (dt, *J* = 17.9, 5.4 Hz, 1H), 2.09 (d, *J* = 12.3 Hz, 2H), 1.88 (dt, *J* = 9.7, 5.6 Hz, 1H), 1.82 – 1.75 (m, 3H), 1.74 (s, 3H, CH_3), 1.56 (qd, *J* = 11.1, 10.5, 6.1 Hz, 1H), 1.46 (dd, *J* = 12.5, 5.0 Hz, 1H), 1.37 (s, 3H, CH_3), 1.33 – 1.28 (m, 1H), 1.24 (s, 3H), 0.68 (s, 6H, 2 × CH_3); ^{13}C NMR (126 MHz, DMSO) δ 175.16, 160.60, 152.79, 149.13, 141.86, 140.85, 129.61, 129.43, 129.01, 128.55, 120.60, 56.02, 54.97, 54.11, 51.40, 48.52, 44.75, 44.06, 43.52, 42.37, 38.17, 37.92, 34.74, 31.58, 24.51, 23.77, 20.69, 13.56; HRMS (ESI) *m/z* calcd for $C_{28}H_{34}N_2O_2$ $[M + H]^+$ 431.2693, found 431.2687.

5c: White powder, yield 30%; m.p. 260-262°C; ^{1}H NMR (500 MHz, Chloroform-*d*) δ 12.59 (s, 1H, COOH), 8.38 (d, *J* = 8.9 Hz, 1H, Br-C_6H_3), 8.25 (d, *J* = 2.1 Hz, 1H, Br-C_6H_3), 7.78 (dd, *J* = 9.0, 2.2 Hz, 1H, Br-C_6H_3), 5.85 (dd, *J* = 5.2, 2.6 Hz, 1H, H6), 3.50 – 3.24 (m, 1H), 2.89 – 2.69 (m, 1H), 2.62 – 2.39 (m, 2H), 2.33 – 2.17 (m, 2H), 1.95 – 1.79 (m, 4H), 1.74 (s, 3H, CH_3), 1.67 (tt, *J* = 10.6, 5.2 Hz, 1H), 1.53 (td, *J* = 9.8, 8.3, 5.5 Hz, 2H), 1.41 (s, 3H, CH_3), 1.39 –

1.32 (m, 1H), 1.29 – 1.22 (m, 2H), 0.76 (s, 3H, CH_3), 0.74 (s, 3H, CH_3); ^{13}C NMR (126 MHz, $CDCl_3$) δ 176.77, 162.28, 152.49, 148.53, 143.31, 138.48, 133.06, 131.29, 129.16, 123.15, 121.12, 56.44, 55.19, 48.72, 43.83, 42.91, 42.59, 38.93, 38.09, 34.64, 31.96, 31.82, 31.37, 24.68, 23.32, 21.69, 20.80, 13.56; HRMS (ESI) *m/z* calcd for $C_{28}H_{33}B_rN_2O_2$ $[M + H]^+$ 509.1798, found 509.1794.

5d: White powder, yield 30%; m.p. 275-277°C; ^{1}H NMR (500 MHz, DMSO-*d*6) δ 11.93 (s, 1H, COOH), 8.20 (d, *J* = 2.2 Hz, 1H, Br-C_6H_3), 7.96 (d, *J* = 8.9 Hz, 1H, Br-C_6H_3), 7.87 (dd, *J* = 8.9, 2.2 Hz, 1H, Br-C_6H_3), 5.86 (dd, *J* = 5.2, 2.5 Hz, 1H, H6), 3.16 (dd, *J* = 15.5, 5.9 Hz, 1H), 2.87 (dd, *J* = 15.6, 2.5 Hz, 1H), 2.34 – 2.26 (m, 1H), 2.20 – 2.11 (m, 1H), 2.05 (dd, *J* = 9.2, 6.7 Hz, 1H), 1.98 – 1.91 (m, 1H), 1.78 – 1.71 (m, 4H), 1.68 (s, 3H, CH_3), 1.63 – 1.57 (m, 1H), 1.52 (dq, *J* = 10.7, 5.1 Hz, 2H), 1.34 (s, 3H, CH_3), 1.23 – 1.19 (m, 1H), 1.09 (dd, *J* = 11.5, 3.0 Hz, 2H), 0.65 (s, 3H, CH_3), 0.63 (s, 3H, CH_3). ^{13}C NMR (126 MHz, DMSO) δ 175.16, 161.34, 154.04, 148.80, 141.53, 140.71, 132.64, 130.97, 130.59, 122.44, 120.76, 55.99, 54.95, 48.45, 44.74, 44.01, 43.51, 42.48, 38.14, 37.88, 37.22, 34.63, 31.55, 31.47, 24.50, 23.76, 20.67, 13.55; HRMS (ESI) *m/z* calcd for $C_{28}H_{33}BrN_2O_2$ $[M + H]^+$ 509.1798, found 509.1787.

5e. White solid, yield 40%; m.p. ＞300°C; ^{1}H NMR (500 MHz, DMSO-*d*6) δ 11.94 (s, 1H, COOH), 10.24 (s, 1H, OH), 7.81 – 7.79 (m, 1H, HO-C_6H_3), 7.28 (dd, *J* = 9.0, 2.7 Hz, 1H, HO-C_6H_3), 7.19 (d, *J* = 2.6 Hz, 1H, HO-C_6H_3), 5.90 – 5.79 (m, 1H, H6), 3.14 – 3.05 (m, 1H), 2.77 (d, *J* = 15.3 Hz, 1H), 2.36 – 2.28 (m, 1H), 2.17 (dt, *J* = 17.6, 5.1 Hz, 1H), 2.06 (d, *J* = 11.9 Hz, 1H), 2.03 – 1.95 (m, 2H), 1.73 (dd, *J* = 14.8, 7.7 Hz, 4H), 1.67 (s, 3H, CH_3), 1.53 (dd, *J* = 10.6, 5.7 Hz, 1H), 1.47 – 1.41 (m, 1H), 1.32 (s, 3H, CH_3), 1.27 – 1.19 (m, 4H), 0.65 (d, *J* = 2.8 Hz, 6H, 2 × CH_3); ^{13}C NMR (125 MHz, DMSO) δ 174.67, 159.69, 157.86, 148.93, 148.48, 142.94, 135.39, 129.10, 121.40, 119.93, 109.30, 65.64, 58.91, 55.53, 54.46, 48.05, 43.02, 41.71, 37.47, 34.19, 31.31, 31.14, 31.10, 24.01, 23.26, 20.51, 20.19, 13.06; HRMS (ESI) *m/z* calcd for C28H34N2O3 $[M + H]^+$ 447.2642, found 447.2633.

5f: White solid, yield 39%; m.p. ＞280°C; ^{1}H NMR (500 MHz, DMSO-*d*6) δ 11.95 (s, 1H, COOH), 10.27 (s, 1H, HO-C_6H_3), 7.84 (d, *J* = 9.0 Hz, 1H, HO-C_6H_3), 7.28 (dd, *J* = 9.0, 2.7 Hz, 1H, HO-C_6H_3), 7.18 (d, *J* = 2.7 Hz, 1H), 5.84 (dd, *J* = 5.2, 2.5 Hz, 1H, H6), 3.11 (dd, *J* = 15.5, 5.2 Hz, 1H), 2.79 (d, *J* = 15.5 Hz, 1H), 2.31 (t, *J* = 9.2 Hz, 1H), 2.17 (dt, *J* = 17.8, 5.3 Hz, 1H), 2.10 – 2.03 (m, 1H), 1.98 (dd, *J* = 20.2, 9.4 Hz, 1H), 1.80 – 1.69 (m, 4H), 1.65 (s, 3H, CH_3), 1.53 (qd, *J* = 10.6, 5.6 Hz, 1H), 1.43 (dd, *J* = 12.5, 3.9 Hz, 1H), 1.32 (s, 3H), 1.29 – 1.16 (m, 4H), 0.66 (d, *J* = 2.2 Hz, 6H, 2 × CH_3); ^{13}C NMR (126 MHz, DMSO) δ 174.73, 158.02, 156.33, 151.88, 148.97, 141.97, 136.45, 129.63, 121.24, 119.91, 108.85, 62.80, 55.55, 54.48, 48.08, 43.59, 43.05, 41.43, 37.46, 34.23, 31.34, 31.27, 31.11, 24.04, 23.28, 20.53, 20.21, 13.10; HRMS (ESI) *m/z* calcd for $C_{28}H_{34}N_2O_3$ $[M + H]^+$447.2642, found 447.2640.

2.4 *Compound **6***

Compound **6:** White solid, yield: 96%; m.p. 190-192°C; ^{1}H NMR (500 MHz, Chloroform-*d*) δ 5.70 – 5.66 (m, 1H, H4), 3.93 (dt, *J* = 25.1, 7.0 Hz, 2H, -OCH_2CH_2O-), 3.84 (h, *J* = 7.2 Hz, 2H), 2.40 – 2.20 (m, 4H), 2.06 – 1.95 (m, 2H), 1.84 – 1.74 (m, 2H), 1.72 – 1.60 (m, 4H), 1.55 – 1.46 (m, 2H), 1.44 – 1.37 (m, 1H), 1.25 (s, 3H, CH_3), 1.19 – 1.16 (m, 1H), 1.15 (s, 3H, CH_3), 1.14 – 1.09 (m, 1H), 1.04 – 0.96 (m, 2H), 0.89 (td, *J* = 11.6, 4.3 Hz, 1H), 0.77 (s, 3H, CH_3); ^{13}C NMR (126 MHz, $CDCl_3$) δ 199.65, 171.61, 123.81, 111.81, 65.23, 63.24, 58.15, 55.76, 53.80, 41.86, 39.31, 38.64, 35.72, 35.12, 34.02, 32.96, 31.96, 24.60, 23.77, 22.95, 20.85, 17.42, 12.98; HRMS (ESI) *m/z*: calcd for $C_{23}H_{34}O_3$ $[M+H]^+$ 359.2581, found $[M+H]^+$ 359.2581.

2.5 *Compound **7***

Compound **7:** White solid, yield: 60%; m.p. 187-188°C; ^{1}H NMR (500 MHz, Chloroform-*d*) δ 5.53 (dd, *J* = 5.4, 2.5 Hz, 1H, H6), 4.02 – 3.91 (m, 2H, -OCH_2CH_2O-), 3.86 (m, *J* = 7.3, 6.7 Hz, 2H, -OCH_2CH_2O-), 2.57 – 2.40 (m, 2H, H2), 2.12 – 2.05 (m, 2H, H7), 2.03 – 1.97 (m, 1H), 1.79 (dd, *J* = 10.8, 8.2 Hz, 1H), 1.75 – 1.69 (m, 2H), 1.67 – 1.57 (m, 3H), 1.57 – 1.46 (m, 2H), 1.44 – 1.37 (m, 1H), 1.28 (s, 3H, CH_3), 1.21 (s, 6H, 2 × CH_3), 1.19 – 1.08 (m, 2H), 1.08 – 0.98 (m, 2H), 0.83 (s, 3H, CH_3), 0.76 (s, 3H, CH_3); ^{13}C NMR (125 MHz, $CDCl_3$) δ 216.89, 149.85, 119.89, 111.91,

65.23, 63.23, 58.20, 56.66, 48.91, 48.68, 41.85, 39.42, 37.11, 33.74, 32.10, 31.65, 30.75, 30.27, 27.26, 24.61, 23.76, 23.02, 21.10, 19.37, 12.94; HRMS (ESI) *m/z*: calcd for $C_{23}H_{34}O_3$ $[M+H]^+$ 359.2581, found 359.2585.

2.6 *Compound **9b-9o** and **10b-10o***

9b: White solid, yield: 70%; m.p. 150-152°C; ^{1}H NMR (500 MHz, Chloroform-*d*) δ 8.01 (ddq, *J* = 24.5, 6.7, 3.3 Hz, 2H, C_6H_4), 7.65 (dq, *J* = 6.6, 3.4 Hz, 2H, C_6H_4), 5.83 (dd, *J* = 5.2, 2.5 Hz, 1H, H6), 3.30 (d, *J* = 15.3 Hz, 1H), 2.84 (d, *J* = 15.3 Hz, 1H), 2.56 (t, *J* = 9.1 Hz, 1H), 2.22 (ddt, *J* = 16.4, 13.5, 8.4 Hz, 2H), 2.13 (s, 3H, CH_3), 1.93 – 1.78 (m, 2H), 1.78 (s, 3H, CH_3), 1.68 (ddd, *J* = 13.2, 6.4, 3.6 Hz, 2H), 1.64 – 1.58 (m, 1H), 1.54 (d, *J* = 8.7 Hz, 2H), 1.39 (s, 3H, CH_3), 1.37 – 1.20 (m, 4H), 0.75 (s, 3H, CH_3), 0.65 (s, 3H, CH_3); ^{13}C NMR (125 MHz, $CDCl_3$) δ 209.36, 160.73, 152.42, 149.28, 142.42, 140.90, 129.09, 128.93, 128.66, 128.32, 120.52, 63.77, 57.16, 48.81, 44.63, 44.11, 42.35, 38.98, 38.11, 34.60, 31.91, 31.64, 31.58, 31.47, 24.54, 23.01, 21.35, 20.53, 13.41; HRMS (ESI) *m/z* calcd for $C_{29}H_{36}N_2O$ $[M + H]^+$ 429.2900, found 429.2900.

9c: White solid, yield: 27%; m.p. 98-100°C; ^{1}H NMR (500 MHz, Chloroform-*d*) δ 8.02 (dd, *J* = 9.2, 5.8 Hz, 1H, F-C_6H_3), 7.61 (dd, *J* = 9.3, 2.8 Hz, 1H, F-C_6H_3), 7.44 (td, *J* = 8.7, 2.8 Hz, 1H, F-C_6H_3), 5.84 (dd, *J* = 5.2, 2.4 Hz, 1H, H6), 3.29 (d, *J* = 15.4 Hz, 1H), 2.83 (d, *J* = 15.4 Hz, 1H), 2.58 (t, *J* = 9.0 Hz, 1H), 2.25 (dt, *J* = 18.2, 5.7 Hz, 2H), 2.15 (s, 3H, CH_3), 2.13 (d, *J* = 4.6 Hz, 1H), 1.83 (ddd, *J* = 20.8, 9.4, 3.1 Hz, 2H), 1.75 (s, 3H, CH_3), 1.69 (d, *J* = 4.8 Hz, 1H), 1.63 (dd, *J* = 10.8, 5.9 Hz, 1H), 1.54 (t, *J* = 10.7 Hz, 2H), 1.39 (s, 3H, CH_3), 1.25 (s, 4H), 0.75 (s, 3H, CH_3), 0.66 (s, 3H, CH_3). ^{13}C NMR (126 MHz, $CDCl_3$) δ 209.46, 163.34, 161.36, 160.12, 153.39, 149.05, 149.05, 141.58, 141.47, 139.59, 131.07, 130.99, 120.69, 118.97, 118.77, 112.04, 111.87, 63.78, 57.16, 48.79, 44.58, 44.14, 42.29, 38.98, 38.10, 34.58, 31.91, 31.70, 31.58, 31.49, 29.85, 24.56, 23.02, 21.37, 20.56, 13.44; HRMS (ESI) *m/z* calcd for $C_{29}H_{38}N_2O$ $[M + H]^+$ 447.2806, found 447.2784.

9d: White solid, yield: 25%; m.p. 135-137°C; ^{1}H NMR (500 MHz, Chloroform-*d*) δ 7.96 (dd, *J* = 9.2, 5.7 Hz, 1H, F-C_6H_3), 7.65 (dd, *J* = 9.4, 2.8 Hz, 1H, F-C_6H_3), 7.42 (td, *J* = 9.2, 8.8, 2.7 Hz, 1H, F-C_6H_3), 5.82 (dd, *J* = 5.2, 2.5 Hz, 1H, H6), 3.26 (d, *J* = 15.3 Hz, 1H), 2.81 (d, *J* = 15.3 Hz, 1H), 2.56 (t, *J* = 9.0 Hz, 1H), 2.29 – 2.17 (m, 2H), 2.13 (s, 3H, CH_3), 2.12 (d, *J* = 3.5 Hz, 1H), 1.89 – 1.76 (m, 2H), 1.73 (s, 3H, CH_3), 1.69 – 1.64 (m, 1H), 1.64 – 1.58 (m, 1H), 1.53 (d, *J* = 9.1 Hz, 2H), 1.37 (s, 3H, CH_3), 1.23 (s, 4H), 0.73 (s, 3H, CH_3), 0.64 (s, 3H, CH_3); ^{13}C NMR (126 MHz, $CDCl_3$) δ 209.37, 163.15, 161.69, 161.16, 151.77, 151.74, 148.97, 143.08, 142.98, 137.98, 130.22, 130.14, 120.67, 119.15, 118.94, 112.71, 112.54, 63.73, 57.11, 48.73, 44.42, 44.09, 42.41, 38.93, 38.06, 34.55, 31.87, 31.65, 31.54, 31.43, 24.52, 22.98, 21.33, 20.52, 13.40; HRMS (ESI) *m/z* calcd for $C_{29}H_{38}N_2O$ $[M + H]^+$ 447.2806, found 447.2784.

9e: White solid, yield: 28%; 145-147°C; ^{1}H NMR (500 MHz, Chloroform-*d*) δ 7.97 (d, *J* = 2.3 Hz, 2H, Cl-C_6H_3), 7.95 (s, 1H, Cl-C_6H_3), 5.83 (dd, *J* = 5.2, 2.4 Hz, 1H, H6), 3.28 (d, *J* = 15.4 Hz, 1H), 2.82 (d, *J* = 15.4 Hz, 1H), 2.56 (t, *J* = 9.0 Hz, 1H), 2.22 (ddt, *J* = 14.4, 11.5, 7.4 Hz, 2H), 2.14 (s, 3H, CH_3), 2.12 (d, *J* = 2.7 Hz, 1H), 1.85 – 1.76 (m, 2H), 1.74 (s, 3H, CH_3), 1.71 – 1.59 (m, 2H), 1.53 (t, *J* = 10.4 Hz, 2H), 1.38 (s, 3H, CH_3), 1.31 – 1.17 (m, 4H), 0.74 (s, 3H, CH_3), 0.65 (s, 3H, CH_3); ^{13}C NMR (126 MHz, $CDCl_3$) δ 209.45, 161.07, 153.52, 148.92, 141.22, 140.90, 134.54, 130.31, 129.65, 127.36, 120.72, 63.75, 57.11, 48.73, 44.56, 44.11, 42.40, 38.93, 38.07, 34.56, 31.87, 31.68, 31.54, 31.41, 24.53, 22.99, 21.35, 20.53, 13.42; HRMS (ESI) *m/z* calcd for $C_{29}H_{37}FN_2O$ $[M+H]^+$ 463.2511, found 463.2495.

9f: White solid, yield: 26%; m.p. 150-152°C; ^{1}H NMR (500 MHz, Chloroform-*d*) δ 8.02 (d, *J* = 2.3 Hz, 1H, Cl-C_6H_3), 7.89 (d, *J* = 8.9 Hz, 1H, Cl-C_6H_3), 7.58 (dd, *J* = 8.9, 2.3 Hz, 1H, Cl-C_6H_3), 5.81 (dd, *J* = 5.3, 2.5 Hz, 1H, H6), 3.26 (d, *J* = 15.4 Hz, 1H), 2.80 (d, *J* = 15.4 Hz, 1H), 2.55 (t, *J* = 9.0 Hz, 1H), 2.26 – 2.16 (m, 2H), 2.12 (s, 3H, CH_3), 2.10 (s, 1H), 1.84 – 1.78 (m, 2H), 1.71 (s, 3H, CH_3), 1.63 (ddq, *J* = 25.6, 10.6, 4.9 Hz, 2H), 1.52 (d, *J* = 9.3 Hz, 2H), 1.36 (s, 3H, CH_3), 1.28 – 1.20 (m, 4H), 0.72 (s, 3H, CH_3), 0.63 (s, 3H, CH_3); ^{13}C NMR (125 MHz, CDCl3) δ 209.36, 161.80, 152.73, 148.83, 142.64, 139.36, 134.31, 129.83, 129.53, 128.03, 120.70, 63.70, 57.07, 48.69, 44.49, 44.06, 42.43, 38.89, 38.03, 34.54, 31.83, 31.64, 31.50, 31.39, 24.50, 22.96, 21.30, 20.50, 13.38; HRMS (ESI) *m/z* calcd for

$C_{29}H_{37}ClN_2O$ [M+H]$^+$ 463.2511, found 463. 2476.

9g: White solid, yield: 30%; m.p. 89-91°C ^{1}H NMR (500 MHz, Chloroform-*d*) δ 8.11 (d, *J* = 2.2 Hz, 1H, Br-C_6H_3), 7.84 (d, *J* = 8.9 Hz, 1H, Br-C_6H_3), 7.67 (dd, *J* = 8.8, 2.2 Hz, 1H, Br-C_6H_3), 5.78 (dd, *J* = 5.4, 2.4 Hz, 1H, H6), 3.00 (dd, *J* = 227.6, 15.4 Hz, 2H), 2.51 (t, *J* = 9.0 Hz, 1H), 2.23 – 2.13 (m, 2H), 2.09 (s, 3H, CH_3), 2.07 (s,1H), 1.82 – 1.75 (m, 2H), 1.69 (s, 3H, CH_3), 1.66 – 1.61 (m, 1H), 1.56 (dq, *J* = 10.4, 4.5 Hz, 1H), 1.49 (d, *J* = 8.9 Hz, 2H), 1.33 (s, 3H, CH_3), 1.26 – 1.14 (m, 4H), 0.68 (s, 3H, CH_3), 0.60 (s, 3H, CH_3); ^{13}C NMR (125 MHz, CDCl3) δ 209.16, 161.08, 153.35, 148.73, 141.43, 141.00, 132.04, 130.63, 130.33, 122.49, 120.59, 63.59, 56.96, 48.58, 44.43, 43.96, 42.31, 38.79, 37.93, 34.43, 31.74, 31.57, 31.40, 31.32, 24.42, 22.87, 21.23, 20.42, 13.31; HRMS (ESI) *m*/*z* calcd for $C_{29}H_{35}BrN_2O$ [M+H]$^+$ 507.2005, found 507.1965.

9h: White solid, yield: 30%; m.p. 112-114°C; ^{1}H NMR (500 MHz, Chloroform-*d*) δ 8.22 (d, *J* = 2.1 Hz, 1H, Br-C_6H_3), 7.84 (d, *J* = 8.8 Hz, 1H, Br-C_6H_3), 7.72 (dd, *J* = 8.9, 2.2 Hz, 1H, Br-C_6H_3), 5.82 (dd, *J* = 5.2, 2.4 Hz, 1H, H6), 3.27 (d, *J* = 15.4 Hz, 1H), 2.79 (d, *J* = 15.4 Hz, 1H), 2.56 (t, *J* = 9.0 Hz, 1H), 2.31 – 2.21 (m, 2H), 2.14 (s, 3H, CH_3), 2.11 (dd, *J* = 6.6, 2.8 Hz, 1H), 1.90 – 1.77 (m, 2H), 1.72 (s, 3H, CH_3), 1.66 (dt, *J* = 9.2, 5.1 Hz, 1H), 1.63 – 1.58 (m, 1H), 1.54 (d, *J* = 9.6 Hz, 2H), 1.37 (s, 3H, CH_3), 1.24 (s, 4H), 0.73 (s, 3H, CH_3), 0.64 (s, 3H, CH_3); ^{13}C NMR (125 MHz, $CDCl_3$) δ 209.41, 161.80, 152.92, 148.82, 142.99, 139.62, 132.42, 131.42, 129.67, 122.45, 120.74, 63.73, 57.10, 48.71, 44.54, 44.10, 42.46, 38.92, 38.04, 34.58, 31.86, 31.67, 31.53, 31.41, 24.52, 22.98, 21.33, 20.52, 13.41; HRMS (ESI) *m*/*z* calcd for $C_{29}H_{35}BrN_2O$ [M+H]$^+$ 507.2005, found 507.1948.

9i: Because both of the ^{1}H NMR and ^{13}C NMR of target compound **10i** have been obtained, so the NMR spectra of **9i,** as the precursors of **10i,** haven't been necessary any more.

9j: As the same as that of above, so the NMR spectra of **9j,** as the precursors of **10j** haven't been necessary any more.

9k: White solid, yield: 33%; m.p. 160-162°C; ^{1}H NMR (500 MHz, Chloroform-*d*) δ 7.88 (d, *J* = 9.1 Hz, 1H, MeO-C_6H_3), 7.35 (d, *J* = 2.7 Hz, 1H, MeO-C_6H_3), 7.32 (dd, *J* = 9.1, 2.7 Hz, 1H, MeO-C_6H_3), 5.83 (dd, *J* = 5.3, 2.5 Hz, 1H, H6), 3.96 (s, 3H, OMe), 3.26 (d, *J* = 15.3 Hz, 1H), 2.81 (d, *J* = 15.2 Hz, 1H), 2.58 (t, *J* = 9.0 Hz, 1H), 2.30 – 2.20 (m, 2H), 2.15 (s, 3H, CH_3), 2.13 (d, *J* = 2.4 Hz, 1H), 1.88 – 1.80 (m, 2H), 1.75 (s, 3H, CH_3), 1.72 – 1.60 (m, 3H), 1.55 (d, *J* = 9.1 Hz, 2H), 1.39 (s, 3H, CH_3), 1.29 – 1.22 (m, 3H), 0.76 (s, 3H, CH_3), 0.65 (s, 3H, CH_3); ^{13}C NMR (125 MHz, CDCl3) δ 209.55, 160.68, 160.02, 149.56, 149.36, 143.04, 143.83, 129.16, 122.10, 120.52, 106.76, 63.80, 57.18, 55.89, 48.79, 44.16, 42.24, 39.00, 38.12, 34.56, 31.94, 31.71, 31.59, 31.54, 24.56, 22.98, 21.35, 20.56, 13.44; HRMS (ESI) *m*/*z* calcd for $C_{30}H_{38}N_2O_2$ [M+H]$^+$ 459.3006, found 459.3003.

9l: White solid, yield: 28%; m.p. 120-122°C; ^{1}H NMR (500 MHz, Chloroform-*d*) δ 7.92 – 7.86 (m, 1H, MeO-C_6H_3), 7.31 (d, *J* = 2.8 Hz, 1H, MeO-C_6H_3), 7.30 (d, *J* = 1.9 Hz, 1H, MeO-C_6H_3), 5.82 (dd, *J* = 5.1, 2.5 Hz, 1H, H6), 3.93 (s, 3H, OMe), 3.25 (d, *J* = 15.4 Hz, 1H), 2.81 (d, *J* = 15.3 Hz, 1H), 2.55 (q, *J* = 11.4, 10.3 Hz, 1H), 2.21 (ddt, *J* = 14.2, 11.2, 7.4 Hz, 2H), 2.14 (s, 3H, CH_3), 2.13 – 2.05 (m, 2H), 1.87 – 1.76 (m, 2H), 1.73 (s, 3H, CH_3), 1.69 – 1.59 (m, 3H), 1.54 (d, *J* = 8.6 Hz, 2H), 1.38 (s, 3H, CH_3), 1.24 (m, 3H), 0.76 (s, 3H, CH_3), 0.65 (s, 3H, CH_3); ^{13}C NMR (125 MHz, $CDCl_3$) δ 209.48, 160.17, 157.94, 152.15, 149.42, 142.19, 138.41, 130.01, 121.66, 120.43, 106.05, 63.78, 57.17, 55.79, 48.82, 44.59, 44.13, 41.99, 38.99, 38.09, 34.50, 31.92, 31.66, 31.58, 29.81, 24.54, 22.99, 21.32, 20.54, 13.41; HRMS (ESI) *m*/*z* calcd for $C_{30}H_{38}N_2O_2$ [M+H]$^+$ 459.3006, found 459.2963

9m: White solid, yield: 25%; m.p. 80-82°C;^{1}H NMR (500 MHz, Chloroform-*d*) δ 8.87 (d, *J* = 2.5 Hz, 1H, NO_2-C_6H_3), 8.41 (dd, *J* = 9.1, 2.6 Hz, 1H, NO_2-C_6H_3), 8.14 (d, *J* = 9.1 Hz, 1H, NO_2-C_6H_3), 5.85 (dd, *J* = 5.3, 2.5 Hz, 1H, H6), 3.34 (d, *J* = 15.5 Hz, 1H), 2.85 (d, *J* = 15.6 Hz, 1H), 2.57 (t, *J* = 9.0 Hz, 1H), 2.28 – 2.18 (m, 2H), 2.13 (s, 3H, CH_3), 2.10 (d, *J* = 3.5 Hz, 1H), 1.87 – 1.77 (m, 2H), 1.75 (s, 3H, CH_3), 1.70 – 1.65 (m, 1H), 1.62 (dt, *J* = 10.4, 4.8 Hz,

1H), 1.54 (d, *J* = 9.6 Hz, 2H), 1.40 (s, 3H, CH_3), 1.37 – 1.30 (m, 2H), 1.28 – 1.23 (m, 2H), 0.73 (s, 3H, CH_3), 0.64 (s, 3H, CH_3); ^{13}C NMR (125 MHz, $CDCl_3$) δ 209.34, 164.56, 155.34, 148.30, 147.25, 144.96, 139.71, 130.54, 124.89, 122.19, 121.10, 63.67, 57.03, 48.64, 44.51, 44.06, 42.89, 38.85, 38.01, 34.59, 31.79, 31.65, 31.49, 31.28, 24.50, 22.98, 21.34, 20.54, 13.40; HRMS (ESI) *m/z* calcd for $C_{29}H_{35}N_3O_3$ [M+H]$^+$ 474.2751, found 474.2743.

9n: White solid, yield: 24%; m.p. 85-87°C; ^{1}H NMR (500 MHz, Chloroform-*d*) δ 8.96 (d, *J* = 2.5 Hz, 1H, NO_2-C_6H_3), 8.45 (dd, *J* = 9.1, 2.5 Hz, 1H, NO_2-C_6H_3), 8.11 (d, *J* = 9.1 Hz, 1H, NO_2-C_6H_3), 5.87 (dd, *J* = 5.2, 2.5 Hz, 1H, H6), 3.36 (d, *J* = 15.6 Hz, 1H), 2.88 (d, *J* = 15.6 Hz, 1H), 2.58 (t, *J* = 9.1 Hz, 1H), 2.33 – 2.22 (m, 2H), 2.15 (s, 3H, CH_3), 2.13 (d, *J* = 2.3 Hz, 1H), 1.83 (ddd, *J* = 12.9, 9.1, 3.1 Hz, 2H), 1.78 (s, 3H, CH_3), 1.73 – 1.69 (m, 1H), 1.64 (dt, *J* = 10.4, 5.2 Hz, 1H), 1.56 (d, *J* = 9.2 Hz, 2H), 1.42 (s, 3H, CH_3), 1.37 (d, *J* = 15.3 Hz, 2H), 1.31 – 1.23 (m, 2H), 0.76 (s, 3H, CH_3), 0.67 (s, 3H, CH_3); ^{13}C NMR (125 MHz, $CDCl_3$) δ 209.41, 163.69, 156.29, 148.37, 147.30, 143.64, 141.24, 129.94, 125.59, 122.51, 121.18, 63.76, 57.13, 48.78, 44.82, 44.12, 42.79, 38.94, 38.14, 34.68, 31.87, 31.68, 31.58, 31.40, 24.56, 23.09, 21.41, 20.63, 13.46; HRMS (ESI) *m/z* calcd for $C_{29}H_{35}N_3O_3$ [M+H]$^+$ 474.2751, found 474.2743.

9o: White solid, yield: 62%; m.p. 150-152°C; ^{1}H NMR (500 MHz, Chloroform-*d*) δ 8.14 (s, 1H, 2Cl-C_6H_2), 8.07 (s, 1H, 2Cl-C_6H_2), 5.81 (dd, *J* = 5.2, 2.5 Hz, 1H, H6), 3.25 (d, *J* = 15.5 Hz, 1H), 2.79 (d, *J* = 15.4 Hz, 1H), 2.55 (t, *J* = 9.0 Hz, 1H), 2.25 – 2.16 (m, 2H), 2.12 (s, 3H, CH_3), 2.11 (d, *J* = 2.6 Hz, 1H), 1.78 (ddp, *J* = 13.9, 11.4, 3.8, 3.3 Hz, 2H), 1.70 (s, 3H, CH_3), 1.66 (dd, *J* = 9.2, 5.1 Hz, 1H), 1.59 (td, *J* = 11.8, 11.2, 6.2 Hz, 1H), 1.51 (t, *J* = 10.8 Hz, 2H), 1.36 (s, 3H, CH_3), 1.31 (dd, *J* = 9.6, 3.2 Hz, 1H), 1.25 – 1.18 (m, 3H), 0.70 (s, 3H, CH_3), 0.63 (s, 3H, CH_3); ^{13}C NMR (125 MHz, $CDCl_3$) δ 209.33, 162.16, 153.87, 148.60, 141.11, 139.68, 133.18, 133.03, 129.75, 129.07, 120.85, 63.69, 57.06, 48.67, 44.48, 44.06, 42.51, 38.87, 38.02, 34.53, 31.81, 31.64, 31.50, 31.33, 24.50, 22.98, 21.32, 20.50, 13.39; HRMS (ESI) *m/z* calcd for $C_{29}H_{37}N_3O_3$ [M+H]$^+$ 497.2121, found 497.2096.

10b: White powder, yield 87%; m.p. 95–97°C; ^{1}H NMR (500 MHz, Chloroform-*d*) δ 8.10 – 7.91 (m, 2H, C_6H_4), 7.66 (dd, *J* = 6.4, 3.4 Hz, 2H, C_6H_4), 5.83 (dt, *J* = 5.3, 2.6 Hz, 1H, H6), 3.83 – 3.70 (m, 1H, H20), 3.31 (d, *J* = 15.4 Hz, 1H), 2.83 (d, *J* = 15.4 Hz, 1H), 2.40 – 2.06 (m, 2H), 1.99 – 1.82 (m, 1H), 1.76 (s, 3H, CH_3), 1.75 – 1.66 (m, 3H), 1.63 (ddt, *J* = 15.5, 10.4, 5.0 Hz, 2H), 1.52 (td, *J* = 13.0, 4.0 Hz, 1H), 1.39 (s, 3H, CH_3), 1.32 (ddd, *J* = 18.9, 12.1, 5.3 Hz, 2H), 1.27 – 1.19 (m, 3H), 1.16 (d, *J* = 6.1 Hz, 3H, CH_3), 0.79 (s, 3H, CH_3), 0.76 (s, 3H, CH_3); ^{13}C NMR (125 MHz, $CDCl_3$) δ 160.84, 152.61, 149.16, 142.30, 140.71, 128.97, 128.85, 128.57, 128.19, 120.58, 70.47, 58.40, 56.36, 48.82, 44.43, 42.36, 42.25, 39.90, 38.03, 34.54, 31.97, 31.35, 31.35, 25.76, 24.53, 23.84, 21.12, 20.49, 12.46; HRMS (ESI) *m/z* calcd for $C_{29}H_{38}N_2O$ [M + H]$^+$ 431.3057, found 431.3063.

10c: White solid, yield: 70%; m.p. 130-132°C; ^{1}H NMR (500 MHz, Chloroform-*d*) δ 8.02 (dd, *J* = 9.2, 5.8 Hz, 1H, F-C_6H_3), 7.61 (dd, *J* = 9.3, 2.8 Hz, 1H, F-C_6H_3), 7.44 (td, *J* = 8.7, 2.7 Hz, 1H, F-C_6H_3), 5.83 (dd, *J* = 5.3, 2.5 Hz, 1H, H6), 3.76 (dq, *J* = 10.0, 6.1 Hz, 1H, H20), 3.29 (d, *J* = 15.5 Hz, 1H), 2.81 (d, *J* = 15.4 Hz, 1H), 2.31 – 2.12 (m, 2H), 1.80 – 1.76 (m, 1H), 1.74 (s, 3H, CH_3), 1.73 – 1.68 (m, 2H), 1.61 (dtd, *J* = 19.0, 9.5, 8.6, 4.5 Hz, 2H), 1.55 – 1.50 (m, 1H), 1.38 (s, 3H, CH_3), 1.37 – 1.31 (m, 2H), 1.27 – 1.23 (m, 4H), 1.16 (d, *J* = 6.1 Hz, 3H, CH_3), 0.79 (s, 3H, CH_3), 0.76 (s, 3H, CH_3); HRMS (ESI) *m/z* calcd for $C_{29}H_{37}FN_2O$ [M+H]$^+$ 449.2963, found 449.2948.

10d: White solid, yield: 73%; m.p. 112-114°C; ^{1}H NMR (500 MHz, Chloroform-*d*) δ 8.01 (dd, *J* = 9.1, 5.8 Hz, 1H, F-C_6H_3), 7.70 (dd, *J* = 9.5, 2.8 Hz, 1H, F-C_6H_3), 7.47 (td, *J* = 8.7, 2.8 Hz, 1H, F-C_6H_3), 5.87 (dd, *J* = 5.3, 2.5 Hz, 1H, H6), 3.79 (tt, *J* = 9.2, 4.5 Hz, 1H, H20), 3.32 (d, *J* = 15.4 Hz, 1H), 2.84 (d, *J* = 15.4 Hz, 1H), 2.25 (ddd, *J* = 15.0, 8.1, 3.9 Hz, 2H), 2.08 (s, 1H), 1.81 (td, *J* = 9.0, 7.5, 2.8 Hz, 2H), 1.78 (s, 3H, CH_3), 1.76 – 1.72 (m, 2H), 1.66 (tt, *J* = 10.5, 5.1 Hz, 2H), 1.57 (dd, *J* = 13.0, 4.0 Hz, 1H), 1.42 (s, 3H, CH_3), 1.30 (d, *J* = 7.2 Hz, 4H), 1.20 (d, *J* = 6.2 Hz, 3H, CH_3), 0.83 (s, 3H, CH_3), 0.79 (s, 3H, CH_3); HRMS (ESI) *m/z* calcd for $C_{29}H_{37}FN_2O$ [M+H]$^+$ 449.2963, found 449.2956.

10e: White solid, yield: 82%, m.p. 172-174°C; ^{1}H NMR (500 MHz, Chloroform-*d*) δ 8.00 – 7.90 (m, 2H, Cl-C_6H_3), 7.60 (dd, *J* = 8.9, 2.3 Hz, 1H, Cl-C_6H_3), 5.83 (dd, *J* = 5.3, 2.5 Hz, 1H, H6), 3.75 (dp, *J* = 8.1, 6.0 Hz, 1H, H20),

3.29 (d, *J* = 15.5 Hz, 1H), 2.81 (d, *J* = 15.4 Hz, 1H), 2.27 – 2.08 (m, 1H), 1.82 – 1.75 (m, 1H), 1.74 (s, 3H, CH_3), 1.70 – 1.65 (m, 2H), 1.61 (tq, *J* = 9.1, 4.3, 3.6 Hz, 2H), 1.51 (ddd, *J* = 13.2, 11.0, 4.0 Hz, 1H), 1.37 (s,3H, CH_3), 1.36 – 1.29 (m, 2H), 1.27 – 1.22 (m, 4H), 1.16 (d, *J* = 6.2 Hz, 3H, CH_3),, 0.79 (s, 3H, CH_3), 0.74 (s, 3H, CH_3); HRMS (ESI) *m*/*z* calcd for $C_{29}H_{37}ClN_2O$ $[M+H]^+$ 465.2667, found 465.2662.

10f: White solid, yield: 82%; m.p. 124-126°C; ^1H NMR (500 MHz, Chloroform-*d*) δ 8.04 (d, *J* = 2.3 Hz, 1H Cl-C_6H_3), 7.91 (d, *J* = 8.8 Hz, 1H Cl-C_6H_3), 7.59 (dd, *J* = 8.9, 2.3 Hz, 1H Cl-C_6H_3), 5.82 (dd, *J* = 5.3, 2.5 Hz, 1H, H6), 3.75 (dq, *J* = 12.0, 6.1 Hz, 1H, H20), 3.35 – 3.21 (m, 1H), 2.80 (d, *J* = 15.4 Hz, 1H), 2.29 – 2.14 (m, 2H), 1.80 – 1.75 (m, 1H), 1.73 (s, 3H, CH_3), 1.69 (d, *J* = 7.6 Hz, 1H), 1.61 (dd, *J* = 10.6, 5.6 Hz, 1H), 1.52 (dd, *J* = 13.0, 3.9 Hz, 1H), 1.37 (s, 3H, CH_3), 1.29 (s, 2H), 1.25 (d, *J* = 2.2 Hz, 3H), 1.16 (d, *J* = 6.2 Hz, 3H, CH_3),, 0.79 (s, 3H, CH_3), 0.74 (s, 3H, CH_3); HRMS (ESI) *m*/*z* calcd for $C_{29}H_{37}ClN_2O$ $[M+H]^+$465.2667, found 465.2667.

10g: White solid, yield: 71%; m.p. ＞280°C; ^1H NMR (500 MHz, Chloroform-*d*) δ 8.14 (d, *J* = 2.2 Hz, 1H, Br-C_6H_3), 7.87 (d, *J* = 8.9 Hz, 1H, Br-C_6H_3), 7.70 (dd, *J* = 8.9, 2.1 Hz, 1H, Br-C_6H_3), 5.81 (dd, *J* = 5.3, 2.5 Hz, 1H, H6), 3.74 (dq, *J* = 11.8, 6.0 Hz, 1H, H20), 3.27 (d, *J* = 15.5 Hz, 1H), 2.79 (d, *J* = 15.5 Hz, 1H), 2.19 (dt, *J* = 14.7, 5.1 Hz, 2H), 2.02 (s, 1H), 1.76 (dd, *J* = 10.9, 2.4 Hz, 2H), 1.72 (s, 3H, CH_3), 1.70 – 1.65 (m, 2H), 1.59 (qd, *J* = 12.6, 11.5, 6.5 Hz, 1H), 1.50 (td, *J* = 13.0, 3.9 Hz, 1H), 1.36 (s, 3H, CH_3), 1.31 – 1.25 (m, 2H), 1.25 – 1.21 (m, 3H), 1.15 (d, *J* = 6.1 Hz, 3H, CH_3), 0.77 (s, 3H, CH_3), 0.72 (s, 3H, CH_3); HRMS (ESI) *m*/*z* calcd for $C_{29}H_{37}BrN_2O$ $[M+H]^+$ 509.2162, found 509.2153.

10h: White solid; Yield 71%; m.p. 186-187°C; ^1H NMR (500 MHz, Chloroform-*d*) δ 8.14 (d, *J* = 2.2 Hz, 1H, Br-C_6H_3), 7.87 (d, *J* = 8.9 Hz, 1H, Br-C_6H_3), 7.70 (dd, *J* = 8.9, 2.1 Hz, 1H, Br-C_6H_3), 5.81 (dd, *J* = 5.3, 2.5 Hz, 1H, H6), 3.74 (dq, *J* = 11.8, 6.0 Hz, 1H, H20), 3.27 (d, *J* = 15.5 Hz, 1H), 2.79 (d, *J* = 15.5 Hz, 1H), 2.19 (dt, *J* = 14.7, 5.1 Hz, 2H), 2.02 (s, 1H), 1.76 (dd, *J* = 10.9, 2.4 Hz, 2H), 1.72 (s, 3H, CH_3), 1.70 – 1.65 (m, 2H), 1.59 (qd, *J* = 12.6, 11.5, 6.5 Hz, 1H), 1.50 (td, *J* = 13.0, 3.9 Hz, 1H), 1.36 (s, 3H, CH_3), 1.31 – 1.25 (m, 2H), 1.25 – 1.21 (m, 3H, CH_3), 1.15 (d, *J* = 6.1 Hz, 3H), 0.77 (s, 3H, CH_3), 0.72 (s, 3H, CH_3); HRMS (ESI) *m*/*z* calcd for $C_{29}H_{37}BrN_2O$ $[M+H]^+$ 509.2162, found 509.2152

10i: White solid, yield: 28%; m.p. ＞280°C; ^1H NMR (500 MHz, DMSO-*d*$_6$) δ 10.23 (s, 1H, OH), 7.79 (d, *J* = 9.0 Hz, 1H, HO-C_6H_3), 7.27 (dd, *J* = 9.0, 2.7 Hz, 1H, HO-C_6H_3), 7.19 (d, *J* = 2.7 Hz, 1H, HO-C_6H_3), 5.82 (dd, *J* = 5.2, 2.5 Hz, 1H H6), 3.49 (dq, *J* = 12.2, 6.0 Hz, 1H, H20), 3.09 (d, *J* = 15.3 Hz, 1H), 2.75 (d, *J* = 15.3 Hz, 1H), 2.22 (dt, *J* = 1z2.9, 3.4 Hz, 1H), 2.13 (dt, *J* = 17.8, 5.3 Hz, 1H), 1.70 (ddd, *J* = 16.3, 10.7, 5.6 Hz, 1H), 1.65 (s, 3H, CH_3), 1.64 – 1.55 (m, 3H), 1.51 (dp, *J* = 10.4, 5.4 Hz, 1H), 1.42 (qd, *J* = 13.2, 3.9 Hz, 1H), 1.31 (s, 3H, CH_3), 1.26 (d, *J* = 9.0 Hz, 1H), 1.19 (tdd, *J* = 13.0, 9.0, 3.9 Hz, 3H), 1.14 – 1.08 (m, 2H), 1.01 (d, *J* = 6.0 Hz, 3H, CH_3), 0.70 (s, 3H, CH_3), 0.64 (s, 3H, CH_3); ^{13}C NMR (126 MHz, DMSO) δ 159.72, 157.84, 148.92, 148.54, 142.94, 135.37, 129.08, 121.37, 120.02, 109.32, 68.30, 57.62, 55.92, 48.59, 48.31, 43.33, 41.92, 41.68, 37.45, 34.19, 31.50, 31.14, 30.89, 25.35, 24.14, 23.79, 20.50, 20.22, 11.97; HRMS (ESI) *m*/*z* calcd for $C_{29}H_{38}N_2O_2$ $[M+H]^+$ 447.3006, found 447.3011.

10j: White solid, yield: 89%; m.p. ＞280°C; ^1H NMR (500 MHz, DMSO-*d*$_6$) δ 10.26 (s, 1H, OH), 7.83 (d, *J* = 9.0 Hz, 1H, HO-C_6H_3), 7.28 (dd, *J* = 9.0, 2.7 Hz, 1H, HO-C_6H_3), 7.18 (d, *J* = 2.6 Hz, 1H, HO-C_6H_3), 5.88 – 5.69 (m, 1H, H6), 3.50 (tt, *J* = 12.1, 6.0 Hz, 1H, H20) 3.11 (d, *J* = 15.5 Hz, 1H), 2.78 (d, *J* = 15.4 Hz, 1H), 2.26 – 2.18 (m, 1H), 2.15 (dt, *J* = 17.6, 5.1 Hz, 1H), 2.04 – 1.93 (m, 1H), 1.78 – 1.69 (m, 1H), 1.65 (s, 3H, CH_3), 1.60 (d, *J* = 7.2 Hz, 1H), 1.56 – 1.50 (m, 1H), 1.43 (dd, *J* = 13.0, 4.0 Hz, 1H), 1.32 (s, 3H, CH_3), 1.23 (d, *J* = 4.7 Hz, 4H), 1.19 – 1.14 (m, 2H), 1.01 (d, *J* = 6.0 Hz, 3H, CH_3), 0.72 (s, 3H, CH_3), 0.67 (s, 3H, CH_3); ^{13}C NMR (126 MHz, DMSO) δ 157.57, 156.23, 151.46, 148.62, 141.67, 136.59, 129.14, 120.63, 119.79, 108.74, 68.53, 57.50, 55.75, 48.24, 43.72, 41.71, 41.12, 37.33, 33.86, 31.36, 30.94, 30.71, 30.49, 25.16, 23.91, 23.36, 20.43, 19.86, 11.69; HRMS (ESI) *m*/*z* calcd for $C_{29}H_{38}N_2O_2$ $[M+H]^+$ 447.3006, found 447.2993

10k: White solid, yield: 88%; m.p. 198-200°C; ^{1}H NMR (500 MHz, Chloroform-*d*) δ 7.88 (d, *J* = 9.1 Hz, 1H, MeO-C_6H_3), 7.35 (d, *J* = 2.7 Hz, 1H, MeO-C_6H_3), 7.32 (dd, *J* = 9.1, 2.8 Hz, 1H, MeO-C_6H_3), 5.83 (dd, *J* = 5.4, 2.5 Hz, 1H H6), 3.96 (s, 3H, OMe), 3.75 (dt, *J* = 12.2, 6.0 Hz, 1H, H20), 3.26 (d, *J* = 15.4 Hz, 1H), 2.80 (d, *J* = 15.3 Hz, 1H), 2.29 – 2.12 (m, 2H), 1.79 (dd, *J* = 10.6, 2.6 Hz, 1H), 1.75 (s, 3H, CH_3), 1.73 – 1.67 (m, 2H), 1.62 (tt, *J* = 10.4, 5.3 Hz, 2H), 1.52 (td, *J* = 13.0, 4.0 Hz, 2H), 1.39 (s, 3H, CH_3), 1.34 (dd, *J* = 17.3, 4.1 Hz, 2H), 1.26 (dd, *J* = 7.3, 2.1 Hz, 3H), 1.16 (d, *J* = 6.1 Hz, 3H, CH_3), 0.79 (s, 3H, CH_3), 0.77 (s, 3H, CH_3); HRMS (ESI) *m*/*z* calcd for $C_{30}H_{40}N_2O_2$ $[M+H]^+$ 461.3163, found 461.3155

10l: White solid, yield: 84%; m.p. 160-162°C; ^{1}H NMR (500 MHz, Chloroform-*d*) δ 7.92 (d, *J* = 9.7 Hz, 1H, MeO-C_6H_3), 7.32 (d, *J* = 7.3 Hz, 2H, MeO-C_6H_3), 5.83 (dd, *J* = 5.2, 2.5 Hz, 1H, H6), 3.95 (s, 3H, OMe), 3.75 (dt, *J* = 12.1, 6.0 Hz, 1H, H20), 3.28 (d, *J* = 15.4 Hz, 1H), 2.81 (d, *J* = 15.4 Hz, 1H), 2.41 – 2.07 (m, 2H), 1.83 – 1.76 (m, 1H), 1.74 (s, 3 H, CH_3), 1.72 – 1.67 (m, 2H), 1.63 (qd, *J* = 10.6, 5.4 Hz, 2H), 1.53 (td, *J* = 13.0, 4.1 Hz, 2H), 1.38 (s, 3H, CH_3), 1.36 – 1.30 (m, 2H), 1.25 (s, 3H), 1.16 (d, *J* = 6.1 Hz, 3H, CH_3), 0.80 (s, 3H, CH_3), 0.77 (s, 3H, CH_3); HRMS (ESI) *m*/*z* calcd for $C_{29}H_{38}N_2O_2$ $[M+H]^+$ 461.3163, found 461.3169.

10m: White solid, yield: 81%; m.p. 70-71°C;^{1}H NMR (500 MHz, Chloroform-*d*) δ 8.89 (d, *J* = 2.5 Hz, 1H, NO_2-C_6H_3), 8.43 (dd, *J* = 9.2, 2.5 Hz, 1H, NO_2-C_6H_3), 8.15 (d, *J* = 9.1 Hz, 1H, NO_2-C_6H_3), 5.85 (dd, *J* = 5.3, 2.5 Hz, 1H, H6), 3.75 (dt, *J* = 12.1, 6.1 Hz, 1H, H20), 3.36 (d, *J* = 15.6 Hz, 1H), 2.86 (d, *J* = 15.6 Hz, 1H), 2.28 – 2.14 (m, 2H), 1.76 (s, 3H, CH_3), 1.76 – 1.67 (m, 3H), 1.62 (qd, *J* = 12.7, 11.7, 6.4 Hz, 2H), 1.53 (td, *J* = 13.1, 4.0 Hz, 2H), 1.41 (s, 3H, CH_3), 1.37 – 1.32 (m, 2H), 1.28 (dd, *J* = 8.1, 6.1 Hz, 1H), 1.17 (d, *J* = 6.1 Hz, 3H, CH_3), 0.80 (s, 3H, CH_3), 0.75 (s, 3H, CH_3); HRMS (ESI) *m*/*z* calcd for $C_{29}H_{37}N_3O_3$ $[M+H]^+$ 476.2908, found 476.2902.

10n: White solid, yield: 82%; m.p. 75-77°C; ^{1}H NMR (500 MHz, Chloroform-*d*) δ 8.96 (d, *J* = 2.5 Hz, 1H, NO_2-C_6H_3), 8.44 (dd, *J* = 9.1, 2.5 Hz, 1H, NO_2-C_6H_3), 8.11 (d, *J* = 9.1 Hz, 1H, NO_2-C_6H_3), 5.87 (dd, *J* = 5.2, 2.5 Hz, 1H, H6), 3.75 (dq, *J* = 15.1, 5.7, 5.2 Hz, 1H, H20), 3.36 (d, *J* = 15.7 Hz, 1H), 2.87 (d, *J* = 15.7 Hz, 1H), 2.36 – 2.13 (m, 2H), 1.78 (s, 3H, CH_3), 1.74 – 1.69 (m, 3H), 1.64 (dd, *J* = 10.5, 6.0 Hz, 2H), 1.55 (dd, *J* = 12.9, 4.0 Hz, 2H), 1.41 (s, 3H, CH_3), 1.36 (d, *J* = 4.1 Hz, 2H), 1.33 (d, *J* = 4.5 Hz, 1H), 1.17 (d, *J* = 6.1 Hz, 3H, CH_3), 0.80 (s, 3H, CH_3), 0.76 (s, 3H, CH_3); HRMS (ESI) *m*/*z* calcd for $C_{29}H_{37}N_3O_3$ $[M+H]^+$ 476.2908, found 476.2902.

10o: White solid, yield: 81%; m.p. 228-230°C; ^{1}H NMR (500 MHz, Chloroform-*d*) δ 8.15 (d, *J* = 1.5 Hz, 1H, Cl-C_6H_2), 8.08 (d, *J* = 1.4 Hz, 1H, Cl-C_6H_2), 5.88 – 5.74 (m, 1H, H6), 3.75 (dq, *J* = 12.2, 6.2 Hz, 1H, H20), 3.27 (d, *J* = 15.5 Hz, 1H), 2.78 (d, *J* = 15.5 Hz, 1H), 2.30 – 2.10 (m, 2H), 1.80 – 1.73 (m, 1H), 1.71 (s, 3H, CH_3), 1.60 (qd, *J* = 11.5, 11.0, 5.9 Hz, 2H), 1.50 (td, *J* = 13.1, 3.9 Hz, 2H), 1.36 (s, 3H, CH_3), 1.32 – 1.27 (m, 2H), 1.25 (d, *J* = 7.3 Hz, 4H), 1.15 (d, *J* = 6.2 Hz, 3H, CH_3), 0.78 (s, 3H, CH_3), 0.72 (s, 3H, CH_3); HRMS (ESI) *m*/*z* calcd for $C_{29}H_{36}Cl_2N_2O$ $[M+H]^+$ 499.2278, found 499.2261.

2.7 *Compound **11a-11o** and **12a-12o***

11a: White solid, yield: 64%; m.p. 243-245°C; ^{1}H NMR (500 MHz, Chloroform-*d*) δ 9.67 (s, 1H, CONH), 9.21 (s, 1H, pyrazinyl), 7.74 (d, *J* = 8.0 Hz, 2H, C_6H_5), 7.39 (t, *J* = 7.8 Hz, 2H, C_6H_5), 7.16 (t, *J* = 7.4 Hz, 1H, C_6H_5), 5.84 (dd, *J* = 5.3, 2.4 Hz, 1H, H6), 3.23 (d, *J* = 15.9 Hz, 1H), 2.72 (d, *J* = 15.8 Hz, 1H), 2.57 (t, *J* = 9.1 Hz, 1H), 2.27 – 2.23 (m, 1H), 2.22 (d, *J* = 5.4 Hz,2H), 2.14 (s, 3H, CH_3), 2.11 (s, 2H), 1.84 – 1.73 (m, 2H), 1.69 (s, 3H, CH_3), 1.59 – 1.50 (m, 2H), 1.40 (d, *J* = 4.3 Hz, 2H), 1.38 (s, 3H, CH_3), 1.27 (s, 1H), 1.24 (s, 1H), 0.78 (s, 3H, CH_3), 0.65 (s, 3H, CH_3); ^{13}C NMR (125 MHz, $CDCl_3$) δ 209.41, 161.45, 157.52, 154.87, 148.14, 142.27, 141.01, 137.47, 129.24, 124.70, 121.24, 119.88, 63.67, 57.03, 48.80, 44.05, 44.00, 41.36, 38.84, 37.90, 33.98, 31.82, 31.74, 31.65, 31.4`8, 24.50, 22.94, 21.22, 20.55, 13.38; HRMS (ESI) *m*/*z* calcd for $C_{32}H_{39}N_3O_2$ $[M+H]^+$ 498.3115, found 498.3115.

11b: White solid, yield: 60%; m.p. 180-182°C; ^{1}H NMR (500 MHz, Chloroform-*d*) δ 9.63 (s, 1H, CONH), 9.21 (d, *J* = 1.9 Hz, 1H, pyrazinyl), 7.58 (s, 1H, 2-MeO-C_6H_4), 7.54 (d, *J* = 8.3 Hz, 1H, 2-MeO-C_6H_4), 7.34 – 7.20 (m, 1H,2- MeO-C_6H_4), 6.98 (d, *J* = 7.6 Hz, 1H, 2-MeO-C_6H_4), 5.84 (dd, *J* = 5.2, 2.5 Hz, 1H, H6), 3.23 (d, *J* = 15.9 Hz, 1H,

H20), 2.73 (d, *J* = 15.9 Hz, 1H), 2.57 (t, *J* = 9.1 Hz, 1H), 2.39 (s, 3H, OMe), 2.23 (ddd, *J* = 14.0, 6.9, 3.3 Hz, 2H), 2.14 (s, 3H, CH_3), 1.83 – 1.74 (m, 3H, CH_3), 1.70 (d, *J* = 1.9 Hz, 3H), 1.68 – 1.59 (m, 2H), 1.58 – 1.48 (m, 2H), 1.38 (s, 3H, CH_3), 1.27 (tq, *J* = 11.2, 5.6, 4.9 Hz, 4H), 0.79 (s, 3H, CH_3), 0.65 (s, 3H, CH_3). ^{13}C NMR (125 MHz, $CDCl_3$) δ 209.49, 161.44, 157.53, 154.83, 148.20, 142.36, 141.02, 139.21, 137.39, 129.10, 125.58, 121.25, 120.51, 117.02, 63.71, 57.07, 48.83, 44.08, 44.02, 41.38, 38.86, 37.92, 34.00, 31.84, 31.79, 31.68, 31.50, 24.52, 22.96, 21.65, 21.24, 20.57, 13.41; HRMS (ESI) *m/z* calcd for $C_{33}H_{41}N_3O_2$ $[M+H]^+$ 512.3272, found 512.3243.

11c: White solid, yield: 57%; m.p. 230-232°C; ^1H NMR (500 MHz, Chloroform-*d*) δ 9.62 (s, 1H, CONH), 9.21 (s, 1H, pyrazinyl), 7.59 (d, *J* = 1.9 Hz, 1H, 3-MeO-C_6H_4), 7.54 (dd, *J* = 8.1, 2.0 Hz, 1H, 3-MeO-C_6H_4), 7.28 (d, *J* = 7.8 Hz, 1H, 3-MeO-C_6H_4), 6.98 (d, *J* = 7.5 Hz, 1H, 3-MeO-C_6H_4), 5.85 (dd, *J* = 5.2, 2.5 Hz, 1H, H6), 3.24 (d, *J* = 15.8 Hz, 1H, H20), 2.73 (d, *J* = 15.9 Hz, 1H), 2.58 (t, *J* = 9.1 Hz, 1H), 2.39 (s, 3H, OMe), 2.29 – 2.21 (m, 2H), 2.14 (s, 3H, CH_3), 2.13 (d, *J* = 4.9 Hz, 2H), 1.85 – 1.76 (m, 2H), 1.70 (s, 3H, CH_3), 1.57 – 1.50 (m, 2H), 1.39 (s, 3H, CH_3), 1.33 – 1.22 (m, 4H), 0.79 (s, 3H, CH_3), 0.66 (s, 3H, CH_3); ^{13}C NMR (125 MHz, $CDCl_3$) δ 209.43, 161.45, 157.53, 154.84, 148.24, 142.39, 141.04, 139.22, 137.41, 129.10, 125.58, 121.26, 120.53, 117.04, 63.73, 57.10, 48.87, 44.10, 44.05, 41.41, 38.89, 37.95, 34.01, 31.87, 31.80, 31.68, 31.53, 24.54, 22.99, 21.66, 21.27, 20.59, 13.42; HRMS (ESI) *m/z* calcd for $C_{33}H_{41}N_3O_2$ $[M+H]^+$ 512.3272, found 512.3245.

11d: White solid, yield: 55%;m.p. 240-241°C; ^1H NMR (500 MHz, Chloroform-*d*) δ 9.61 (s, 1H, CONH), 9.20 (s, 1H, pyrazinyl), 7.62 (d, *J* = 8.3 Hz, 2H, 4-MeO-C_6H_4), 7.19 (d, *J* = 8.1 Hz, 2H, 4-MeO-C_6H_4), 5.84 (dd, *J* = 5.3, 2.4 Hz, 1H, H6), 3.23 (d, *J* = 15.9 Hz, 1H, H20), 2.72 (d, *J* = 15.8 Hz, 1H), 2.57 (t, *J* = 9.0 Hz, 1H), 2.34 (s, 3H, OMe), 2.24 (dt, *J* = 18.2, 5.6 Hz, 2H), 2.14 (s, 3H, CH_3), 2.12 (s, 2H), 1.79 (qd, *J* = 7.3, 6.6, 3.3 Hz, 3H), 1.69 (s, 3H, CH_3), 1.54 (t, *J* = 8.1 Hz, 2H), 1.38 (s, 3H, CH_3), 1.29 – 1.20 (m, 4H), 0.78 (s, 3H, CH_3), 0.65 (s, 3H, CH_3); ^{13}C NMR (125 MHz, $CDCl_3$) δ 209.42, 161.36, 157.49, 154.75, 148.25, 142.42, 141.01, 134.97, 134.39, 129.76, **129.76**, 121.23, 119.93, **119.93**, 63.72, 57.09, 48.86, 44.09, 44.04, 41.39, 38.88, 37.94, 33.99, 31.86, 31.78, 31.67, 31.52, 24.53, 22.98, 21.26, 21.07, 20.57, 13.41; HRMS (ESI) *m/z* calcd for $C_{33}H_{41}N_3O_2$ $[M+H]^+$ 512.3272, found 512.3251.

11e: White solid, yield: 48%; m.p. 215-217°C; ^1H NMR (500 MHz, Chloroform-*d*) δ 10.11 (d, *J* = 3.2 Hz, 1H, CONH), 9.19 (s, 1H, pyrazinyl), 8.57 (td, *J* = 8.1, 1.6 Hz, 1H, 2-F-C_6H_4), 7.22 – 7.18 (m, 1H, 2-F-C_6H_4), 7.15 (ddd, *J* = 10.7, 8.3, 1.6 Hz, 1H, 2-F-C_6H_4), 7.12 – 7.05 (m, 1H, 2-F-C_6H_4), 5.84 (dd, *J* = 5.2, 2.4 Hz, 1H, H6), 3.24 (d, *J* = 15.9 Hz, 1H), 2.73 (d, *J* = 15.8 Hz, 1H), 2.57 (t, *J* = 9.1 Hz, 1H), 2.33 – 2.19 (m, 2H), 2.14 (s, 3H, CH_3), 1.86 – 1.72 (m, 3H), 1.70 (s, 3H, CH_3), 1.59 – 1.51 (m, 2H), 1.39 (s, 3H, CH_3), 1.35 – 1.20 (m, 4H), 0.79 (s, 3H, CH_3), 0.65 (s, 3H, CH_3); ^{13}C NMR (125 MHz, $CDCl_3$) δ 209.42, 161.54, 157.82, 155.18, 153.81, 151.87, 148.14, 142.13, 140.91, 126.40, 126.33, 124.88, 124.85, 124.66, 124.60, 121.28, 121.22, 115.08, 114.93, 63.73, 63.58, 57.09, 48.87, 44.09, 44.07, 41.43, 38.89, 37.96, 33.99, 31.86, 31.67, 31.53, 29.82, 24.54, 23.00, 21.27, 20.59, 13.41; HRMS (ESI) *m/z* calcd for $C_{32}H_{38}FN_3O_2$ $[M+H]^+$ 516.3021, found 516.3012.

11f: White solid, yield: 37%; m.p. 278-280°C; ^1H NMR (500 MHz, Chloroform-*d*) δ 9.71 (s, 1H, CONH), 9.20 (s, 1H, pyrazinyl), 7.71 (dt, *J* = 10.8, 2.2 Hz, 1H, 3-F-C_6H_4), 7.37 (td, *J* = 7.1, 6.2, 3.1 Hz, 1H, 3-F-C_6H_4), 7.35 – 7.30 (m, 1H, 3-F-C_6H_4), 6.86 (td, *J* = 8.3, 2.4 Hz, 1H, 3-F-C_6H_4), 5.85 (dd, *J* = 5.2, 2.4 Hz, 1H, H6), 3.24 (d, *J* = 15.8 Hz, 1H), 2.73 (d, *J* = 15.8 Hz, 1H), 2.57 (t, *J* = 9.1 Hz, 1H), 2.25 (d, *J* = 5.4 Hz, 1H), 2.14 (d, *J* = 3.0 Hz, 3H, CH_3), 1.85 – 1.75 (m, 3H), 1.70 (s, 3H, CH_3), 1.62 (d, *J* = 12.6 Hz, 2H), 1.54 (d, *J* = 8.6 Hz, 3H), 1.38 (s, 3H, CH_3), 1.28 – 1.20 (m, 4H), 0.78 (s, 3H, CH_3), 0.66 (s, 3H, CH_3); ^{13}C NMR (125 MHz, $CDCl_3$) δ 209.40, 164.23, 161.60, 157.69, 155.26, 148.14, 141.95, 141.13, 139.06, 138.98, 130.40, 130.33, 121.35, 115.27, 115.25, 111.56, 111.39, 107.51, 107.30, 63.73, 57.10, 48.88, 44.10, 44.08, 41.42, 38.89, 37.96, 34.04, 31.87, 31.76, 31.67, 31.54, 24.55, 23.01, 21.28, 20.59, 13.42; HRMS (ESI) *m/z* calcd for $C_{32}H_{38}FN_3O_2$ $[M+H]^+$ 516.3021, found 516.3013.

11g: White solid, yield: 45%; m.p. 160-162°C; ^1H NMR (500 MHz, Chloroform-*d*) δ 9.13 (d, *J* = 1.3 Hz, 1H, CONH), 8.10 (t, *J* = 6.3 Hz, 1H, pyrazinyl), 7.36 – 7.31 (m, 2H, 4-F-C_6H_4), 7.02 (td, *J* = 8.6, 1.8 Hz, 2H, 4-F-C_6H_4), 5.79 (dd, *J* = 5.5, 2.4 Hz, 1H, H6), 4.65 (dq, *J* = 16.0, 8.8, 7.6 Hz, 2H), 3.20 (d, *J* = 15.9 Hz, 1H), 2.69 (d, *J* = 15.8 Hz, 1H), 2.56 (t, *J* = 9.0 Hz, 1H), 2.25 – 2.17 (m, 2H), 2.13 (d, *J* = 1.2 Hz, 3H, CH_3), 1.73 (dddd, *J* = 30.8, 17.6, 7.3, 3.4 Hz, 4H), 1.59 – 1.58 (s, 3H, CH_3), 1.56 – 1.49 (m, 2H), 1.32 – 1.30 (m, 3H, CH_3), 1.24 (ddt, *J* = 14.1, 7.9, 3.9 Hz, 3H), 0.76 (s, 3H, CH_3), 0.64 (d, *J* = 1.2 Hz, 3H, CH_3); ^{13}C NMR (125 MHz, $CDCl_3$) δ 209.36, 163.85, 163.30, 161.35, 157.63, 154.58, 148.32, 142.29, 140.87, 134.16, 134.13, 129.50, 129.44, 121.11, 115.78, 115.61, 63.71, 57.08, 48.86, 44.06, 44.02,

42.71, 41.36, 38.87, 37.88, 33.89, 31.84, 31.79, 31.64, 31.50, 24.51, 22.98, 21.23, 20.54, 13.38; HRMS (ESI) *m/z* calcd for $C_{32}H_{38}FN_3O_2$ $[M+H]^+$ 516.3021, found 516.3014.

11h: White solid, yield: 48%; m.p. 235-237°C; ^1H NMR (500 MHz, Chloroform-*d*) δ 10.58 (s, 1H, CONH), 9.18 (s, 1H, pyrazinyl), 8.66 (dd, *J* = 8.3, 1.4 Hz, 1H, 2-Cl-C_6H_4), 7.42 (d, *J* = 8.0 Hz, 1H, 2-Cl-C_6H_4), 7.33 (t, *J* = 7.8 Hz, 1H, 2-Cl-C_6H_4), 7.07 (t, *J* = 7.7 Hz, 1H, 2-Cl-C_6H_4), 5.84 (dd, *J* = 5.3, 2.5 Hz, 1H, H6), 3.23 (d, *J* = 15.9 Hz, 1H), 2.72 (d, *J* = 15.9 Hz, 1H), 2.56 (t, *J* = 9.0 Hz, 1H), 2.25 (s, 1H), 2.21 (q, *J* = 4.7 Hz, 2H), 2.13 (s, 3H, CH_3), 2.13 (s, 2H), 1.83 – 1.76 (m, 2H), 1.71 (s, 3H, CH_3), 1.53 (d, *J* = 8.5 Hz, 2H), 1.39 (s, 3H, CH_3), 1.33 (d, *J* = 10.6 Hz, 2H), 1.29 (s, 2H), 0.79 (s, 3H, CH_3), 0.65 (s, 3H, CH_3); ^{13}C NMR (125 MHz, $CDCl_3$) δ 209.37, 161.51, 157.76, 155.16, 148.07, 142.19, 140.88, 134.64, 129.28, 128.02, 124.80, 123.18, 121.27, 120.84, 63.70, 57.07, 48.90, 48.86, 44.06, 41.46, 38.87, 37.94, 33.98, 31.84, 31.80, 31.64, 31.51, 24.51, 22.98, 21.25, 20.60, 13.39; HRMS (ESI) *m/z* calcd for $C_{32}H_{38}ClN_3O_2$ $[M+H]^+$ 532.2725, found 532.2728.

11i: White solid, yield: 45%;m.p. 180-182°C; ^1H NMR (500 MHz, Chloroform-*d*) δ 9.68 (s, 1H, CONH), 9.20 (s, 1H, pyrazinyl), 7.83 (d, *J* = 2.0 Hz, 1H, 3-Cl-C_6H_4), 7.61 (dd, *J* = 8.0, 2.0 Hz, 1H, 3-Cl-C_6H_4), 7.31 (t, *J* = 8.1 Hz, 1H, 3-Cl-C_6H_4), 7.13 (dd, *J* = 7.9, 1.9 Hz, 1H, 3-Cl-C_6H_4), 5.85 (dd, *J* = 5.3, 2.4 Hz, 1H, H6), 3.24 (d, *J* = 15.9 Hz, 1H), 2.73 (d, *J* = 15.8 Hz, 1H), 2.57 (t, *J* = 9.0 Hz, 1H), 2.24 (dt, *J* = 18.7, 5.9 Hz, 2H), 2.14 (s, 3H, CH_3), 1.76 (ddd, *J* = 18.8, 8.0, 3.9 Hz, 3H), 1.70 (s, 3H, CH_3), 1.67 – 1.58 (m, 2H), 1.57 – 1.52 (m, 2H), 1.38 (s, 3H, CH_3), 1.30 (dd, *J* = 11.7, 6.9 Hz, 2H), 1.25 – 1.24 (m, 2H), 0.78 (s, 3H, CH_3), 0.65 (s, 3H, CH_3); ^{13}C NMR (125 MHz, $CDCl_3$) δ 209.43, 161.58, 157.69, 155.27, 148.08, 141.89, 141.11, 138.64, 134.93, 130.28, 124.76, 121.34, 119.96, 117.91, 63.71, 57.06, 48.83, 44.08, 44.06, 41.41, 38.86, 37.93, 34.03, 31.84, 31.77, 31.68, 31.51, 24.53, 22.98, 21.26, 20.58, 13.41; HRMS (ESI) *m/z* calcd for $C_{32}H_{38}ClN_3O_2$ $[M+H]^+$ 532.2725, found 532.2728.

11j: White solid, yield: 44%;m.p. 239-240°C; ^1H NMR (500 MHz, Chloroform-*d*) δ 9.66 (s, 1H, CONH), 9.18 (s, 1H, pyrazinyl), 7.69 (d, *J* = 8.4 Hz, 2H, 4-Cl-C_6H_4), 7.41 – 7.28 (m, 2H, 4-Cl-C_6H_4), 5.83 (dd, *J* = 5.2, 2.4 Hz, 1H, H6), 3.23 (d, *J* = 15.8 Hz, 1H), 2.72 (d, *J* = 15.9 Hz, 1H), 2.56 (t, *J* = 9.0 Hz, 1H), 2.25 (d, *J* = 5.3 Hz, 1H), 2.23 – 2.18 (m, 1H), 2.13 (s, 3H, CH_3), 2.11 (d, *J* = 5.3 Hz, 1H), 1.79 (dd, *J* = 10.2, 2.6 Hz, 1H), 1.74 (d, *J* = 3.0 Hz, 1H), 1.68 (s, 3H, CH_3), 1.53 (d, *J* = 7.6 Hz, 2H), 1.37 (s, 3H, CH_3), 1.26 (dq, *J* = 21.6, 6.2, 5.5 Hz, 4H), 0.77 (s, 3H, CH_3), 0.64 (s, 3H, CH_3); ^{13}C NMR (125 MHz, $CDCl_3$) δ 209.34, 161.49, 157.64, 155.14, 148.09, 141.98, 141.04, 136.10, 129.65, 129.26, 121.30, 121.11, 63.68, 57.04, 48.82, 44.05, 44.03, 41.38, 38.84, 37.91, 33.99, 31.82, 31.74, 31.64, 31.49, 24.51, 22.97, 21.24, 20.55, 13.38; HRMS (ESI) *m/z* calcd for $C_{32}H_{38}ClN_3O_2$ $[M+H]^+$ 532.2725, found 532.2728.

11k: White solid, yield: 58%; m.p. 260-262°C; ^1H NMR (500 MHz, Chloroform-*d*) δ 10.54 (s, 1H, CONH), 9.19 (s, 1H, pyrazinyl), 8.58 (dd, *J* = 8.0, 1.7 Hz, 1H, 2-MeO-C_6H_4), 7.10 (td, *J* = 7.8, 1.7 Hz, 1H, 2-MeO-C_6H_4), 7.03 (td, *J* = 7.7, 1.3 Hz, 1H, 2-MeO-C_6H_4), 6.94 (dd, *J* = 8.1, 1.3 Hz, 1H, 2-MeO-C_6H_4), 5.85 (dd, *J* = 5.3, 2.5 Hz, 1H, H6), 3.96 (s, 3H, OMe), 3.23 (d, *J* = 15.8 Hz, 1H), 2.73 (d, *J* = 15.8 Hz, 1H), 2.58 (t, *J* = 9.0 Hz, 1H), 2.27 (d, *J* = 5.4 Hz, 1H), 2.23 – 2.19 (m, 1H), 2.15 (s, 3H, CH_3), 2.14 – 2.08 (m, 1H), 1.85 – 1.76 (m, 2H), 1.72 (s, 3H, CH_3), 1.68 – 1.61 (m, 2H), 1.60 – 1.49 (m, 2H), 1.39 (s, 3H, CH_3), 1.33 – 1.15 (m, 4H), 0.80 (s, 3H, CH_3), 0.66 (s, 3H, CH_3); ^{13}C NMR (125 MHz, $CDCl_3$) δ 209.45, 161.34, 157.52, 154.55, 148.72, 148.33, 142.86, 140.78, 127.61, 124.21, 121.43, 121.18, 119.63, 110.32, 63.76, 57.13, 56.15, 48.90, 44.12, 44.02, 41.42, 38.92, 38.02, 34.01, 31.90, 31.69, 31.56, 31.48, 24.56, 23.00, 21.29, 20.61, 13.43; HRMS (ESI) *m/z* calcd for $C_{33}H_{41}N_3O_3$ $[M+H]^+$ 528.3221, found 528.3185.

11l: White solid, yield: 46%; m.p. 213-215°C; ^1H NMR (500 MHz, Chloroform-*d*) δ 9.66 (s, 1H, CONH), 9.19 (s, 1H, pyrazinyl), 7.54 (t, *J* = 2.2 Hz, 1H, 3-MeO-C_6H_4), 7.28 (d, *J* = 8.1 Hz, 1H, 3-MeO-C_6H_4), 7.17 (dd, *J* = 8.0, 1.9 Hz, 1H, 3-MeO-C_6H_4), 6.71 (dd, *J* = 8.2, 2.4 Hz, 1H, 3-MeO-C_6H_4), 5.84 (dd, *J* = 5.2, 2.5 Hz, 1H, H6), 3.84 (s, 3H, OMe), 3.23 (d, *J* = 15.9 Hz, 1H), 2.72 (d, *J* = 15.8 Hz, 1H), 2.56 (t, *J* = 9.1 Hz, 1H), 2.30 – 2.23 (m, 1H), 2.23 – 2.17 (m, 2H), 2.13 (s,3H, CH_3), 2.11 (d, *J* = 2.6 Hz, 2H), 1.83 – 1.74 (m, 2H), 1.69 (s, 3H, CH_3), 1.63 – 1.58 (m, 1H), 1.53 (d, *J* = 8.2 Hz, 2H), 1.38 (s, 3H, CH_3), 1.24 (d, *J* = 3.8 Hz, 4H), 0.78 (s, 3H, CH_3), 0.65 (s, 3H, CH_3); ^{13}C NMR (125 MHz, $CDCl_3$) δ 209.37, 161.47, 160.41, 157.55, 154.93, 148.16, 142.23, 141.00, 138.69, 129.89, 121.25, 112.09, 110.66, 105.55, 63.69, 57.05, 55.48, 48.83, 44.05, 44.02, 41.38, 38.85, 37.91, 33.98, 31.83, 31.74, 31.64, 31.50, 24.51, 22.97, 21.24, 20.56, 13.38; HRMS (ESI) *m/z* calcd for $C_{33}H_{41}N_3O_3$ $[M+H]^+$ 528.3221, found 528.3220.

11m: White solid, yield: 31%; m.p. 270-272°C; ^1H NMR (500 MHz, Chloroform-*d*) δ 9.56 (s, 1H, CONH), 9.18 (s, 1H, pyrazinyl), 7.69 – 7.57 (m, 2H, 4-MeO-C_6H_4), 6.95 – 6.85 (m, 2H, 4-MeO-C_6H_4), 5.82 (dd, *J* = 5.4, 2.5 Hz, 1H, H6), 3.79 (s, 3H, OMe), 3.21 (d, *J* = 15.8 Hz, 1H), 2.70 (d, *J* = 15.8 Hz, 1H), 2.55 (t, *J* = 9.1 Hz, 1H), 2.24 (d,

J = 5.8 Hz, 1H), 2.20 – 2.16 (m, 2H), 2.12 (s, 3H, CH_3), 1.84 – 1.74 (m, 2H), 1.67 (s, 3H, CH_3), 1.64 – 1.57 (m, 2H), 1.55 – 1.47 (m, 2H), 1.36 (s, 3H, CH_3), 1.31 – 1.18 (m, 4H), 0.77 (s, 3H, CH_3), 0.63 (s, 3H, CH_3); ^{13}C NMR (125 MHz, $CDCl_3$) δ 209.35, 161.18, 157.44, 156.67, 154.63, 148.18, 142.39, 140.87, 130.66, 121.51, 121.17, 114.36, 63.64, 57.00, 55.56, 48.78, 44.01, 41.32, 38.80, 37.86, 33.92, 31.79, 31.72, 31.60, 31.45, 24.47, 22.92, 21.19, 20.51, 13.34; HRMS (ESI) *m*/*z* calcd for $C_{33}H_{41}N_3O_3$ $[M+H]^+$ 528.3220, found 528.3223.

11n: White solid, yield: 50%; m.p. 165 -167°C; ^{1}H NMR (500 MHz, Chloroform-*d*) δ 9.08 (d, *J* = 1.9 Hz, 1H, pyrazinyl), 7.85 (t, *J* = 5.9 Hz, 1H, CONH), 7.32 (t, *J* = 7.7 Hz, 2H, beta-phenethyl), 7.28 – 7.25 (m, 3H beta-phenethyl), 5.80 (dd, *J* = 5.2, 2.5 Hz, 1H, H6), 3.74 (q, *J* = 6.6 Hz, 2H), 3.24 – 3.08 (m, 1H), 2.94 (t, *J* = 6.8 Hz, 2H), 2.67 (d, *J* = 15.8 Hz, 1H), 2.57 (t, *J* = 9.0 Hz, 1H), 2.22 (dt, *J* = 18.1, 5.7 Hz, 2H), 2.14 (s, 3H, CH_3), 1.76 (q, *J* = 8.6, 7.5 Hz, 4H), 1.69 – 1.65 (m, 1H), 1.61 (dd, *J* = 10.6, 5.6 Hz, 1H), 1.53 (s, 3H, CH_3), 1.42 (d, *J* = 7.6 Hz, 1H), 1.28 (s, 3H, CH_3), 1.25 (s, 4H), 0.75 (s, 3H, CH_3), 0.65 (d, *J* = 1.9 Hz, 3H, CH_3); ^{13}C NMR (125 MHz, $CDCl_3$) δ 209.53, 163.64, 157.45, 154.29, 148.34, 142.43, 140.61, 138.90, 128.99, **128.99,** 128.87, **128.87,** 126.76, 121.06, 63.72, 57.08, 48.82, 44.08, 43.97, 41.29, 40.49, 38.87, 37.87, 35.91, 33.79, 31.85, 31.76, 31.69, 31.49, 24.52, 22.94, 21.22, 20.55, 13.40; HRMS (ESI) *m*/*z* calcd for $C_{34}H_{43}N_3O_2$ $[M+H]^+$ 526.3428, found 526.3414.

11o: White solid, yield: 51%; m.p. 110-112°C; ^{1}H NMR (500 MHz, Chloroform-*d*) δ 9.04 (d, *J* = 2.8 Hz, 1H, pyrazinyl), 7.65 (d, *J* = 8.5 Hz, 1H, CONH), 5.83 – 5.65 (m, 1H, H6), 3.94 (tdd, *J* = 10.8, 7.4, 4.0 Hz, 1H, H1'' of cyclohexyl), 3.14 (dd, *J* = 15.8, 2.6 Hz, 1H), 2.64 (d, *J* = 15.8 Hz, 1H), 2.53 (t, *J* = 9.1 Hz, 1H), 2.22 – 2.14 (m, 2H), 2.09 (s, 3H, CH_3), 2.07 (s, 1H), 1.97 (dt, *J* = 12.9, 3.8 Hz, 2H), 1.82 – 1.65 (m, 6H), 1.63 – 1.60 (m, 1H), 1.58 (s, 3H, CH_3), 1.45 – 1.35 (m, 3H), 1.29 (s, 3H, CH_3), 1.27 – 1.14 (m, 6H), 0.72 (d, *J* = 2.4 Hz, 3H, CH_3), 0.60 (d, *J* = 2.7 Hz, 3H, CH_3); ^{13}C NMR (125 MHz, $CDCl_3$) δ 209.28, 162.68, 157.21, 154.00, 148.34, 142.63, 140.61, 120.96, 63.62, 57.00, 48.77, 47.95, 43.99, 43.89, 41.22, 38.80, 37.81, 33.84, 33.02, 31.78, 31.67, 31.58, 31.44, 25.62, 24.78, 24.45, 22.89, 21.15, 20.45, 13.32; HRMS (ESI) *m*/*z* calcd for $C_{32}H_{45}N_3O_2$ $[M+Na]^+$ 527.3482, found 527.3415.

12a: White solid, yield: 96%; m.p. 168-170°C; ^{1}H NMR (500 MHz, Chloroform-*d*) δ 9.68 (s, 1H, CONH), 9.21 (d, *J* = 3.5 Hz, 1H, pyrazinyl), 7.75 (d, *J* = 7.9 Hz, 2H, C_6H_5), 7.40 (t, *J* = 7.9 Hz, 2H, C_6H_5), 7.17 (t, *J* = 7.4 Hz, 1H, C_6H_5), 5.84 (dd, *J* = 5.2, 2.5 Hz, 1H, H6), 3.75 (tt, *J* = 10.4, 5.2 Hz, 1H, H20), 3.25 (d, *J* = 15.9 Hz, 1H), 2.72 (d, *J* = 15.9 Hz, 1H), 2.27 – 2.15 (m, 2H), 1.81 – 1.72 (m, 2H), 1.70 (s, 3H, CH_3), 1.62 (dd, *J* = 10.6, 5.5 Hz, 2H), 1.57 – 1.51 (m, 2H), 1.39 (s, 3H, CH_3), 1.36 – 1.30 (m, 2H), 1.26 (d, *J* = 3.6 Hz, 4H), 1.17 (d, *J* = 6.1 Hz, 3H, CH_3), 0.80 (s, 6H, 2 × CH_3); HRMS (ESI) *m*/*z* calcd for $C_{32}H_{41}N_3O_2$ $[M+H]^+$ 500.3271, found 500.3235.

12b: White solid, yield: 95%; m.p. 110-111; ^{1}H NMR (500 MHz, Chloroform-*d*) δ 9.91 (s, 1H, CONH), 9.21 (d, *J* = 3.3 Hz, 1H, pyrazinyl), 8.35 (d, *J* = 8.1 Hz, 1H, 2-MeO-C_6H_4), 7.29 (t, *J* = 7.8 Hz, 1H, 2-MeO-C_6H_4), 7.23 (d, *J* = 7.6 Hz, 1H, 2-MeO-C_6H_4), 7.09 (t, *J* = 7.5 Hz, 1H, 2-MeO-C_6H_4), 5.84 (dd, *J* = 5.3, 2.6 Hz, 1H, H6), 3.75 (dd, *J* = 10.1, 5.8 Hz, 1H, H20), 3.26 (d, *J* = 15.9 Hz, 1H), 2.72 (d, *J* = 16.0 Hz, 1H), 2.43 (s, 3H, OMe), 2.33 – 2.15 (m, 2H), 2.04 (s, 1H), 1.84 – 1.71 (m, 3H, CH_3), 1.70 (d, *J* = 8.2 Hz, 3H), 1.65 – 1.57 (m, 2H), 1.55 – 1.51 (m, 1H), 1.46 (d, *J* = 11.4 Hz, 3H), 1.39 (s, 3H, CH_3), 1.30 – 1.22 (m, 2H), 1.17 (d, *J* = 6.1 Hz, 3H, CH_3), 0.81 (d, *J* = 5.9 Hz, 6H, 2 × CH_3); HRMS (ESI) *m*/*z* calcd for $C_{33}H_{43}N_3O_2$ $[M+Na]^+$ 536.3248, found 536.3204.

12c: White solid, yield: 94%; m.p. 170-172°C; ^{1}H NMR (500 MHz, DMSO-*d*$_6$) δ 10.17 (s, 1H, CONH), 9.00 (s, 1H, pyrazinyl), 7.84 – 7.50 (m, 2H, 3-MeO-C_6H_4), 7.28 (t, *J* = 7.8 Hz, 1H, 3-MeO-C_6H_4), 6.98 (d, *J* = 7.5 Hz, 1H, 3-MeO-C_6H_4), 6.04 – 5.65 (m, 1H, H6), 4.12 (d, *J* = 5.5 Hz, 1H), 3.50 (dt, *J* = 9.6, 5.8 Hz, 1H, H20), 3.10 (d, *J* = 15.9 Hz, 1H), 2.75 (d, *J* = 15.9 Hz, 1H), 2.34 (s, 3H, OMe), 2.26 – 2.08 (m, 2H), 1.71 (s, 3H), 1.60 (d, *J* = 7.2 Hz, 3H, CH_3), 1.54 (dd, *J* = 10.5, 5.5 Hz, 1H), 1.45 (dd, *J* = 13.0, 3.7 Hz, 1H), 1.39 (s, 3H, CH_3), 1.23 (ddd, *J* = 21.6, 16.8, 6.3 Hz, 3H, CH_3), 1.16 – 1.08 (m, 2H), 1.01 (d, *J* = 6.0 Hz, 3H, CH_3), 0.73 (d, *J* = 3.8 Hz, 6H, 2 × CH_3); HRMS (ESI) *m*/*z* calcd for $C_{33}H_{43}N_3O_2$ $[M+H]^+$ 514.34280, found 514.3398.

12d: White solid, yield: 95%; m.p. 223-225°C; ^{1}H NMR (500 MHz, Chloroform-*d*) δ 9.62 (s, 1H, CONH), 9.20 (s, 1H, pyrazinyl), 7.63 (d, *J* = 8.2 Hz, 2H, 4-MeO-C_6H_4), 7.20 (d, *J* = 8.1 Hz, 2H, 4-MeO-C_6H_4), 5.84 (dd, *J* = 5.3, 2.5 Hz, 1H, H6), 3.75 (dt, *J* = 11.8, 5.9 Hz, 1H, H20), 3.24 (d, *J* = 15.9 Hz, 1H), 2.71 (d, *J* = 15.8 Hz, 1H), 2.35 (s, 3H, CH_3), 2.21 (ddt, *J* = 16.0, 10.9, 4.9 Hz, 2H), 1.78 (td, *J* = 8.9, 7.6, 2.4 Hz, 1H), 1.70 (s, 3H, CH_3), 1.63 (tt, *J* = 10.6, 5.3 Hz, 2H), 1.54 (dd, *J* = 13.1, 3.8 Hz, 2H), 1.45 (d, *J* = 12.3 Hz, 1H), 1.38 (s, 3H), 1.35 – 1.28 (m, 2H), 1.25 (s, 2H, CH_3),

1.17 (d, J = 6.2 Hz, 3H, CH_3), 0.79 (s, 6H, 2 × CH_3); HRMS (ESI) *m/z* calcd for $C_{33}H_{43}N_3O_2$ $[M+H]^+$ 514.34280, found 514.3395.

12e: White solid, yield: 84%; m.p. 180-182°C; ^1H NMR (500 MHz, Chloroform-*d*) δ 10.13 (d, J = 3.2 Hz, 1H, CONH), 9.19 (s, 1H, pyrazinyl), 8.57 (d, J = 1.6 Hz, 1H, 2-F-C_6H_4), 7.20 (t, J = 7.8 Hz, 1H, 2-F-C_6H_4), 7.15 (ddd, J = 10.7, 8.3, 1.6 Hz, 1H, 2-F-C_6H_4), 7.12 – 7.07 (m, 1H, 2-F-C_6H_4), 5.84 (dd, J = 5.3, 2.5 Hz, 1H, H6), 3.75 (dt, J = 9.9, 6.0 Hz, 1H, H20), 3.25 (d, J = 15.9 Hz, 1H), 2.72 (d, J = 15.9 Hz, 1H), 2.25 – 2.18 (m, 2H), 2.02 (dtd, J = 20.7, 13.6, 7.8 Hz, 1H), 1.80 – 1.72 (m, 2H), 1.70 (s, 3H, CH_3), 1.62 (dt, J = 10.6, 5.4 Hz, 2H), 1.39 (s, 3H, CH_3), 1.25 (s, 3H, CH_3), 1.17 (d, J = 6.1 Hz, 4H), 0.99 (d, J = 6.7 Hz, 3H, CH_3), 0.80 (s, 6H, 2 × CH_3); ^{19}F NMR (471 MHz, $CDCl_3$) δ -131.78. HRMS (ESI) *m/z* calcd for $C_{32}H_{40}FN_3O_2$ $[M+Na]^+$ 540.2997, found 540.2974.

12f: White solid, yield: 75%; m.p. 130-132°C; ^1H NMR (500 MHz, Chloroform-*d*) δ 9.72 (s, 1H, CONH), 9.20 (s, 1H, pyrazinyl), 7.71 (dt, J = 10.8, 2.2 Hz, 1H, 3-F-C_6H_4), 7.44 – 7.31 (m, 2H, 3-F-C_6H_4), 6.93 – 6.79 (m, 1H, 3-F-C_6H_4), 5.84 (dd, J = 5.2, 2.5 Hz, 1H, H6), 3.75 (ddd, J = 12.3, 8.0, 4.5 Hz, 1H, H20), 3.25 (d, J = 16.0 Hz, 1H), 2.72 (d, J = 16.0 Hz, 1H), 2.30 – 2.12 (m, 2H), 1.82 – 1.75 (m, 2H), 1.70 (s, 3H), CH_3, 1.65 – 1.59 (m, 2H), 1.57 – 1.50 (m, 2H), 1.38 (s, 3H, CH_3), 1.34 (d, J = 10.1 Hz, 2H), 1.27 – 1.23 (m, 4H), 1.16 (dd, J = 6.1, 2.6 Hz, 3H, CH_3), 0.79 (d, J = 3.8 Hz, 6H, 2 × CH_3); HRMS (ESI) *m/z* calcd for $C_{32}H_{40}FN_3O_2$ $[M+H]^+$ 518.3177, found 518.3142.

12g: White solid, yield: 81%; m.p. 218-220°C; ^1H NMR (500 MHz, Chloroform-*d*) δ 9.12 (s, 1H, CONH), 8.10 (t, J = 6.3 Hz, 1H, pyrazinyl), 7.42 – 7.30 (m, 2H, 4-F-C_6H_4), 7.15 – 6.94 (m, 2H, 4-F-C_6H_4), 5.79 (dd, J = 5.3, 2.5 Hz, 1H, H6), 3.74 (dq, J = 9.7, 6.1 Hz, 1H, H20), 3.22 (d, J = 15.9 Hz, 1H), 2.67 (d, J = 15.9 Hz, 1H), 2.27 – 2.15 (m, 2H), 1.72 – 1.65 (m, 4H), 1.59 (s, 3H, CH_3), 1.51 (td, J = 13.1, 4.0 Hz, 2H), 1.41 – 1.34 (m, 2H), 1.31 (s, 3H, CH_3), 1.25 (d, J = 2.3 Hz, 4H), 1.16 (d, J = 6.1 Hz, 3H, CH_3), 0.77 (d, J = 4.9 Hz, 6H, 2 × CH_3); HRMS (ESI) *m/z* calcd for $C_{32}H_{40}FN_3O_2$ $[M+H]^+$ 518.3177, found 518.3143.

12h: White solid, yield: 85%; m.p. 242-244°C; ^1H NMR (500 MHz, Chloroform-*d*) δ 10.60 (s, 1H, CONH), 9.18 (s, 1H, pyrazinyl), 8.67 (dd, J = 8.2, 1.5 Hz, 1H, 2-Cl-C_6H_4), 7.43 (dd, J = 8.0, 1.4 Hz, 1H, 2-Cl-C_6H_4), 7.34 (td, J = 7.9, 1.4 Hz, 1H, 2-Cl-C_6H_4), 7.08 (td, J = 7.7, 1.5 Hz, 1H, 2-Cl-C_6H_4), 5.84 (dd, J = 5.3, 2.5 Hz, 1H, H6), 3.74 (qd, J = 6.2, 3.2 Hz, 1H, H20), 3.26 (d, J = 16.0 Hz, 1H), 2.72 (d, J = 16.0 Hz, 1H), 2.21 (ddd, J = 14.8, 8.2, 3.9 Hz, 2H), 1.81 – 1.75 (m, 1H), 1.72 (s, 3H, CH_3), 1.70 – 1.66 (m, 3H), 1.65 – 1.59 (m, 3H), 1.39 (s, 3H, CH_3), 1.34 (s, 1H), ^1H NMR (500 MHz, Chloroform-*d*) δ 1.25 (d, J = 4.8 Hz, 4H), 1.17 (d, J = 6.2 Hz, 3H, CH_3), 0.80 (s, 3H, CH_3), 0.79 (s, 3H, CH_3); HRMS (ESI) *m/z* calcd for $C_{32}H_{40}ClN_3O_2$ $[M+Na]^+$ 556.2701, found 556.2659.

12i: White solid, yield: 84%;m.p. 308-310°C; ^1H NMR (500 MHz, Chloroform-*d*) δ 9.69 (s, 1H, CONH), 9.21 (s, 1H, pyrazinyl), 7.84 (t, J = 2.0 Hz, 1H, 3-Cl-C_6H_4), 7.69 – 7.50 (m, 1H, 3-Cl-C_6H_4), 7.32 (t, J = 8.1 Hz, 2H, 3-Cl-C_6H_4), 5.85 (dd, J = 5.3, 2.5 Hz, 1H, H6), 3.75 (dt, J = 9.7, 6.0 Hz, 1H, H20), 3.25 (d, J = 15.9 Hz, 1H), 2.73 (d, J = 16.0 Hz, 1H), 2.30 – 2.11 (m, 2H), 1.78 (ddd, J = 18.0, 10.5, 2.4 Hz, 1H), 1.71 (s, 3H), 1.66 – 1.59 (m, 2H), 1.58 – 1.51 (m, 4H), 1.39 (s, 3H), 1.35 (d, J = 3.2 Hz, 1H), 1.27 – 1.24 (m, 4H), 1.17 (d, J = 6.1 Hz, 3H, , CH_3), 0.81 (s, 3H, CH_3), 0.80 (s, 3H, CH_3); HRMS (ESI) *m/z* calcd for $C_{32}H_{40}ClN_3O_2$ $[M+H]^+$ 534.2882, found 534.2815.
^1H NMR (500 MHz, Chloroform-*d*) δ 1.27 – 1.24 (m, 1H), 1.17 (d, J = 6.1 Hz, 1H).

12j: White solid, yield: 88%; m.p. 276-277°C °C; ^1H NMR (500 MHz, Chloroform-*d*) δ 9.67 (s, 1H, CONH), 9.18 (s, 1H, pyrazinyl), 7.91 – 7.62 (m, 2H, 4-Cl-C_6H_4), 7.35 (d, J = 8.8 Hz, 2H, 4-Cl-C_6H_4), 5.84 (dd, J = 5.3, 2.5 Hz, 1H, H6), 3.84 – 3.63 (m, 1H, H20), 3.25 (d, J = 15.9 Hz, 1H), 2.71 (d, J = 15.9 Hz, 1H), 2.21 (dt, J = 18.0, 5.4 Hz, 2H), 1.82 – 1.73 (m, 2H), 1.69 (s, 3H), 1.62 (dd, J = 10.6, 5.5 Hz, 1H), 1.57 – 1.45 (m, 2H), 1.38 (s, 3H, CH_3), 1.34 (d, J = 4.1 Hz, 1H), 1.30 (dd, J = 12.4, 7.7 Hz, 1H), ^1H NMR (500 MHz, Chloroform-*d*) δ 1.25 (d, J = 3.4 Hz, 4H), 1.16 (d, J = 6.1 Hz, 3H, CH_3), 0.78 (d, J = 2.5 Hz, 6H, 2 × CH_3); HRMS (ESI) *m/z* calcd for $C_{32}H_{40}ClN_3O_2$ $[M+H]^+$ 534.2882, found 534.2853.

12k: White solid, yield: 93%; m.p. 257-259°C; ^1H NMR (500 MHz, Chloroform-*d*) δ 10.55 (s, 1H, CONH), 9.18 (t, J = 2.5 Hz, 1H, pyrazinyl), 8.58 (dt, J = 8.0, 1.6 Hz, 1H, 2-MeO-C_6H_4), 7.09 (tt, J = 7.7, 1.6 Hz, 1H, 2-MeO-C_6H_4), 7.03 (td, J = 7.7, 1.6 Hz, 1H, 2-MeO-C_6H_4), 6.94 (dd, J = 8.2, 1.5 Hz, 1H, 2-MeO-C_6H_4), 5.84 (dd, J = 5.3, 2.5 Hz, 1H, H6), 3.96 (d, J = 1.3 Hz, 3H, OMe), 3.80 – 3.68 (m, 1H, H20), 3.24 (d, J = 15.9 Hz, 1H), 2.72 (d, J = 15.9 Hz, 1H), 2.21 (dq, J = 16.4, 5.2 Hz, 2H), 1.78 (td, J = 9.0, 7.6, 2.3 Hz, 2H), 1.72 (s, 3H, CH_3), 1.66 – 1.61 (m, 2H), 1.50 –

1.43 (m, 2H), 1.39 (s, 3H, CH_3), 1.35 – 1.28 (m, 4H), 1.17 (d, *J* = 6.1 Hz, 4H, CH_3), 0.83 – 0.75 (m, 6H, 2 × CH_3); HRMS (ESI) *m*/*z* calcd for $C_{33}H_{43}N_3O_3$ $[M+Na]^+$ 552.3197, found 552.3145.

12l: White solid, yield: 94%; m.p. 217-219°C; ^1H NMR (500 MHz, Chloroform-*d*) δ 9.68 (s, 1H, CONH), 9.20 (d, *J* = 3.6 Hz, 1H, pyrazinyl), 7.56 (t, *J* = 2.2 Hz, 1H, 3-MeO-C_6H_4), 7.29 (d, *J* = 8.1 Hz, 1H, 3-MeO-C_6H_4), 7.18 (dd, *J* = 7.9, 1.9 Hz, 1H, 3-MeO-C_6H_4), 6.73 (dd, *J* = 8.3, 2.4 Hz, 1H, 3-MeO-C_6H_4), 5.84 (dd, *J* = 5.4, 2.5 Hz, 1H, H6), 3.86 (s, 3H, OMe), 3.75 (dt, *J* = 11.7, 5.7 Hz, 1H, H20), 3.25 (d, *J* = 15.9 Hz, 1H), 2.72 (d, *J* = 15.9 Hz, 1H), 2.21 (ddt, *J* = 15.7, 10.5, 4.9 Hz, 2H), 1.77 (ddd, *J* = 17.8, 10.3, 2.5 Hz, 2H), 1.70 (s, 3H, CH_3), 1.61 (ddd, *J* = 13.9, 9.8, 4.6 Hz, 2H), 1.56 (d, *J* = 3.9 Hz, 4H), 1.38 (s, 3H, CH_3), 1.35 – 1.30 (m, 2H), 1.25 (t, *J* = 2.7 Hz,4H), 1.17 (d, *J* = 6.2 Hz, 3H, CH_3), 0.79 (s, 6H, 2 × CH_3); HRMS (ESI) *m*/*z* calcd for $C_{33}H_{43}N_3O_3$ $[M+H]^+$ 530.3377, found 530.3330.

12m: White solid, yield: 96%; m.p. 210-212°C; ^1H NMR (500 MHz, Chloroform-*d*) δ 9.57 (s, 1H, CONH), 9.18 (d, *J* = 1.7 Hz, 1H, pyrazinyl), 7.88 – 7.44 (m, 2H, 4-MeO-C_6H_4), 6.93 (d, *J* = 8.8 Hz, 2H, 4-MeO-C_6H_4), 5.83 (dd, *J* = 5.3, 2.5 Hz, 1H, H6), 3.82 (s, 3H, OMe), 3.75 (dq, *J* = 11.7, 5.9 Hz, 1H, H20), 3.24 (d, *J* = 15.9 Hz, 1H), 2.70 (d, *J* = 15.9 Hz, 1H), 2.21 (dt, *J* = 18.2, 5.3 Hz, 2H), 1.77 (td, *J* = 9.0, 7.6, 2.5 Hz, 2H), 1.69 (s, 3H, CH_3), 1.61 (dd, *J* = 10.6, 5.6 Hz, 2H), 1.52 (td, *J* = 13.1, 4.1 Hz, 2H), 1.37 (s, 3H, CH_3), 1.34 (d, *J* = 4.1 Hz, 1H), 1.31 – 1.28 (m, 1H), 1.25 (d, *J* = 6.7 Hz, 4H), 1.17 (d, *J* = 6.1 Hz, 3H, CH_3), 0.78 (s, 6H, 2 × CH_3); HRMS (ESI) *m*/*z* calcd for $C_{33}H_{43}N_3O_3$ $[M+H]^+$ 530.3377, found 530.3370.

12n: White solid, yield: 90%; m.p. 203-205°C; ^1H NMR (500 MHz, Chloroform-*d*) δ 9.08 (d, *J* = 2.7 Hz, 1H, pyrazinyl), 7.85 (t, *J* = 6.2 Hz, 1H, CONH), 7.33 (t, *J* = 7.5 Hz, 2H, beta-phenethyl), 7.28 (d, *J* = 1.7 Hz, 2H, beta-phenethyl), 7.24 (d, *J* = 7.6 Hz, 1H, beta-phenethyl), 5.80 (dd, *J* = 5.3, 2.5 Hz, 1H), 3.75 (q, *J* = 6.6 Hz, 2H), 3.25 – 3.17 (m, 1H), 2.95 (t, *J* = 6.9 Hz, 2H), 2.66 (d, *J* = 15.8 Hz, 1H), 2.23 – 2.15 (m, 2H), 1.73 – 1.70 (m, 2H), 1.61 (dd, *J* = 10.6, 5.5 Hz, 2H), 1.54 (s, 3H, CH_3), 1.36 (d, *J* = 5.6 Hz, 2H), 1.29 (s, 3H, CH_3), 1.26 (s, 6H), 1.16 (d, *J* = 6.2 Hz, 3H, CH_3), 0.79 (s, 3H, CH_3), 0.77 (s, 3H, CH_3), 1.16 (d, *J* = 6.2 Hz, 4H), 0.79 (s, 3H), 0.77 (s, 3H); HRMS (ESI) *m*/*z* calcd for $C_{34}H_{45}N_3O_2$ $[M+H]^+$ 528.3585, found 528.3555.

12o: White solid, yield: 91%; m.p. 201-203°C; ^1H NMR (500 MHz, Chloroform-*d*) δ 9.05 (d, *J* = 10.1 Hz, 1H, pyrazinyl), 7.67 (d, *J* = 8.5 Hz, 1H, CONH), 5.78 (dd, *J* = 5.2, 2.5 Hz, 1H, H6), 3.96 (tdd, *J* = 10.1, 7.2, 4.0 Hz, 1H, H1'' of cyclohexyl), 3.71 (dt, *J* = 12.2, 6.1 Hz, 1H, H20), 3.20 (d, *J* = 16.0 Hz, 1H), 2.64 (d, *J* = 15.9 Hz, 1H), 2.27 – 2.11 (m, 3H), 1.99 (dt, *J* = 13.2, 4.3 Hz, 2H), 1.80 – 1.62 (m, 8H), 1.60 (s, 3H, CH_3), 1.59 – 1.50 (m, 2H), 1.46 – 1.38 (m, 3H), 1.31 (s, 3H, CH_3), 1.25 – 1.19 (m, 6H), 1.15 (d, *J* = 6.1 Hz, 3H, CH_3), 0.75 (s, 6H, 2 × CH_3); HRMS (ESI) *m*/*z* calcd for $C_{32}H_{47}N_3O_2$ $[M+H]^+$ 506.3741, found 506.3734.

3. The NMR Spectra of Compounds **2-12**

3.1. The ^{1}H NMR and ^{13}C NMR Spectra of Compound ***2-4***

Compound **2**

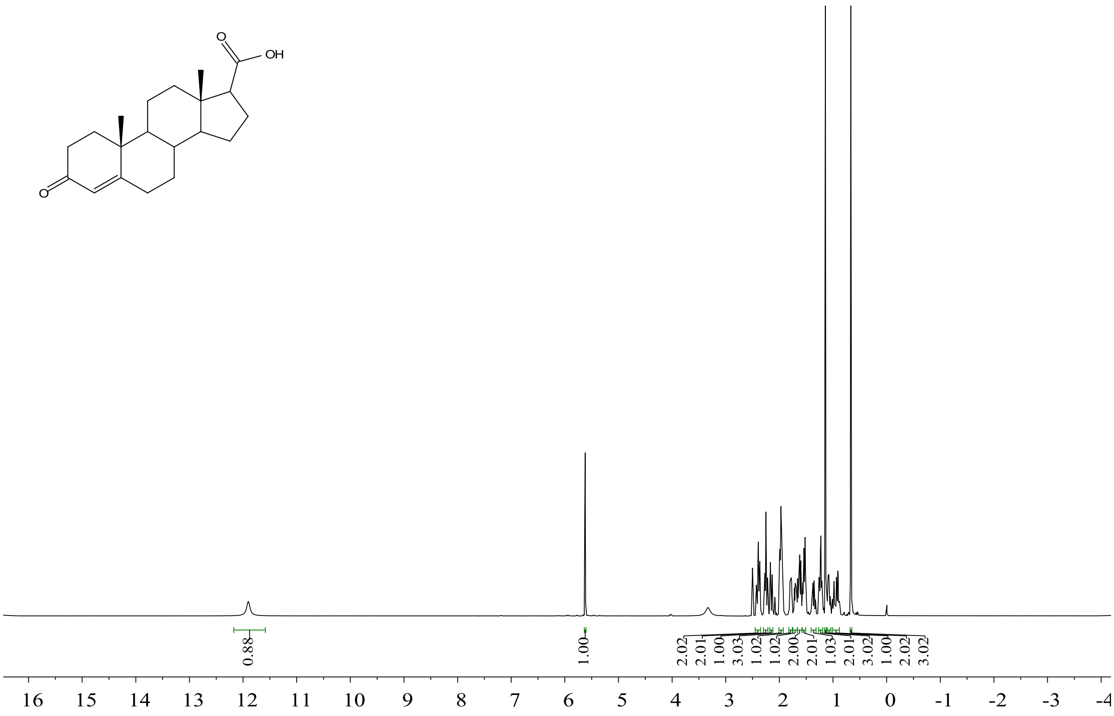

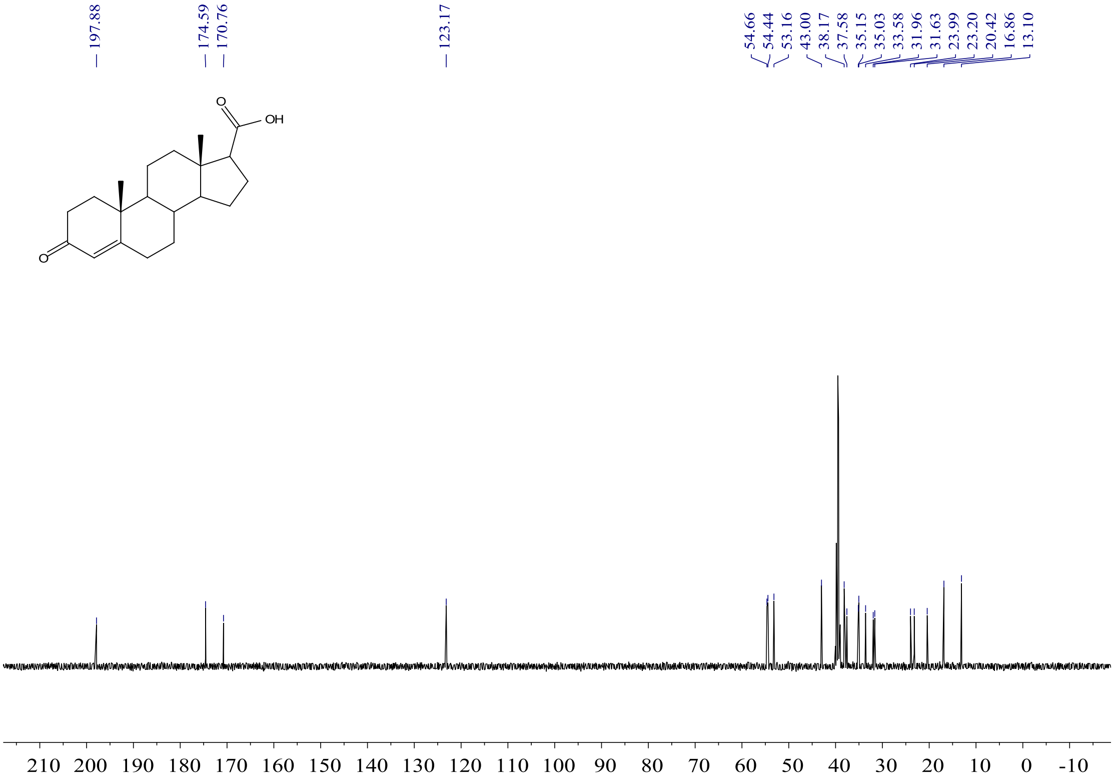

Compound **3**

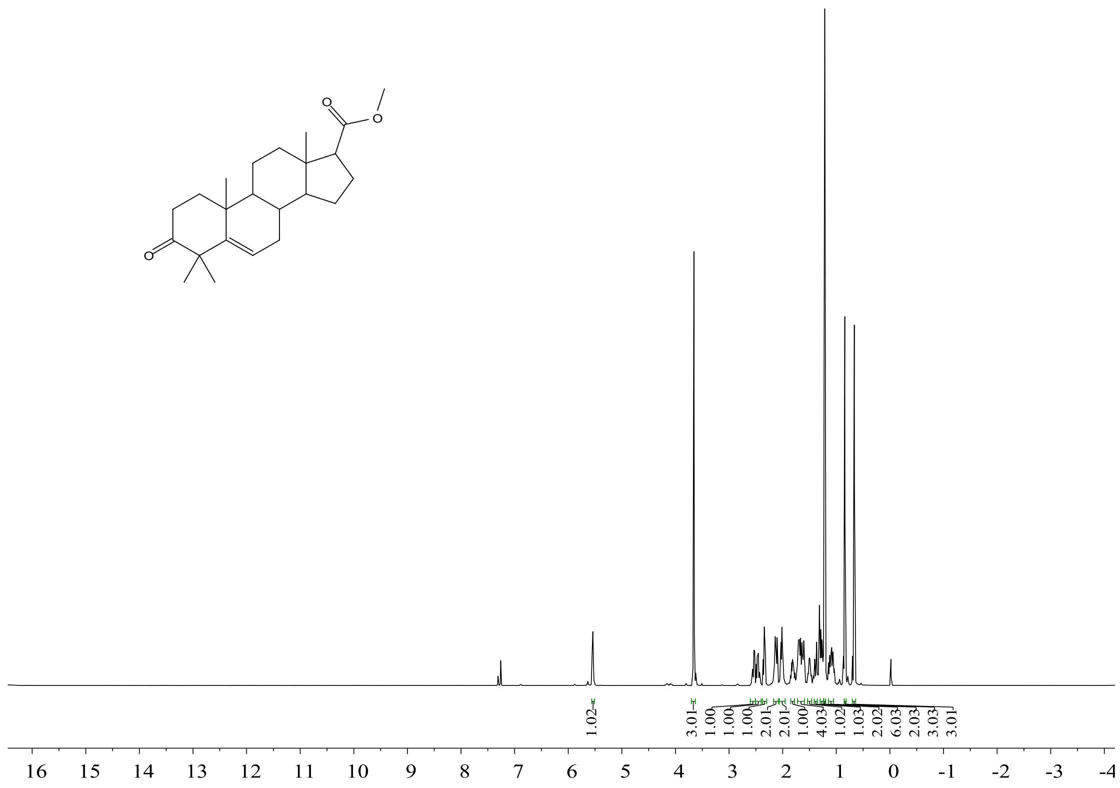

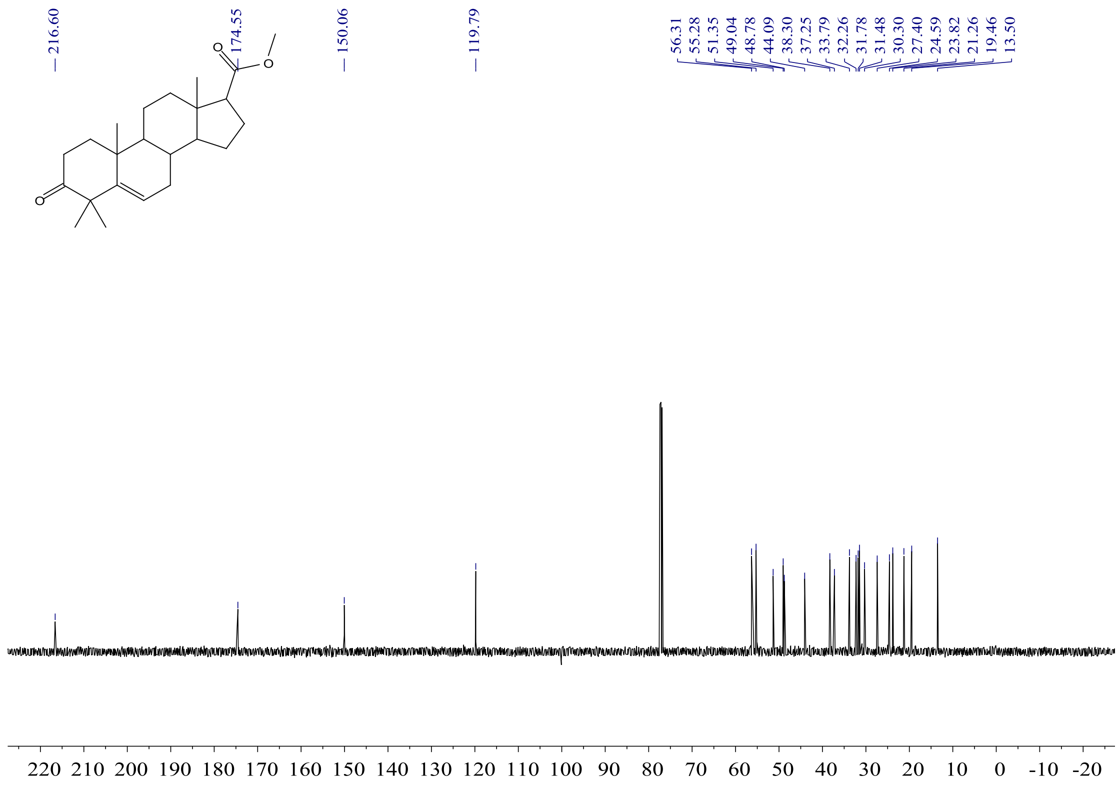

Compound **4**

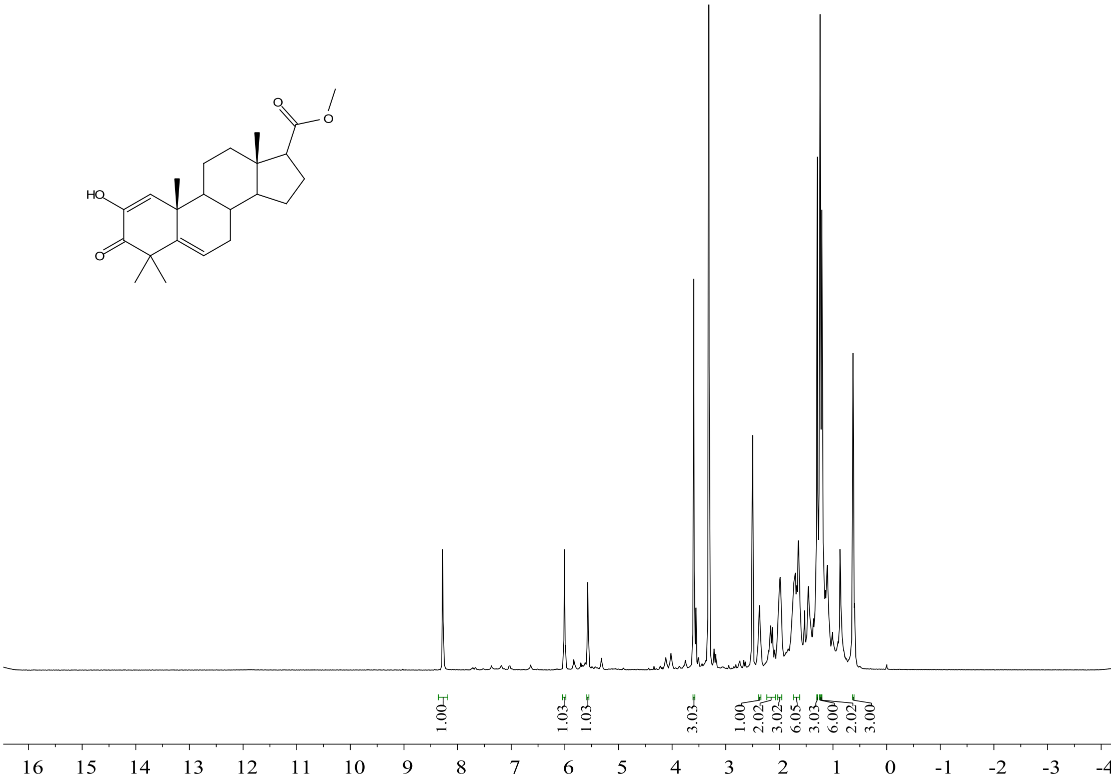

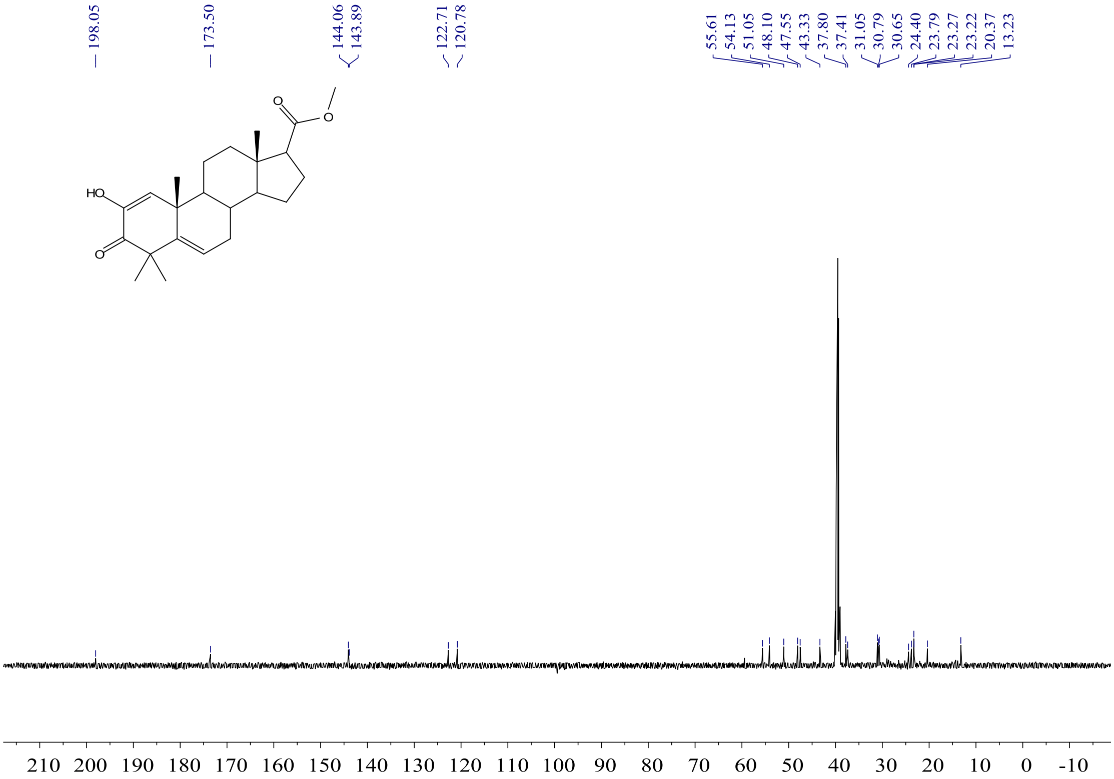

3.2. The ^{1}H NMR and ^{13}C NMR Spectra of Compound **5a-5f**

Compound **5a**

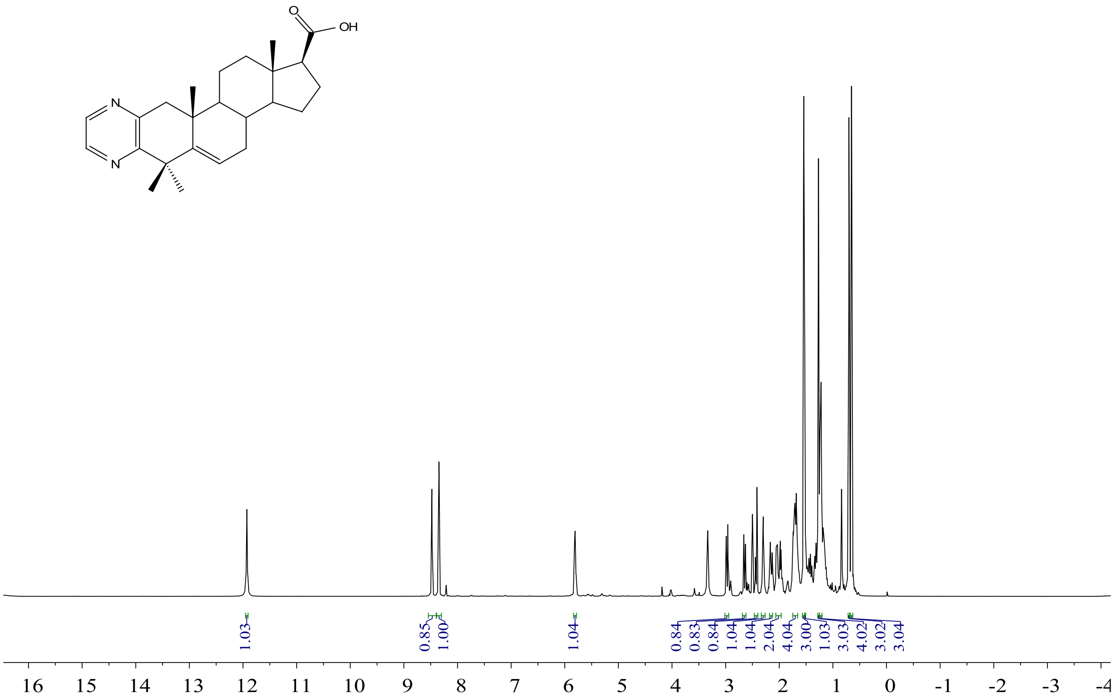
O
OH
N
N
1.03
0.85
1.00
1.04
0.84
0.83
0.84
1.04
1.04
2.04
4.04
3.00
1.03
3.03
4.02
3.02
3.04
16 15 14 13 12 11 10 9 8 7 6 5 4 3 2 1 0 -1 -2 -3 -4

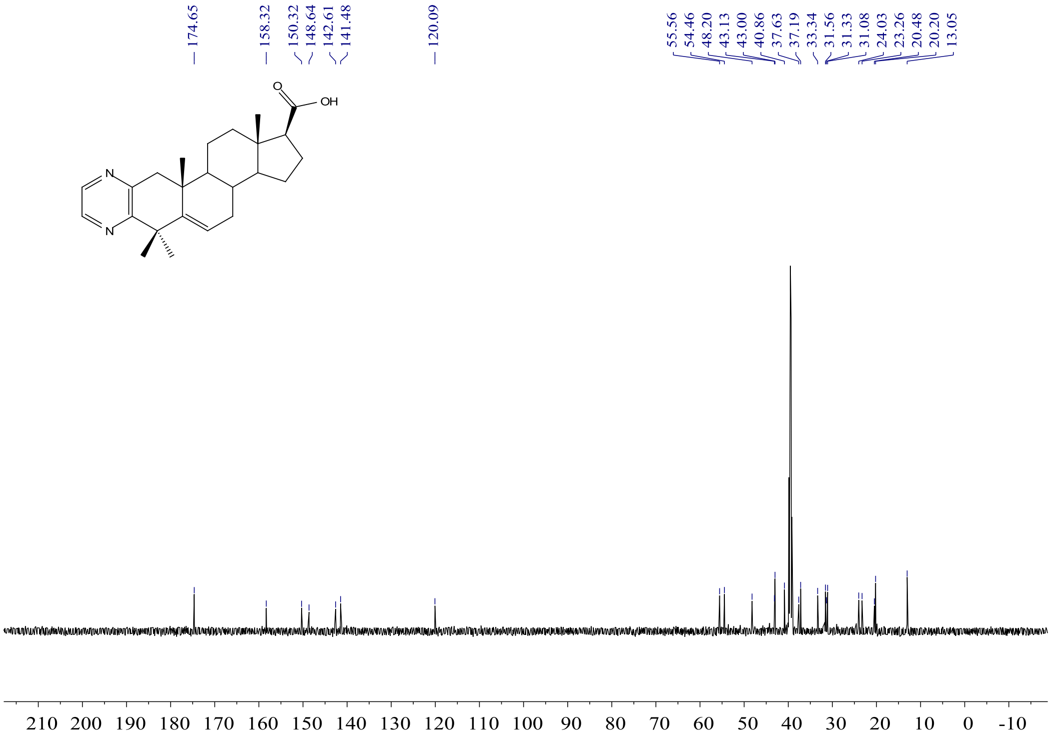
174.65
158.32
150.32
148.64
142.61
141.48
120.09
55.56
54.46
48.20
43.13
43.00
40.86
37.63
37.19
33.34
31.56
31.33
31.08
24.03
23.26
20.48
20.20
13.05
O
OH
N
N
210 200 190 180 170 160 150 140 130 120 110 100 90 80 70 60 50 40 30 20 10 0 -10

Compound **5b**

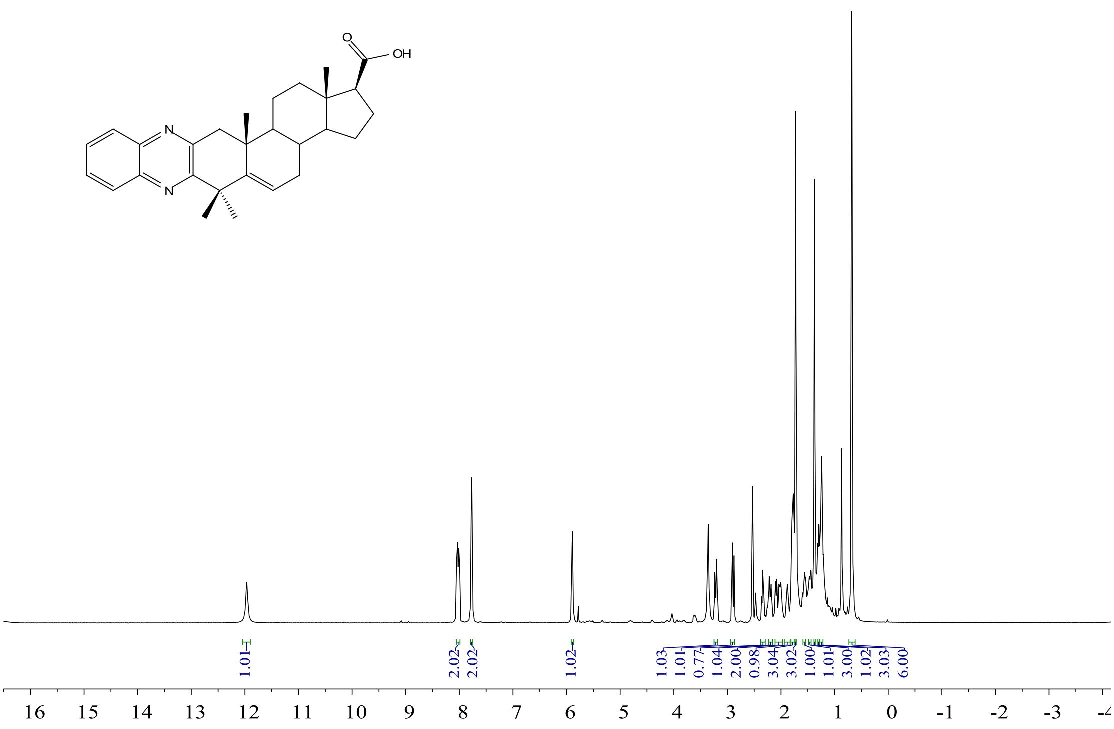

O
OH
N
N
1.01
2.02
2.02
1.02
1.03
1.01
0.77
1.04
2.00
0.98
3.04
3.02
1.00
1.01
3.00
1.02
3.03
6.00
16 15 14 13 12 11 10 9 8 7 6 5 4 3 2 1 0 -1 -2 -3

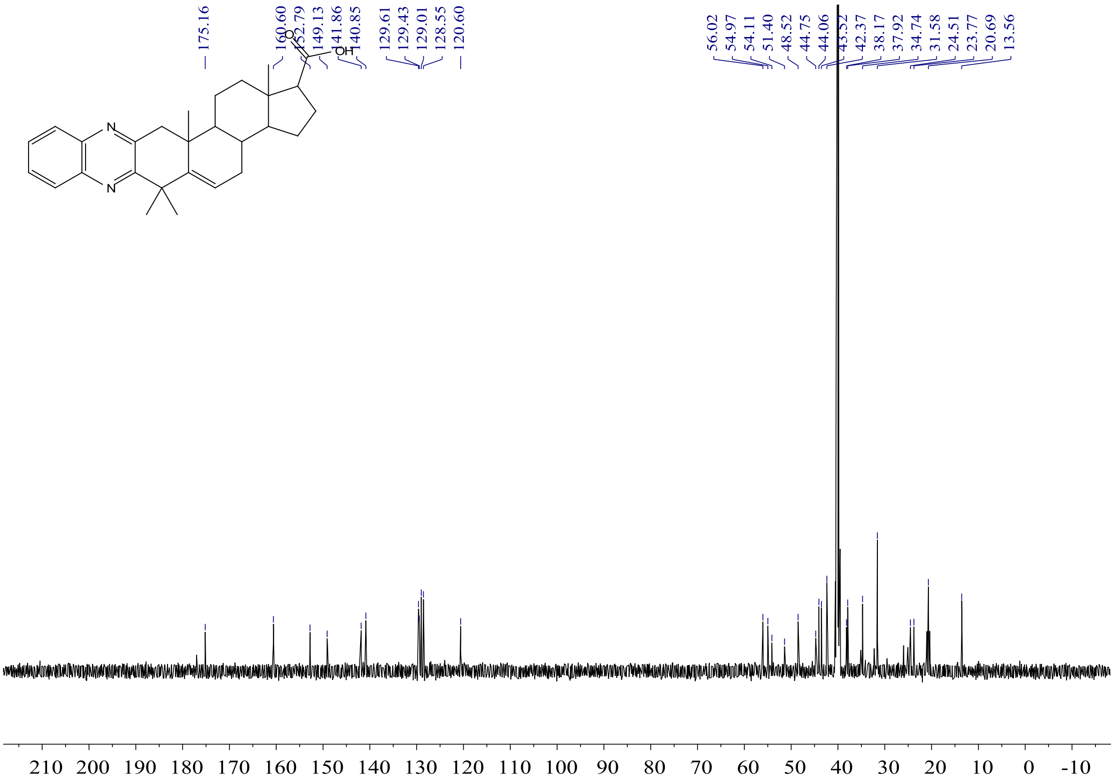

175.16
160.60
152.79
149.13
141.86
140.85
129.61
129.43
129.01
128.55
120.60
56.02
54.97
54.11
51.40
48.52
44.75
44.06
43.52
42.37
38.17
37.92
34.74
31.58
24.51
23.77
20.69
13.56
O
OH
N
N
210 200 190 180 170 160 150 140 130 120 110 100 90 80 70 60 50 40 30 20 10 0 -10

Compound **5c**

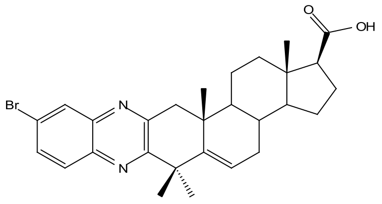
Br
N
N
O
OH

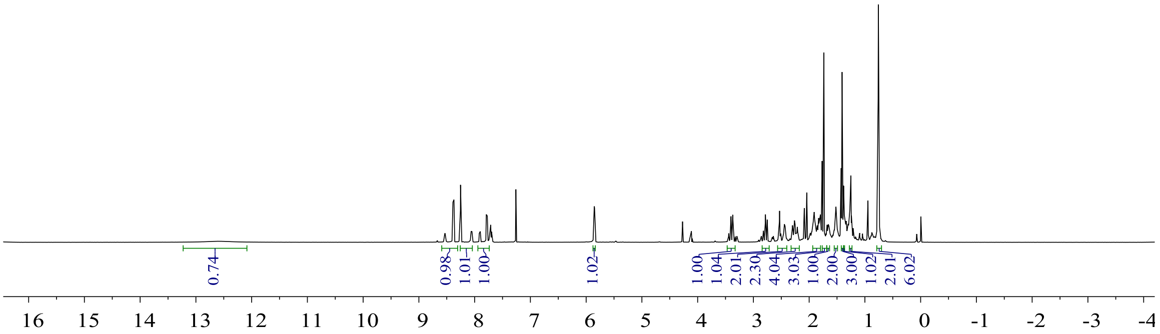
0.74
0.98
1.01
1.00
1.02
1.00
1.04
2.01
2.30
4.04
3.03
1.00
2.00
3.00
1.02
2.01
6.02
16 15 14 13 12 11 10 9 8 7 6 5 4 3 2 1 0 -1 -2 -3 -4

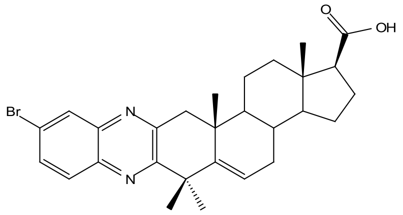
Br
N
N
O
OH

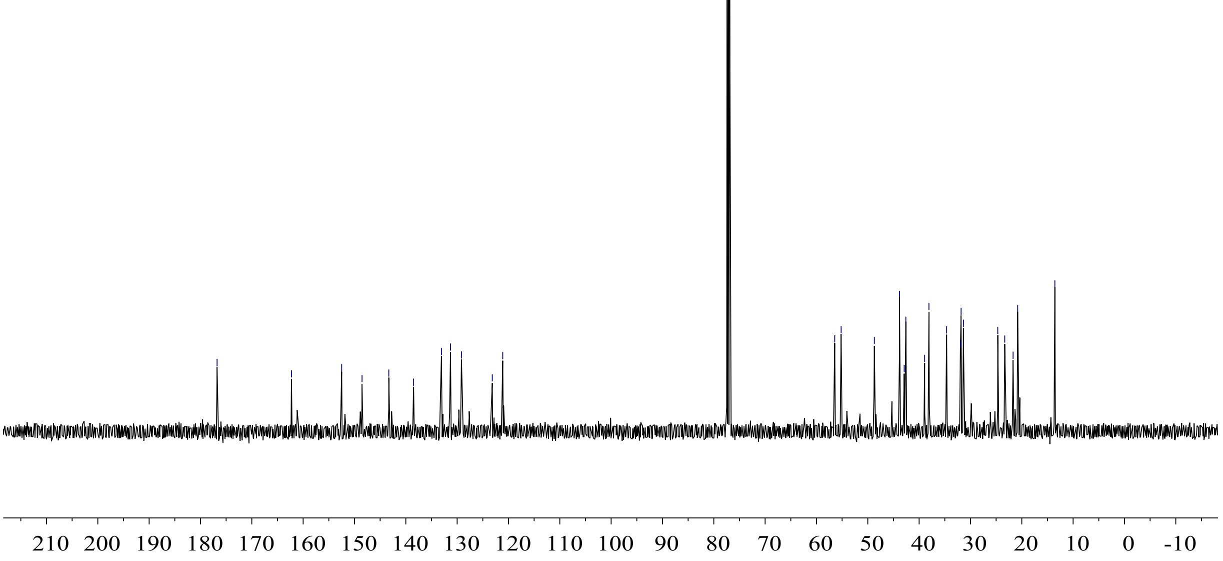
176.77
162.28
152.49
148.53
143.31
138.48
133.06
131.29
129.16
123.15
121.12
56.44
55.19
48.72
43.83
42.91
42.59
38.93
38.09
34.64
31.96
31.82
31.37
24.68
23.32
21.69
20.80
13.56
210 200 190 180 170 160 150 140 130 120 110 100 90 80 70 60 50 40 30 20 10 0 -10

Compound **5d**

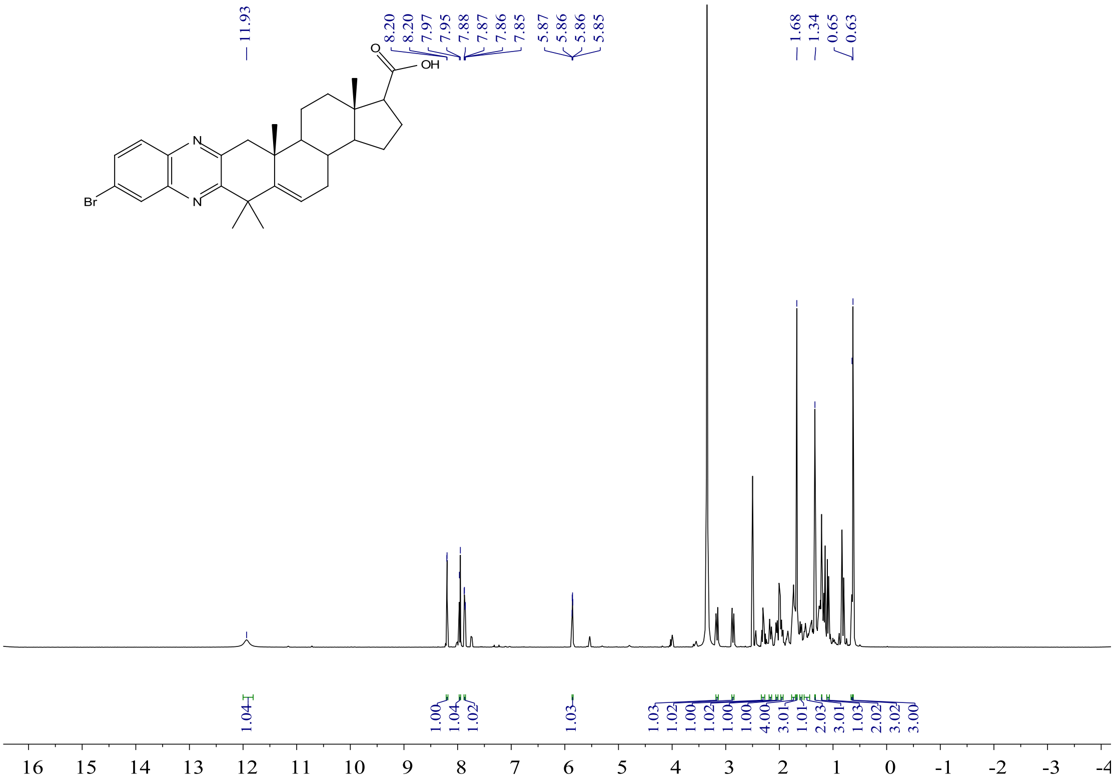

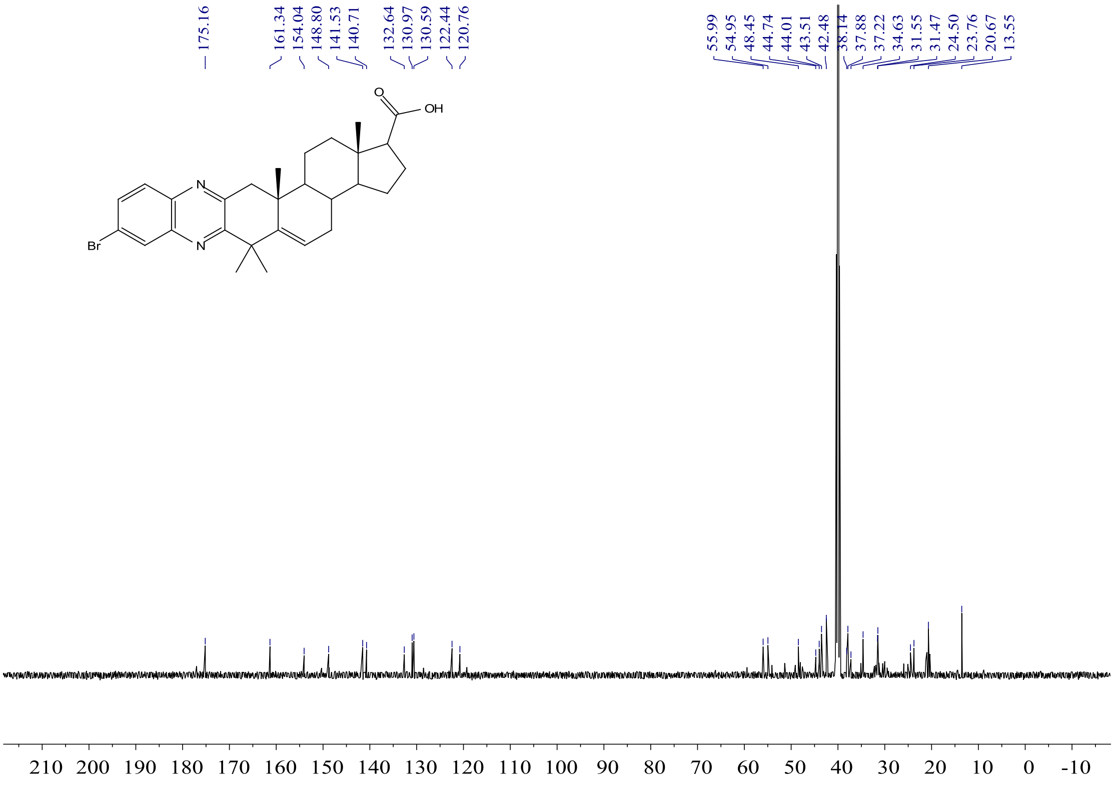

Compound **5e**

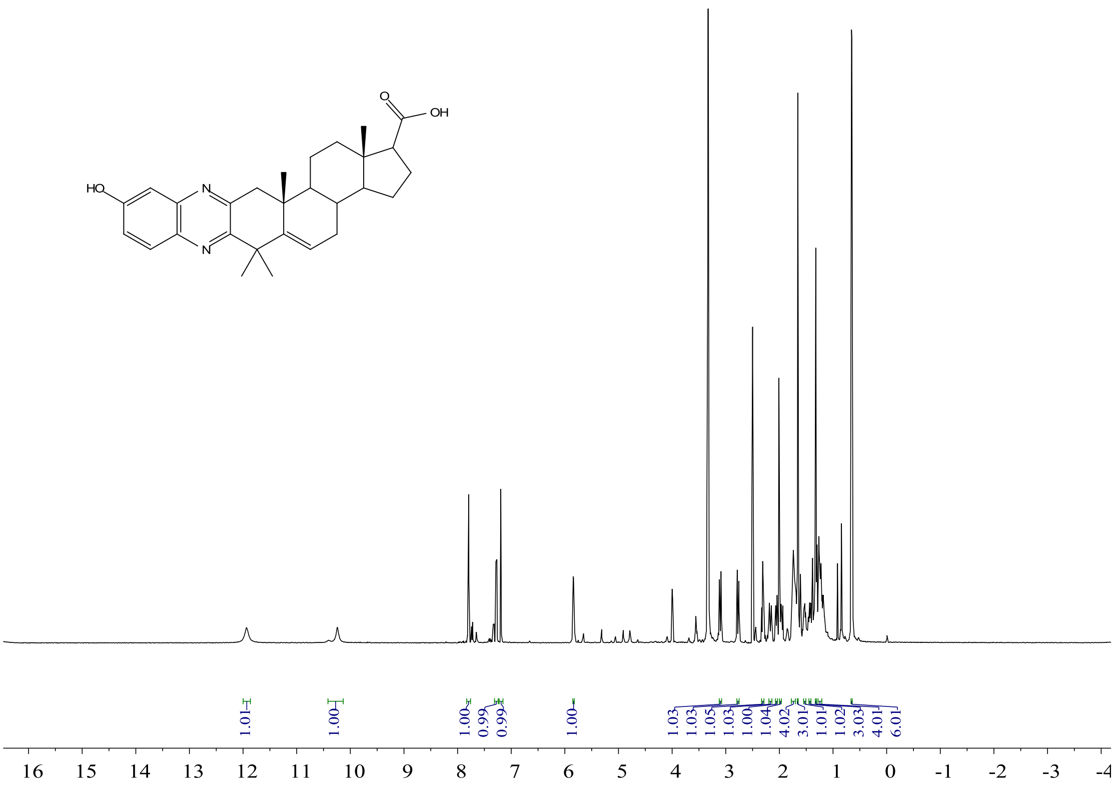
O
OH
HO
N
N
1.01
1.00
1.00
0.99
0.99
1.00
1.03
1.03
1.05
1.03
1.00
1.04
4.02
3.01
1.01
1.02
3.03
4.01
6.01
16 15 14 13 12 11 10 9 8 7 6 5 4 3 2 1 0 -1 -2 -3 -4

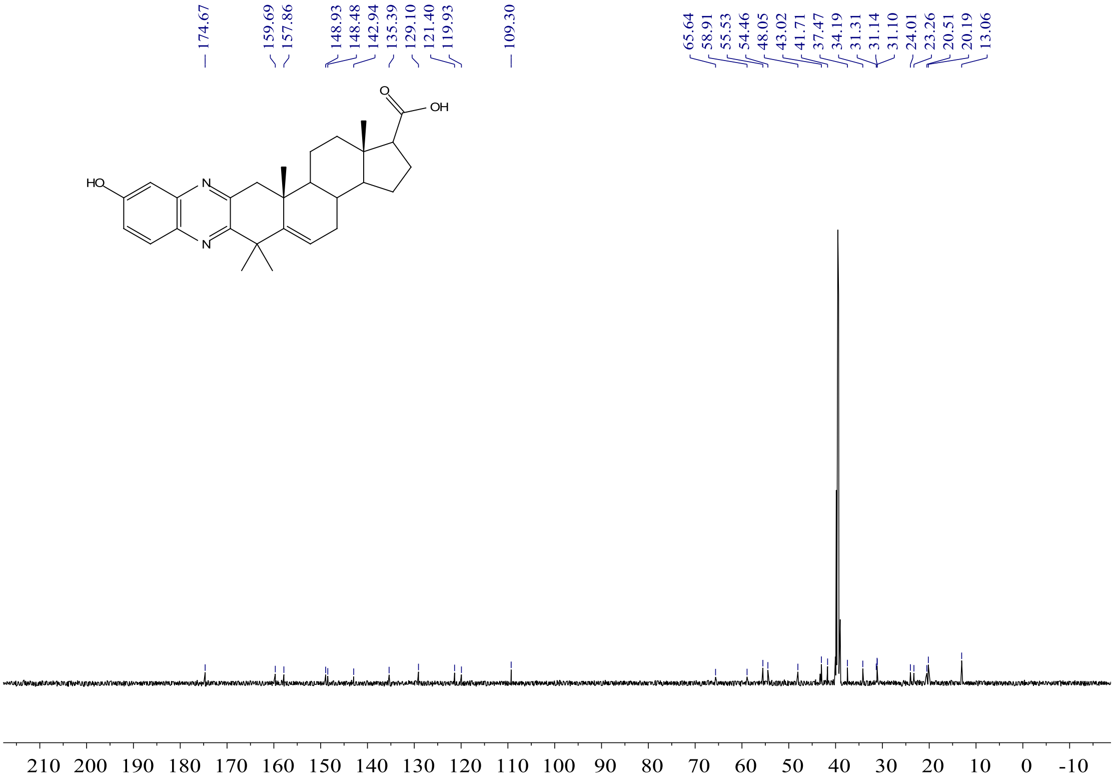
174.67
159.69
157.86
148.93
148.48
142.94
135.39
129.10
121.40
119.93
109.30
65.64
58.91
55.53
54.46
48.05
43.02
41.71
37.47
34.19
31.31
31.14
31.10
24.01
23.26
20.51
20.19
13.06
O
OH
HO
N
N
210 200 190 180 170 160 150 140 130 120 110 100 90 80 70 60 50 40 30 20 10 0 -10

Compound **5f**

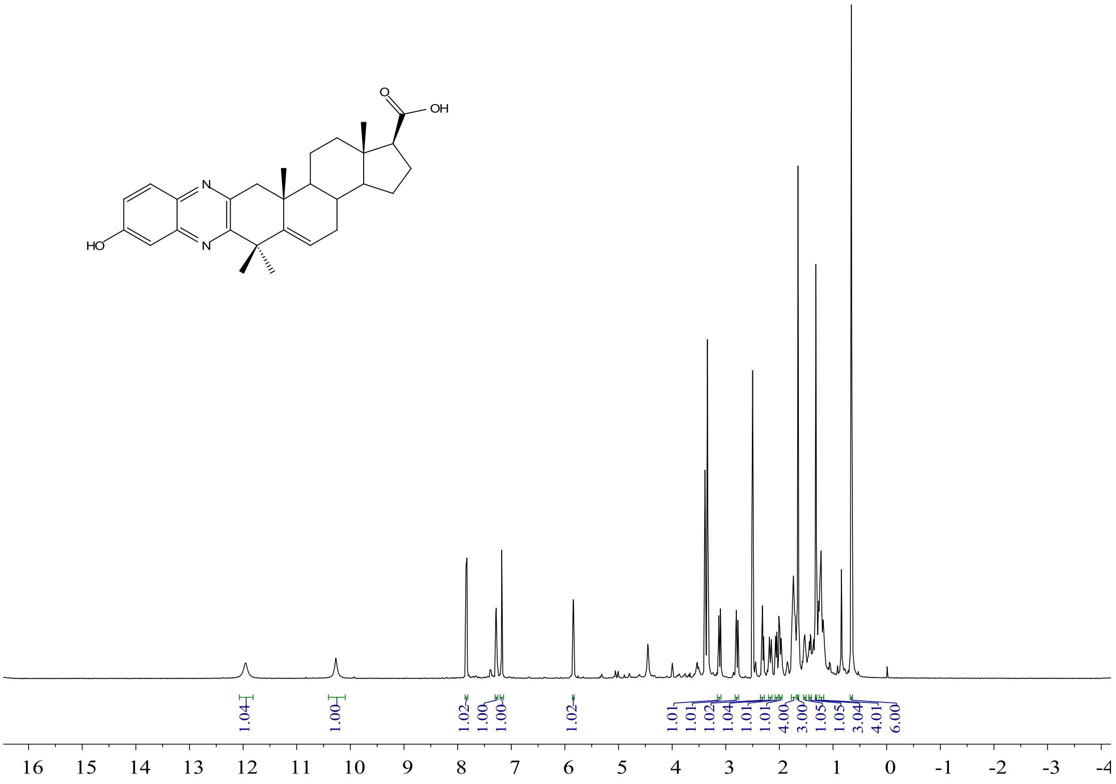
OH
O
N
N
HO
1.04
1.00
1.02
1.00
1.00
1.02
1.01
1.01
1.02
1.04
1.01
1.01
4.00
3.00
1.05
1.05
3.04
4.01
6.00
16 15 14 13 12 11 10 9 8 7 6 5 4 3 2 1 0 -1 -2 -3 -4

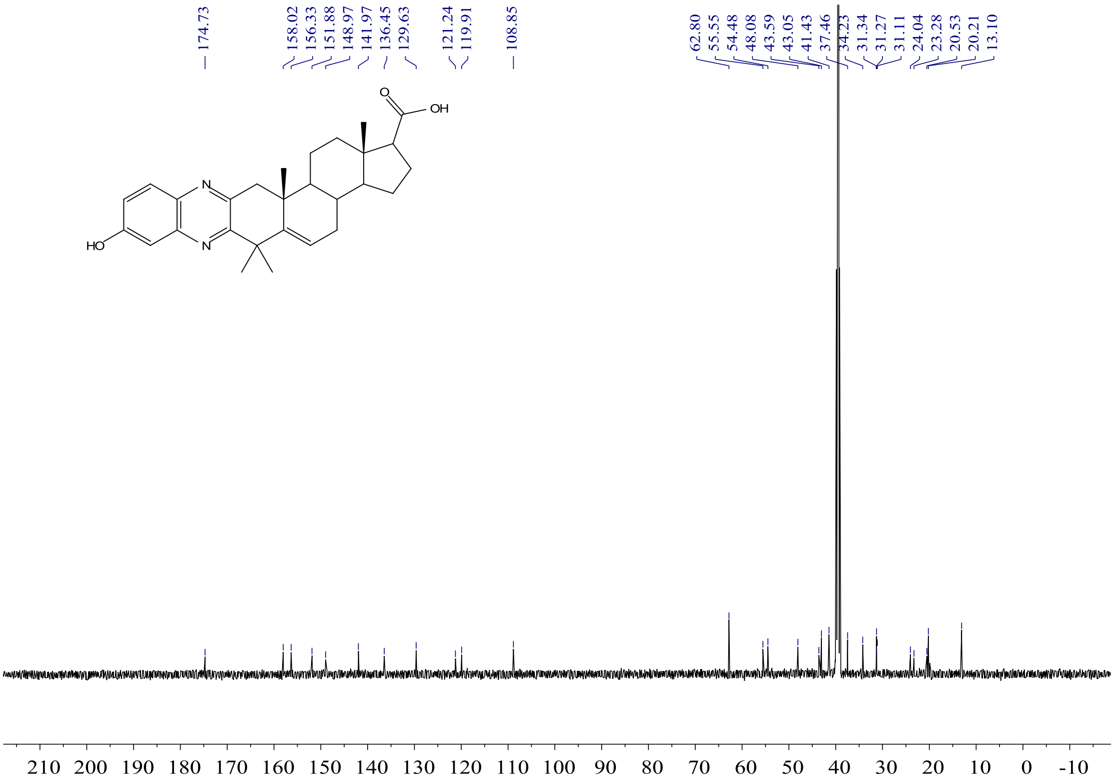
174.73
158.02
156.33
151.88
148.97
141.97
136.45
129.63
121.24
119.91
108.85
62.80
55.55
54.48
48.08
43.59
43.05
41.43
37.46
34.23
31.34
31.27
31.11
24.04
23.28
20.53
20.21
13.10
OH
O
N
N
HO
210 200 190 180 170 160 150 140 130 120 110 100 90 80 70 60 50 40 30 20 10 0 -10

3.3. The ^{1}H NMR and ^{13}C NMR Spectra of Compound **6-8**

Compound **6**

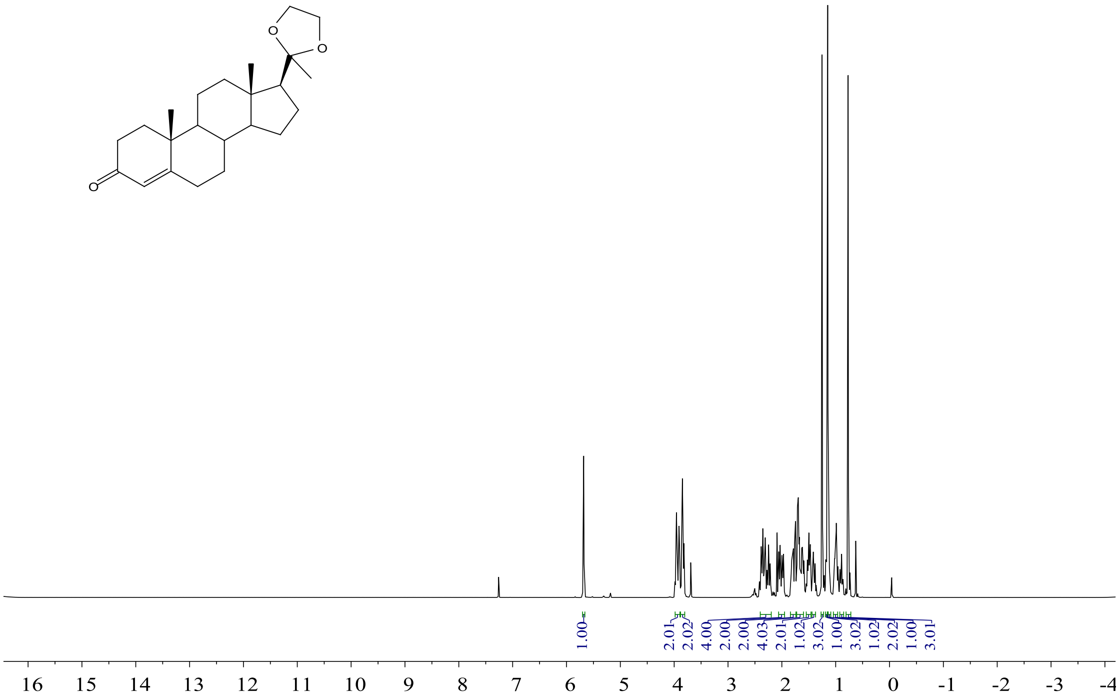

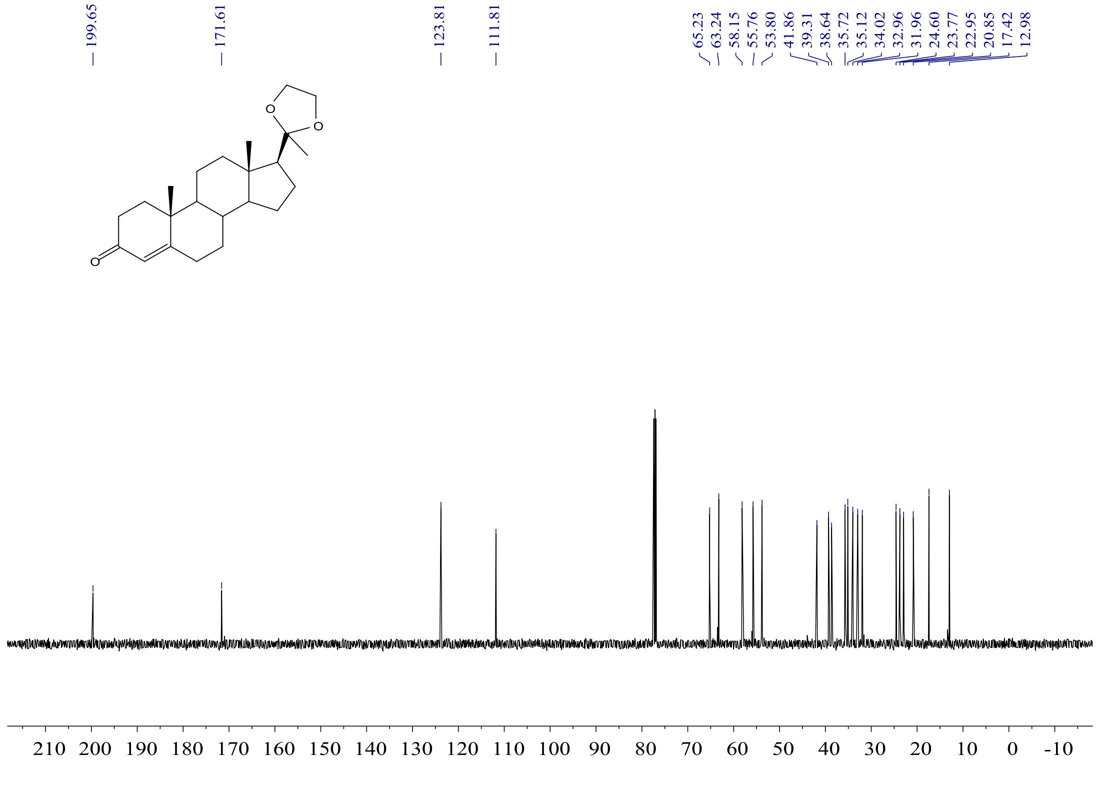

Compound **7**

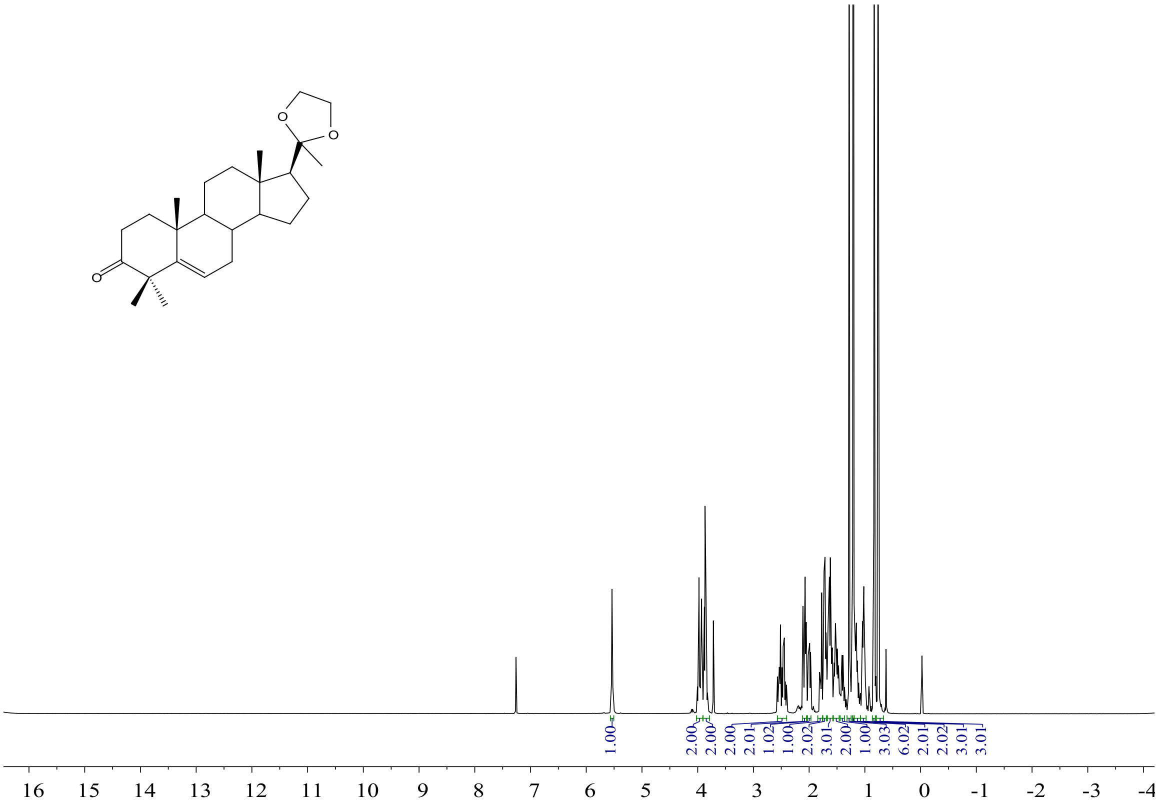
1.00
2.00
2.00
2.00
2.01
1.02
1.00
2.02
3.01
2.00
1.00
3.03
6.02
2.01
2.02
3.01
3.01
16 15 14 13 12 11 10 9 8 7 6 5 4 3 2 1 0 -1 -2 -3 -4

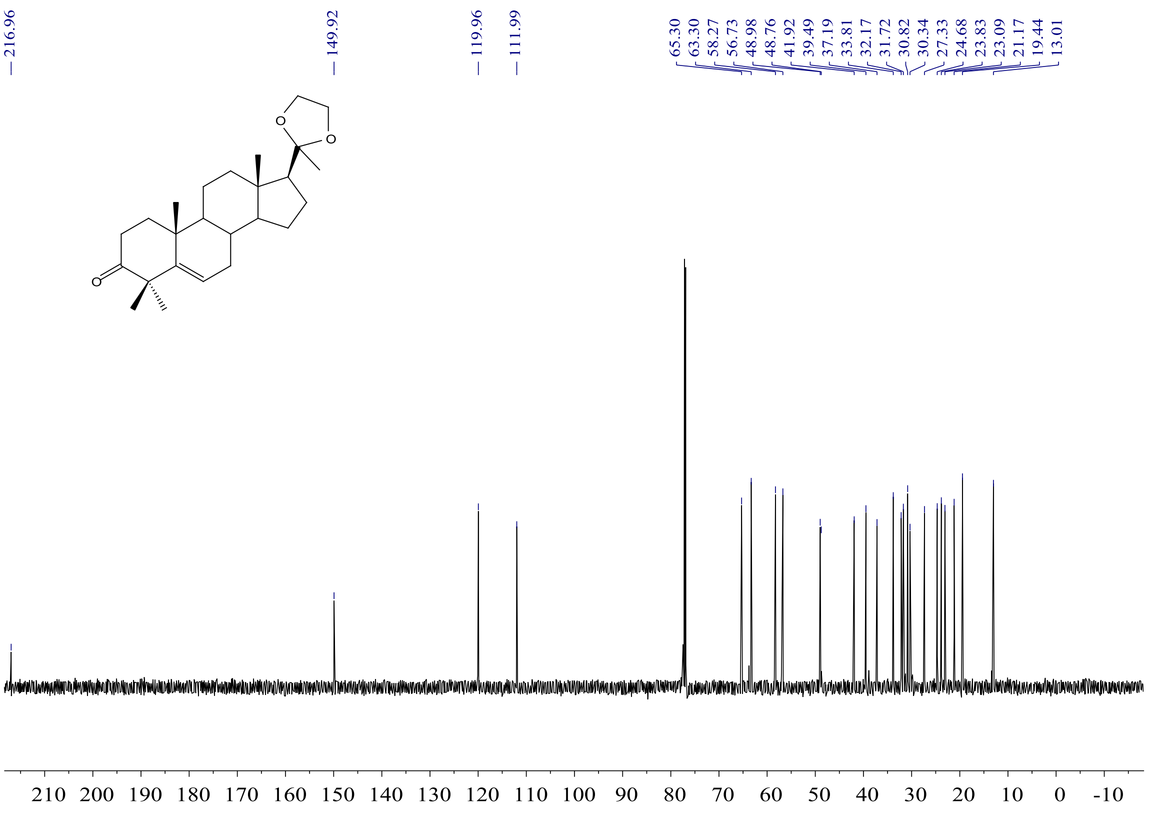
216.96
149.92
119.96
111.99
65.30
63.30
58.27
56.73
48.98
48.76
41.92
39.49
37.19
33.81
32.17
31.72
30.82
30.34
27.33
24.68
23.83
23.09
21.17
19.44
13.01
210 200 190 180 170 160 150 140 130 120 110 100 90 80 70 60 50 40 30 20 10 0 -10

Compound **8**

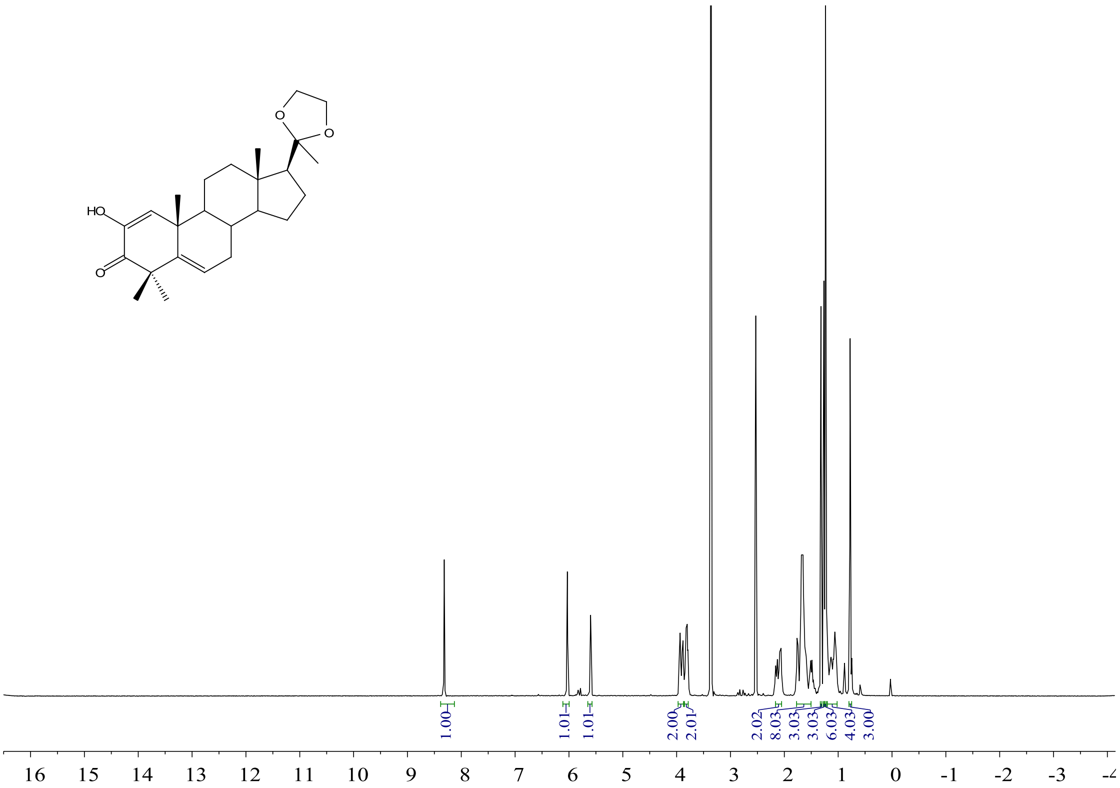
HO
O
O
O
1.00
1.01
1.01
2.00
2.01
2.02
8.03
3.03
3.03
6.03
4.03
3.00
16 15 14 13 12 11 10 9 8 7 6 5 4 3 2 1 0 -1 -2 -3

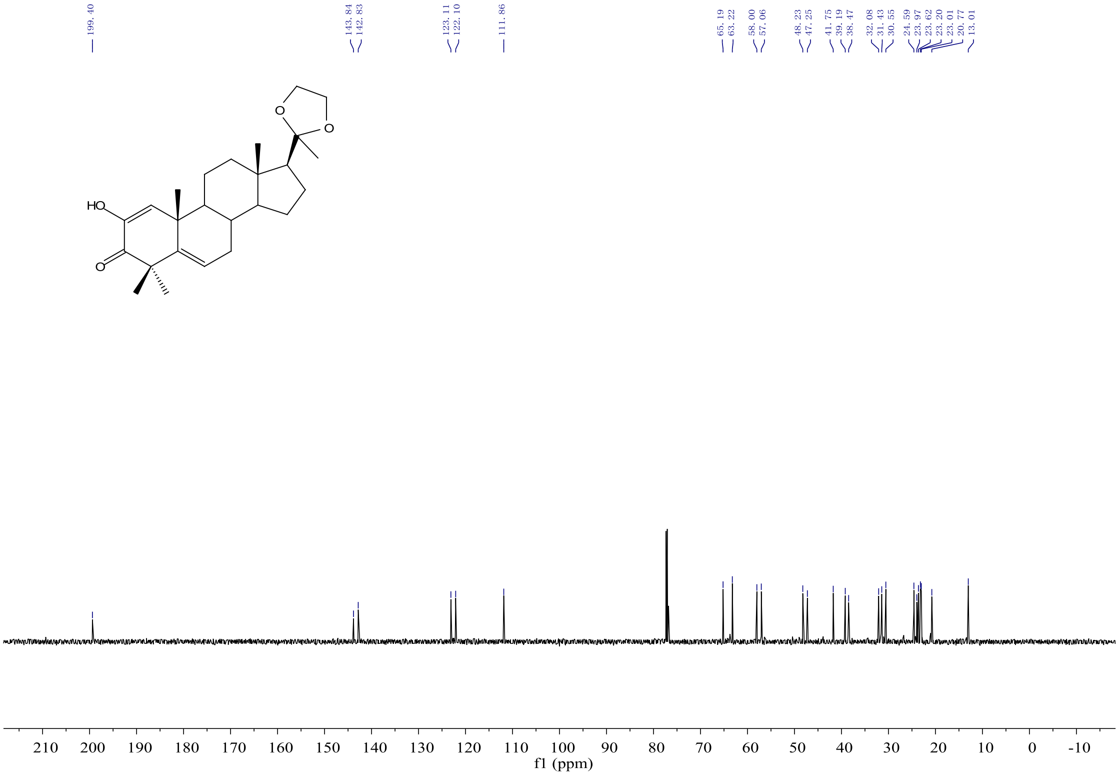
199.40
143.84
142.83
123.11
122.10
111.86
65.19
63.22
58.00
57.06
48.23
47.25
41.75
39.19
38.47
32.08
31.43
30.55
24.59
23.97
23.62
23.20
23.01
20.77
13.01
HO
O
O
O
210 200 190 180 170 160 150 140 130 120 110 100 90 80 70 60 50 40 30 20 10 0 -10
f1 (ppm)

3.4.** The ^{1}H and ^{13}C NMR Spectra of Compound **9a-9o** and the ^{1}H NMR Spectra of Compound **10a-10o

Compound **9a and 10a**

Because both of th**e** ^{1}H NMR and ^{13}C NMR of target compound **10a** have been obtained, so the NMR spectra of **9a**, as the precursor of **10a,** haven't been necessary any more.

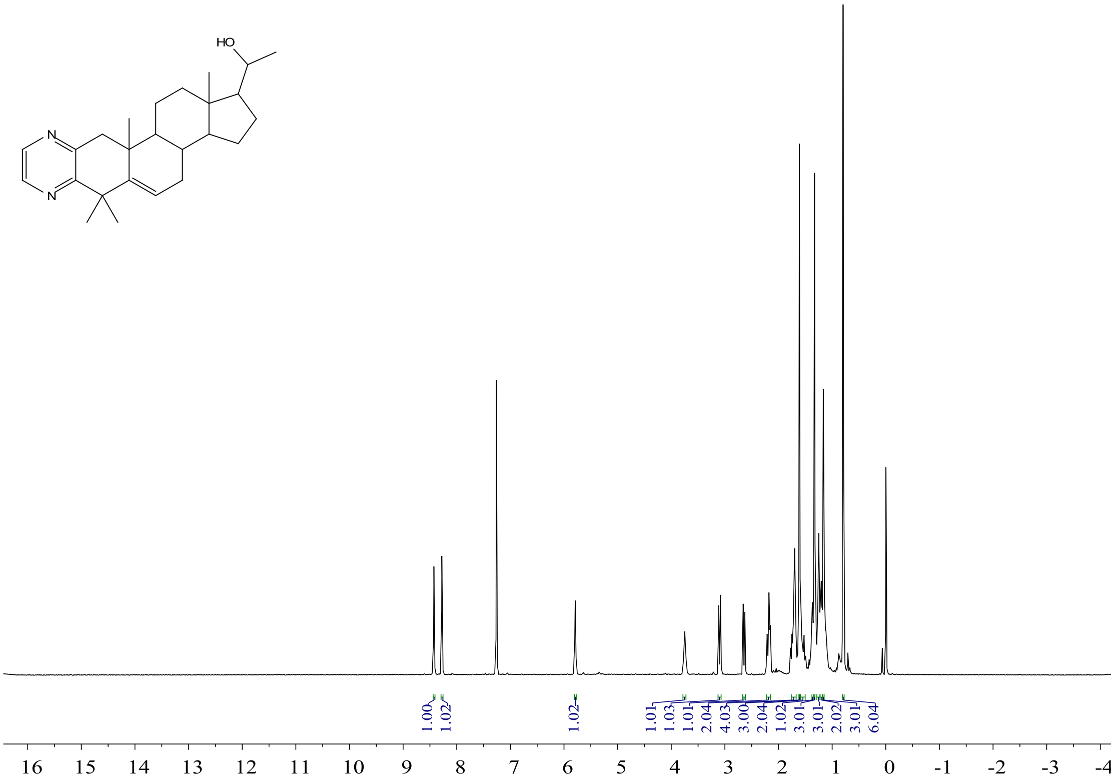

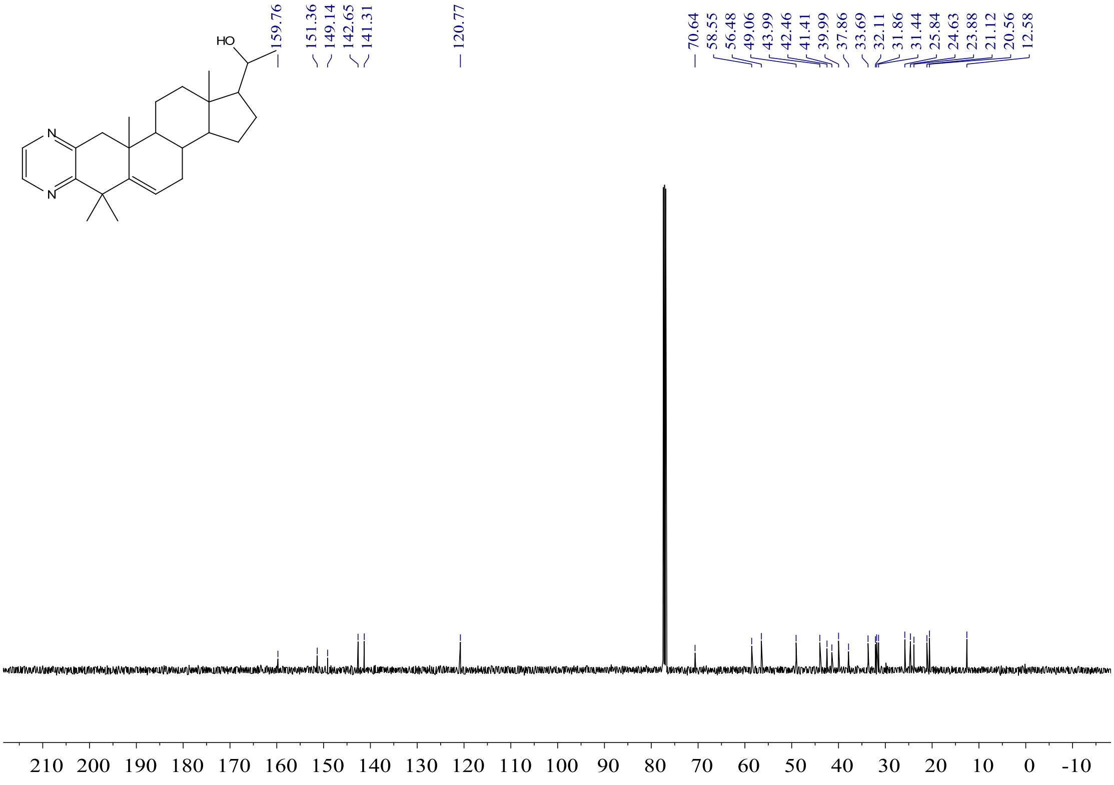

Compound **9b** and **10b**

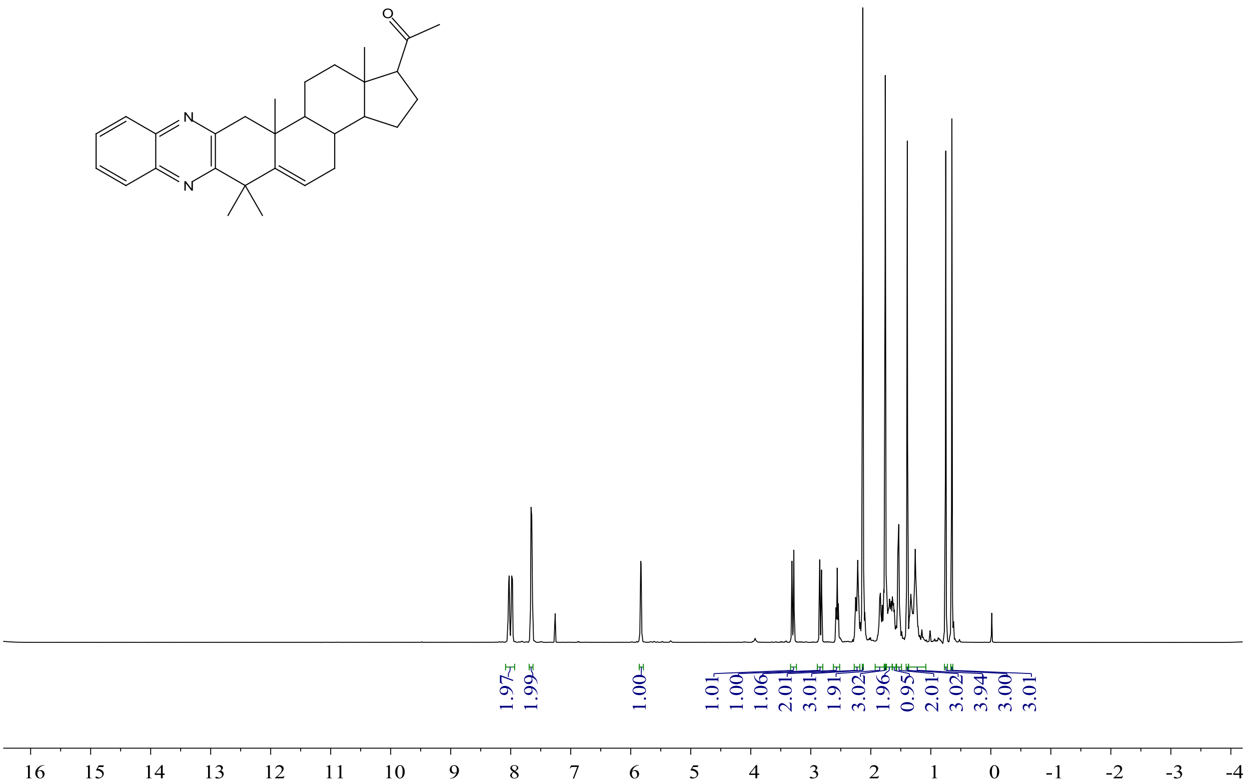

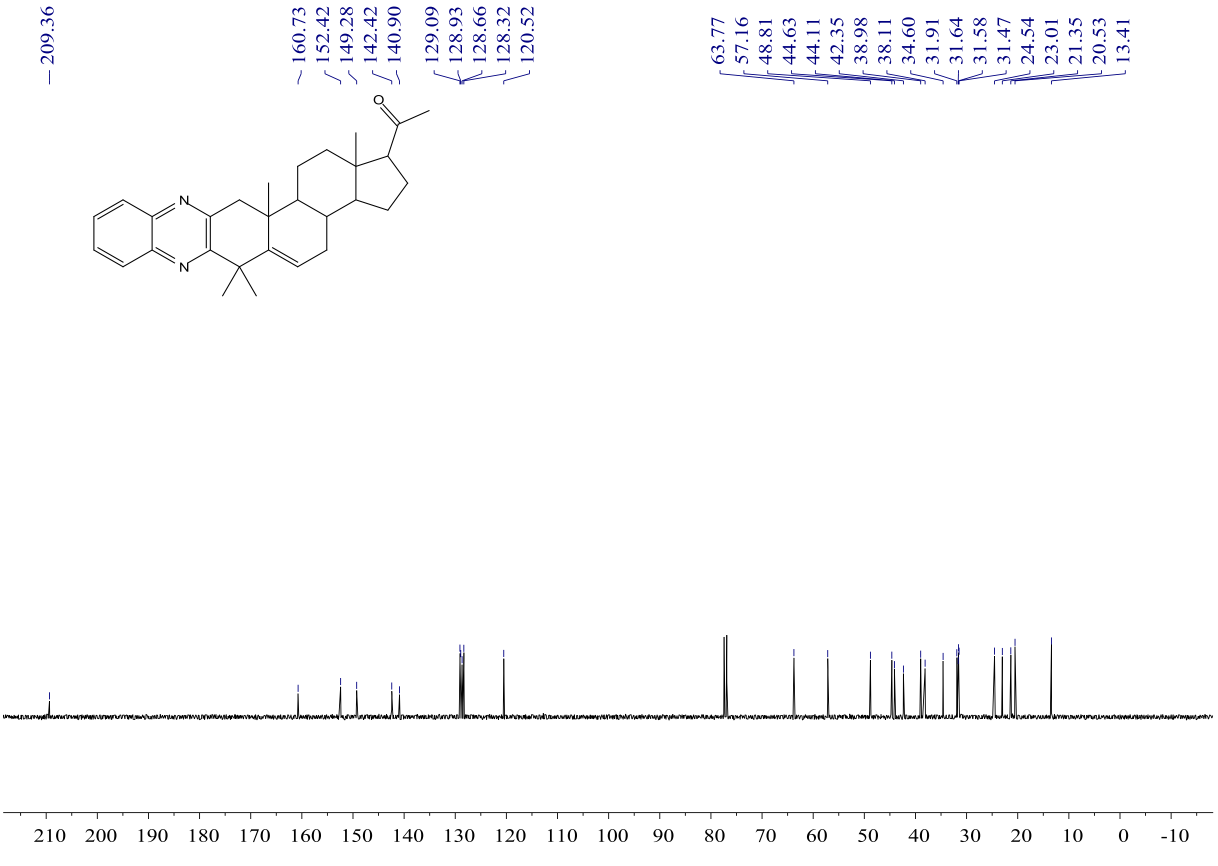

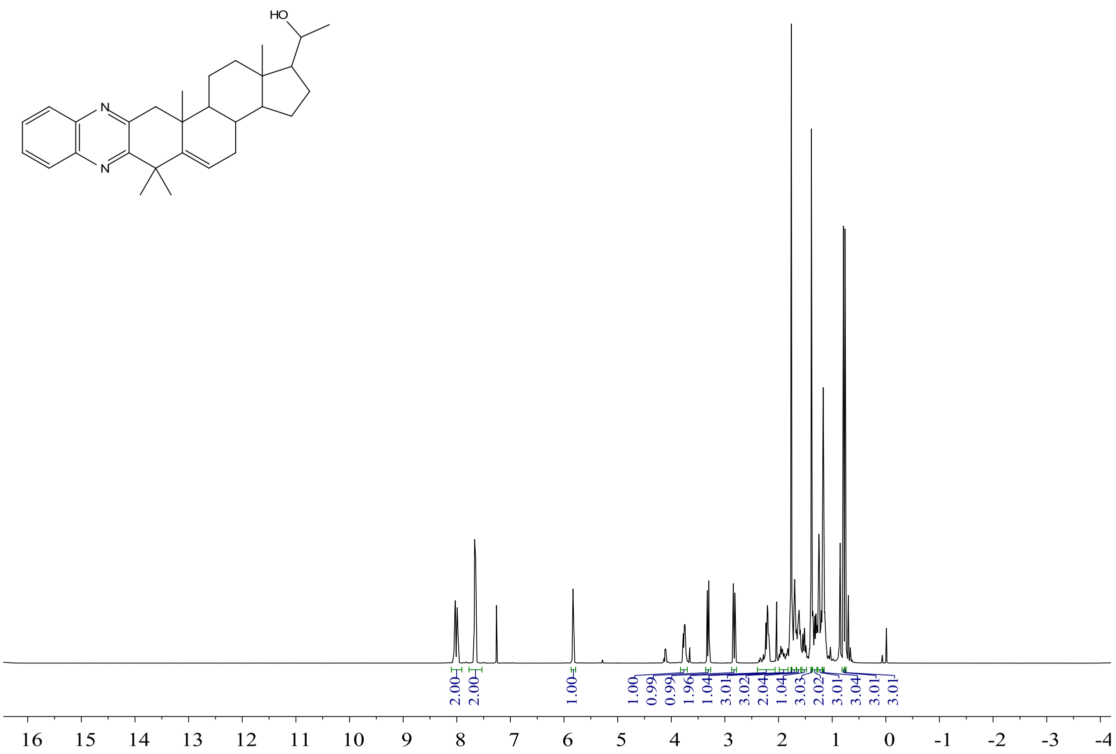

Compound **9c** and **10c**

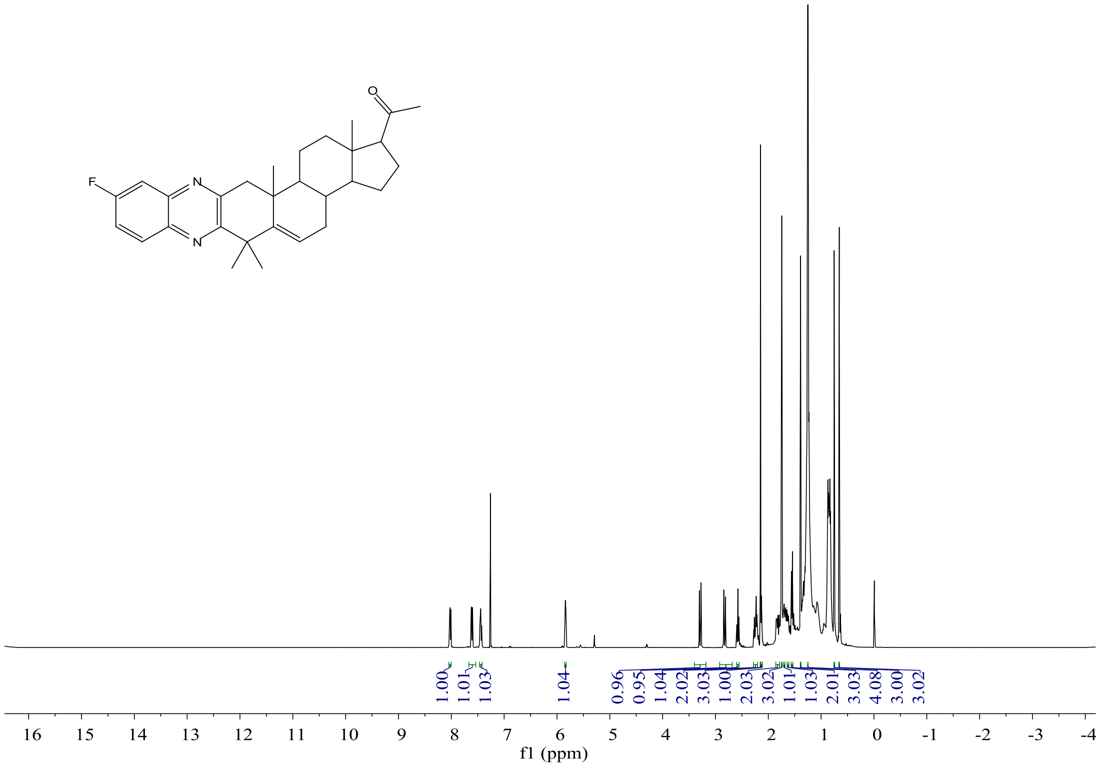

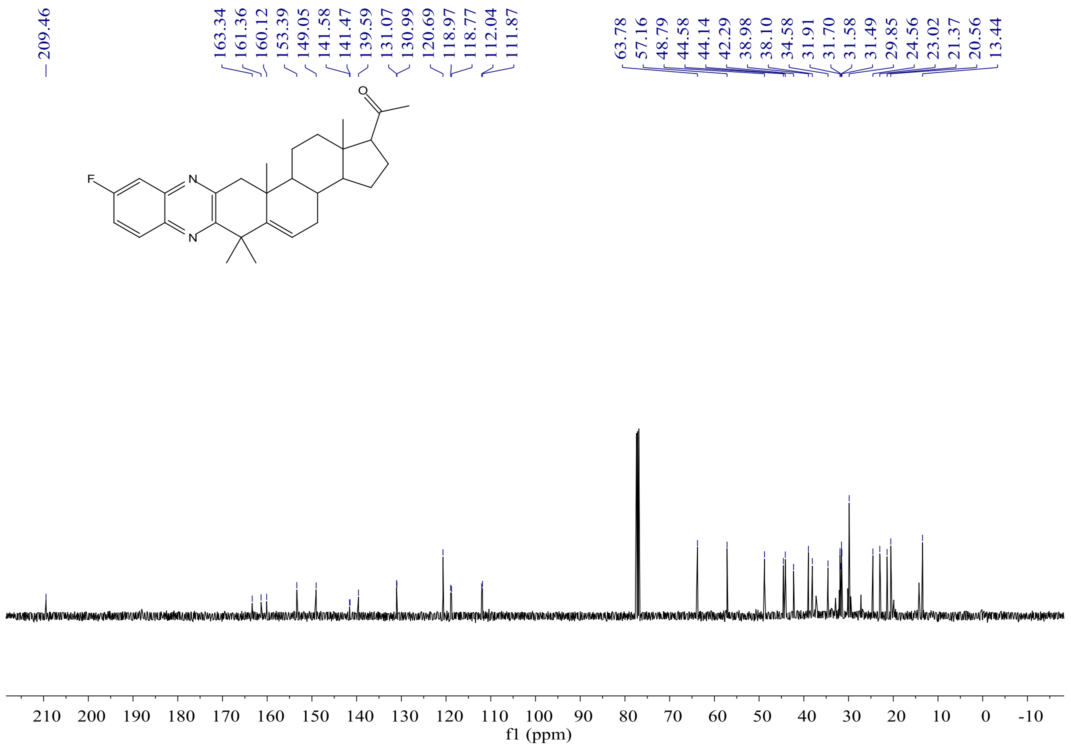
209.46
163.34
161.36
160.12
153.39
149.05
141.58
141.47
139.59
131.07
130.99
120.69
118.97
118.77
112.04
111.87
63.78
57.16
48.79
44.58
44.14
42.29
38.98
38.10
34.58
31.91
31.70
31.58
31.49
29.85
24.56
23.02
21.37
20.56
13.44
F
N
N
O
f1 (ppm)

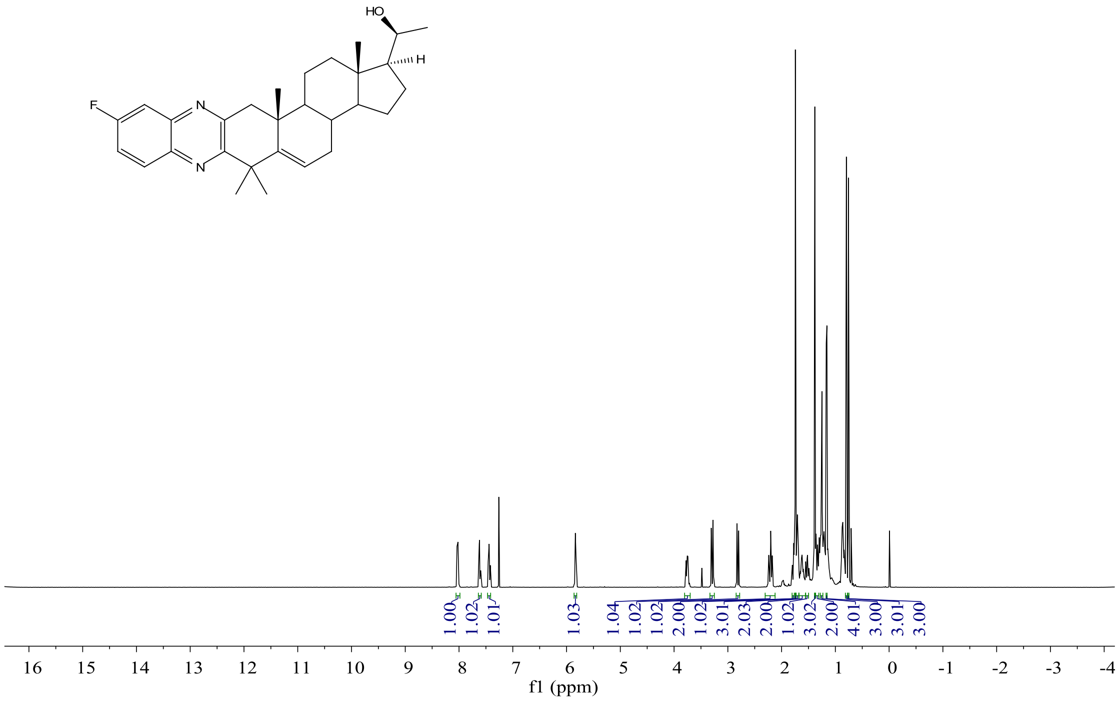
HO
H
F
N
N
1.00
1.02
1.01
1.03
1.04
1.02
1.02
2.00
1.02
3.01
2.03
2.00
1.02
3.02
2.00
4.01
3.00
3.01
3.00
f1 (ppm)

Compound **9d** and **10d**

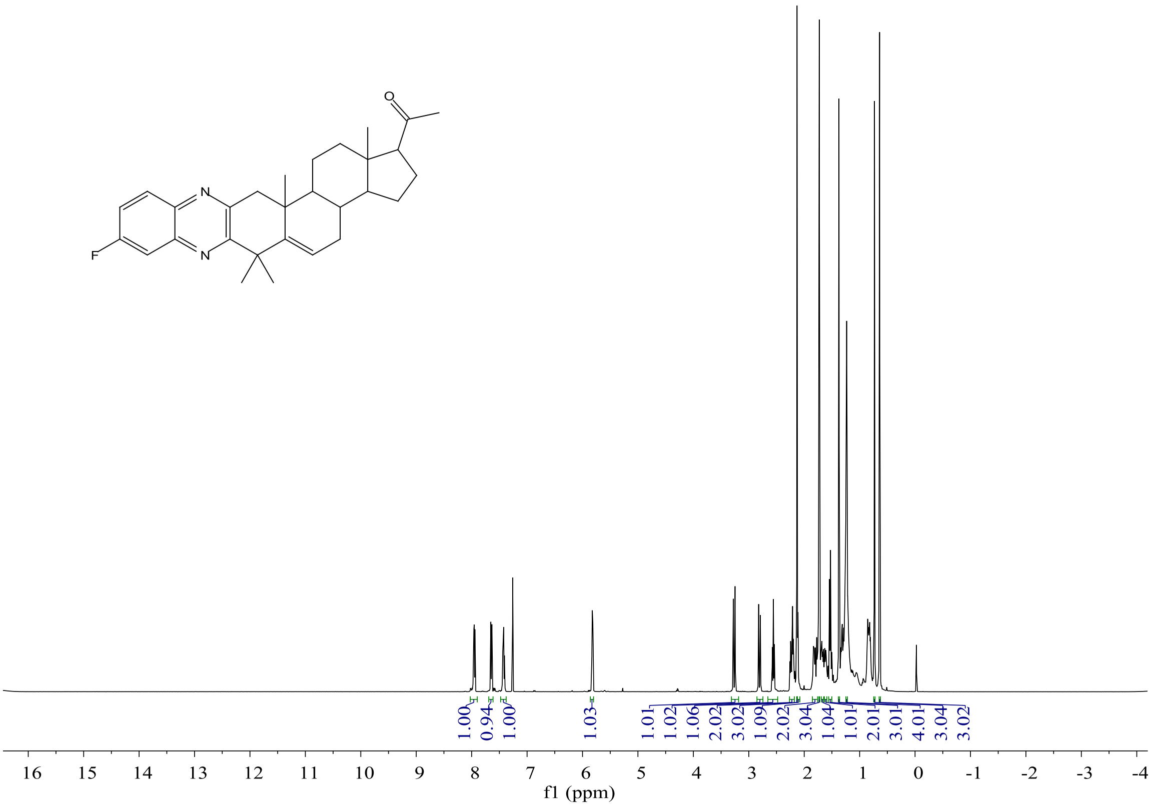

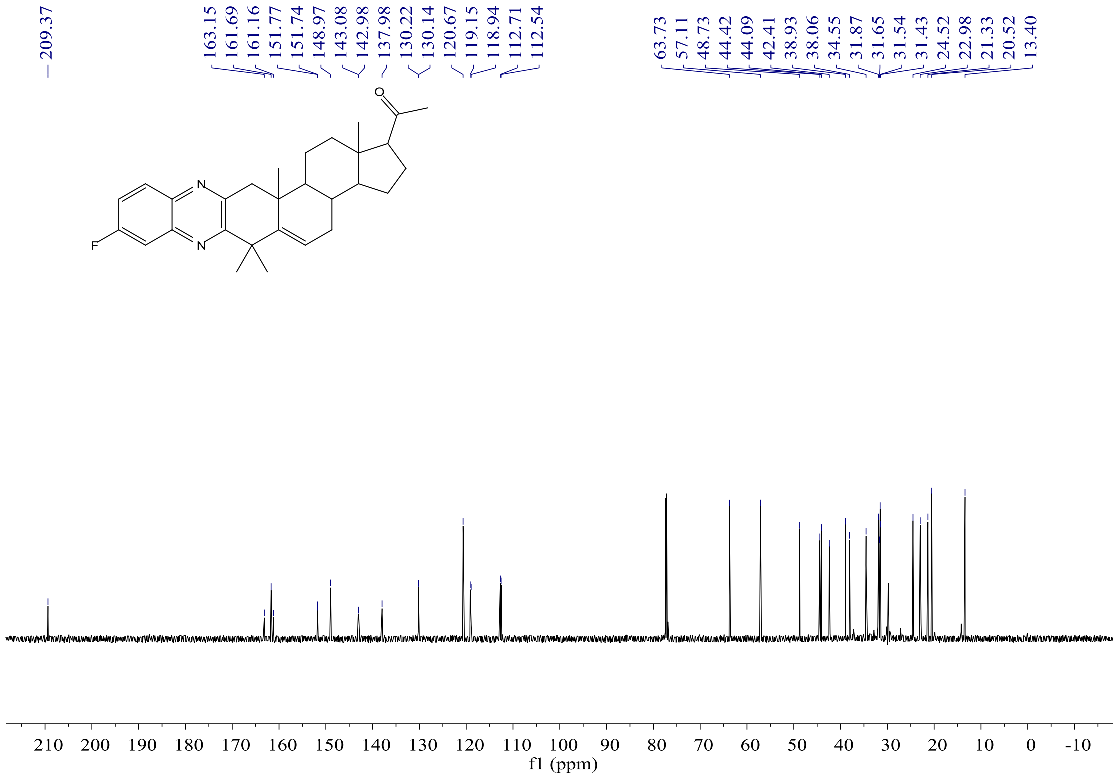

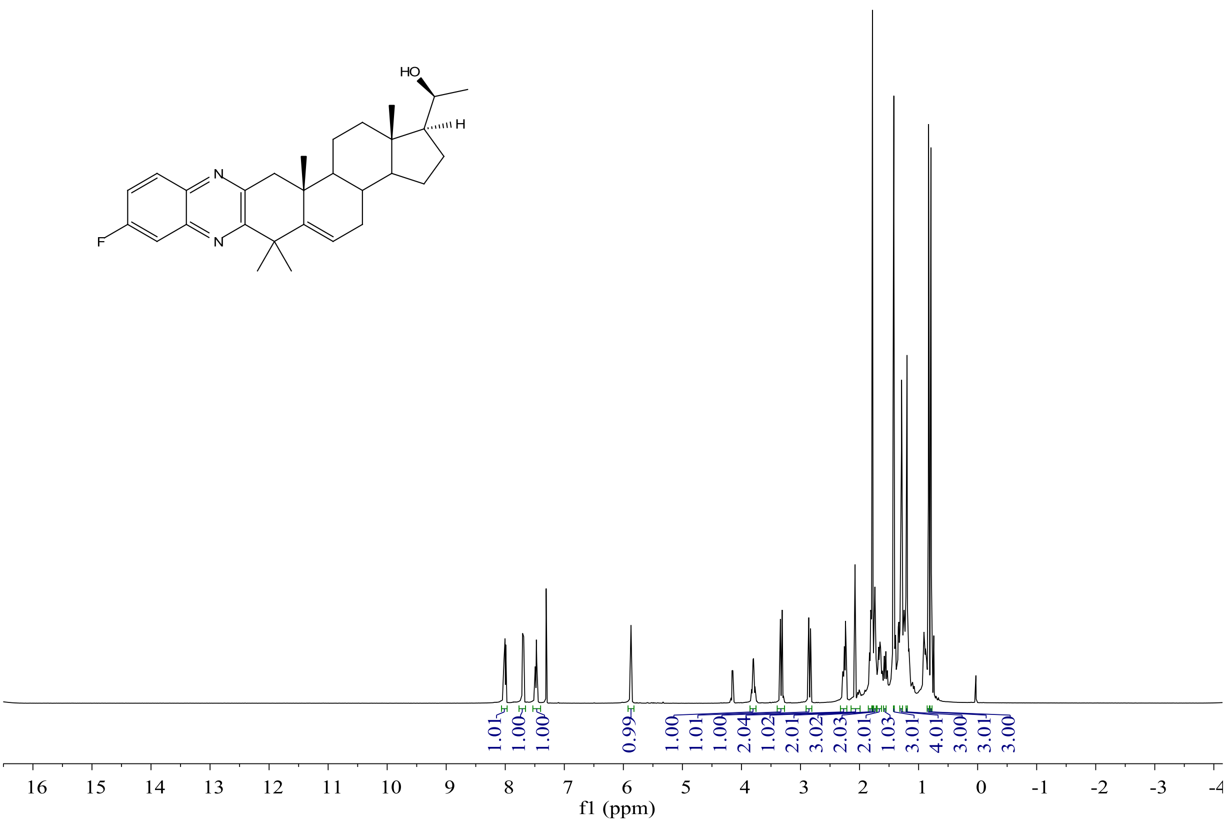

Compound **9e** and **10e**

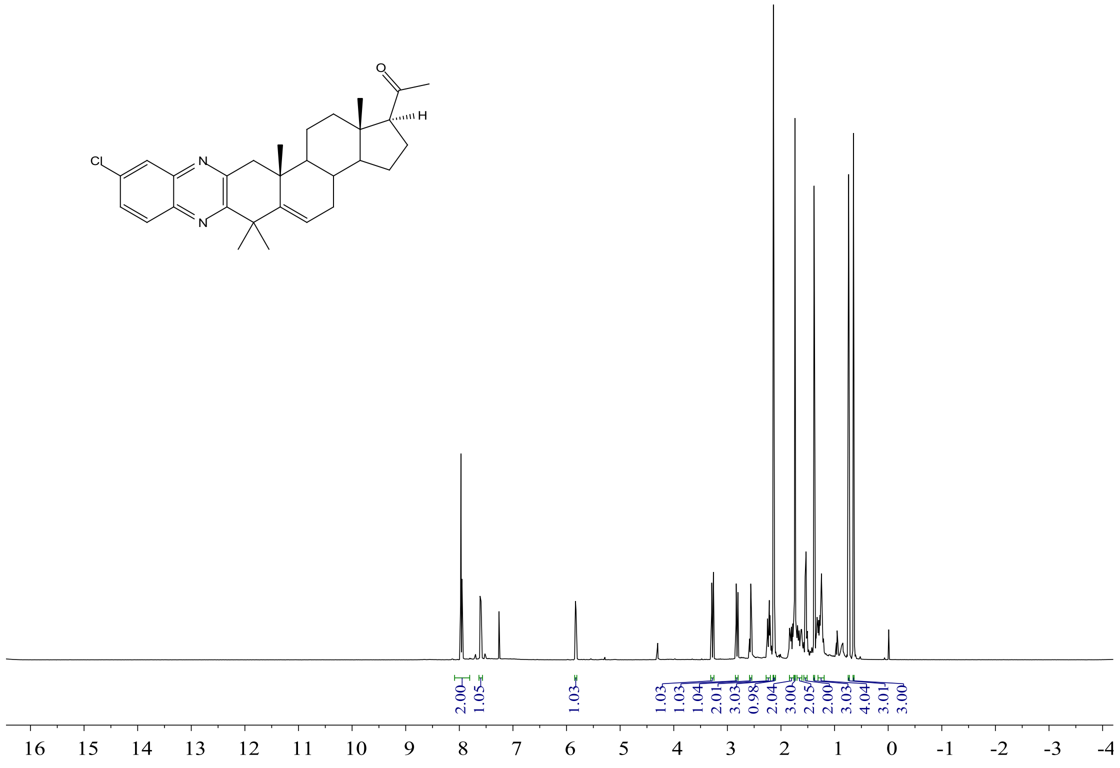

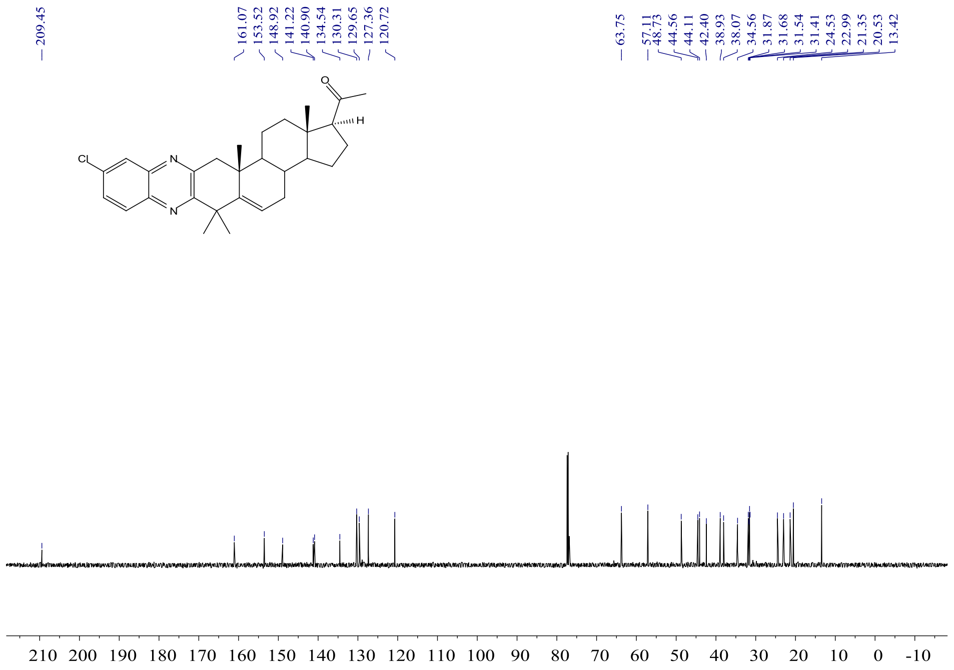
209.45
161.07
153.52
148.92
141.22
140.90
134.54
130.31
129.65
127.36
120.72
63.75
57.11
48.73
44.56
44.11
42.40
38.93
38.07
34.56
31.87
31.68
31.54
31.41
24.53
22.99
21.35
20.53
13.42
Cl
N
N
O
H
210 200 190 180 170 160 150 140 130 120 110 100 90 80 70 60 50 40 30 20 10 0 -10

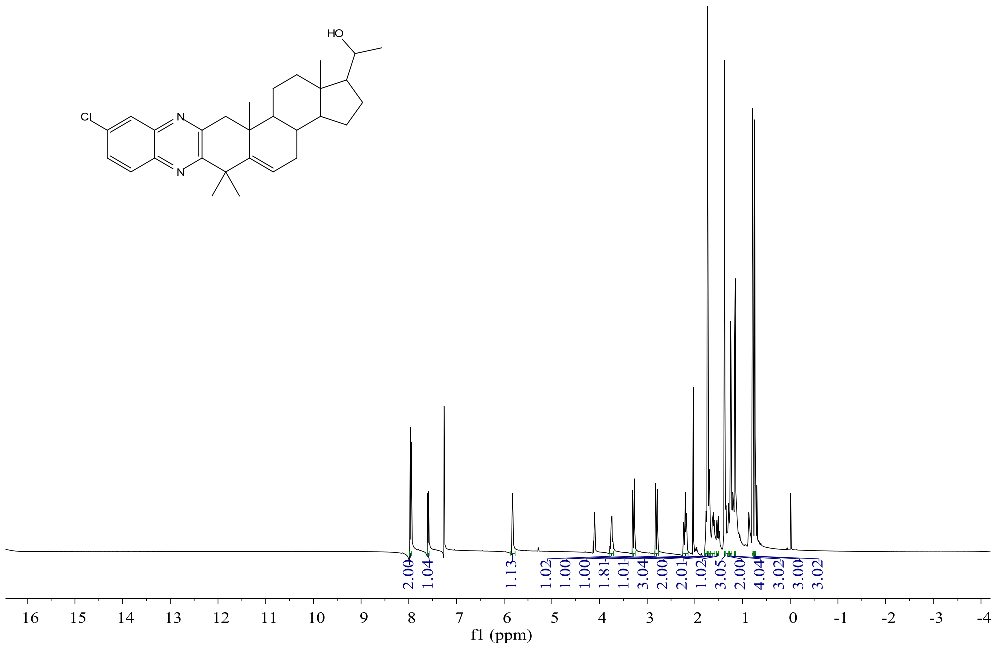
HO
Cl
N
N
2.00
1.04
1.13
1.02
1.00
1.00
1.81
1.01
3.04
2.00
2.01
1.02
3.05
2.00
4.04
3.02
3.00
3.02
16 15 14 13 12 11 10 9 8 7 6 5 4 3 2 1 0 -1 -2 -3 -4
f1 (ppm)

Compound **9f** and **10f**

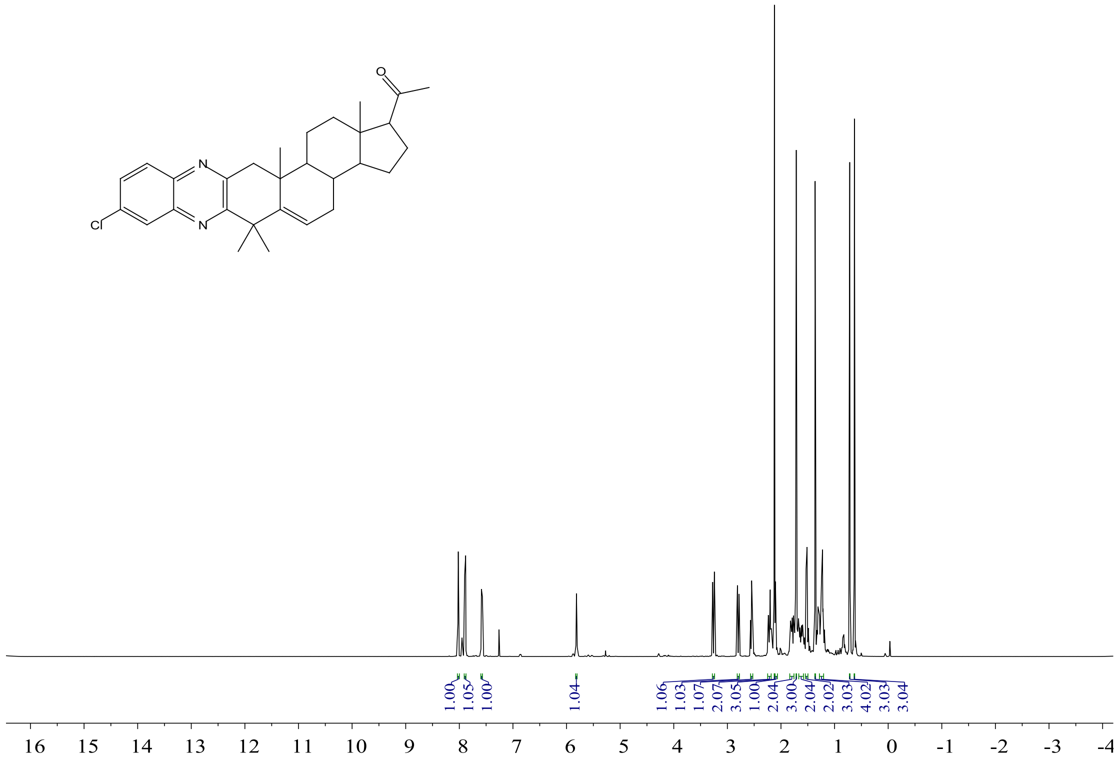

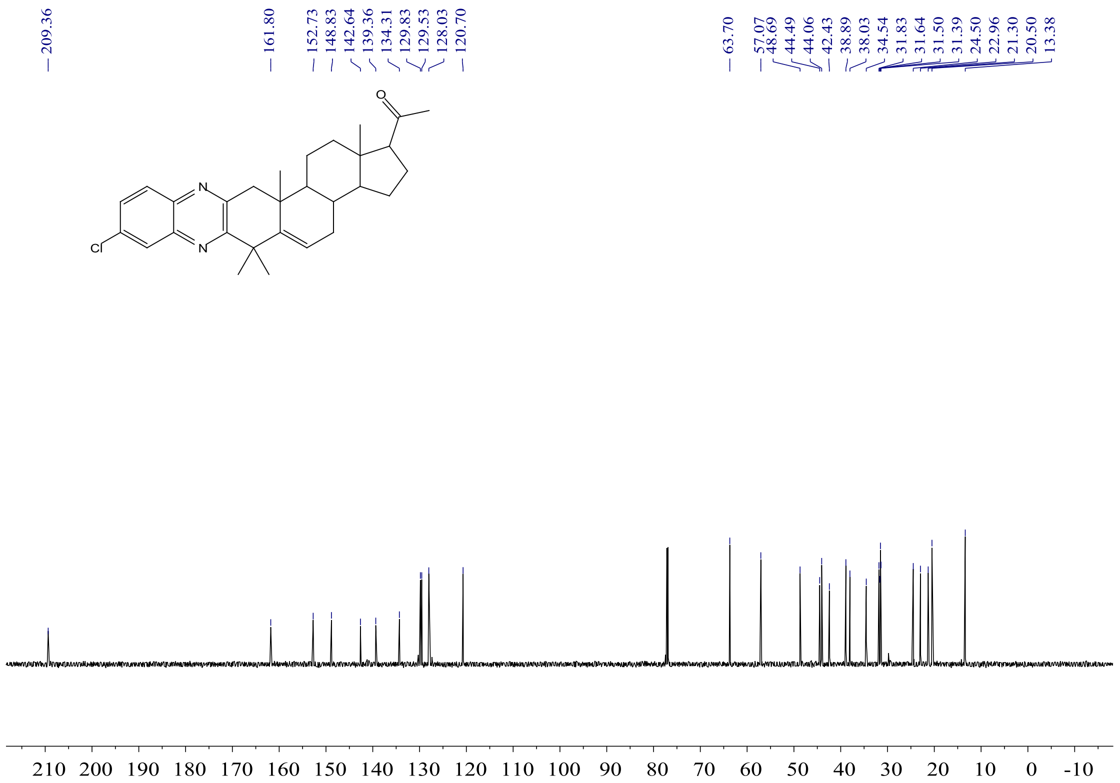

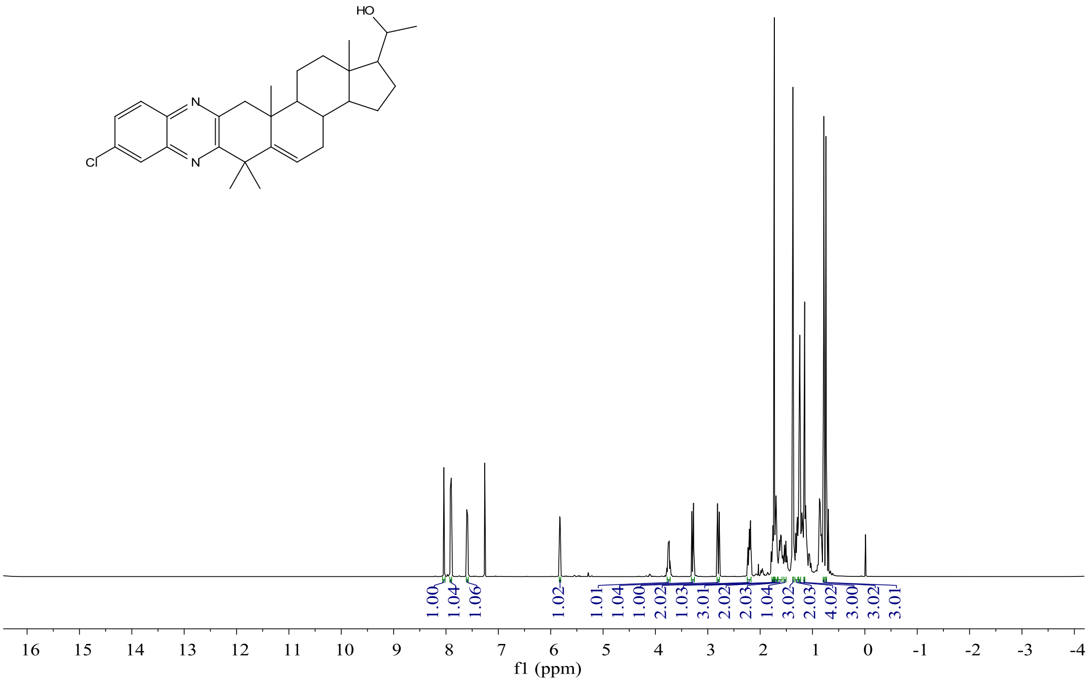

Compound **9g** and **10g**

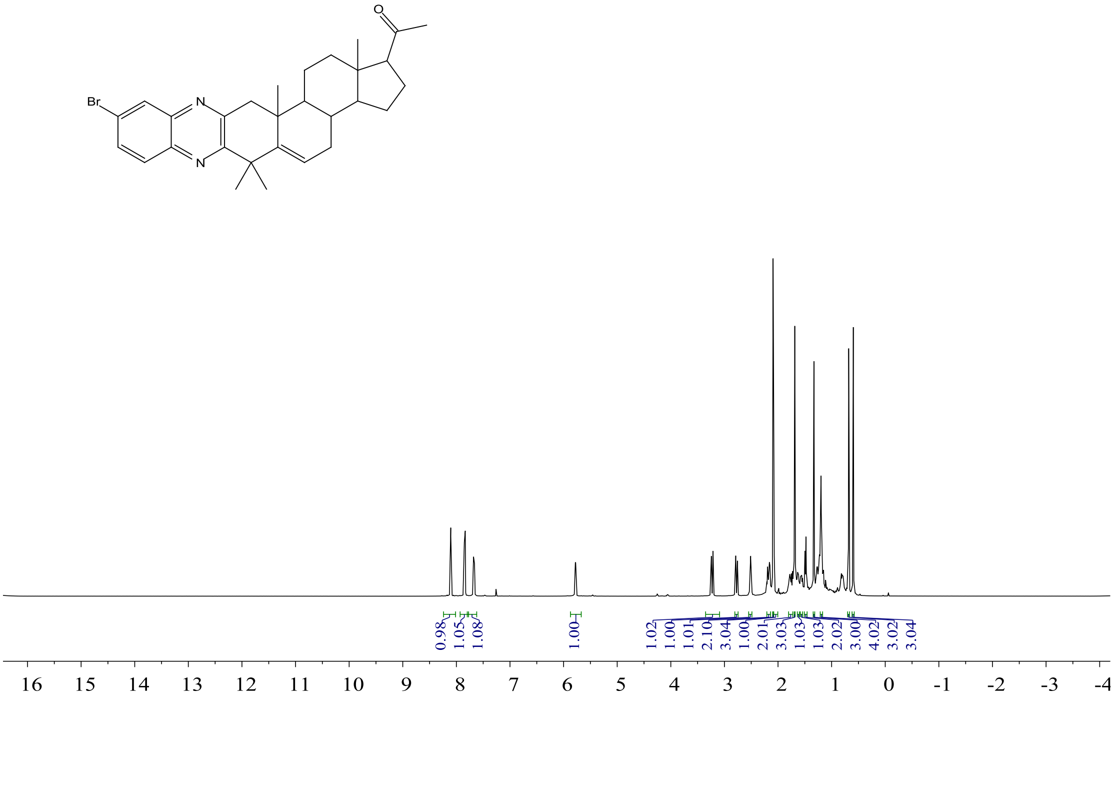

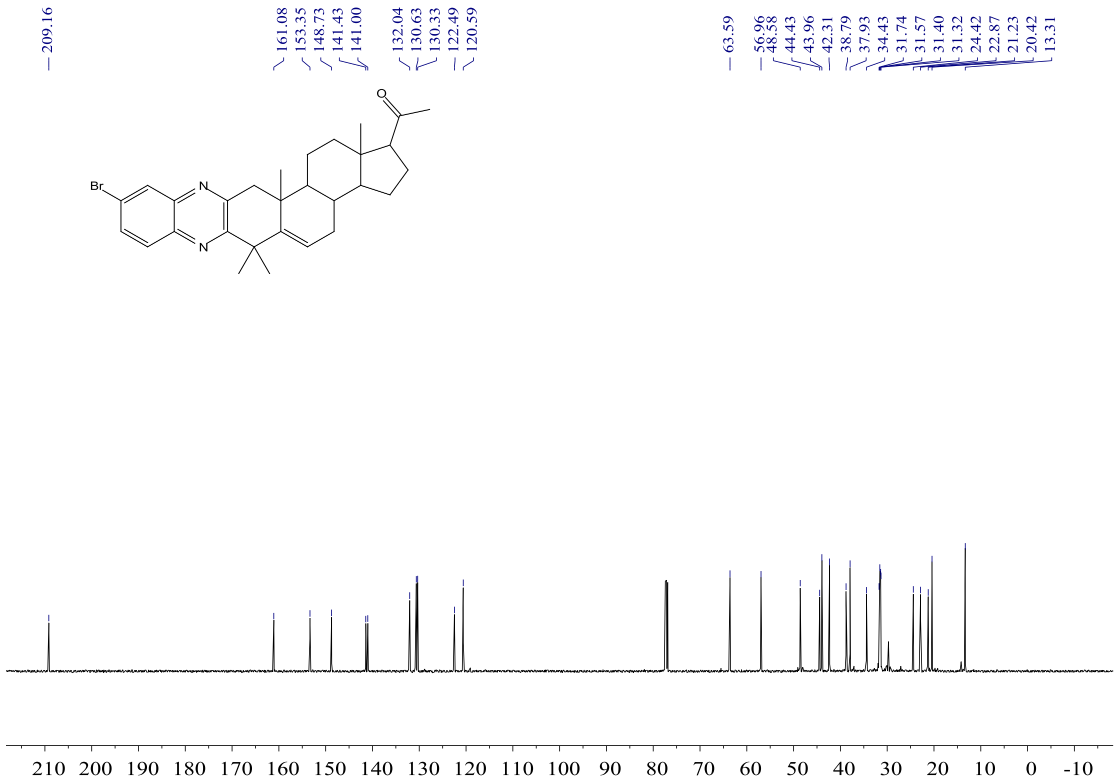

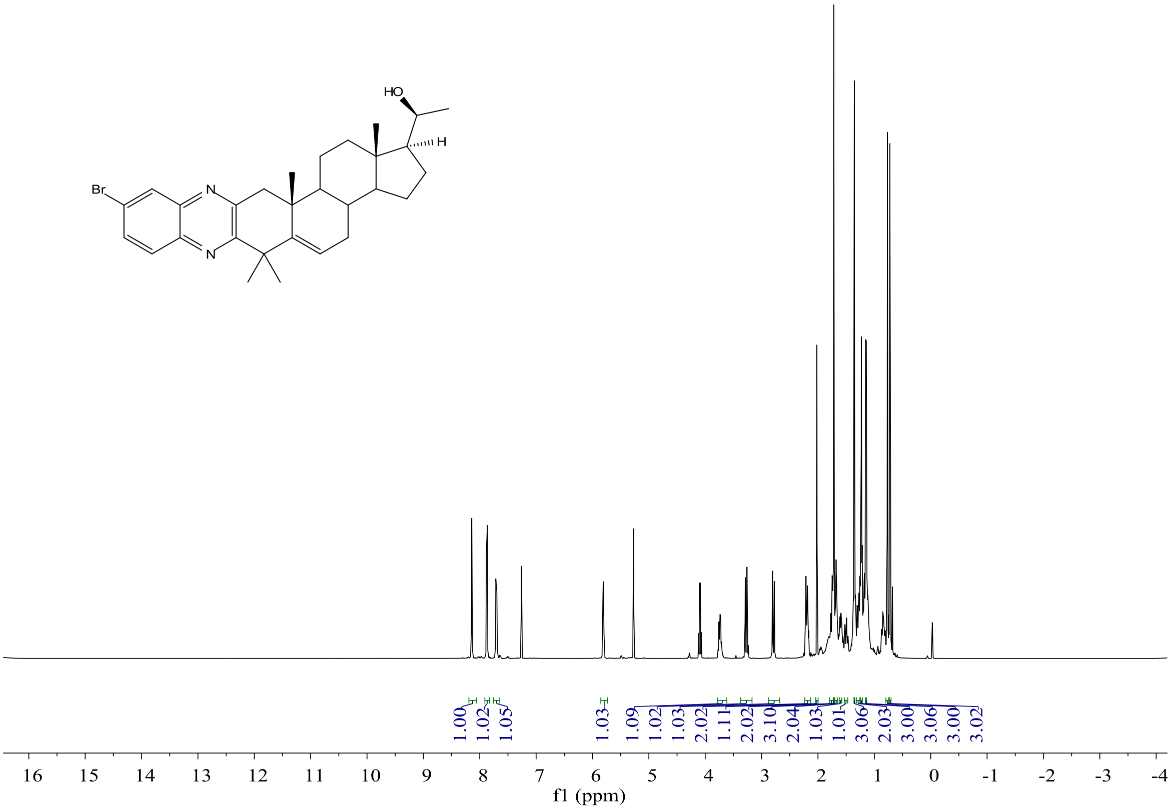

Compound **9h** and **10h**

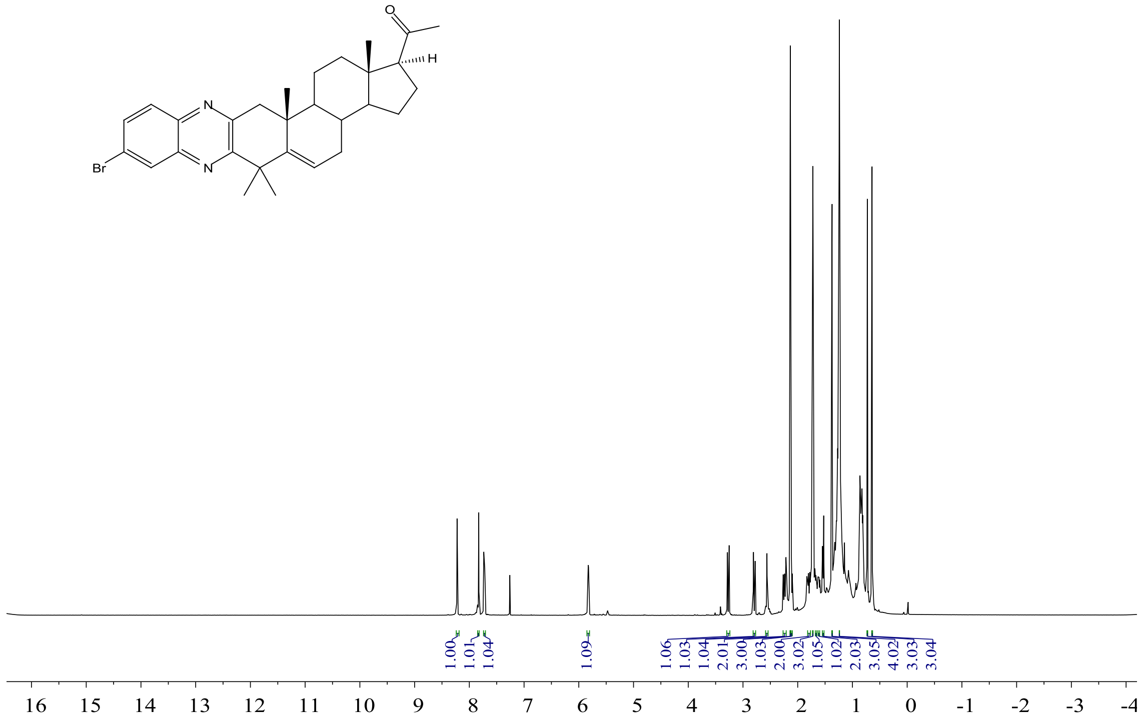

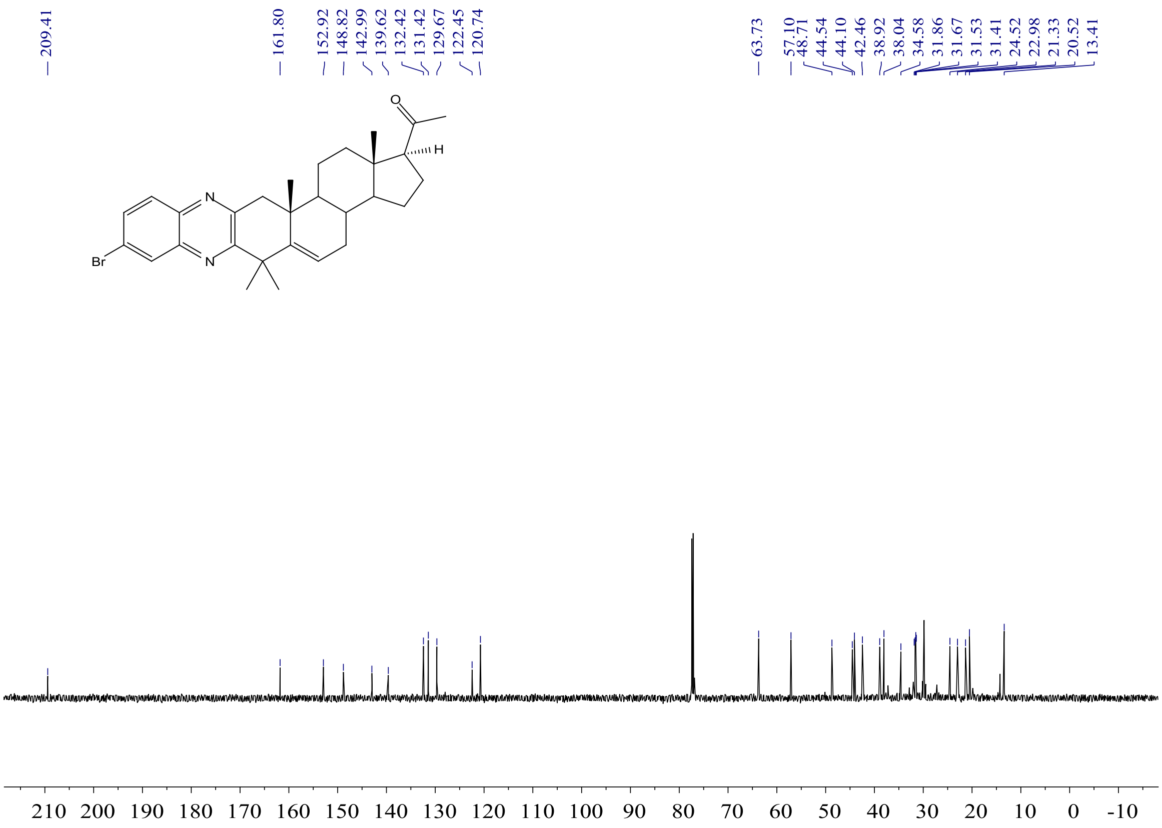

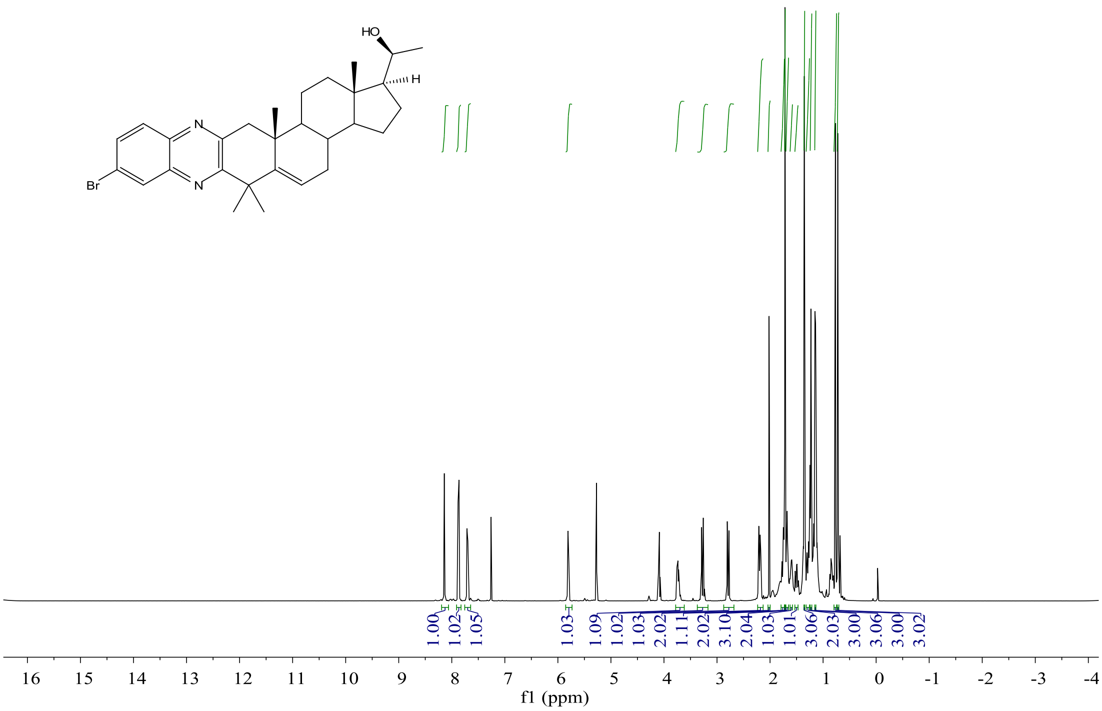

Compound **9i** and **10i**

Because both of the ^{1}H NMR and ^{13}C NMR of target compound **10i** have been obtained, so the NMR spectra of **9i**, as the precursor of **10i,** haven't been necessary any more.

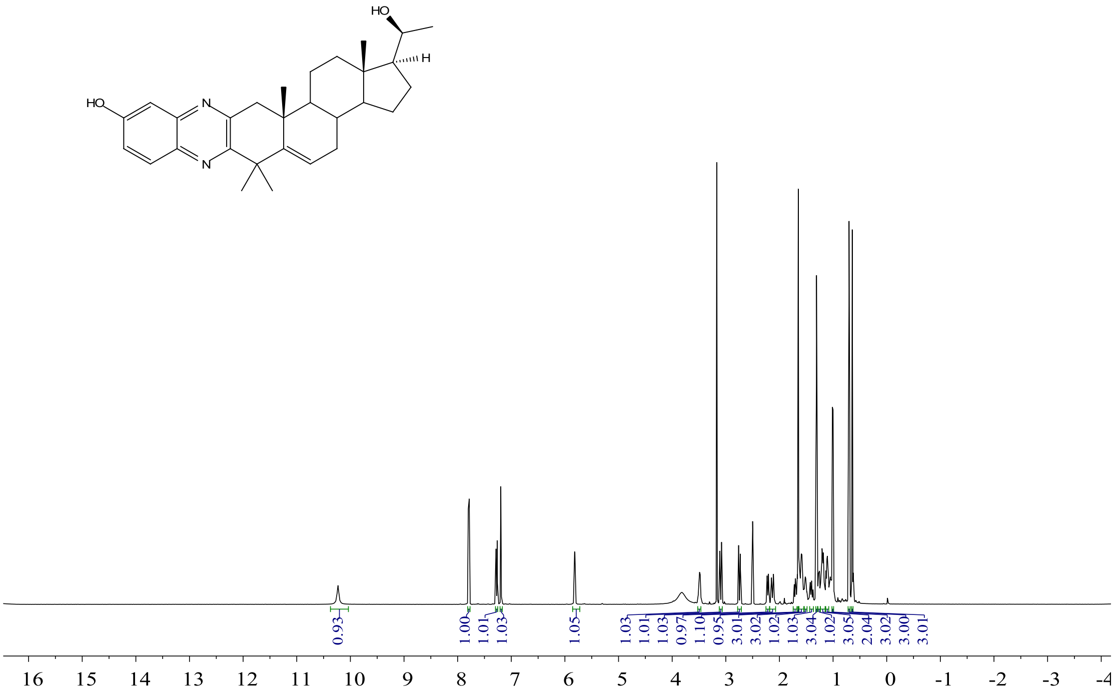

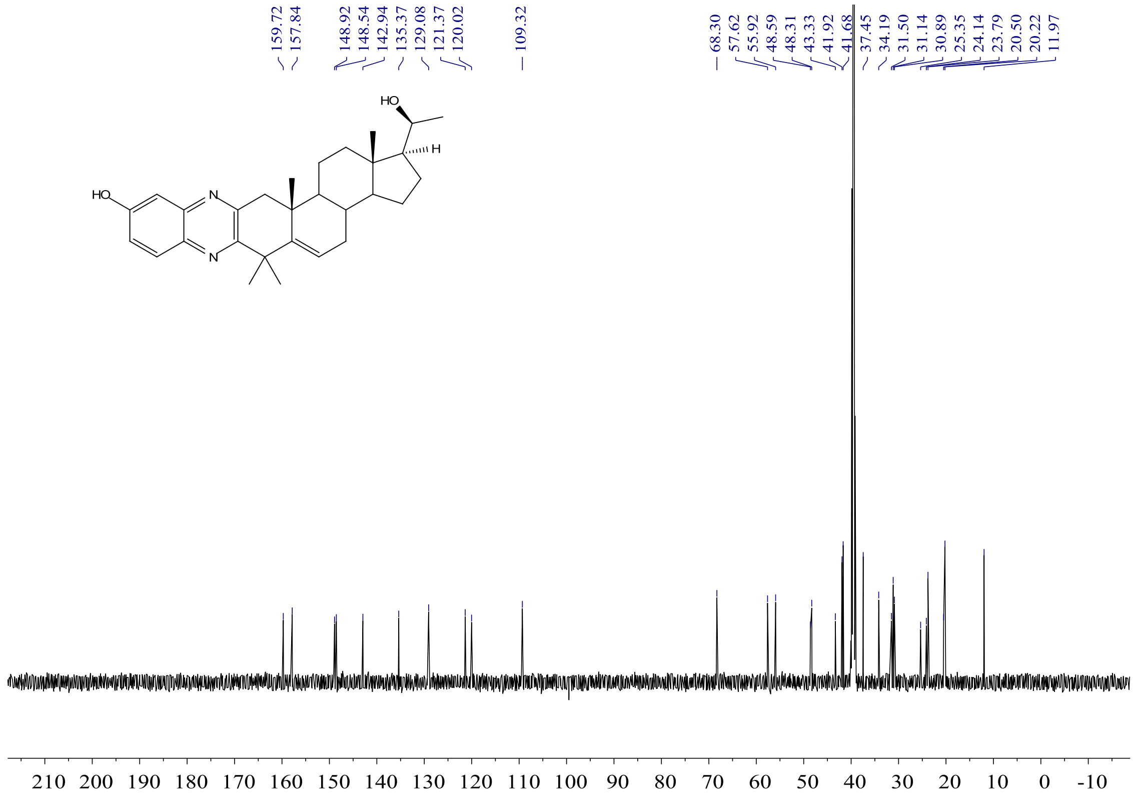

Compound **9j** and **10j**

Because both of the ^{1}H NMR and ^{13}C NMR of target compound **10i** have been obtained, so the NMR spectra of **9i**, as the precursor of **10i,** haven't been necessary any more.

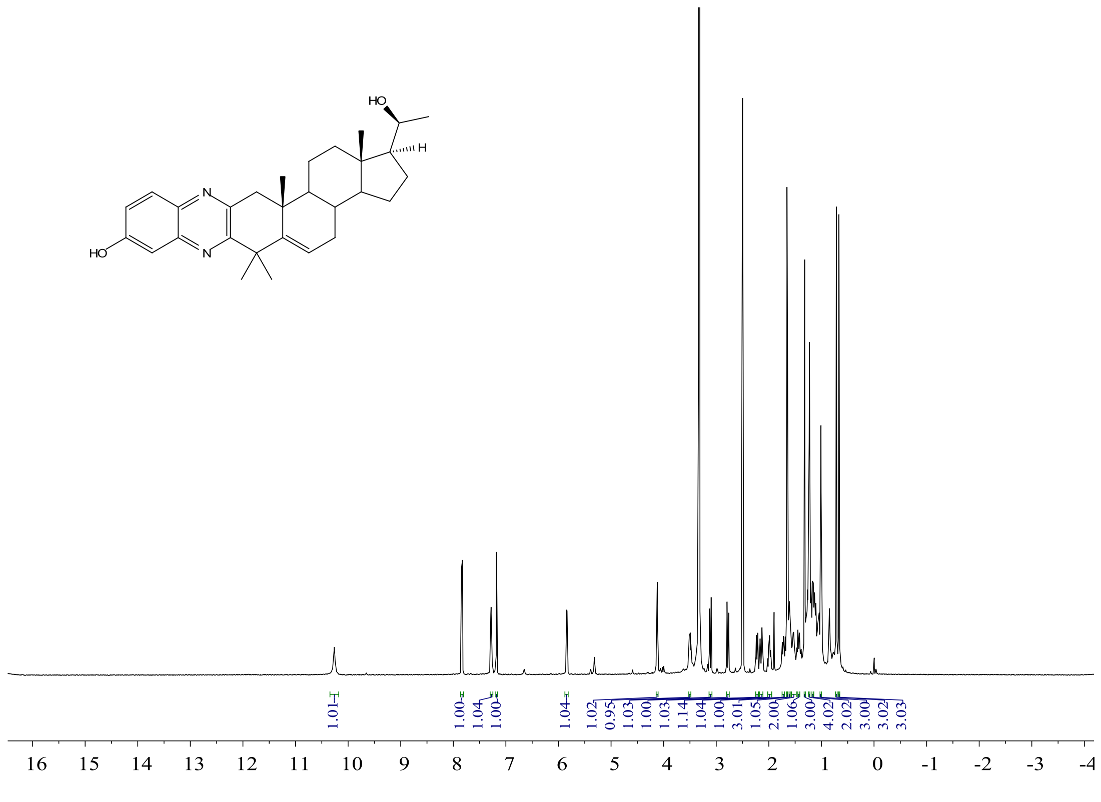

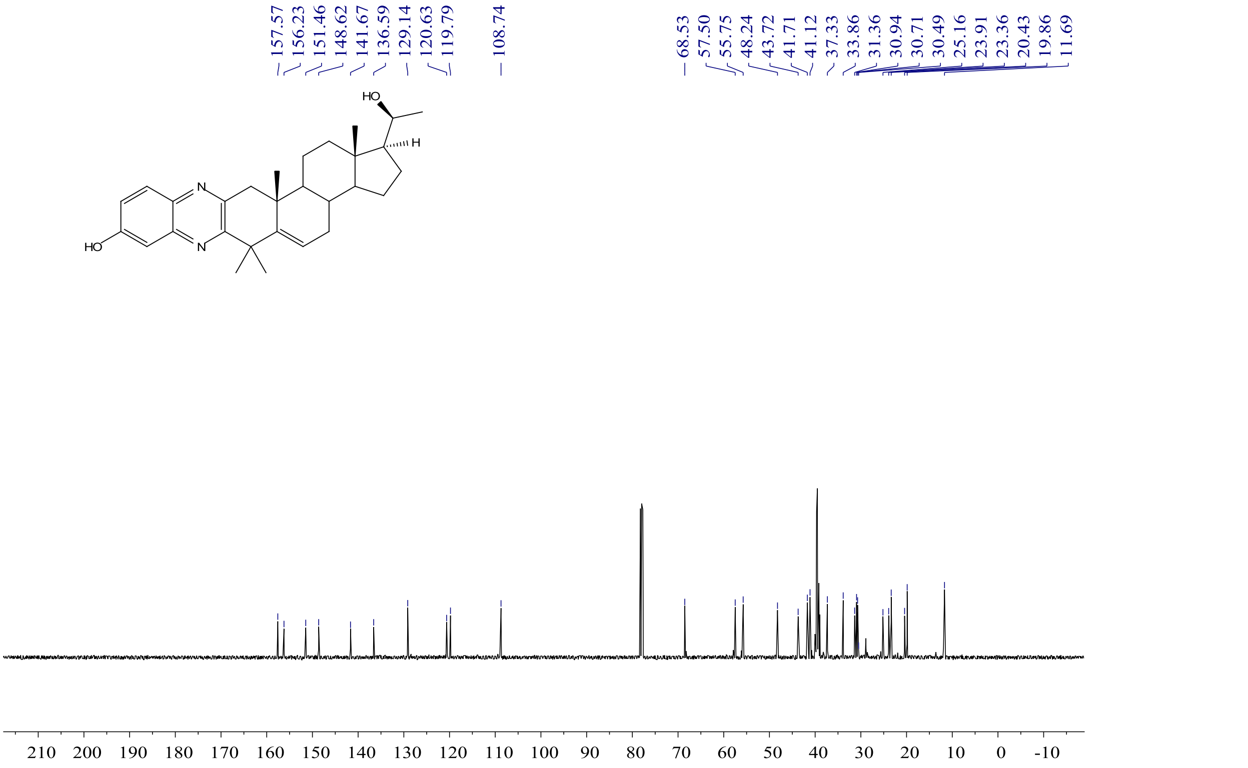

*The first group of inhibitors of type **6** with novel oxepine and azepine B-ring structures belongs to the NCME-series*
The second series of bioactive compounds are congeners of allocolchicine (

Compound **9k** and **10k**

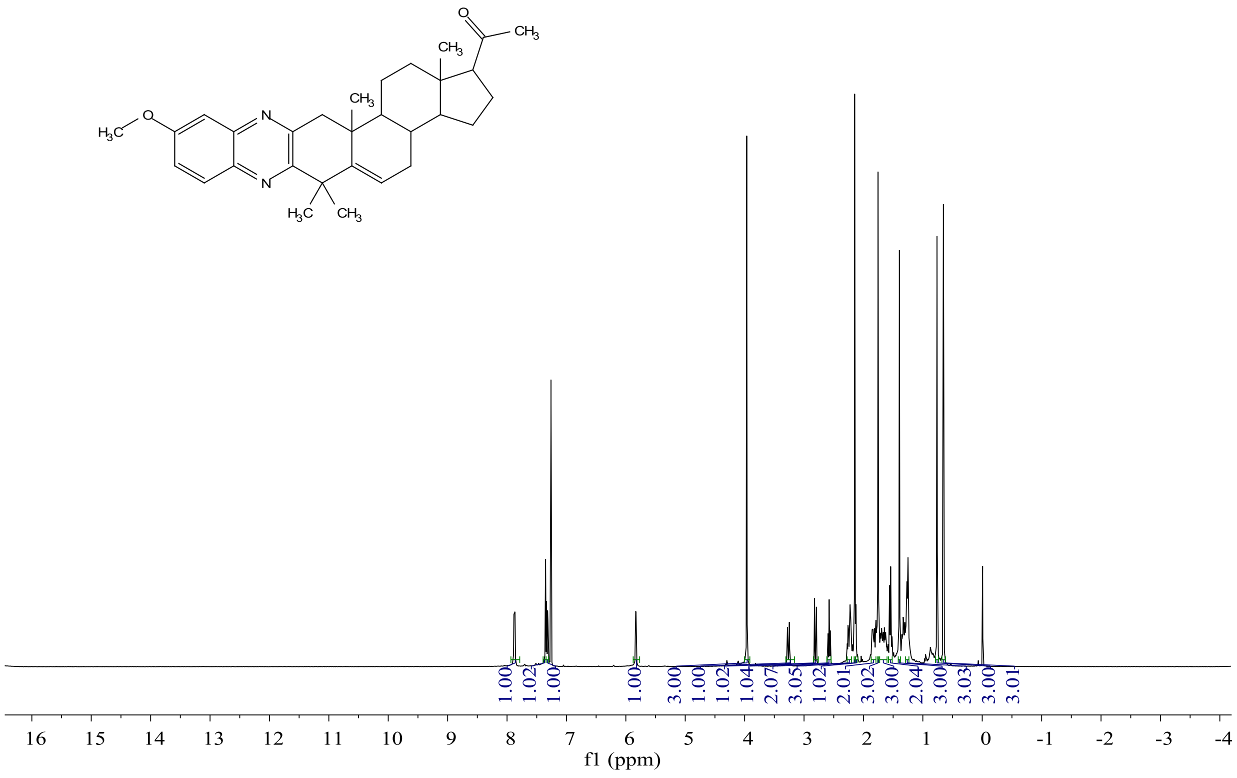

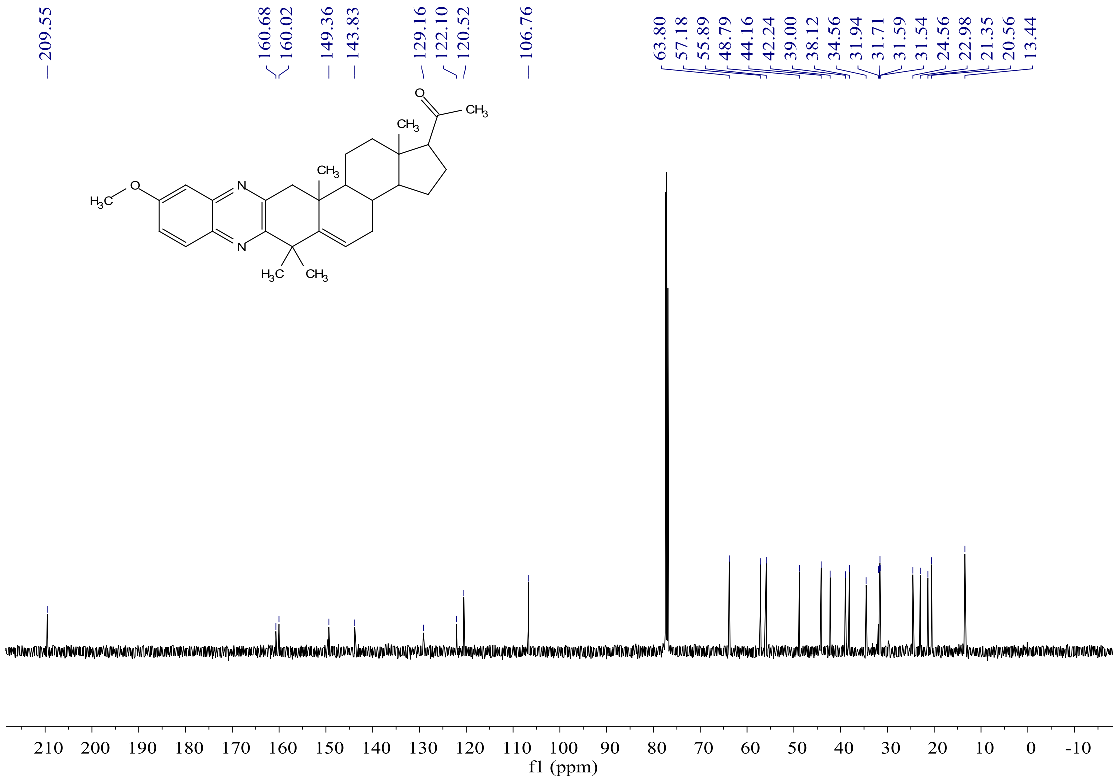
209.55
160.68
160.02
149.36
143.83
129.16
122.10
120.52
106.76
63.80
57.18
55.89
48.79
44.16
42.24
39.00
38.12
34.56
31.94
31.71
31.59
31.54
24.56
22.98
21.35
20.56
13.44
210 200 190 180 170 160 150 140 130 120 110 100 90 80 70 60 50 40 30 20 10 0 -10
f1 (ppm)

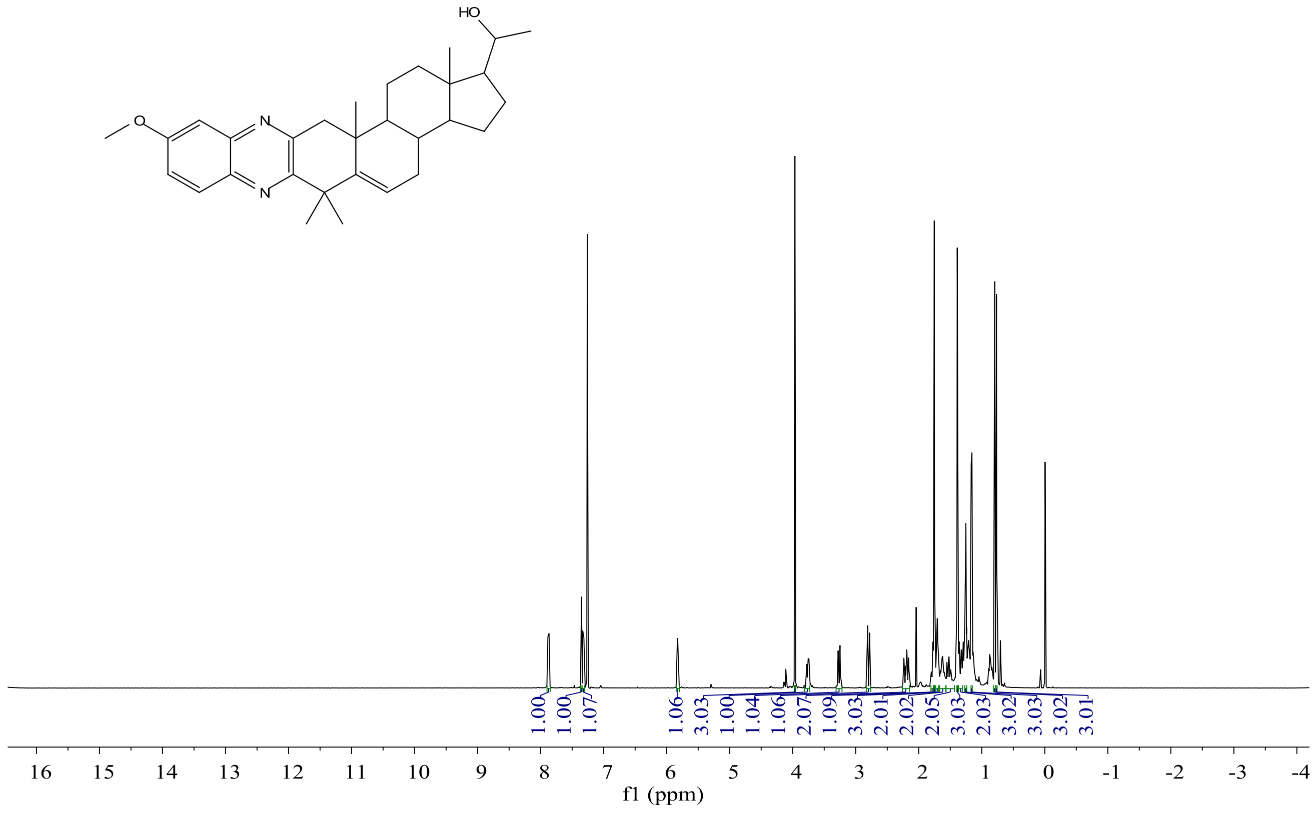
HO
N
O
1.00
1.00
1.07
1.06
3.03
1.00
1.04
1.06
2.07
1.09
3.03
2.01
2.02
2.05
3.03
2.03
3.02
3.03
3.02
3.01
16 15 14 13 12 11 10 9 8 7 6 5 4 3 2 1 0 -1 -2 -3 -4
f1 (ppm)

Compound **9l** and **10l**

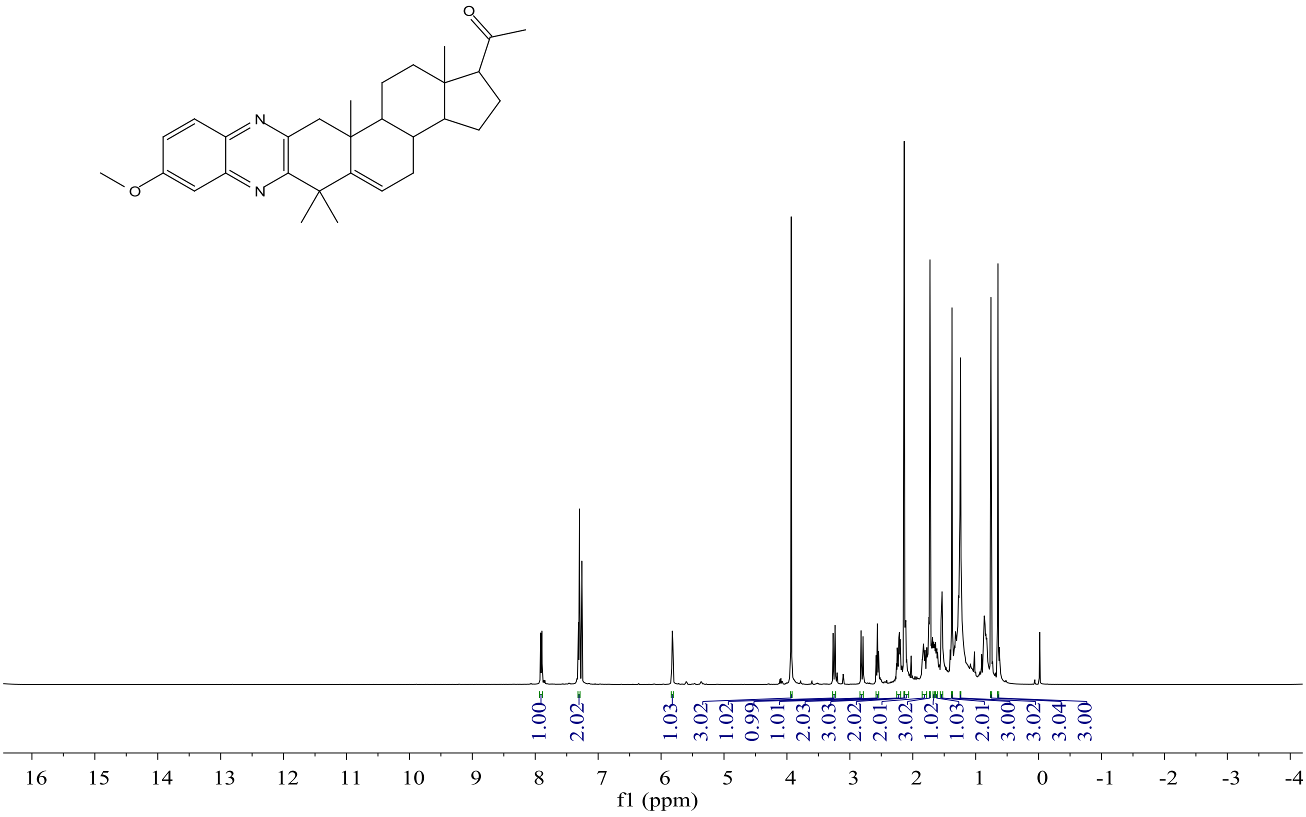

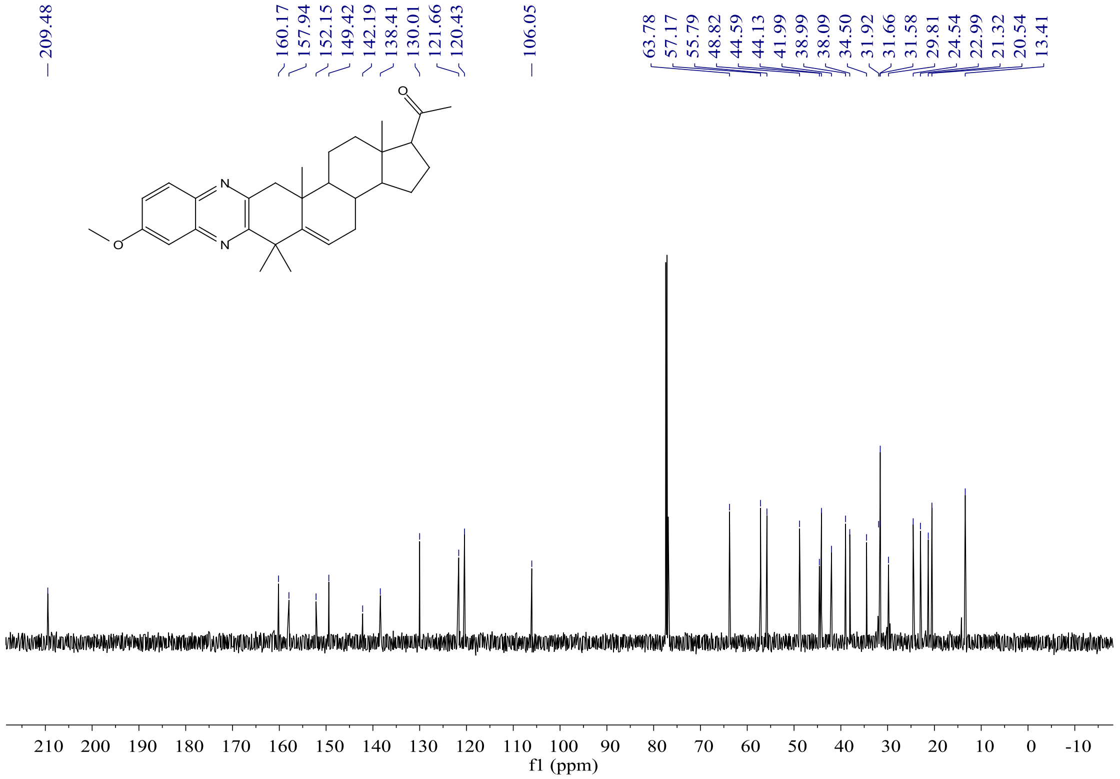

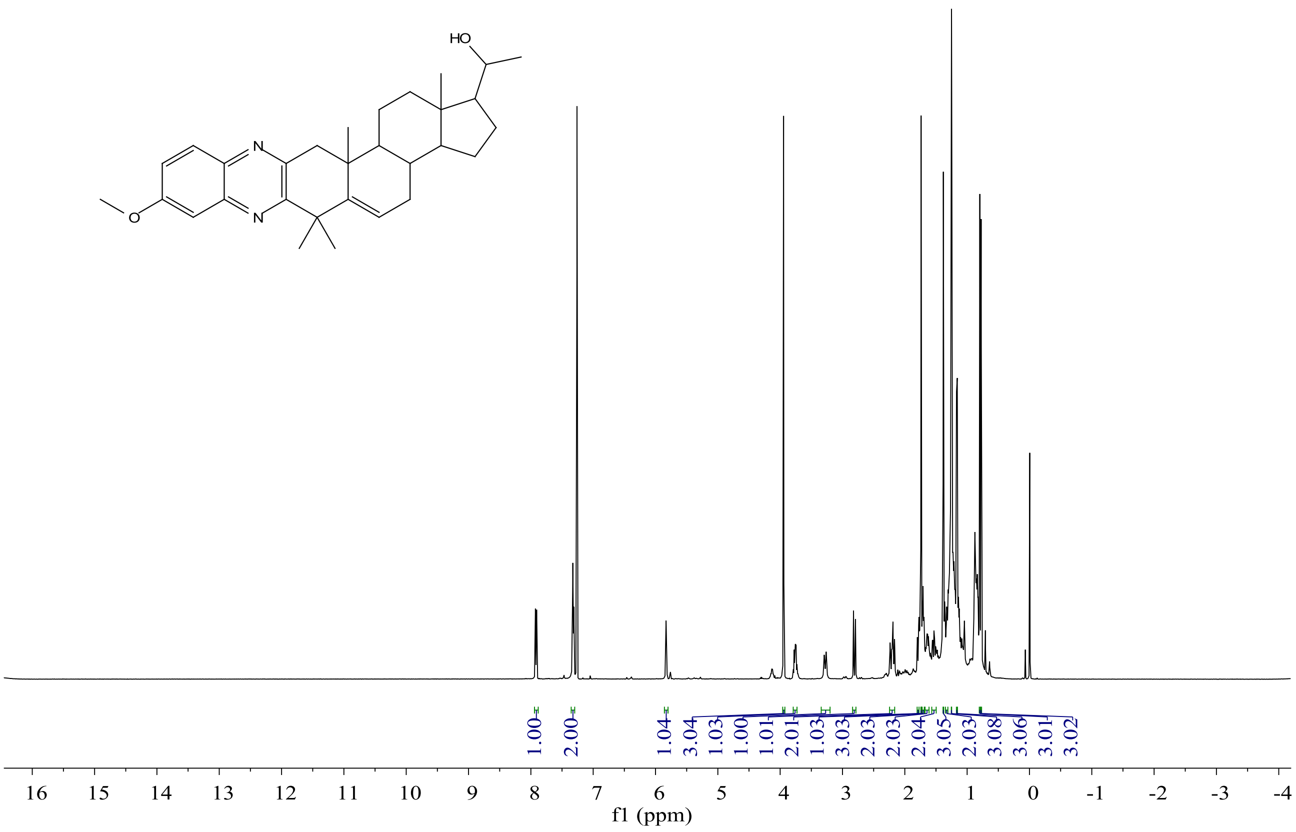

Compound **9m** and **10m**

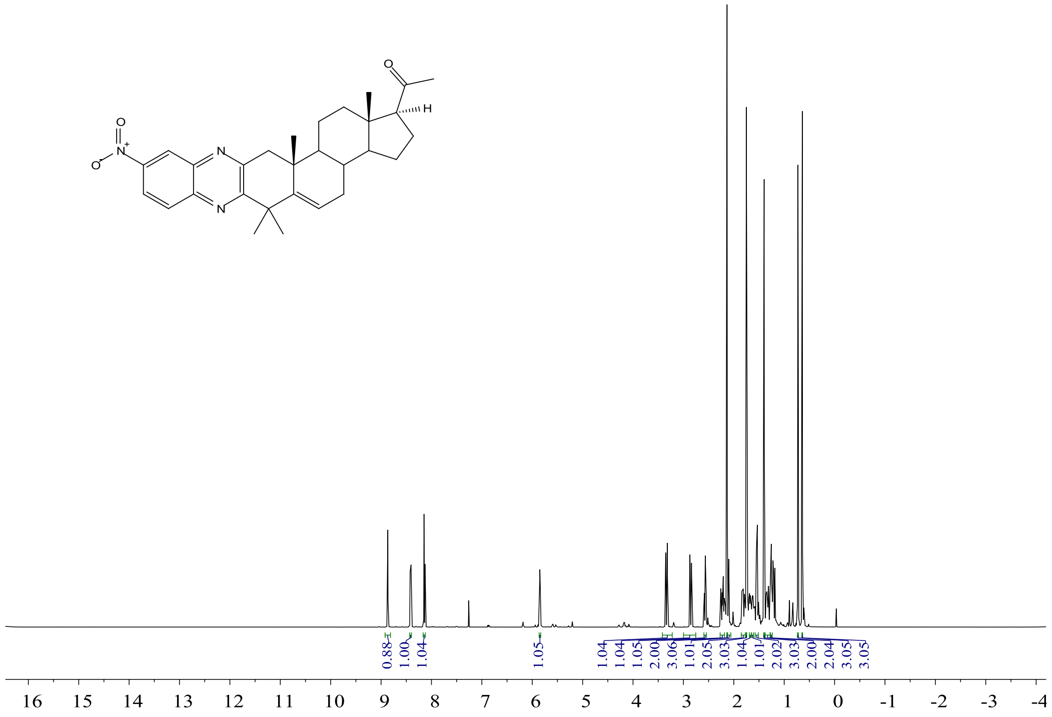

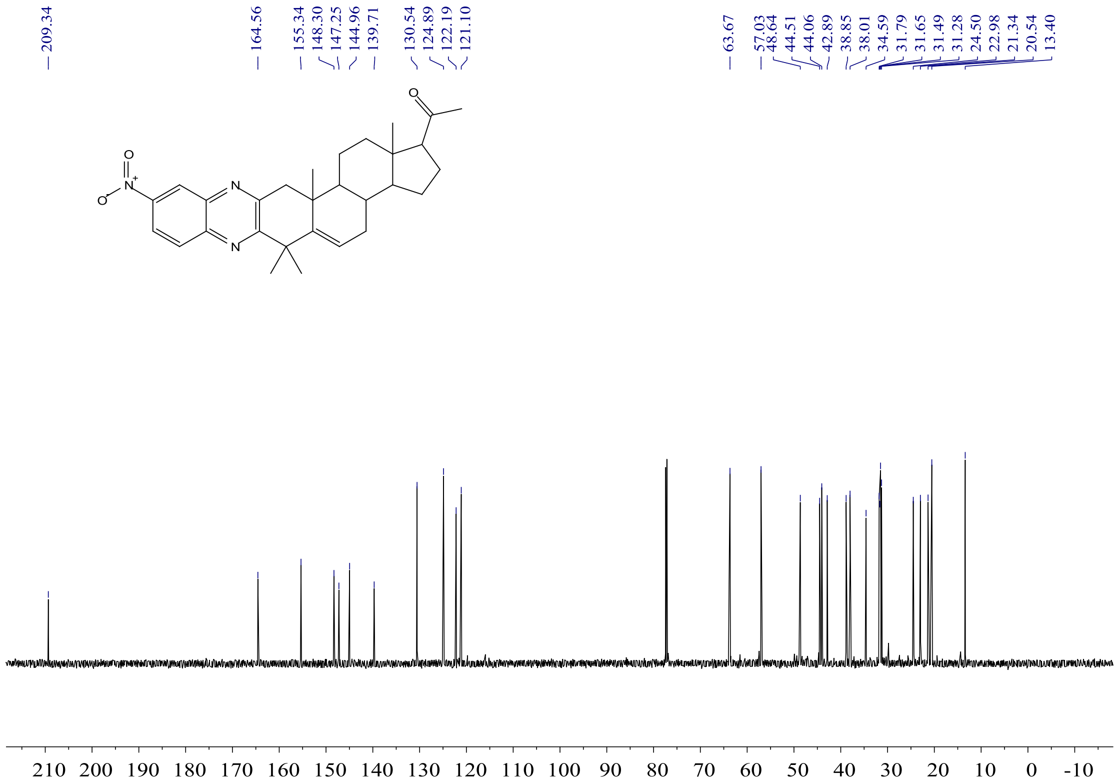
209.34
164.56
155.34
148.30
147.25
144.96
139.71
130.54
124.89
122.19
121.10
63.67
57.03
48.64
44.51
44.06
42.89
38.85
38.01
34.59
31.79
31.65
31.49
31.28
24.50
22.98
21.34
20.54
13.40
O
N+
O-
N
N
210 200 190 180 170 160 150 140 130 120 110 100 90 80 70 60 50 40 30 20 10 0 -10

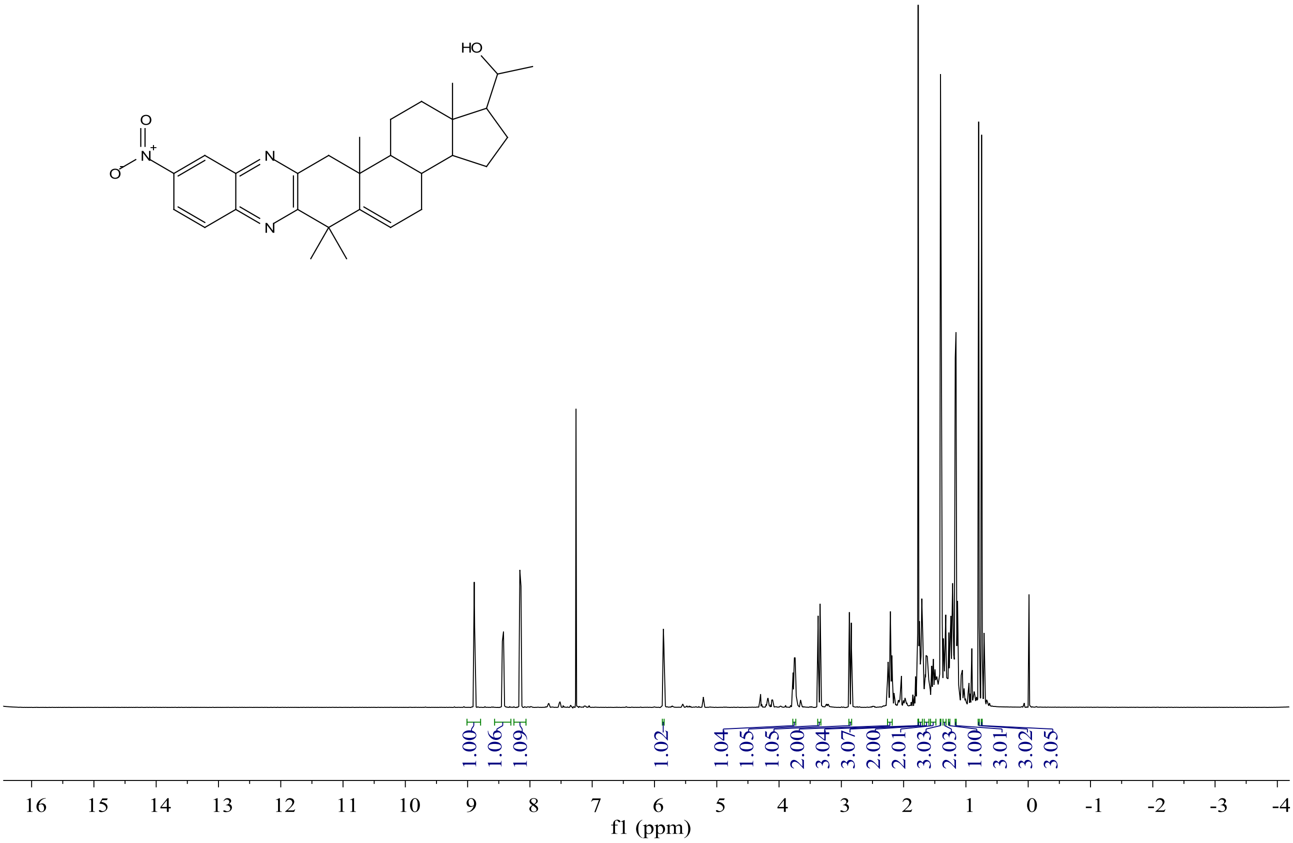
HO
O
N+
O-
N
N
1.00
1.06
1.09
1.02
1.04
1.05
1.05
2.00
3.04
3.07
2.00
2.01
3.03
2.03
1.00
3.01
3.02
3.05
16 15 14 13 12 11 10 9 8 7 6 5 4 3 2 1 0 -1 -2 -3 -4
f1 (ppm)

Compound **9n** and **10n**

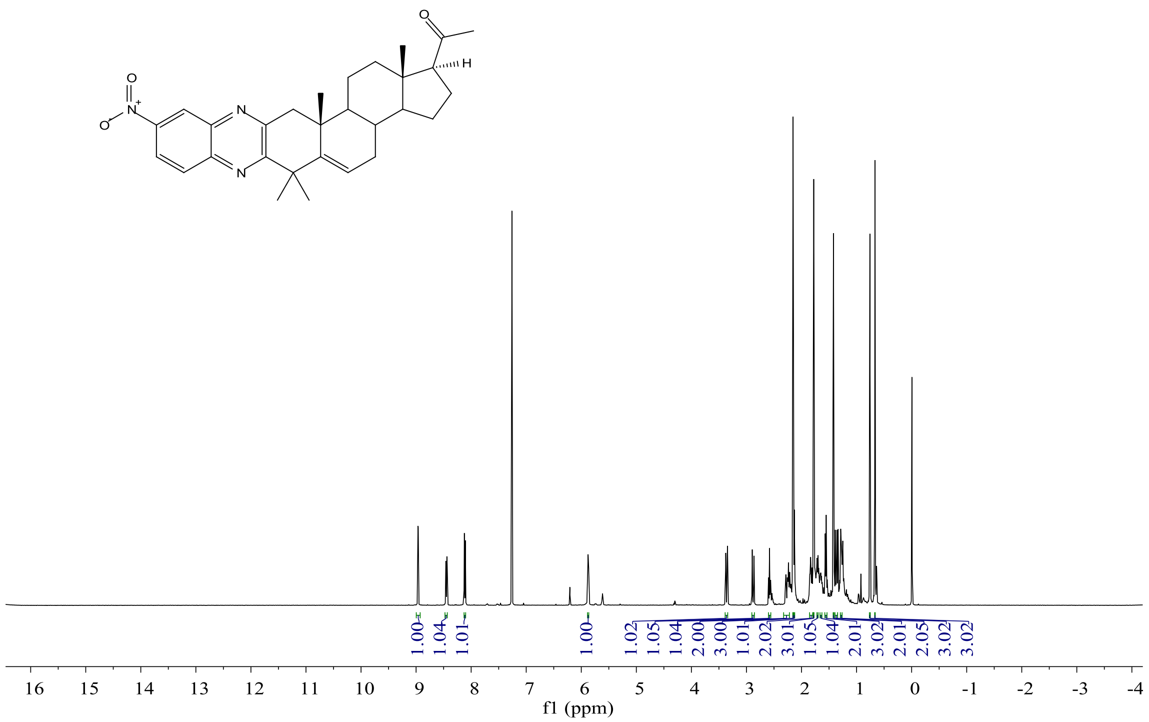

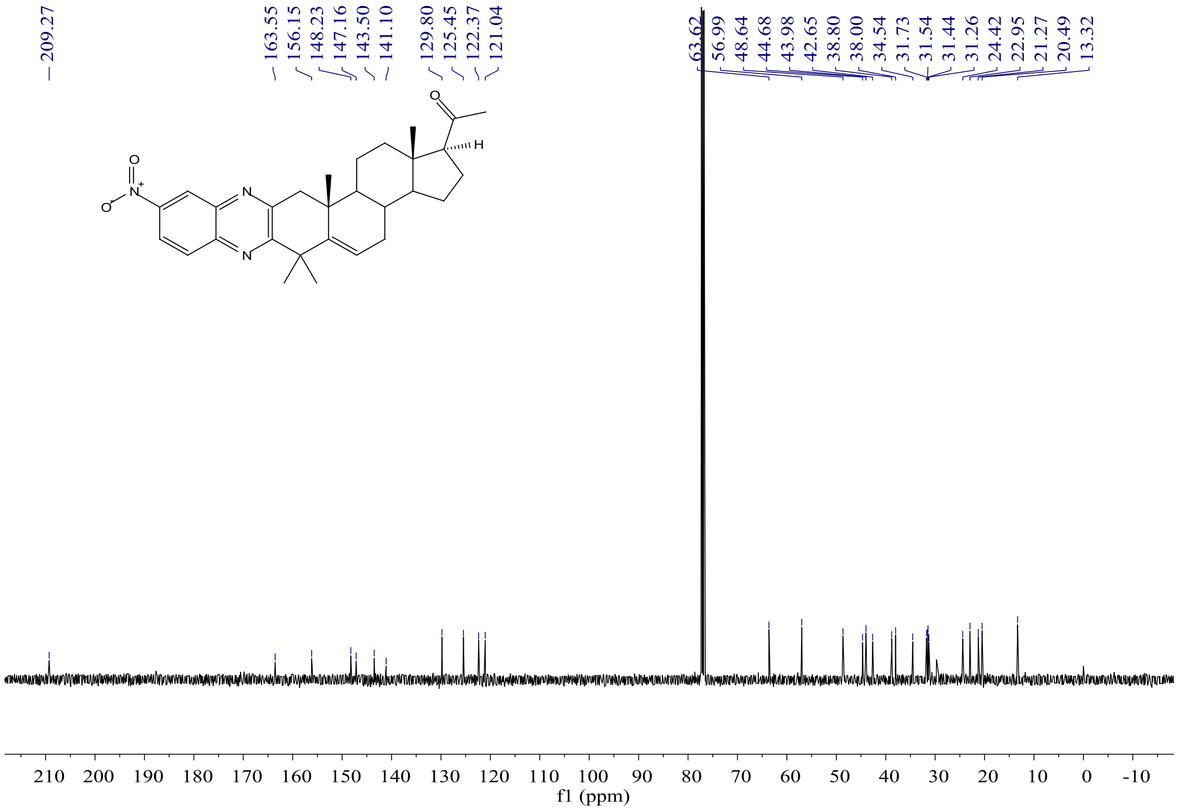

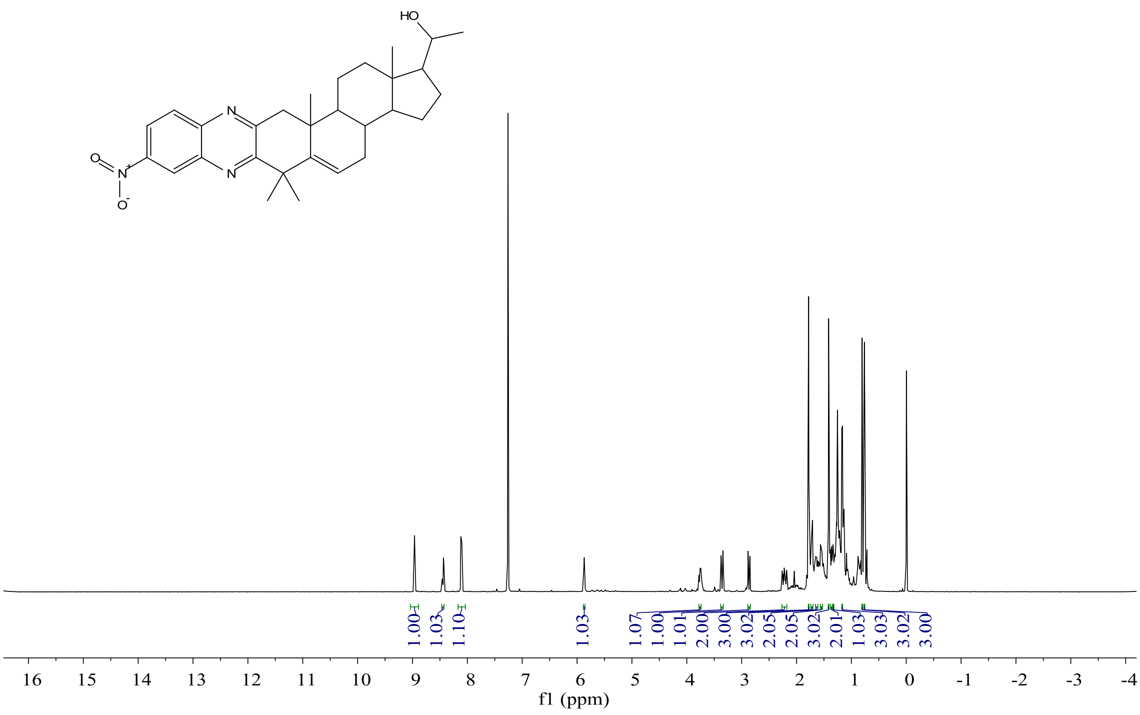

Compound **9o** and **10o**

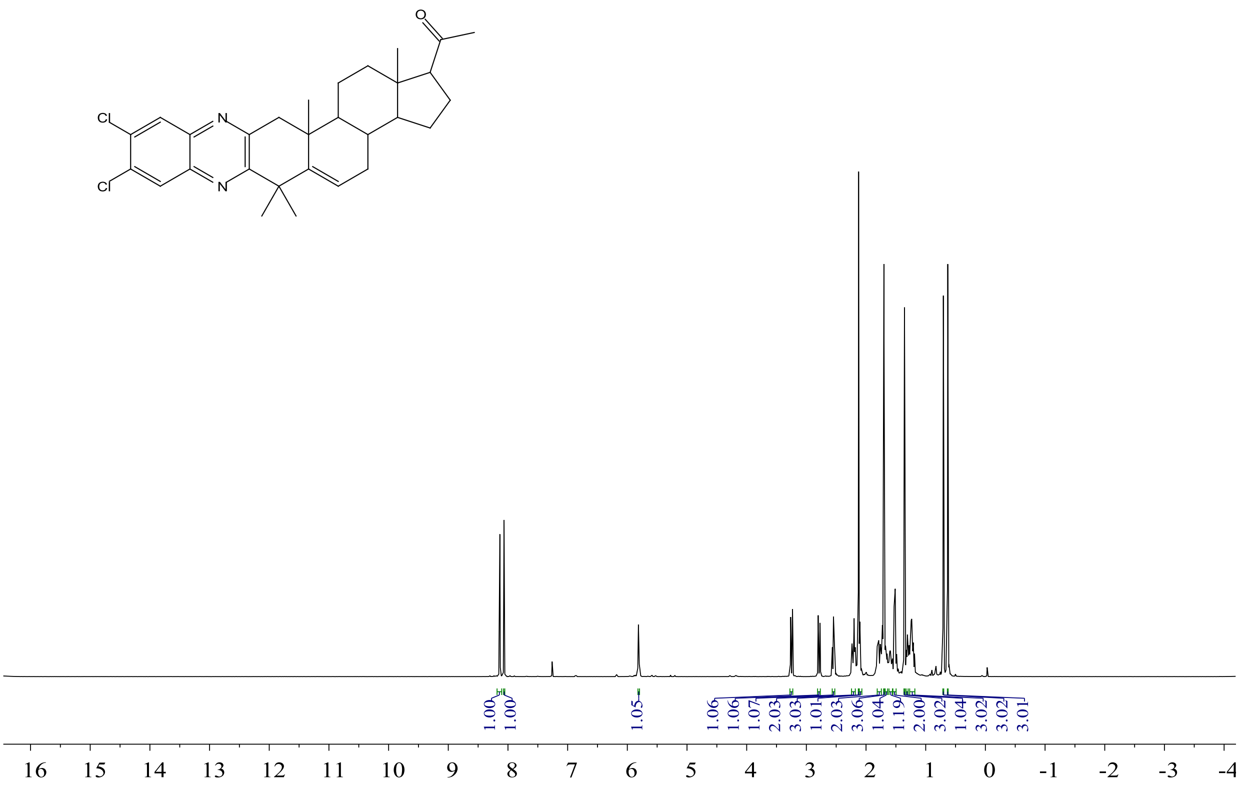

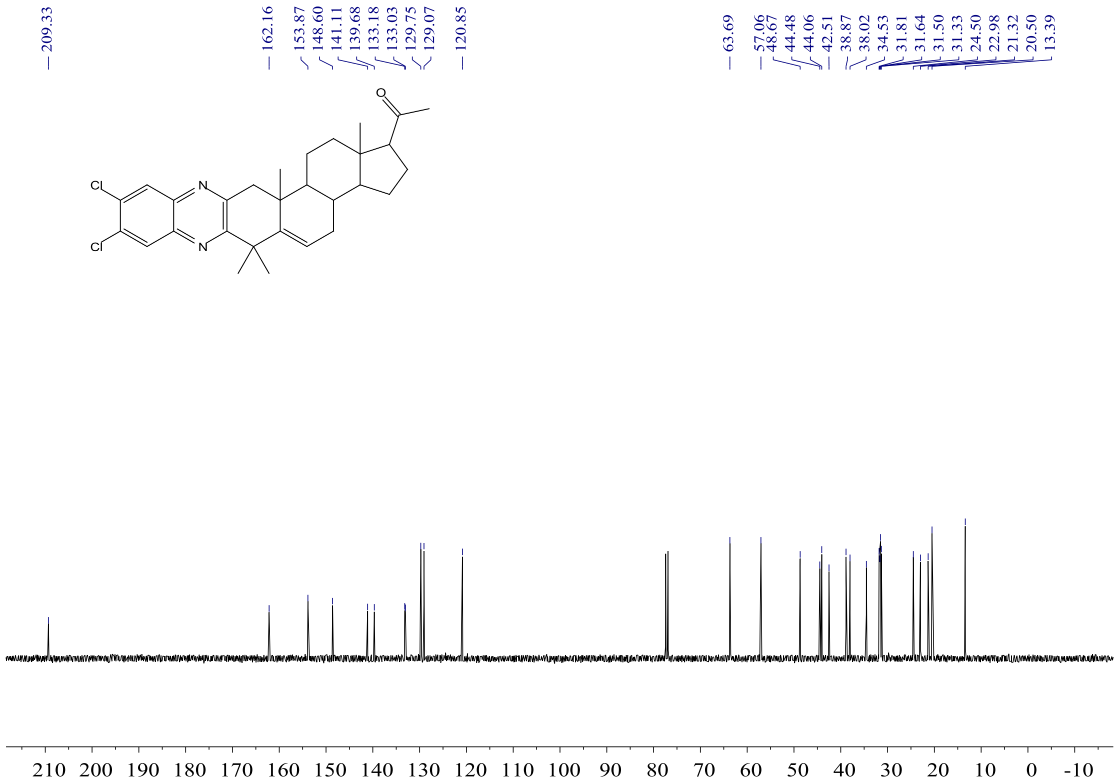
209.33
162.16
153.87
148.60
141.11
139.68
133.18
133.03
129.75
129.07
120.85
63.69
57.06
48.67
44.48
44.06
42.51
38.87
38.02
34.53
31.81
31.64
31.50
31.33
24.50
22.98
21.32
20.50
13.39
O
Cl
Cl
N
N
210 200 190 180 170 160 150 140 130 120 110 100 90 80 70 60 50 40 30 20 10 0 -10

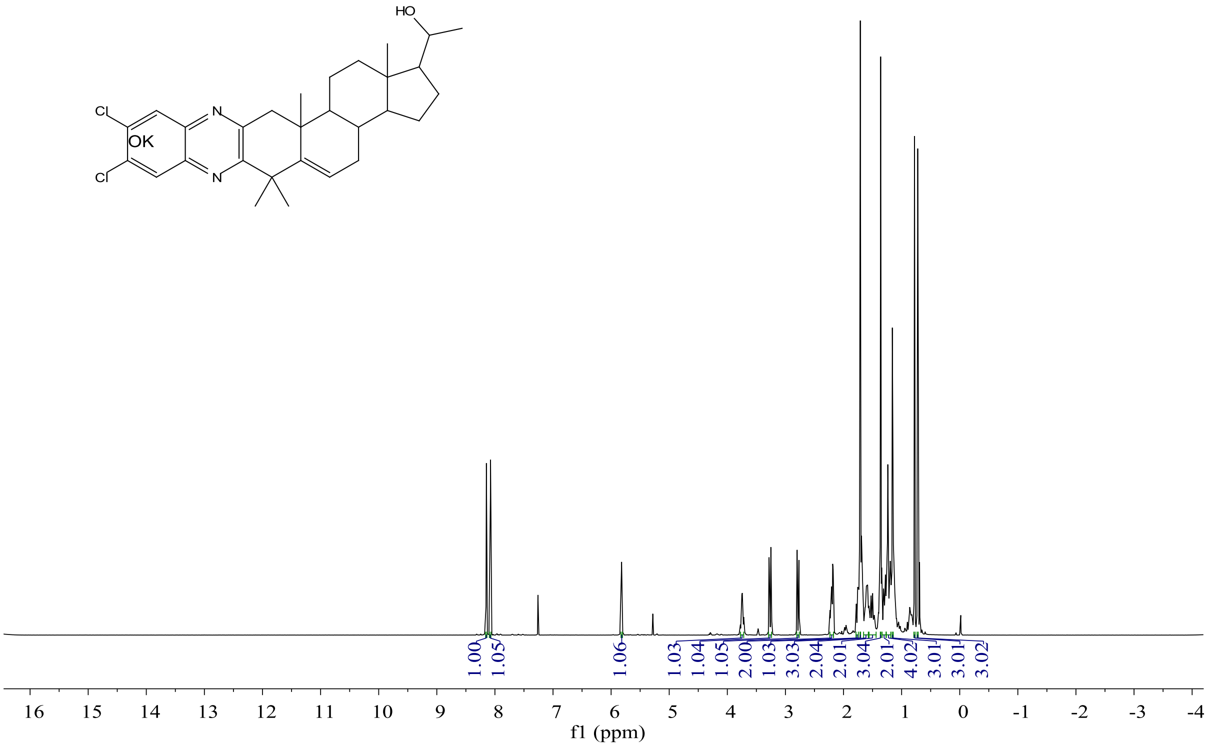
HO
Cl
Cl
N
N
OK
1.00
1.05
1.06
1.03
1.04
1.05
2.00
1.03
3.03
2.04
2.01
3.04
2.01
4.02
3.01
3.01
3.02
16 15 14 13 12 11 10 9 8 7 6 5 4 3 2 1 0 -1 -2 -3 -4
f1 (ppm)

3.5. The ^{1}H and ^{13}C NMR Spectra of Compound ***11a-11o*** *and the ^{1}H NMR Spectra of Compound* ***12a-12o***

Compound **11a** and **12a**

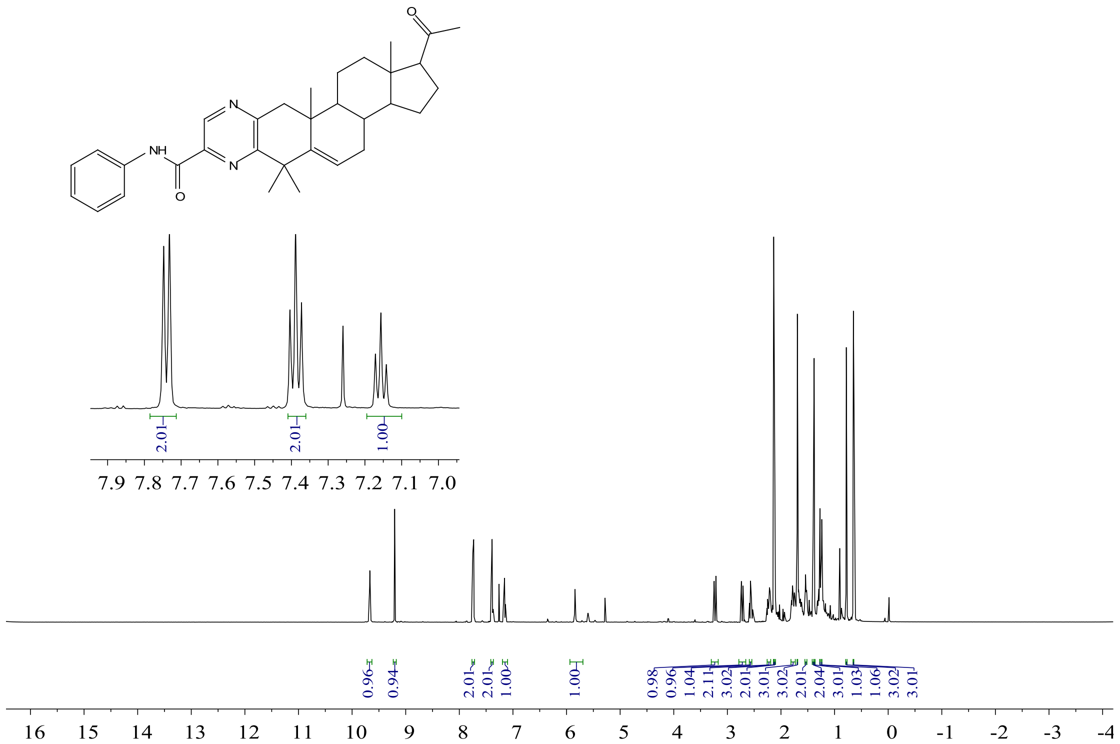

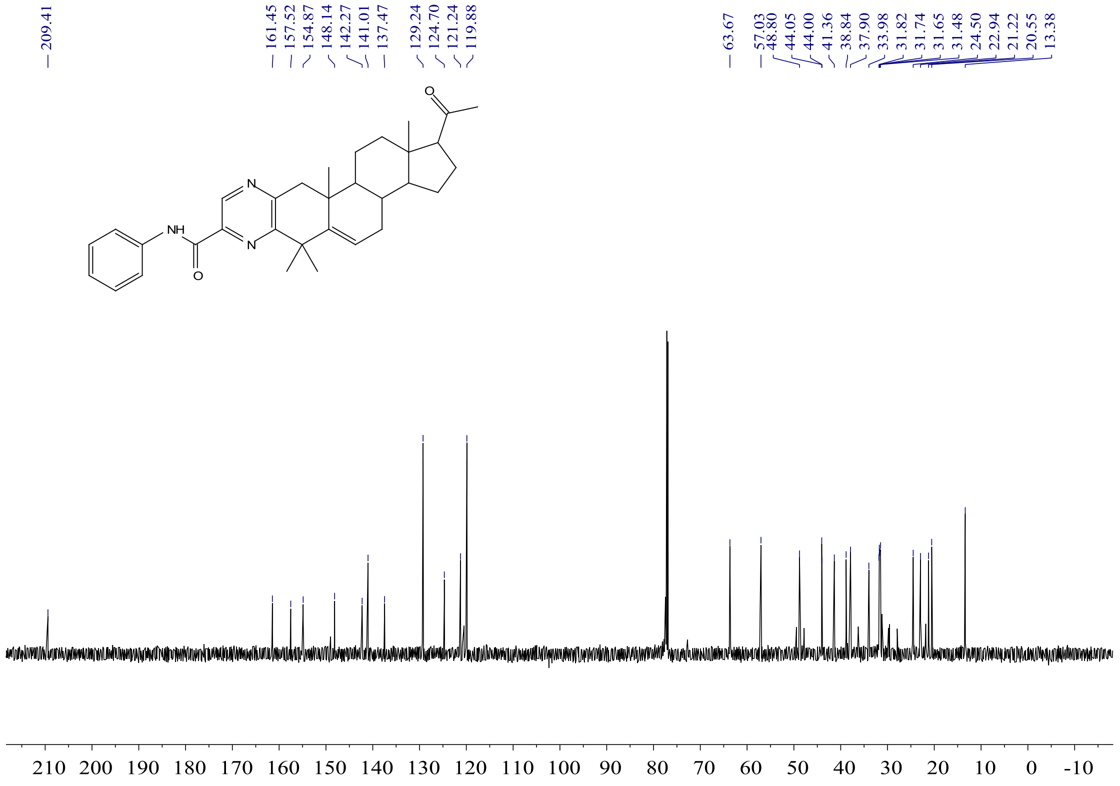

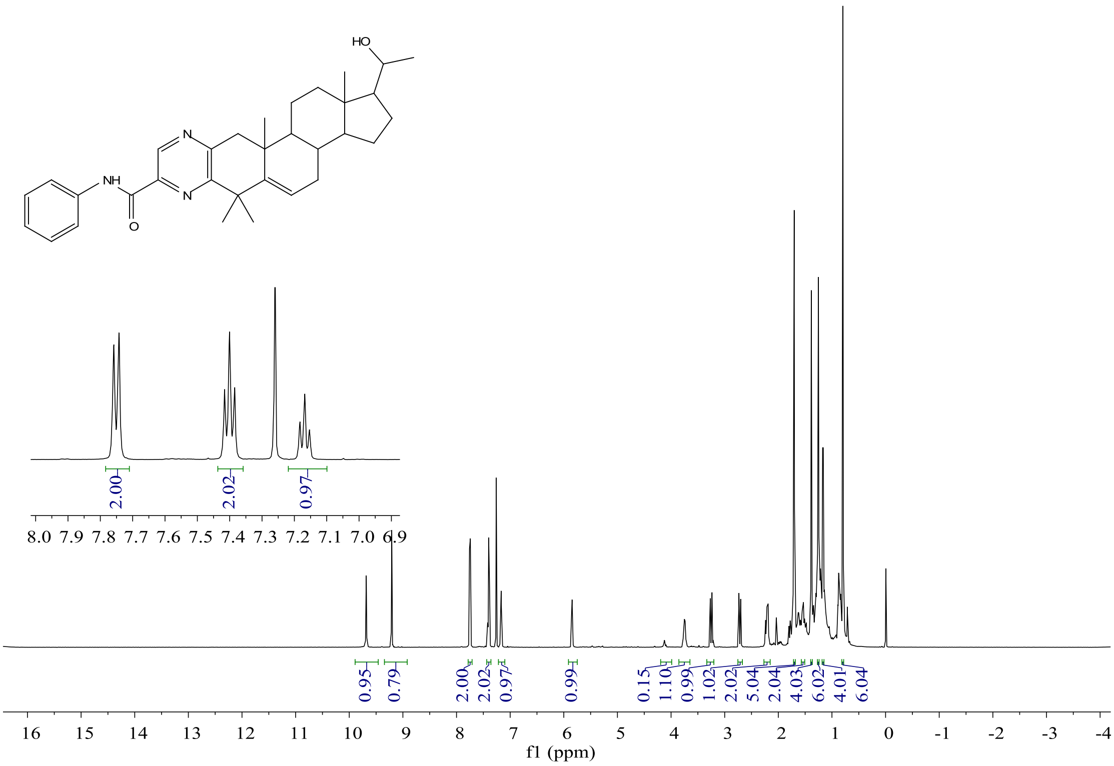

Compound **11b** and **12b**

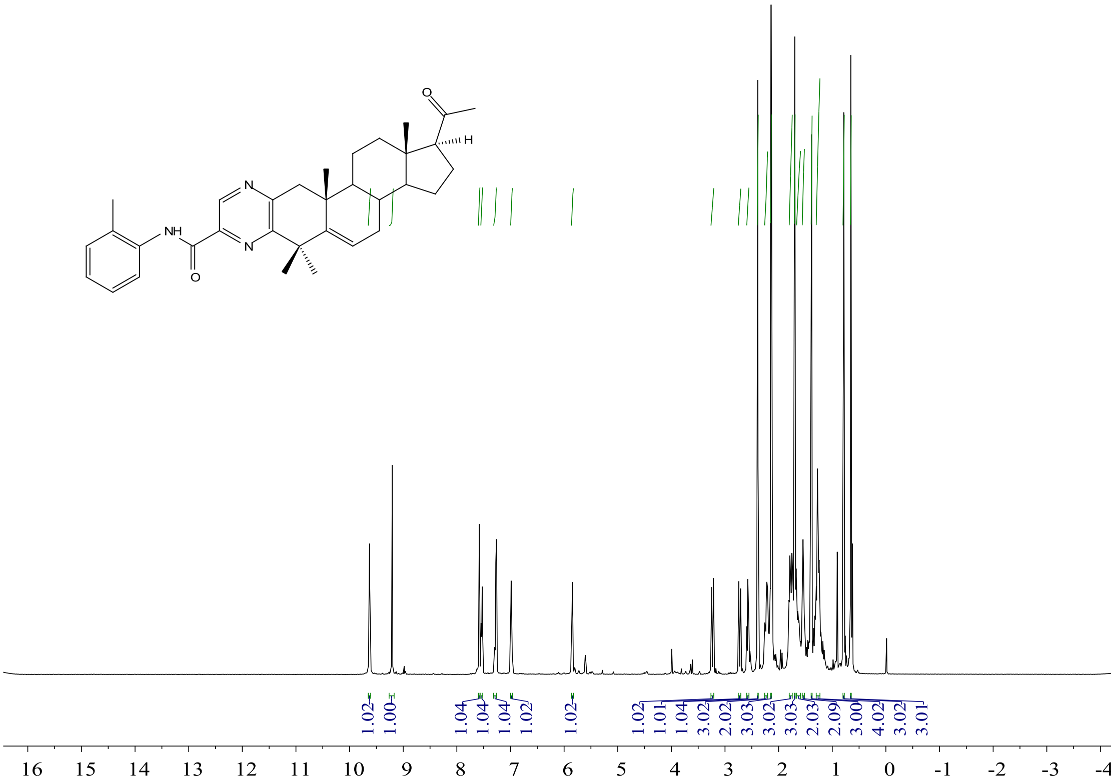

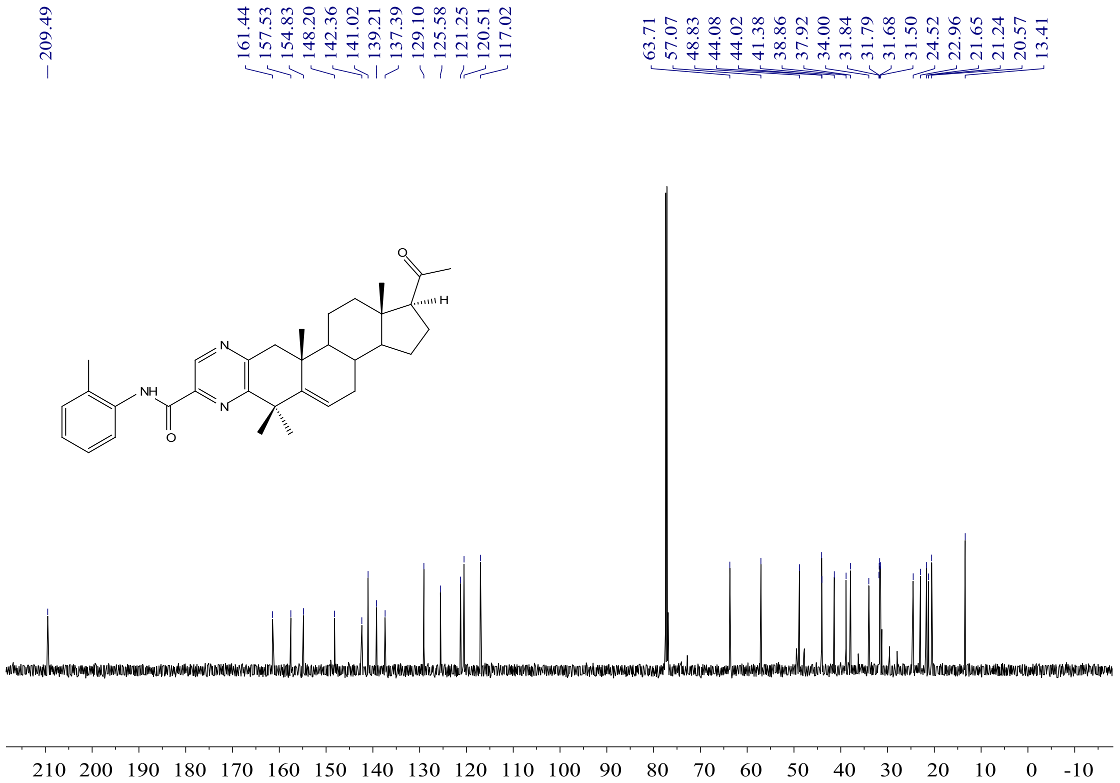

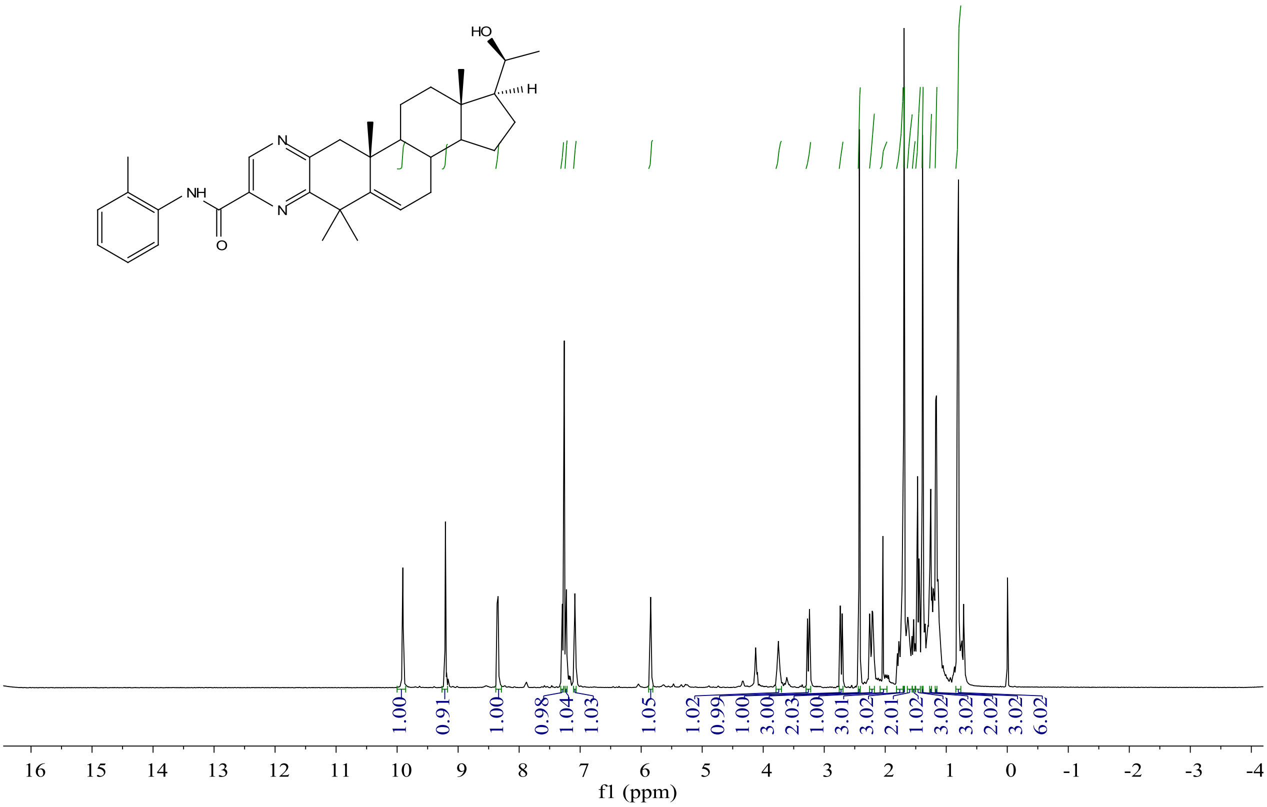

Compound **11c** and **12c**

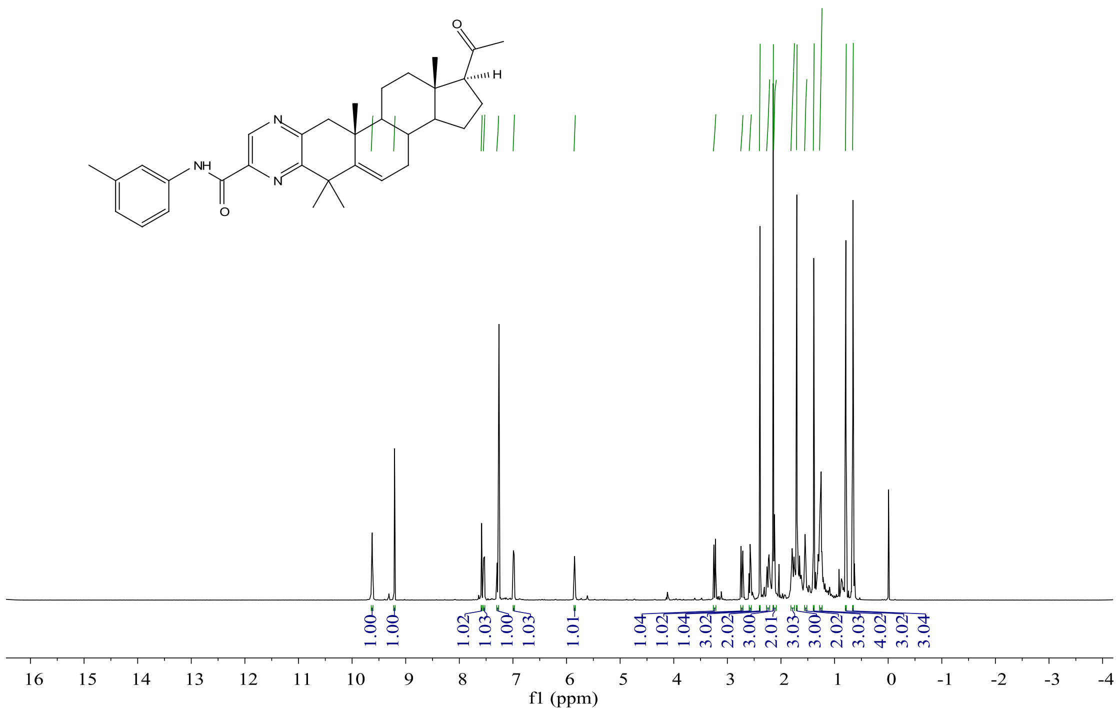

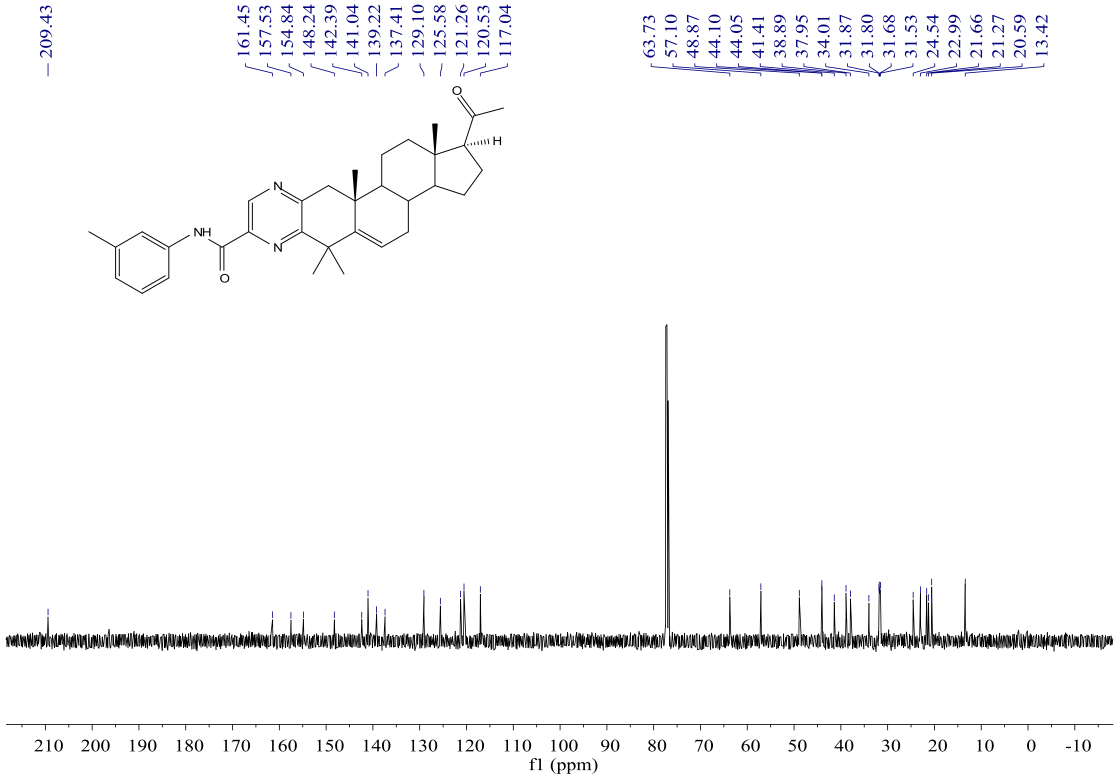

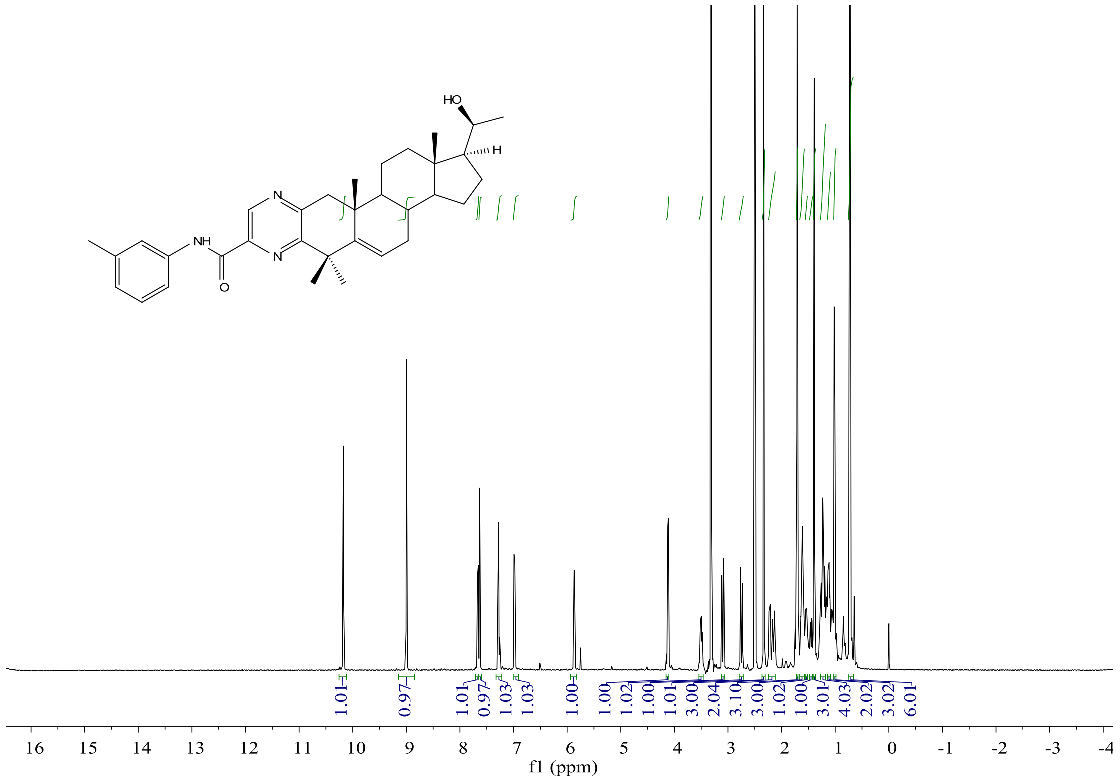

Compound **11d** and **12d**

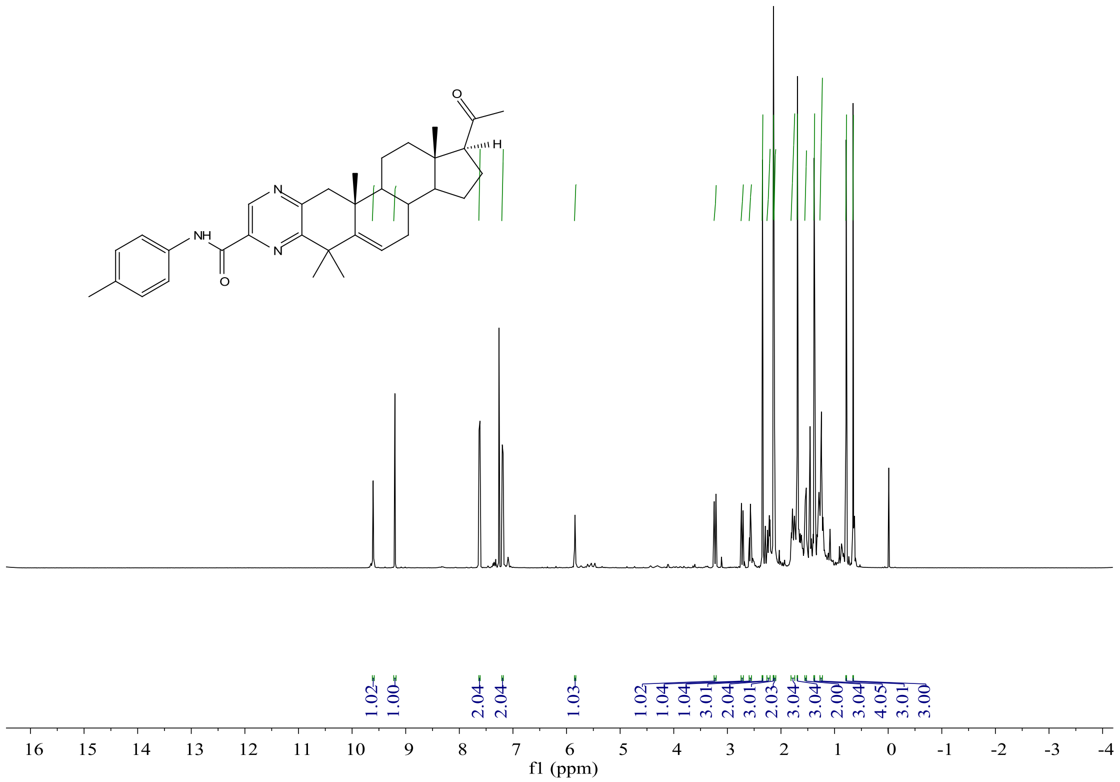

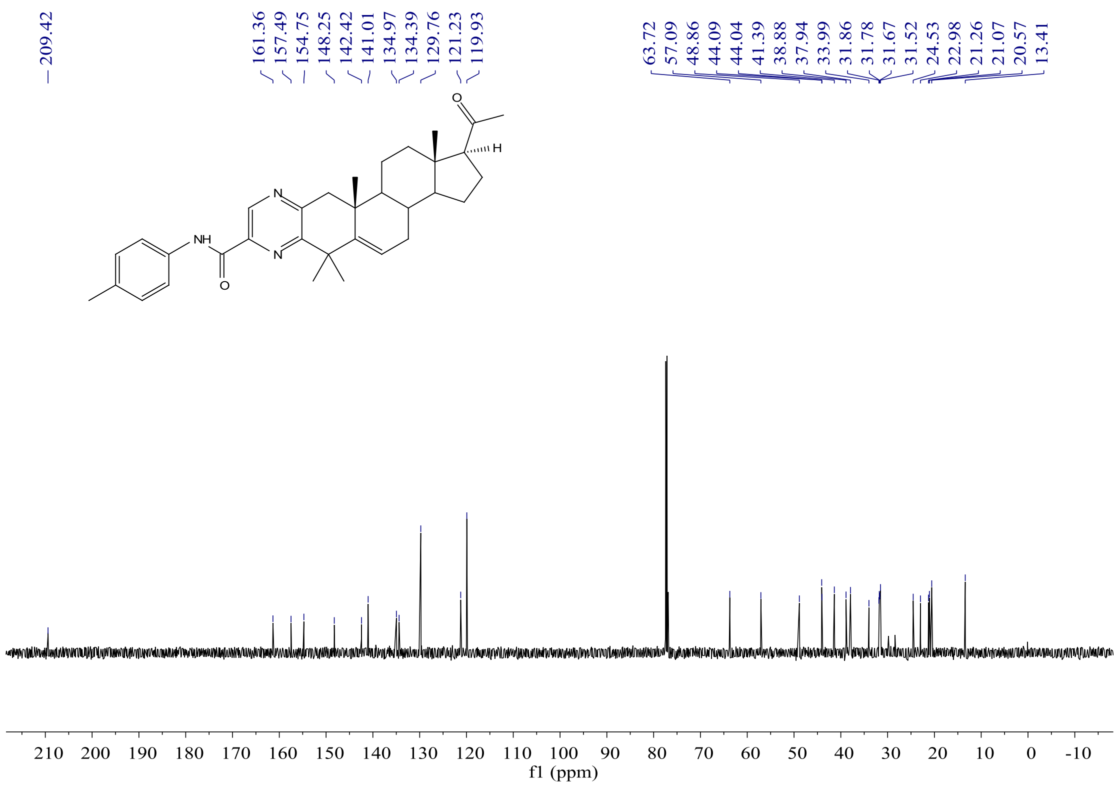

209.42
161.36
157.49
154.75
148.25
142.42
141.01
134.97
134.39
129.76
121.23
119.93
63.72
57.09
48.86
44.09
44.04
41.39
38.88
37.94
33.99
31.86
31.78
31.67
31.52
24.53
22.98
21.26
21.07
20.57
13.41
O
H
N
NH
N
O
210 200 190 180 170 160 150 140 130 120 110 100 90 80 70 60 50 40 30 20 10 0 -10
f1 (ppm)

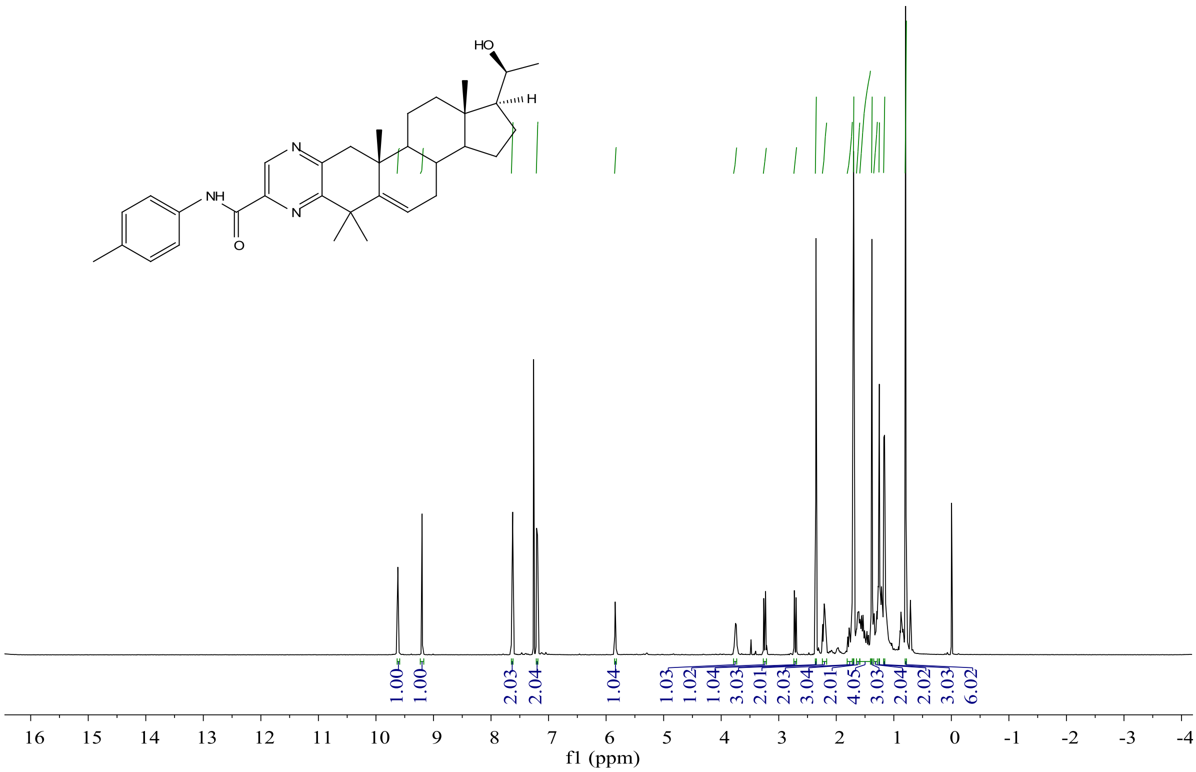

HO
H
N
NH
N
O
1.00
1.00
2.03
2.04
1.04
1.03
1.02
1.04
3.03
2.01
2.03
3.04
2.01
4.05
3.03
2.04
2.02
3.03
6.02
16 15 14 13 12 11 10 9 8 7 6 5 4 3 2 1 0 -1 -2 -3 -4
f1 (ppm)

Compound **11e** and **12e**

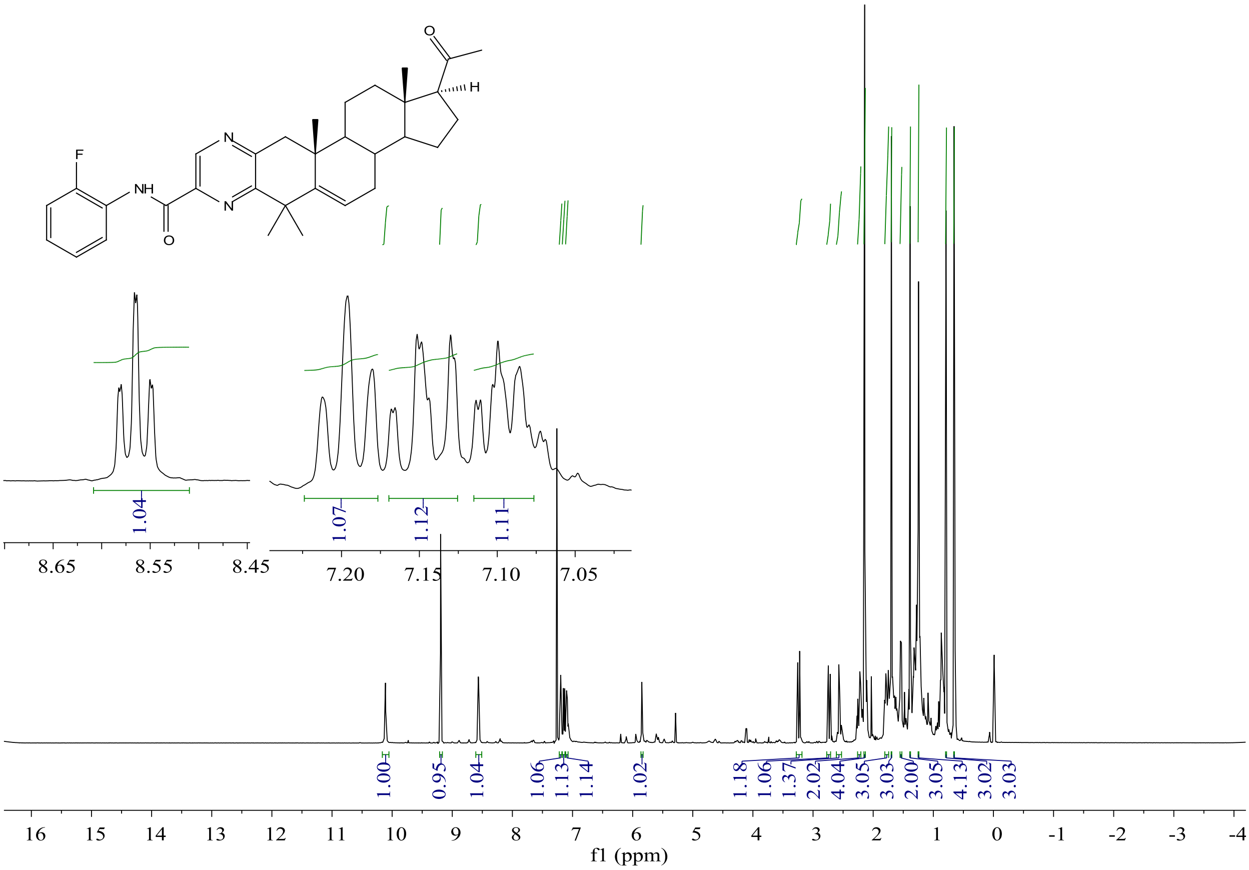

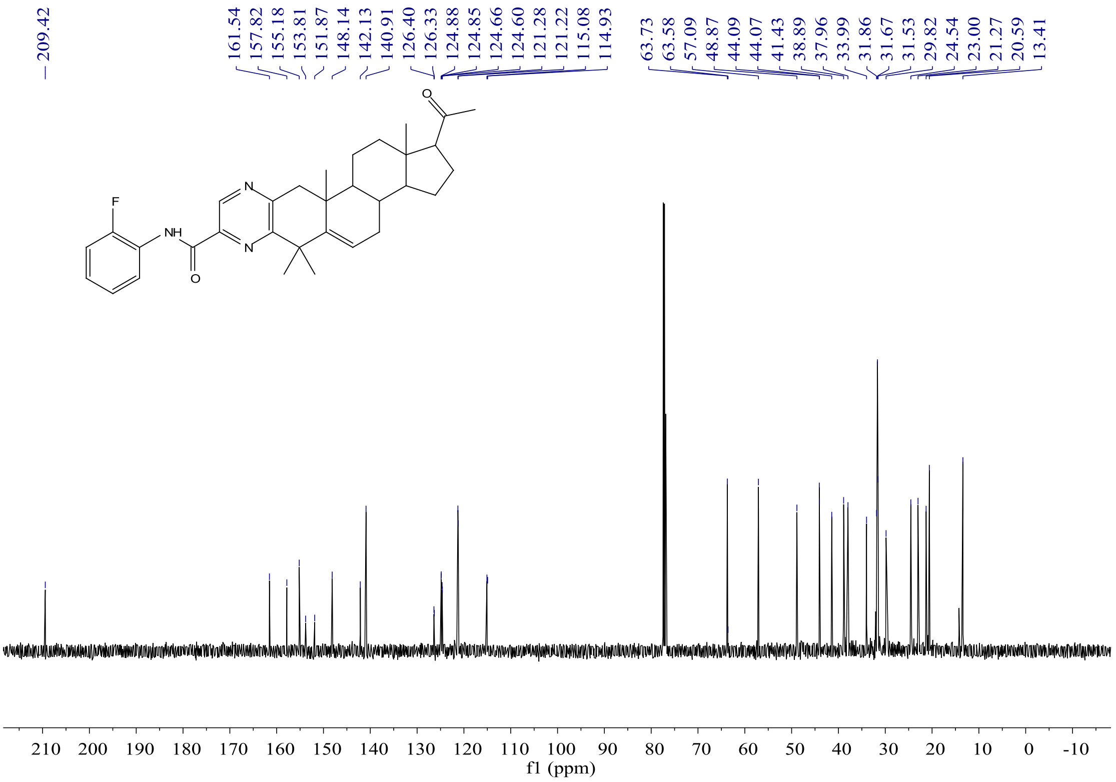

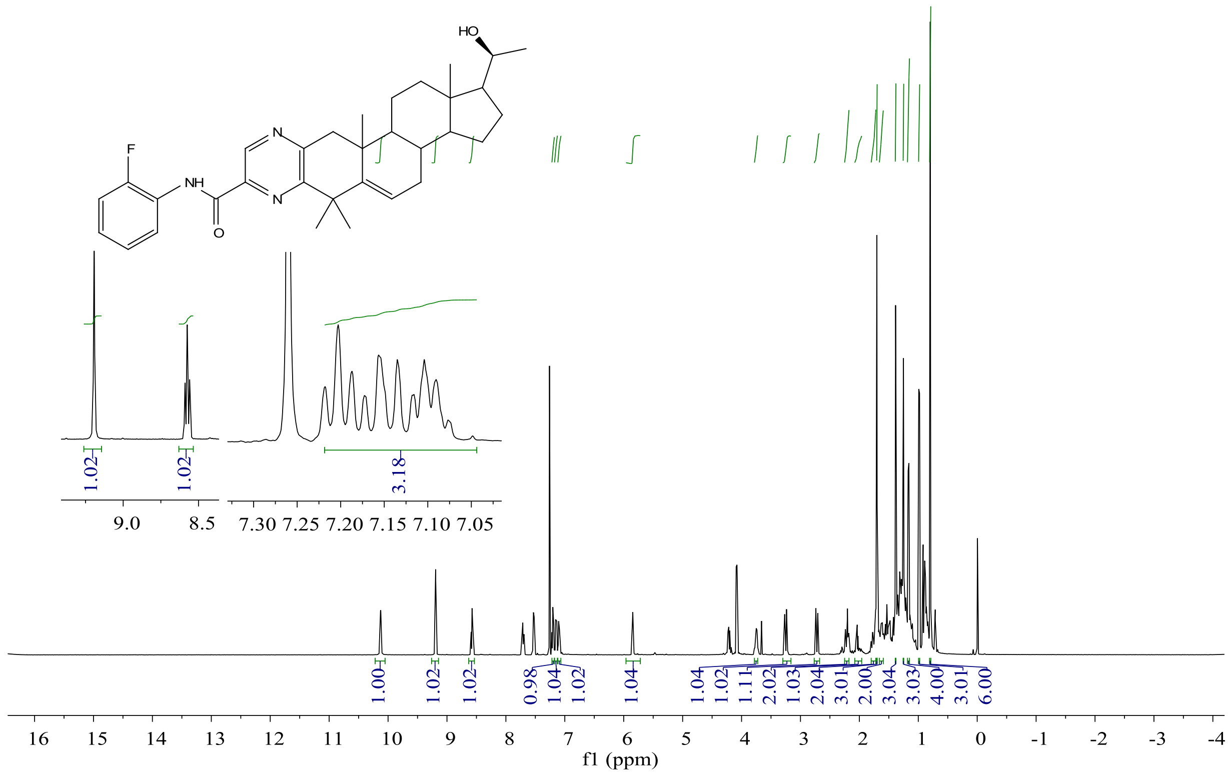

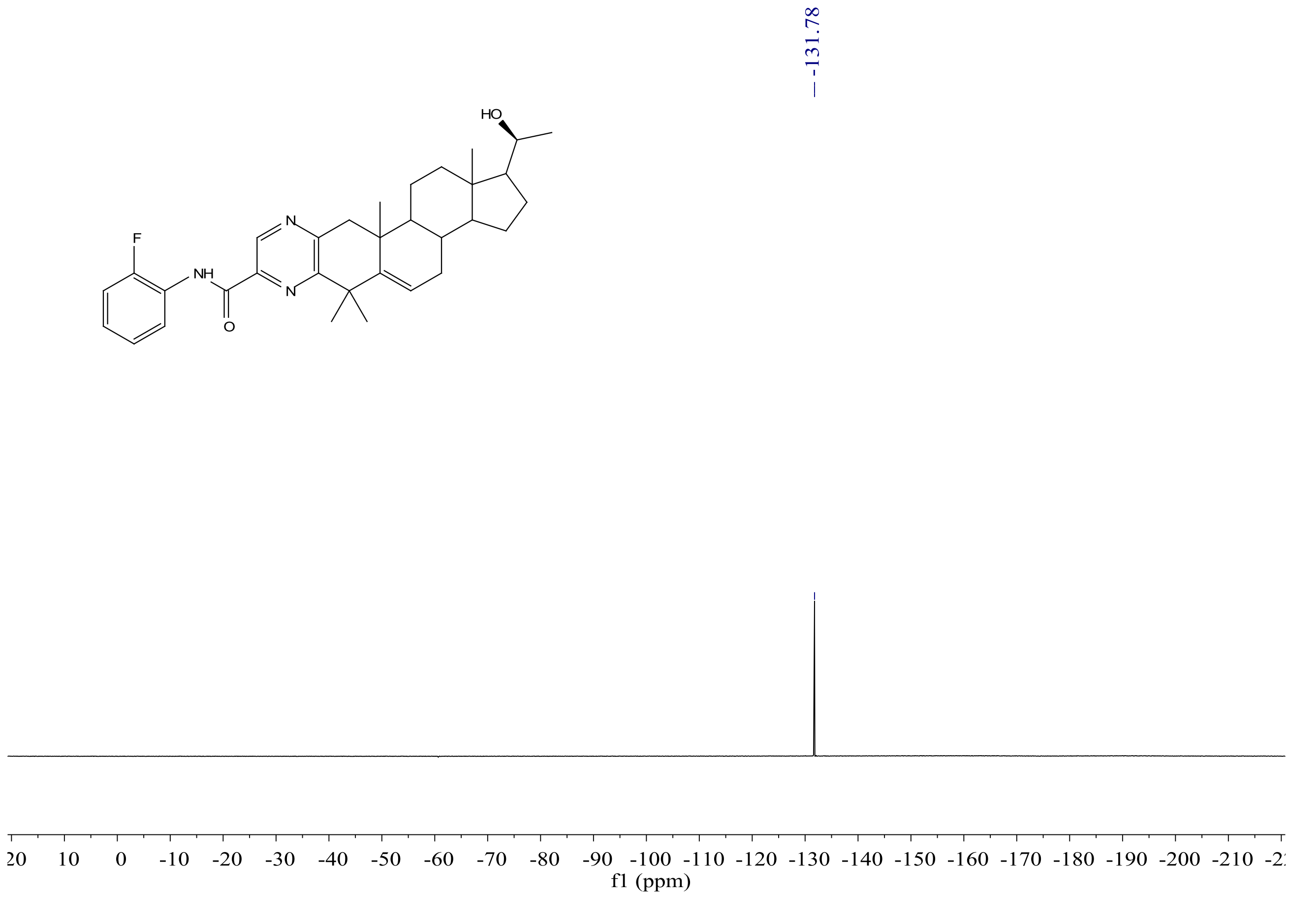

Compound **11f** and **12f**

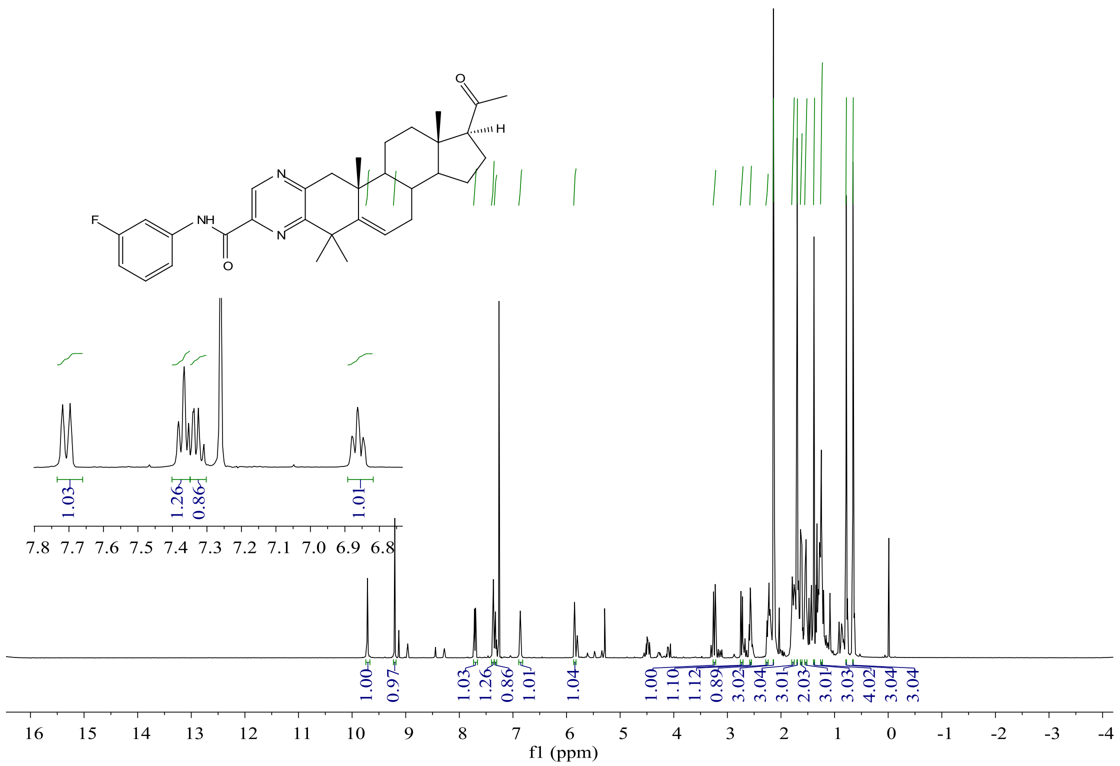

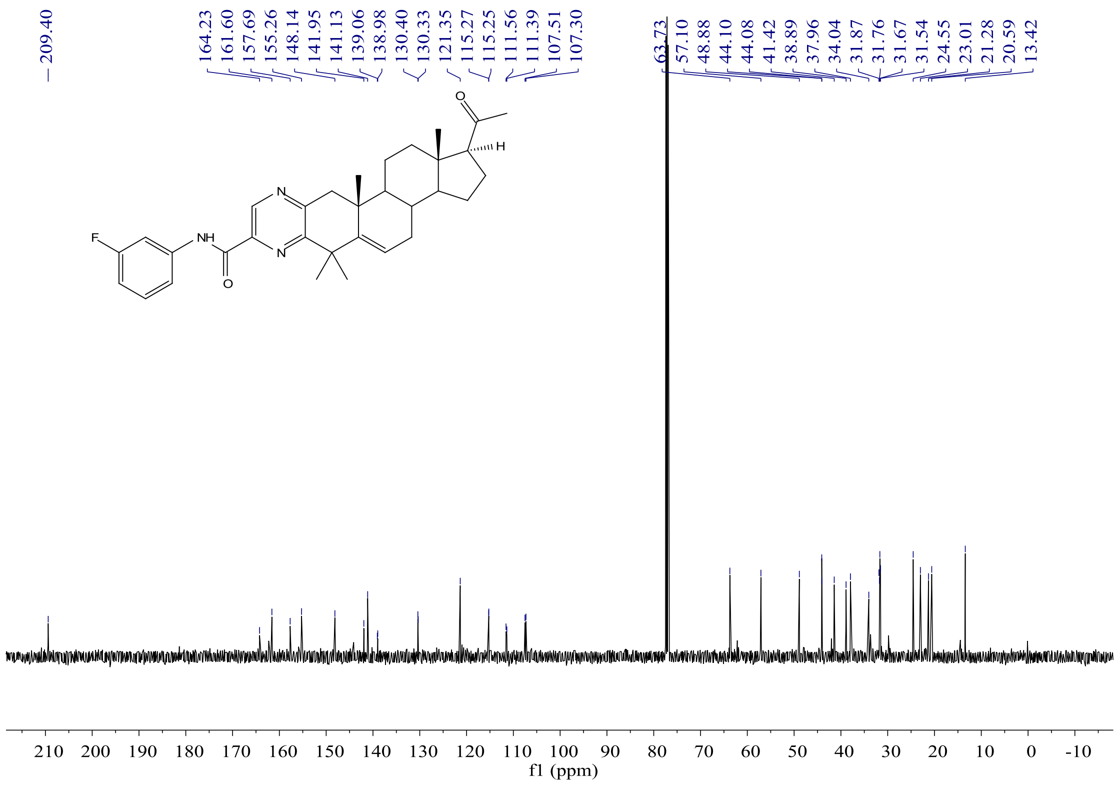

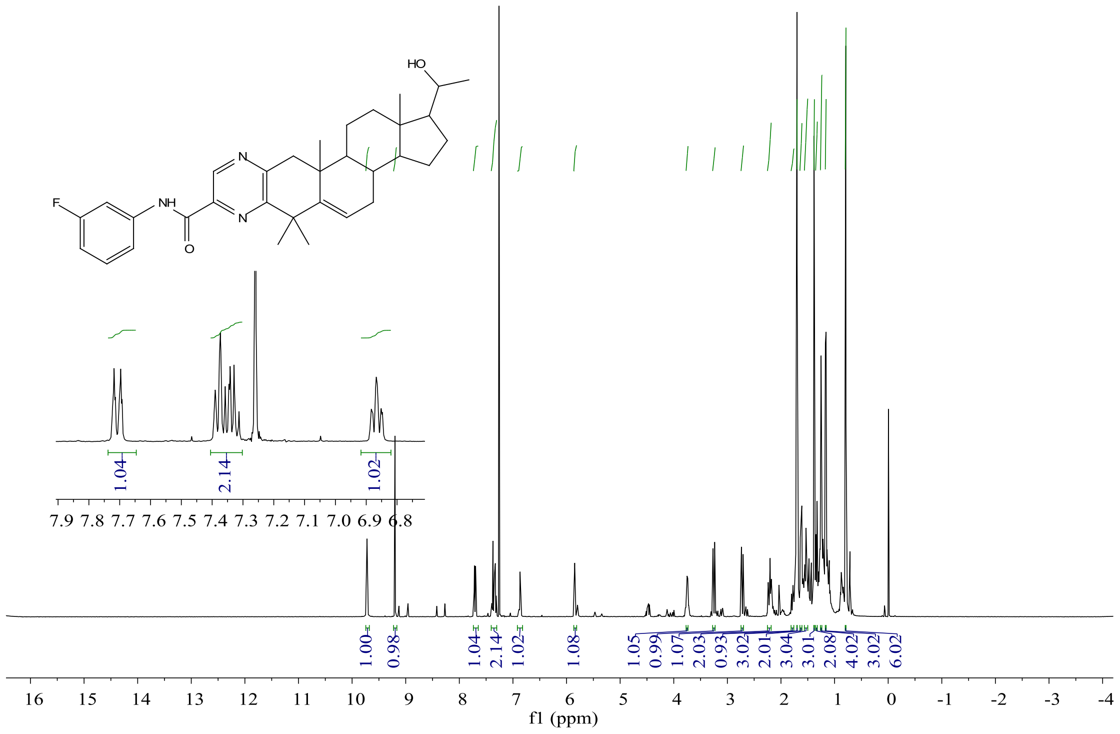

Compound **11g** and **12g**

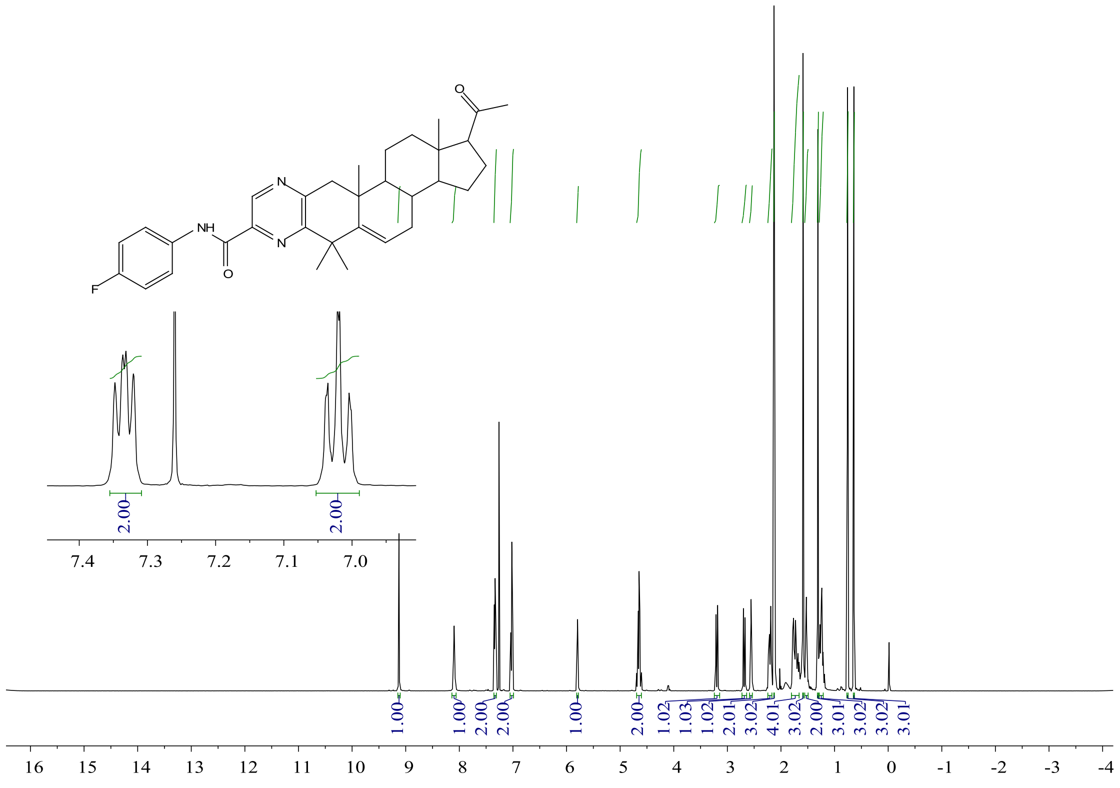

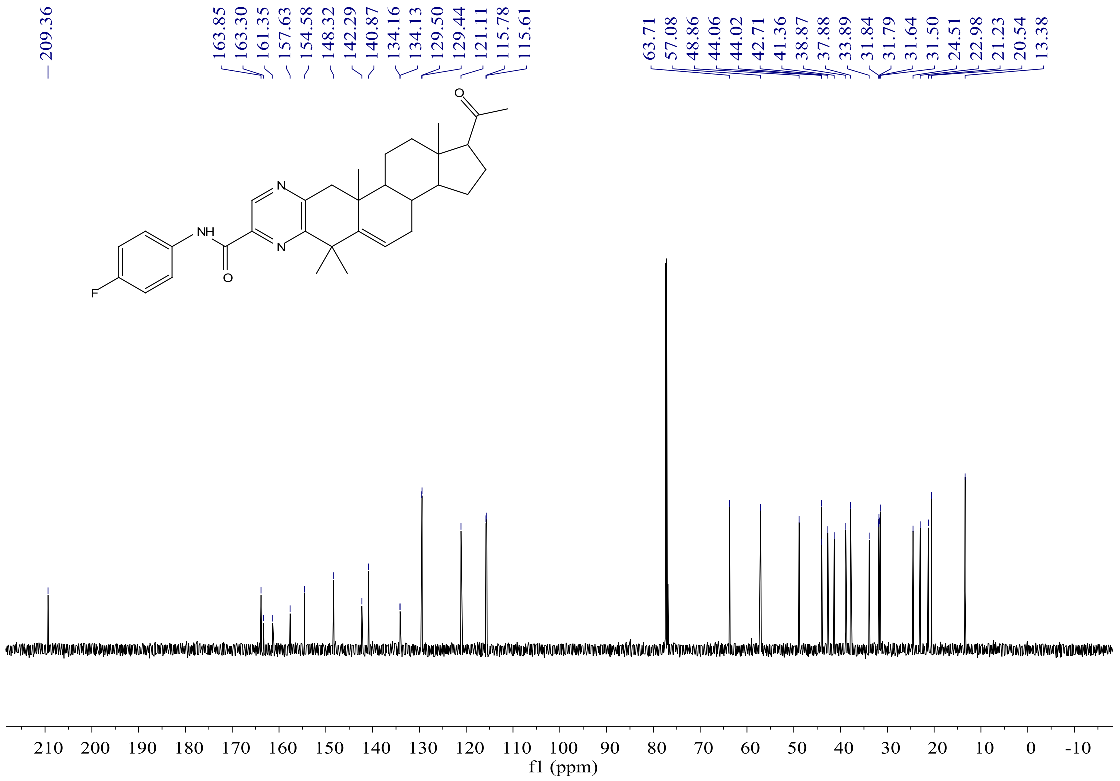

209.36
163.85
163.30
161.35
157.63
154.58
148.32
142.29
140.87
134.16
134.13
129.50
129.44
121.11
115.78
115.61
63.71
57.08
48.86
44.06
44.02
42.71
41.36
38.87
37.88
33.89
31.84
31.79
31.64
31.50
24.51
22.98
21.23
20.54
13.38
N
NH
N
O
F
O
210 200 190 180 170 160 150 140 130 120 110 100 90 80 70 60 50 40 30 20 10 0 -10
f1 (ppm)

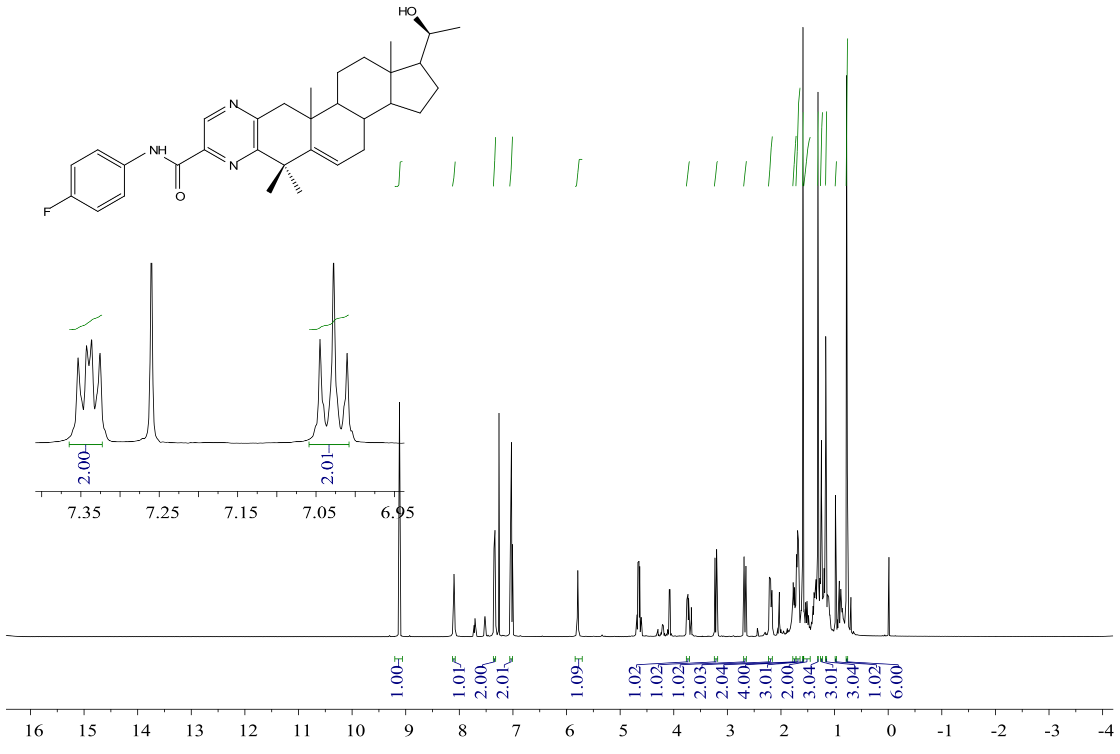

HO
N
NH
N
O
F
2.00
2.01
7.35 7.25 7.15 7.05 6.95
1.00
1.01
2.00
2.01
1.09
1.02
1.02
1.02
2.03
2.04
4.00
3.01
2.00
3.04
3.01
3.04
1.02
6.00
16 15 14 13 12 11 10 9 8 7 6 5 4 3 2 1 0 -1 -2 -3 -4

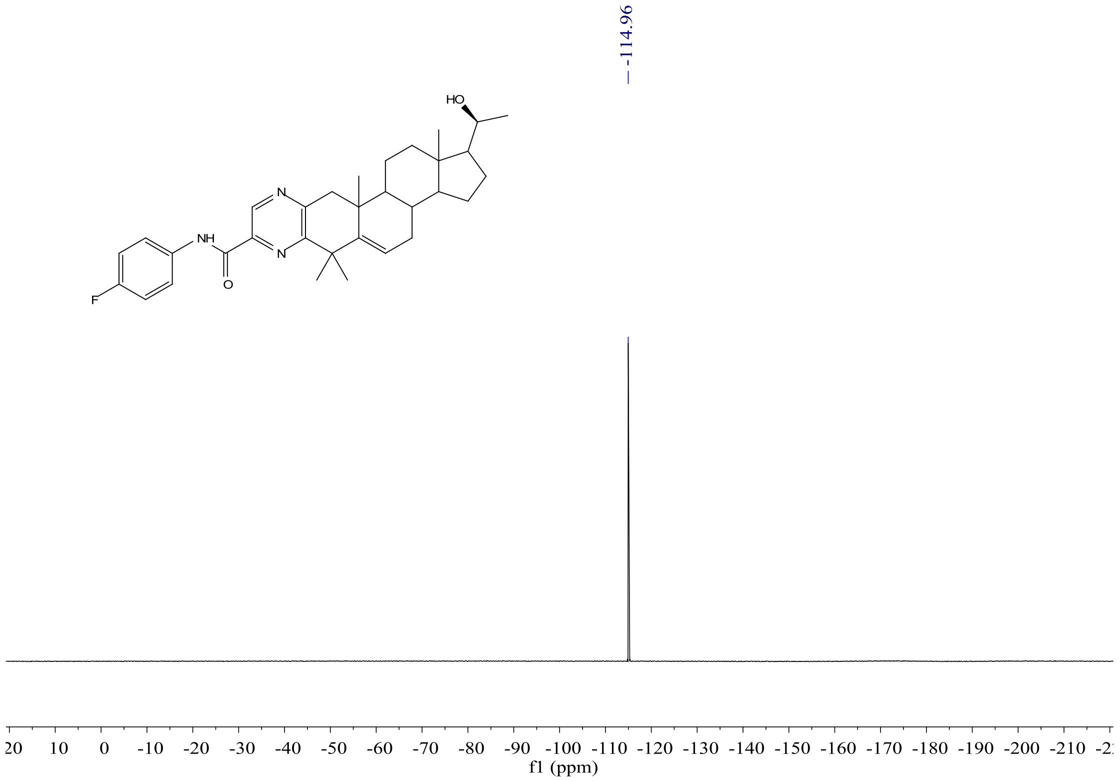

Compound **11h** and **12h**

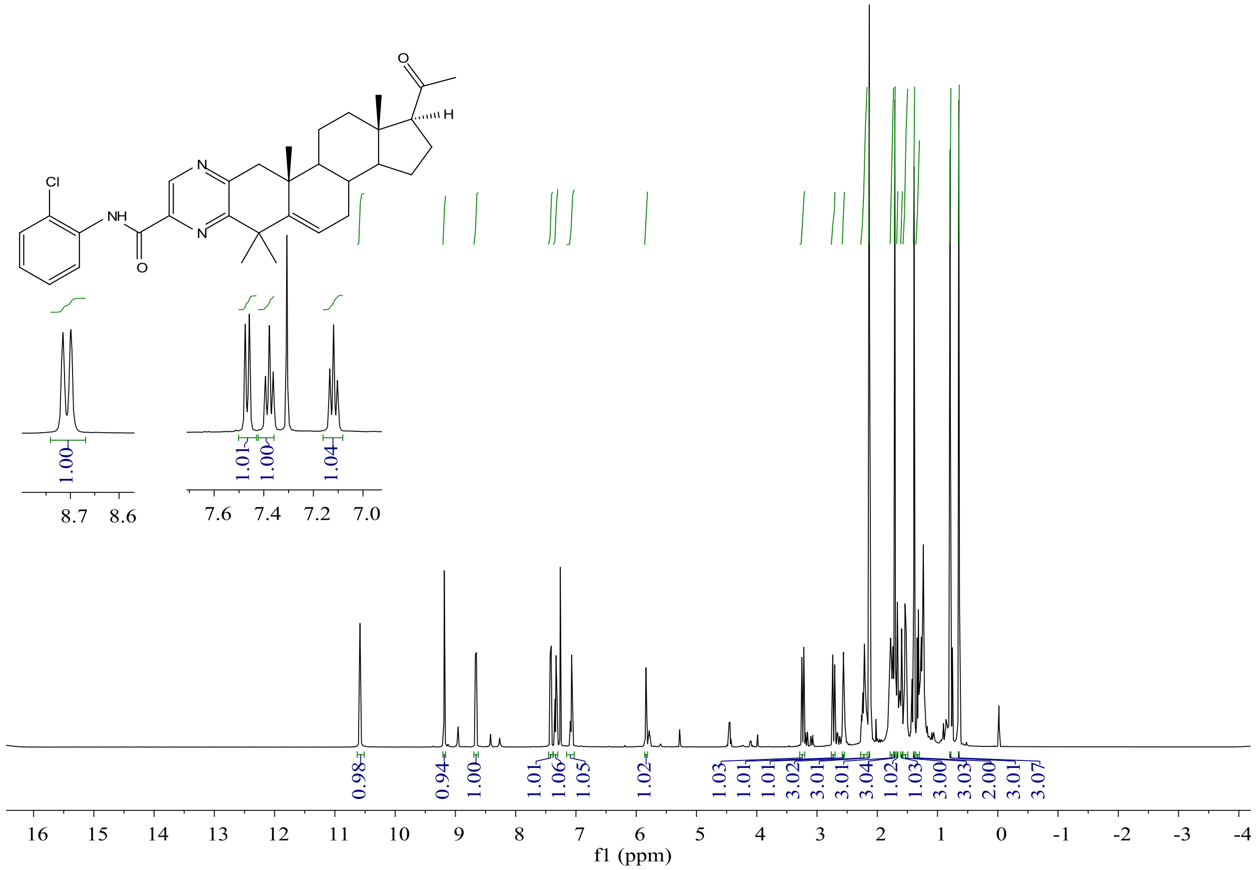

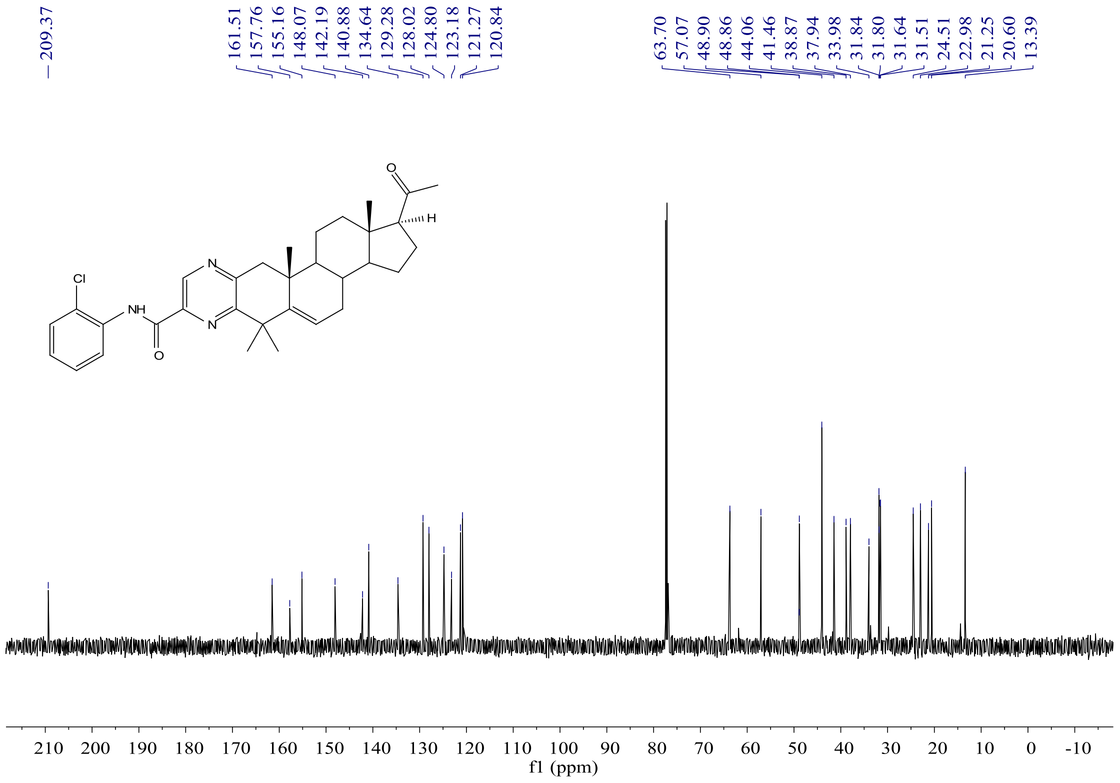

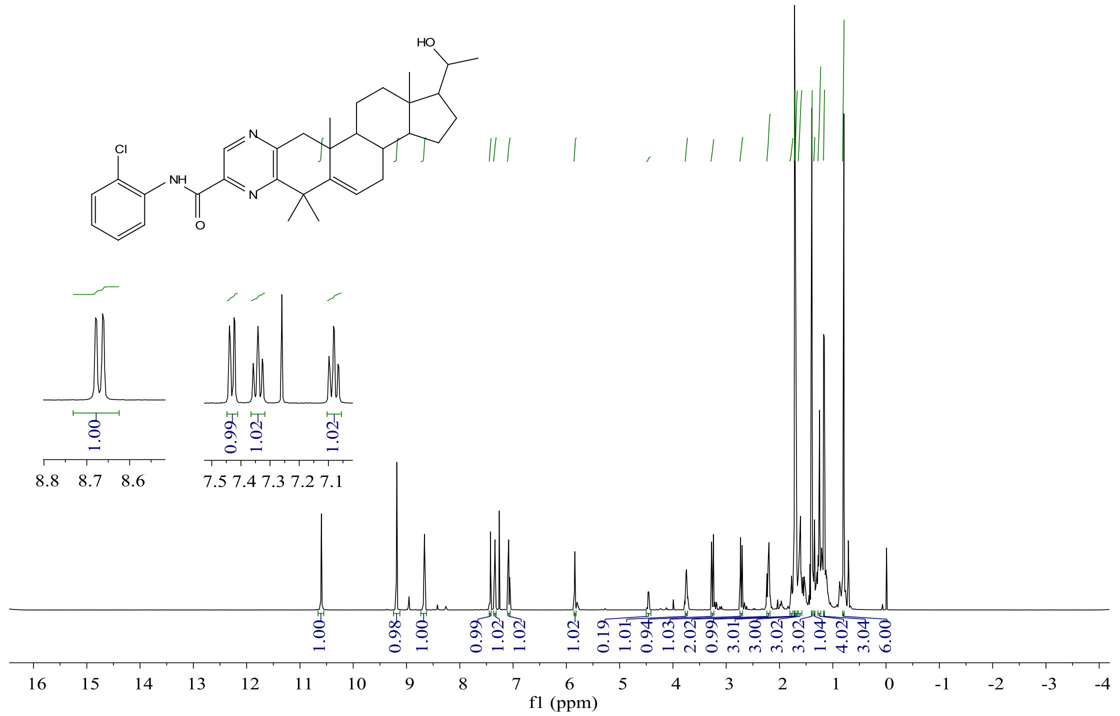

Compound **11i** and **12i**

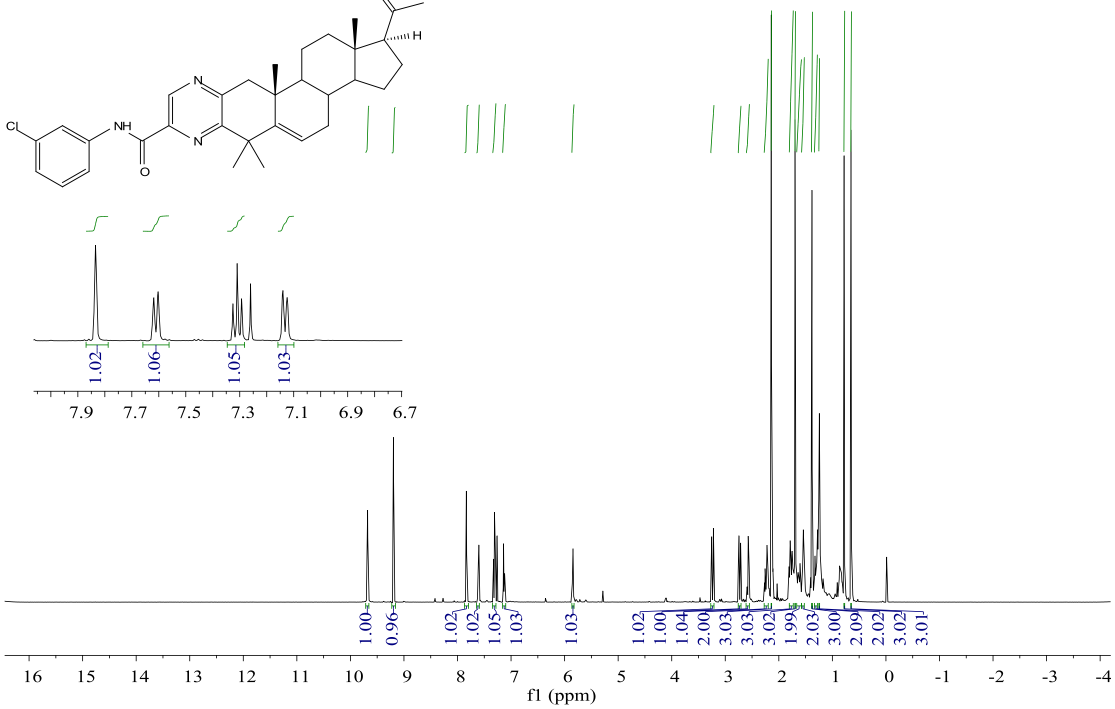
Cl
NH
O
N
N
H
O
1.02
1.06
1.05
1.03
7.9 7.7 7.5 7.3 7.1 6.9 6.7
1.00 0.96 1.02 1.02 1.05 1.03 1.03 1.02 1.00 1.04 2.00 3.03 3.03 3.02 1.99 2.03 3.00 2.09 2.02 3.02 3.01
16 15 14 13 12 11 10 9 8 7 6 5 4 3 2 1 0 -1 -2 -3 -4
f1 (ppm)

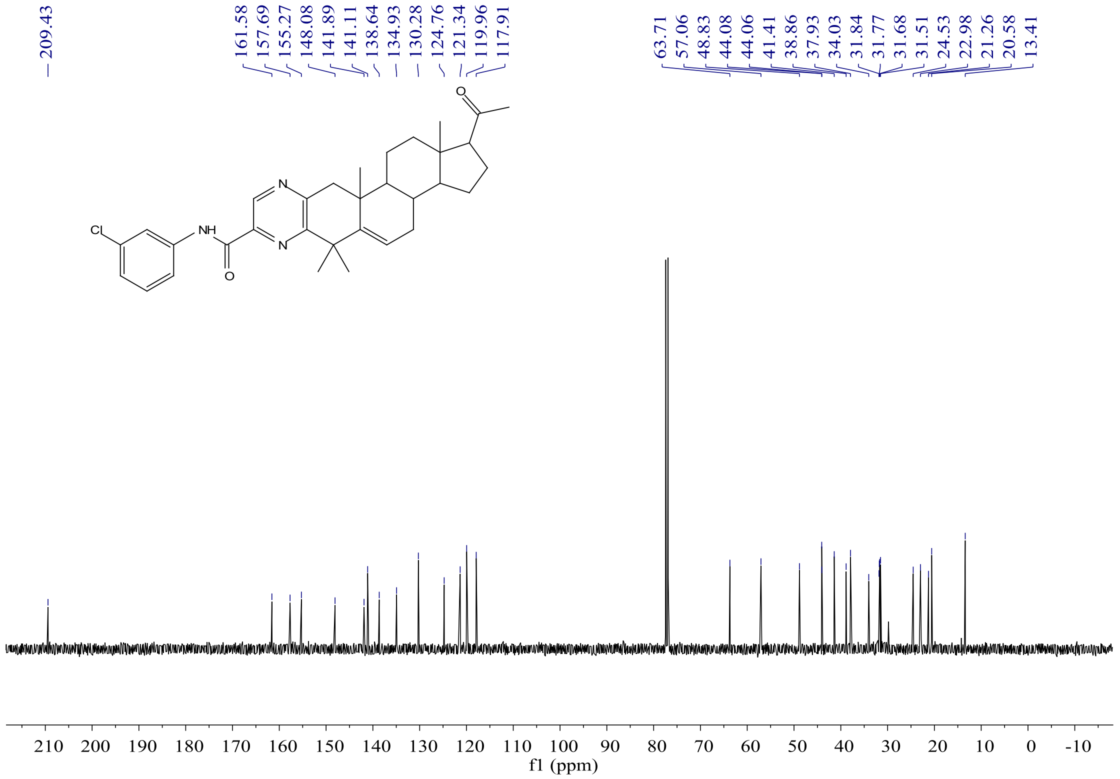
209.43
161.58 157.69 155.27 148.08 141.89 141.11 138.64 134.93 130.28 124.76 121.34 119.96 117.91
63.71 57.06 48.83 44.08 44.06 41.41 38.86 37.93 34.03 31.84 31.77 31.68 31.51 24.53 22.98 21.26 20.58 13.41
Cl
NH
O
N
N
O
210 200 190 180 170 160 150 140 130 120 110 100 90 80 70 60 50 40 30 20 10 0 -10
f1 (ppm)

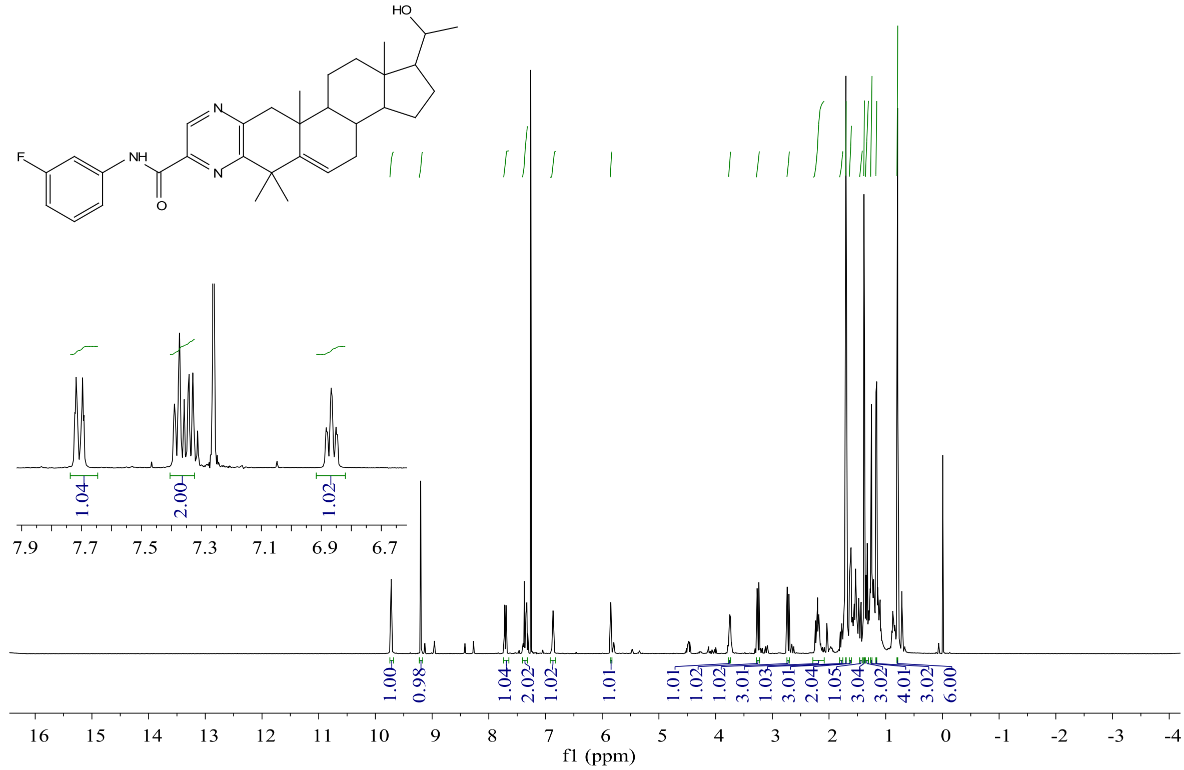

Compound **11j** and **12j**

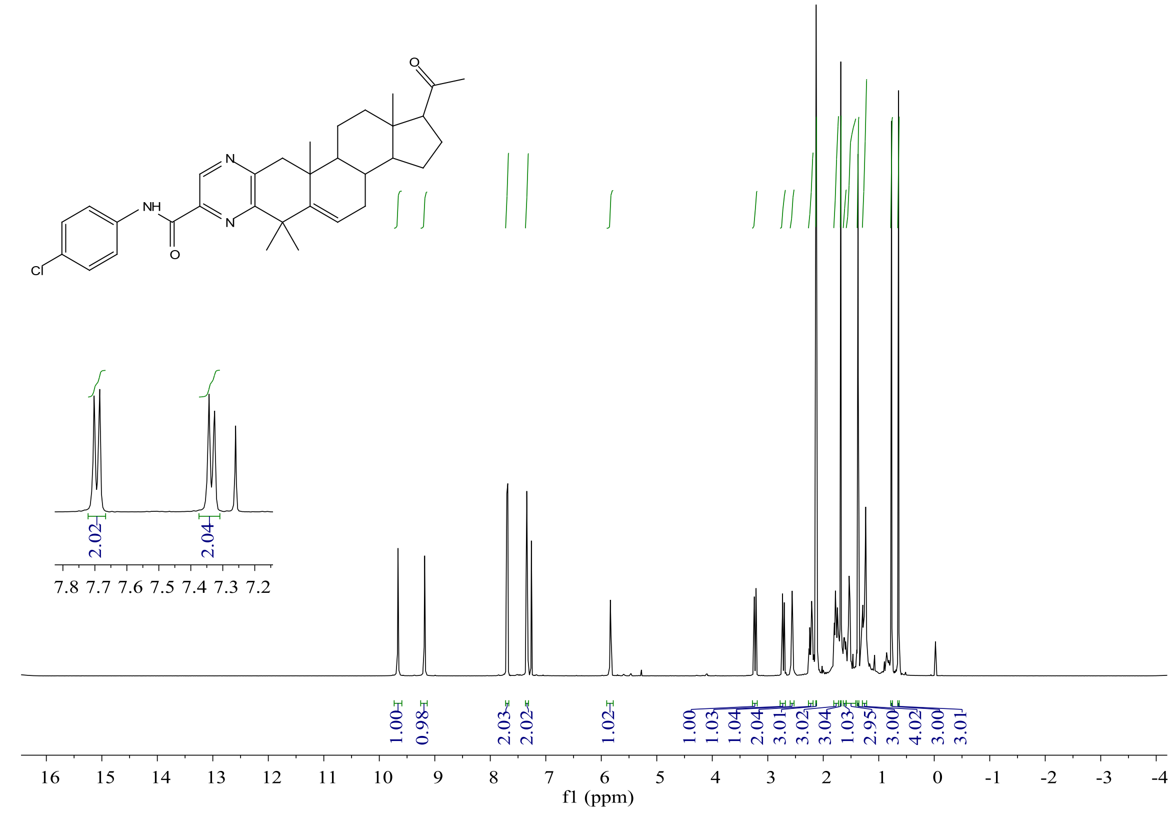

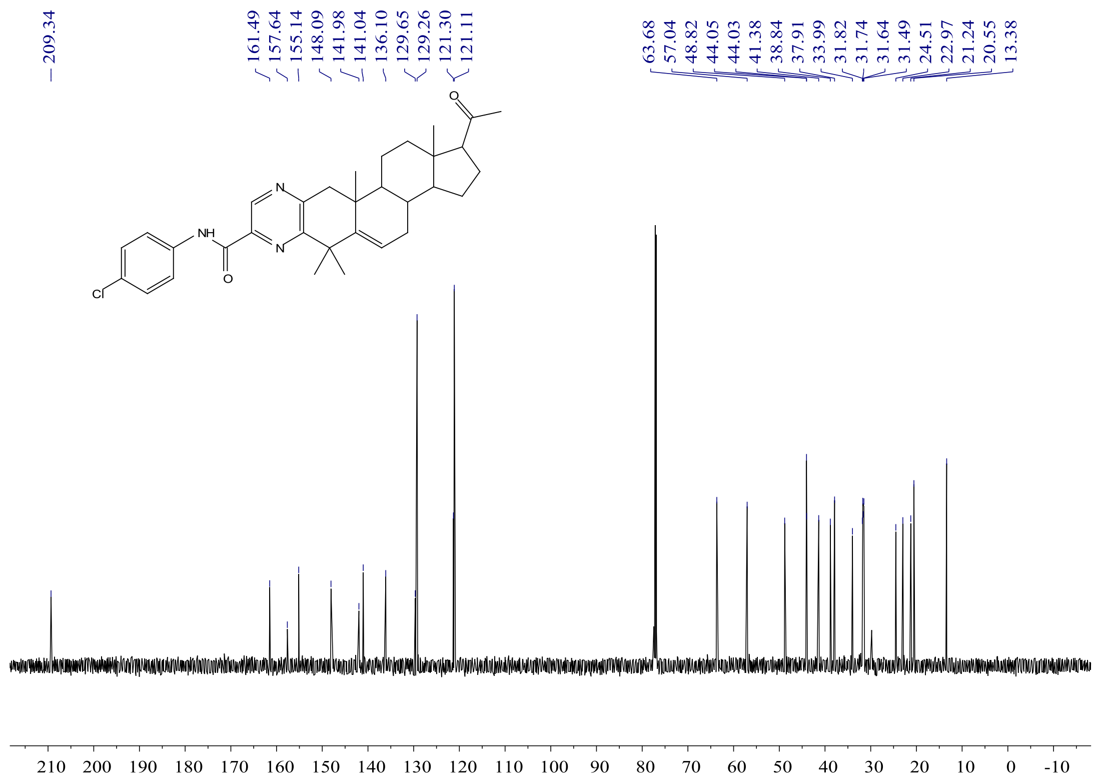

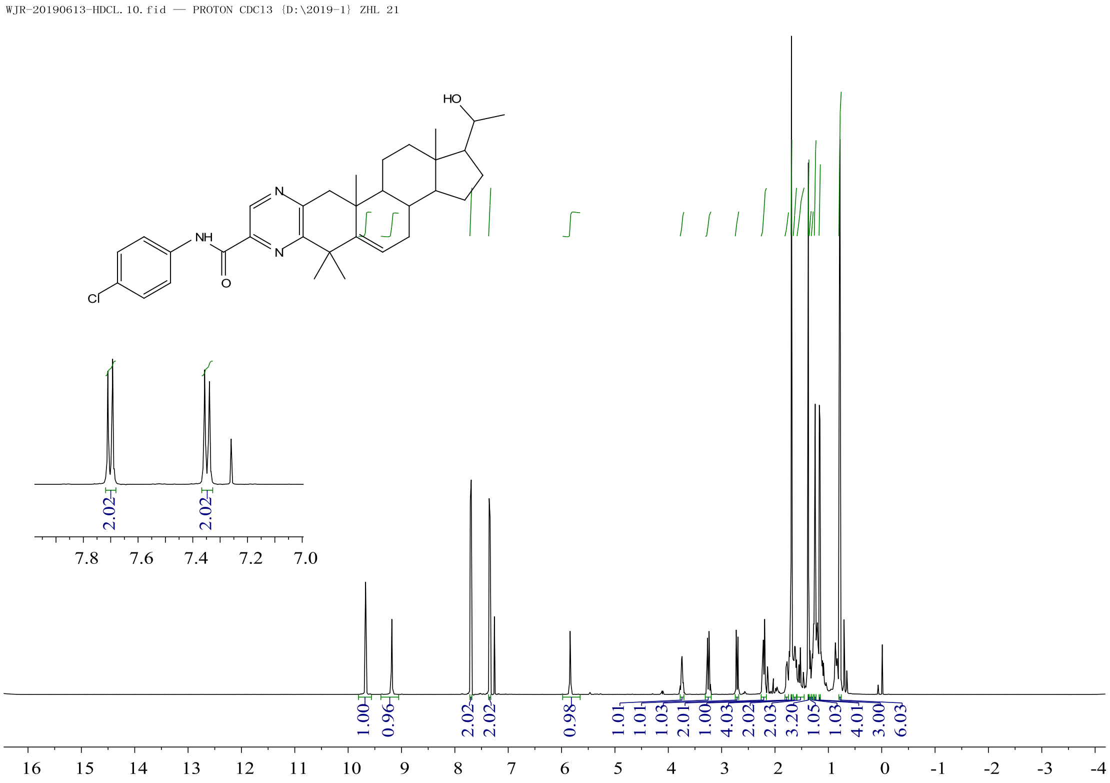

Compound **11k** and **12k**

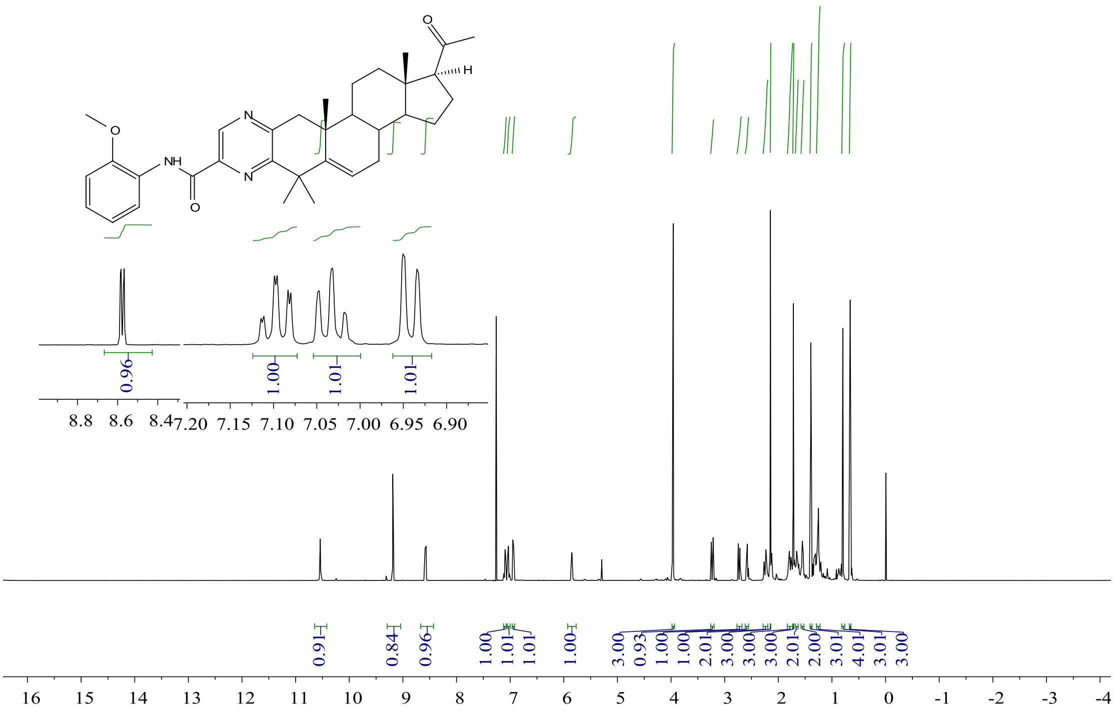

O
NH
N
N
H
O
0.96
1.00
1.01
1.01
8.8 8.6 8.4 7.20 7.15 7.10 7.05 7.00 6.95 6.90
0.91
0.84
0.96
1.00
1.01
1.01
1.00
3.00
0.93
1.00
1.00
2.01
3.00
3.00
3.00
2.01
2.00
3.01
4.01
3.01
3.00
16 15 14 13 12 11 10 9 8 7 6 5 4 3 2 1 0 -1 -2 -3 -4

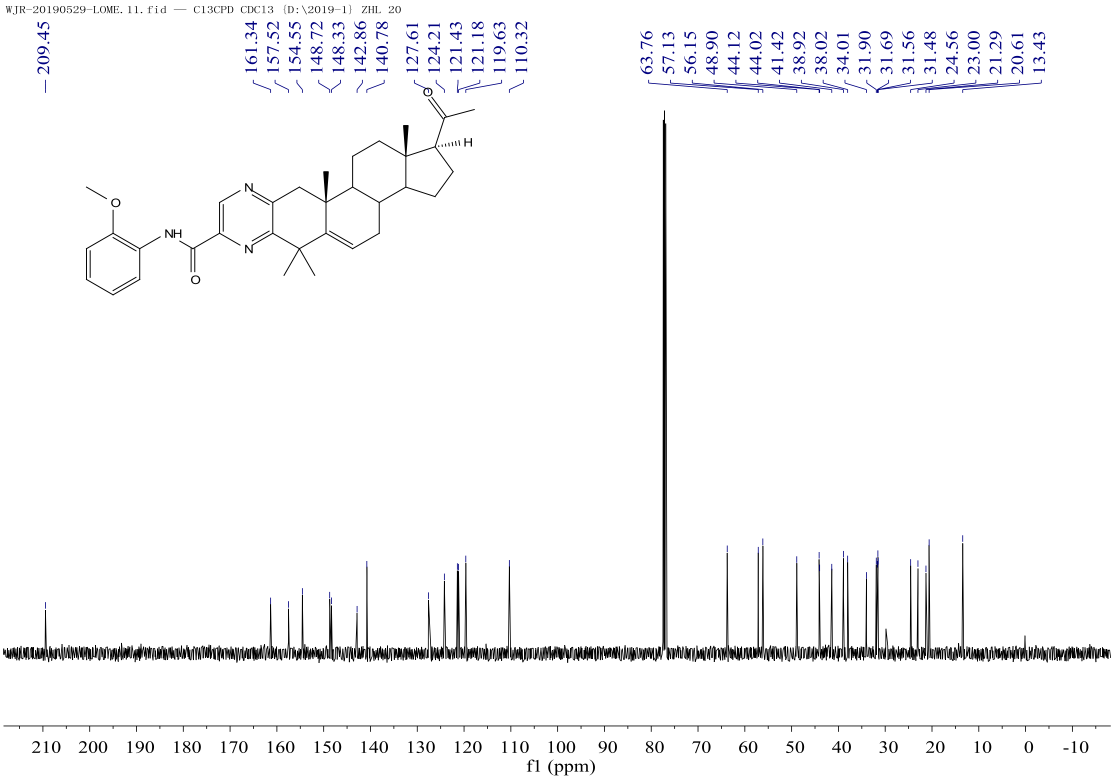

WJR-20190529-LOME.11.fid — C13CPD CDCl3 {D:\2019-1} ZHL 20
209.45
161.34
157.52
154.55
148.72
148.33
142.86
140.78
127.61
124.21
121.43
121.18
119.63
110.32
63.76
57.13
56.15
48.90
44.12
44.02
41.42
38.92
38.02
34.01
31.90
31.69
31.56
31.48
24.56
23.00
21.29
20.61
13.43
O
NH
N
N
H
O
210 200 190 180 170 160 150 140 130 120 110 100 90 80 70 60 50 40 30 20 10 0 -10
f1 (ppm)

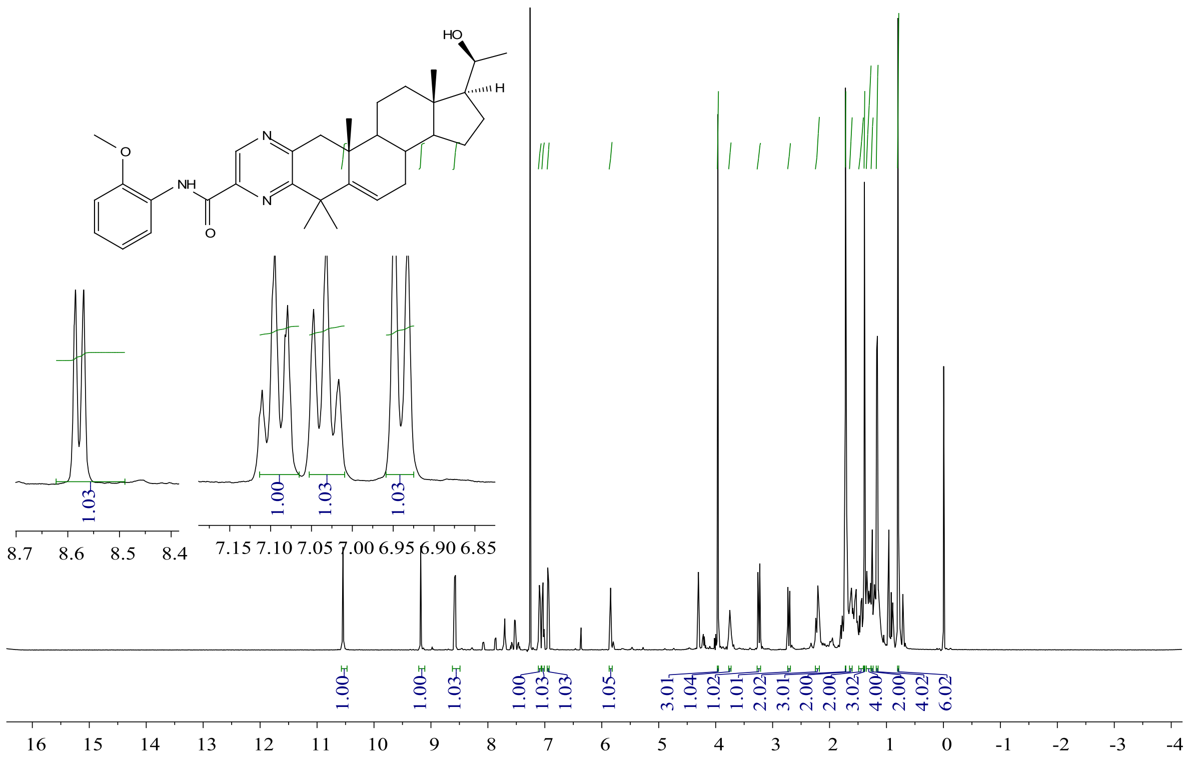

Compound **11l** and **12l**

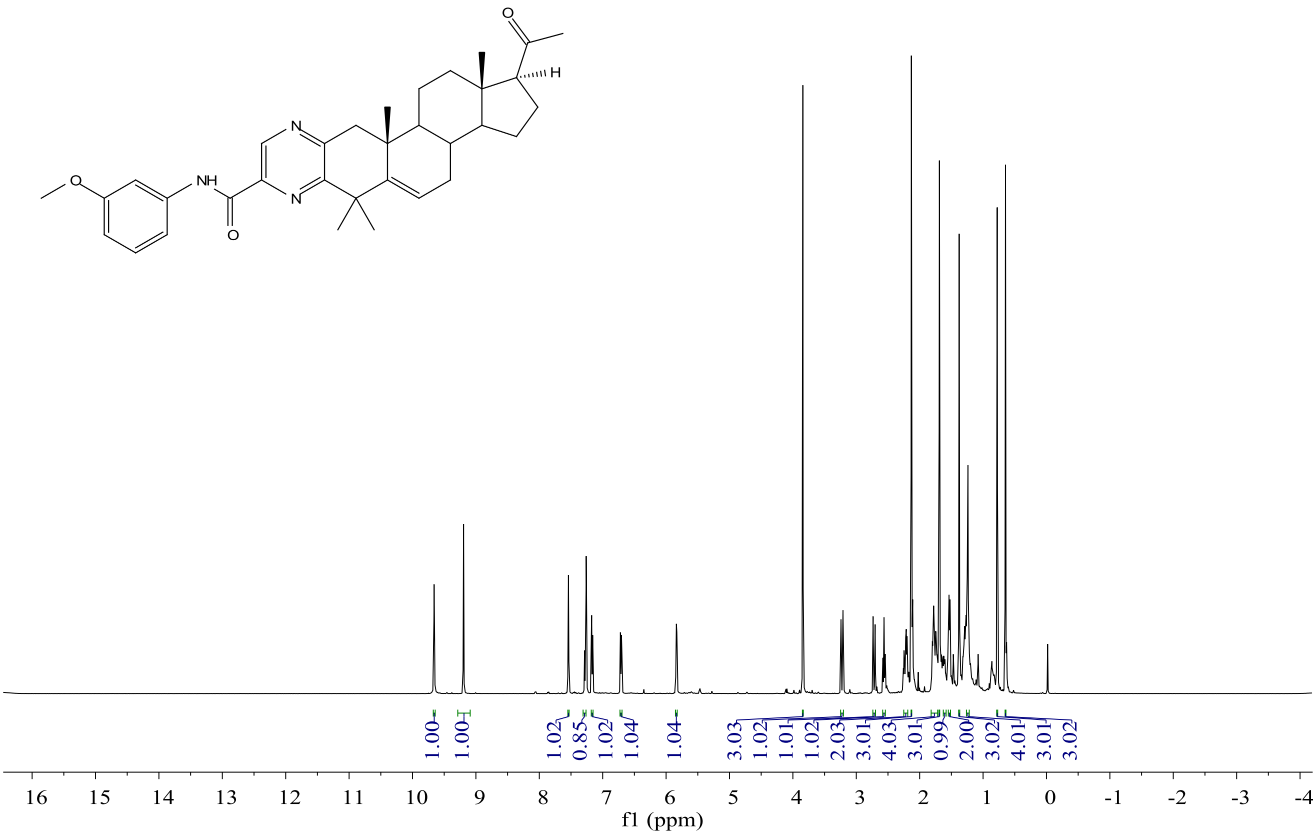

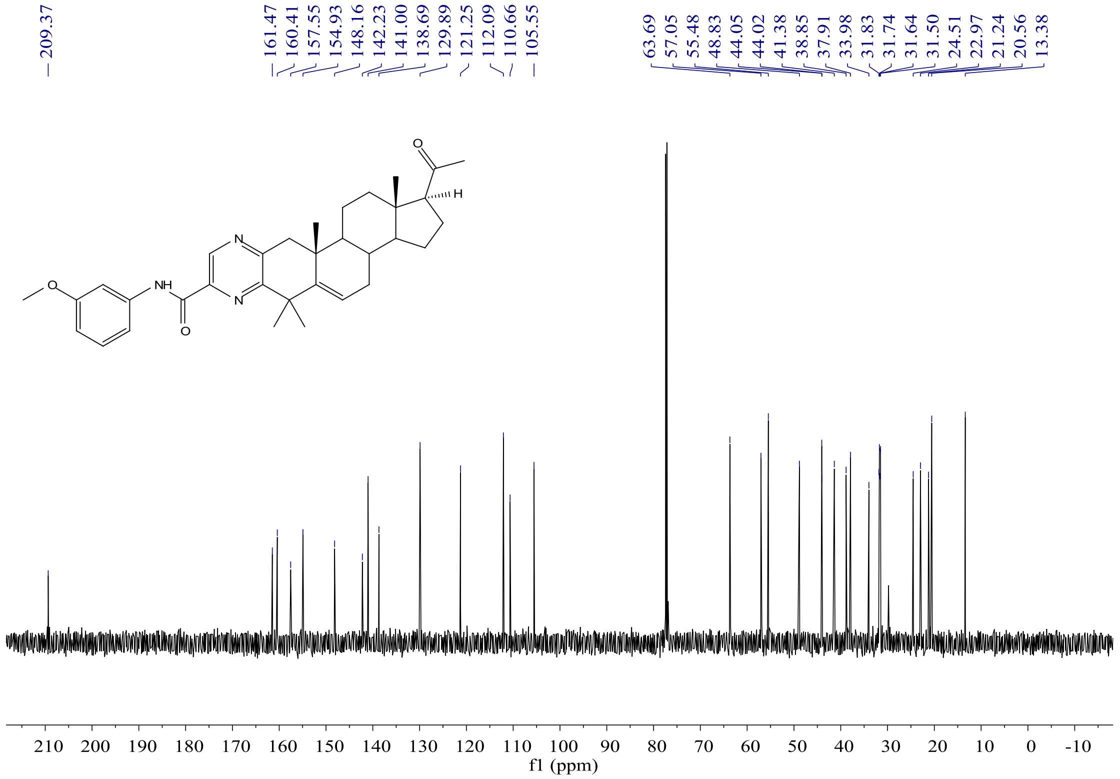
209.37
161.47
160.41
157.55
154.93
148.16
142.23
141.00
138.69
129.89
121.25
112.09
110.66
105.55
63.69
57.05
55.48
48.83
44.05
44.02
41.38
38.85
37.91
33.98
31.83
31.74
31.64
31.50
24.51
22.97
21.24
20.56
13.38
O
H
N
N
NH
O
O
210 200 190 180 170 160 150 140 130 120 110 100 90 80 70 60 50 40 30 20 10 0 -10
f1 (ppm)

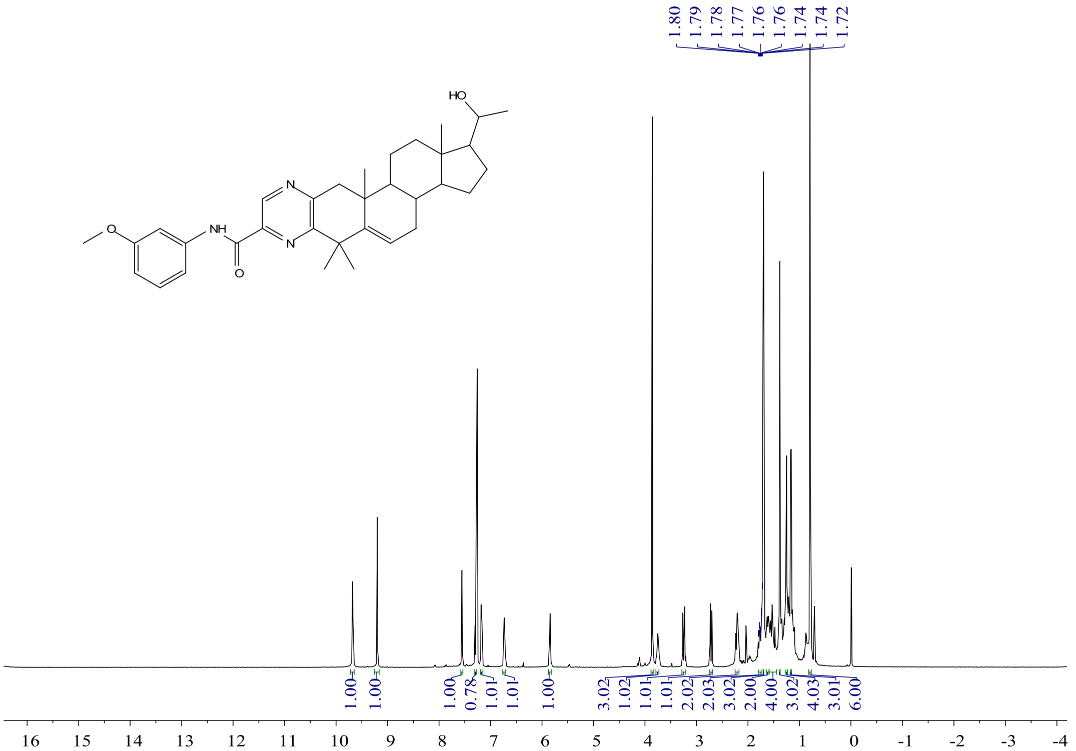
1.80
1.79
1.78
1.77
1.76
1.76
1.74
1.74
1.72
HO
N
N
NH
O
O
1.00
1.00
1.00
0.78
1.01
1.01
1.00
3.02
1.02
1.01
1.01
2.02
2.03
3.02
2.00
4.00
3.02
4.03
3.01
6.00
16 15 14 13 12 11 10 9 8 7 6 5 4 3 2 1 0 -1 -2 -3 -4

Compound **11m** and **12m**

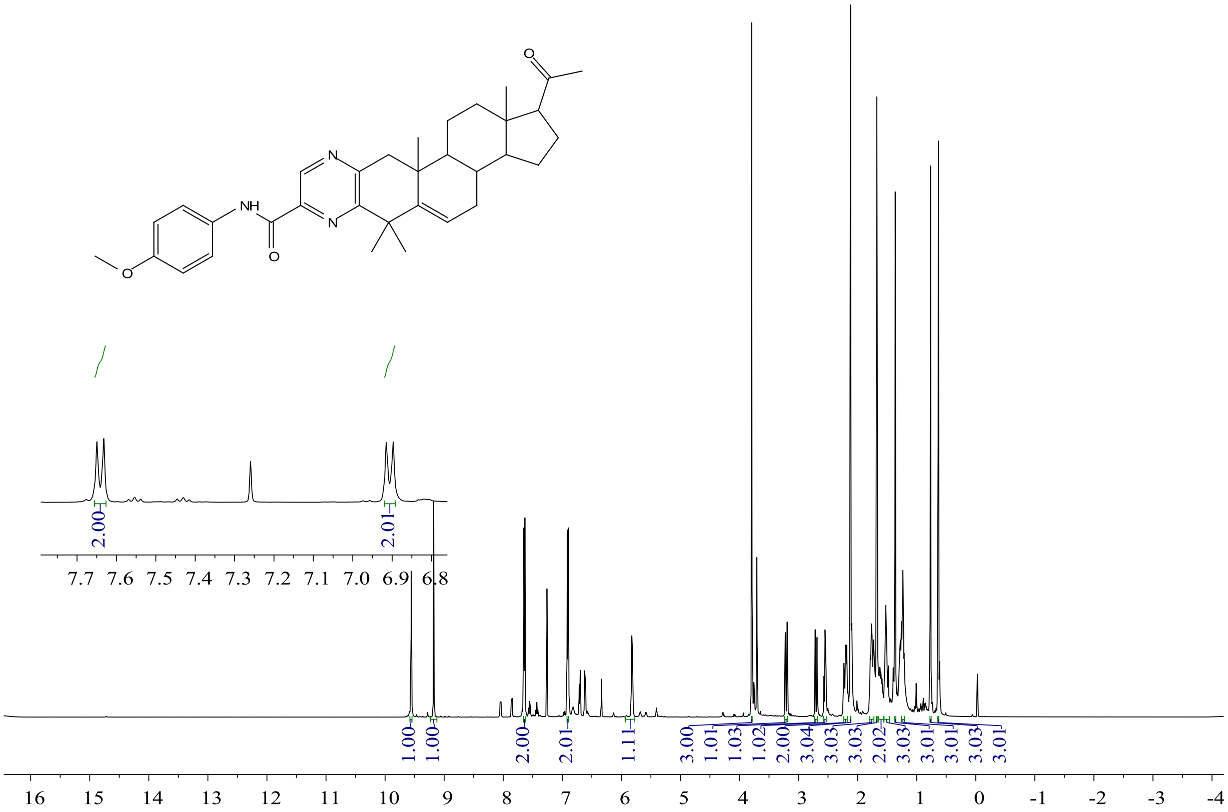

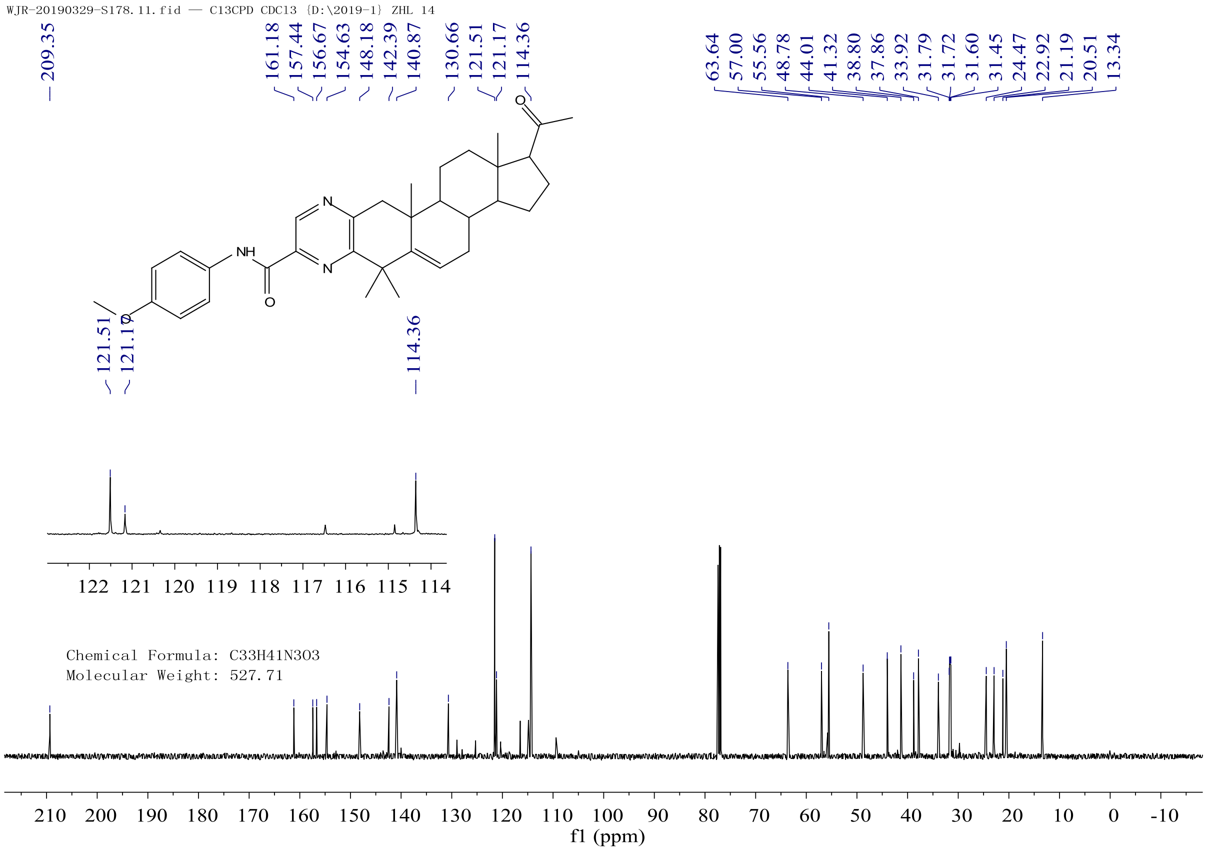

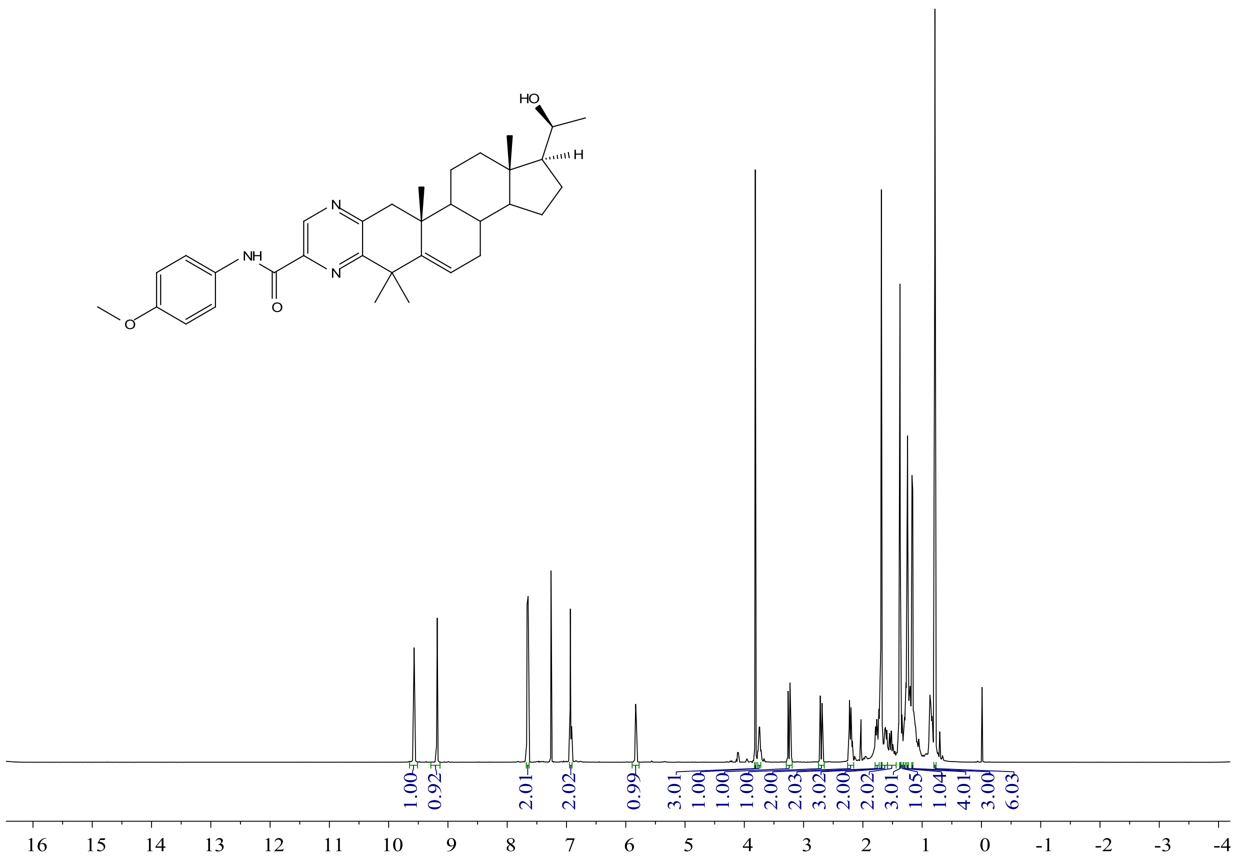

Compound **11n** and **12n**

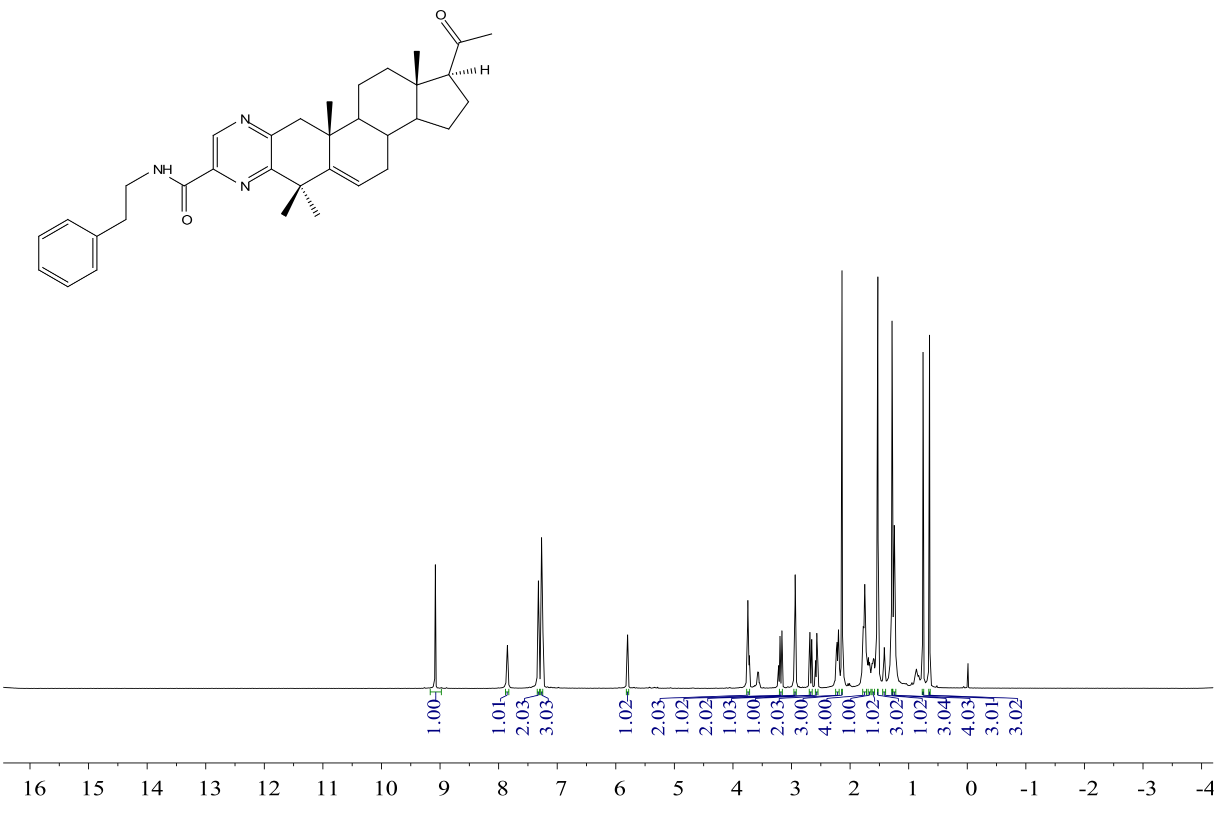

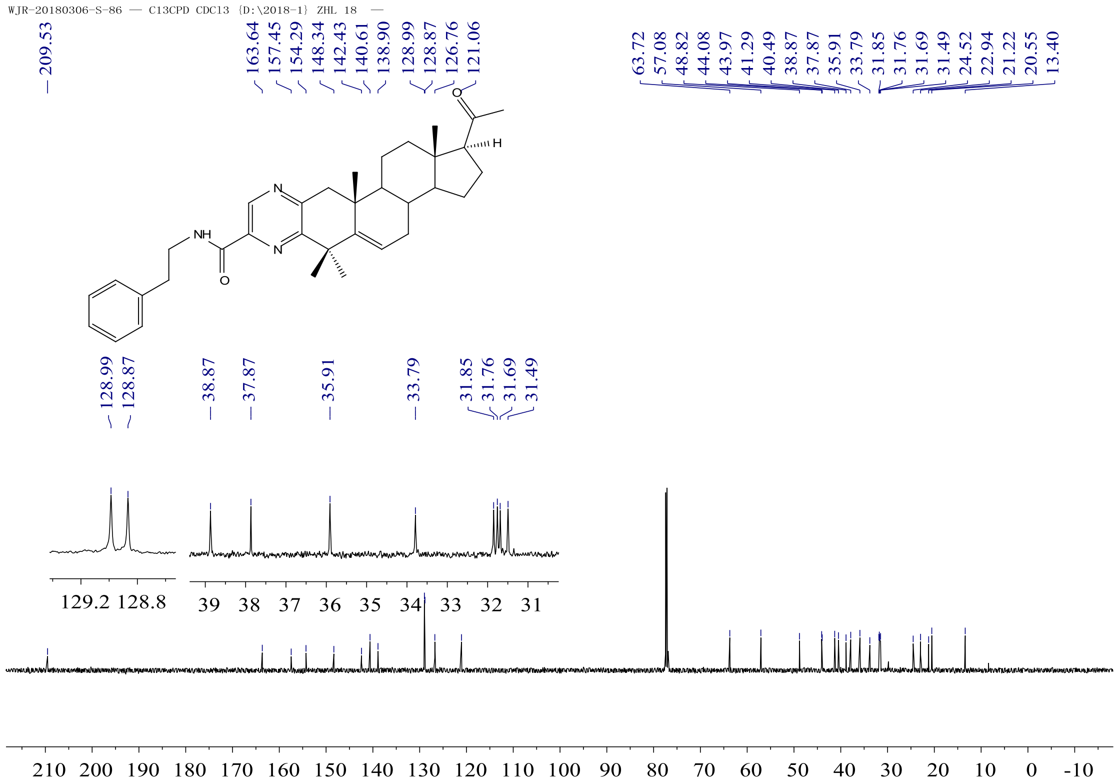
WJR-20180306-S-86 — C13CPD CDCl3 {D:\2018-1} ZHL 18 —
209.53
163.64
157.45
154.29
148.34
142.43
140.61
138.90
128.99
128.87
126.76
121.06
63.72
57.08
48.82
44.08
43.97
41.29
40.49
38.87
37.87
35.91
33.79
31.85
31.76
31.69
31.49
24.52
22.94
21.22
20.55
13.40
129.2 128.8
39 38 37 36 35 34 33 32 31
210 200 190 180 170 160 150 140 130 120 110 100 90 80 70 60 50 40 30 20 10 0 -10

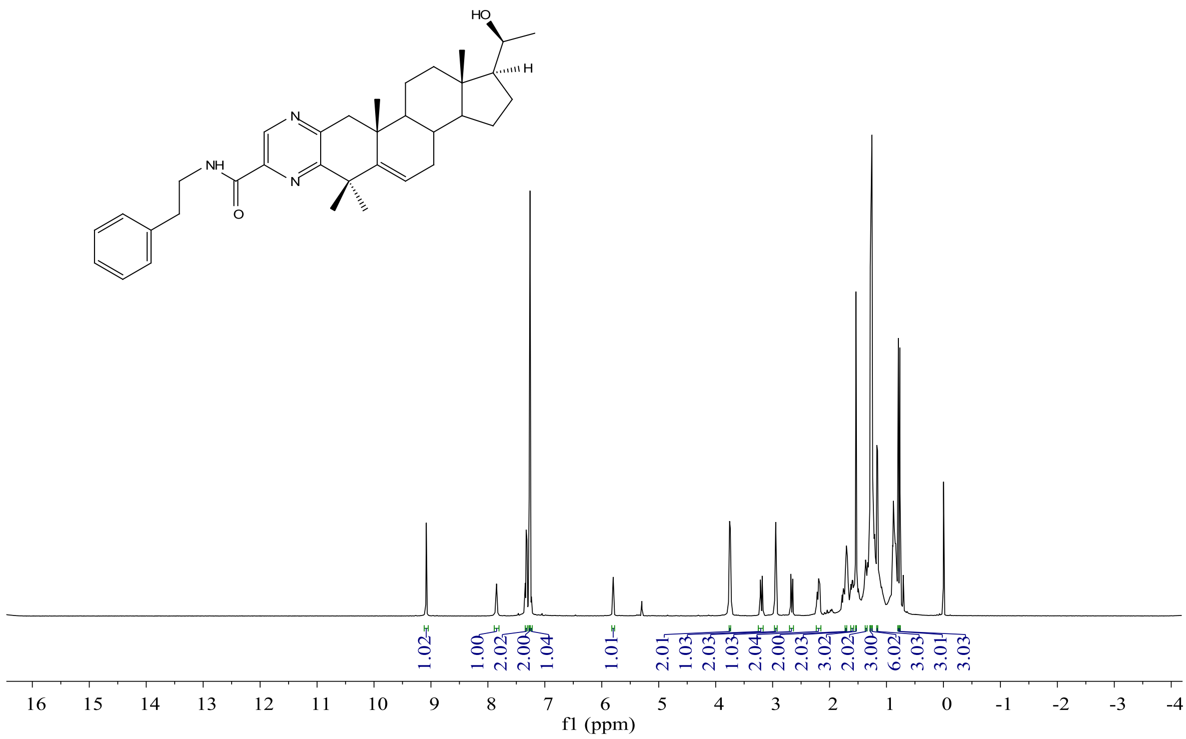
HO
H
N
NH
O
1.02
1.00
2.02
2.00
1.04
1.01
2.01
1.03
2.03
1.03
2.04
2.00
2.03
3.02
2.02
3.00
6.02
3.03
3.01
3.03
16 15 14 13 12 11 10 9 8 7 6 5 4 3 2 1 0 -1 -2 -3 -4
f1 (ppm)

Compound **11o** and **12o**

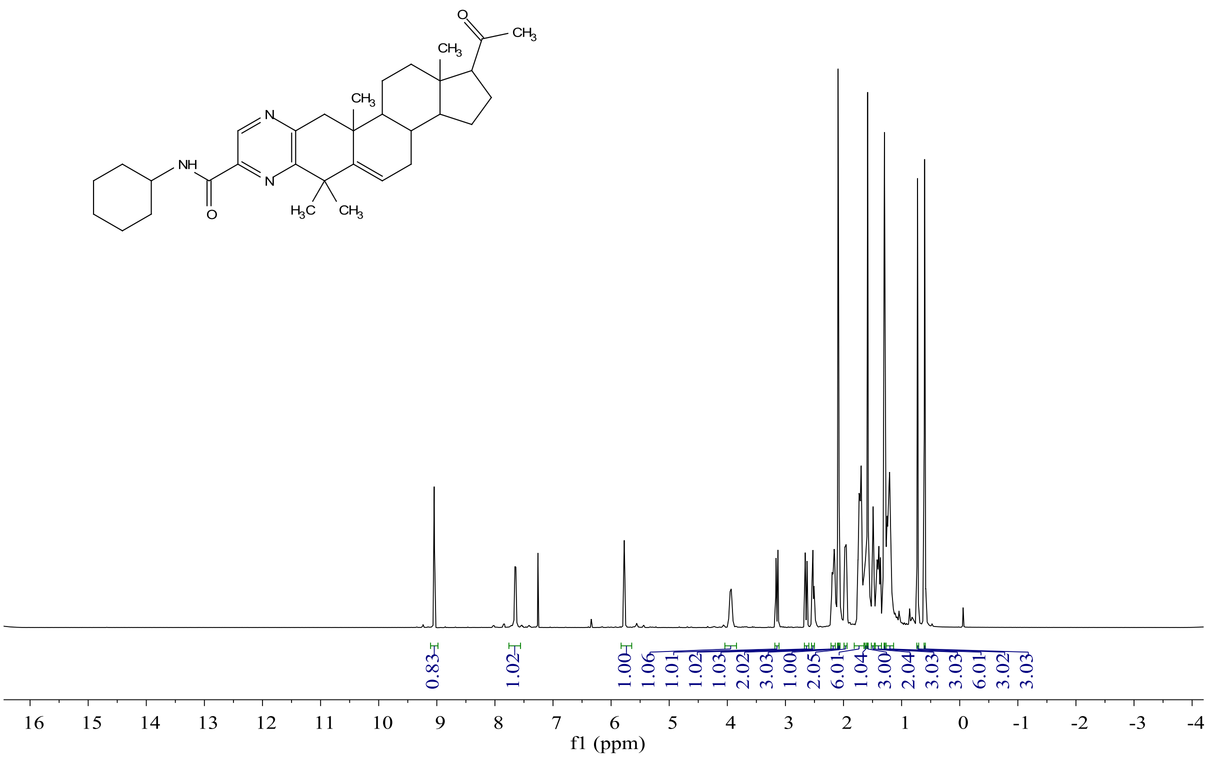

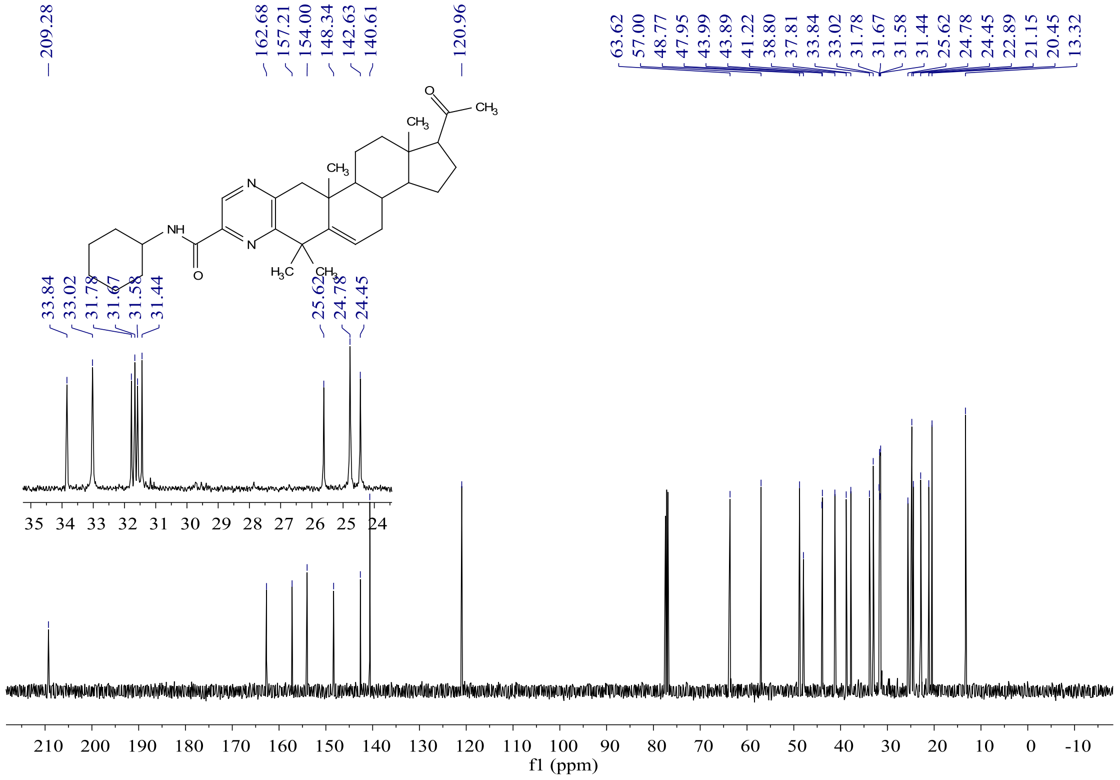

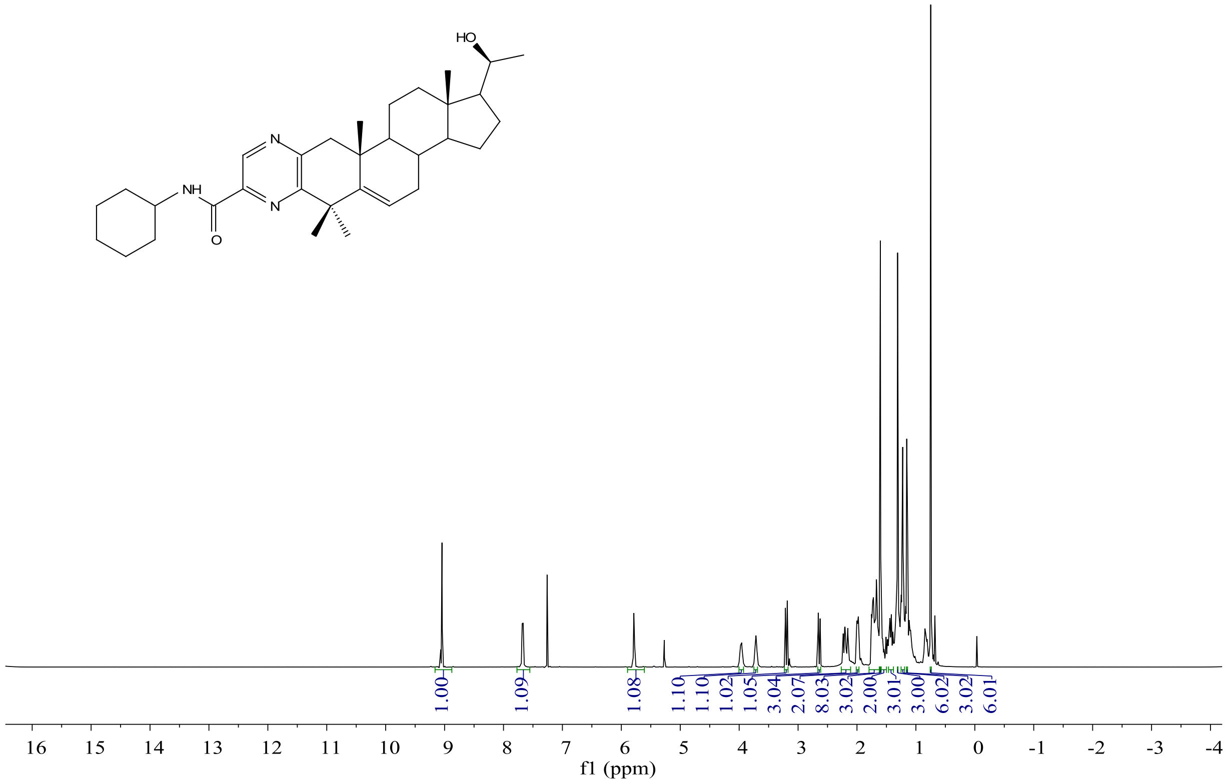

4. The HRMS(ESI+) Spectra of Some Representative Compounds

Compound **5a**

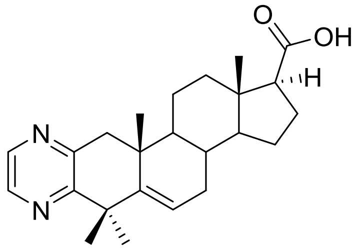

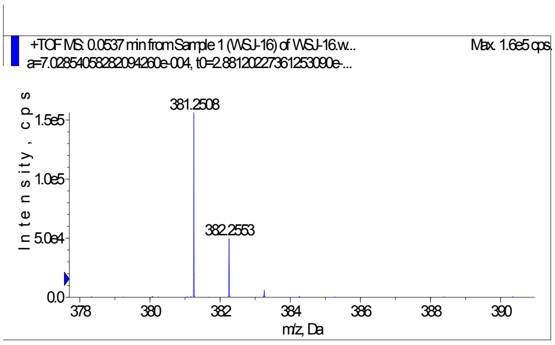

calcd for $[M+H]^+$ 381.2536, found, 381.2538

Compound **5b**

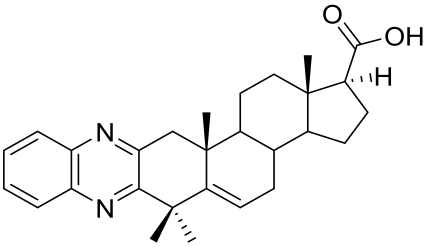

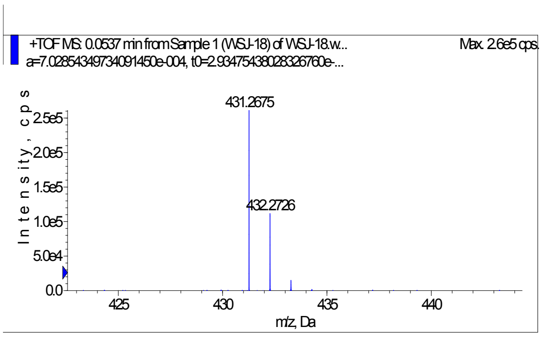

calcd for $[M+H]^+$ 431.2693, found:431.2675

Compound **5c**

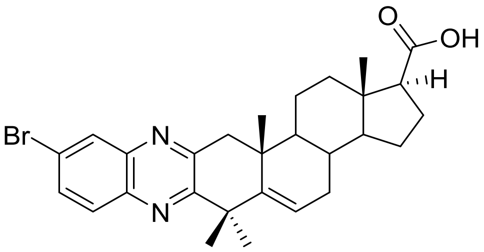

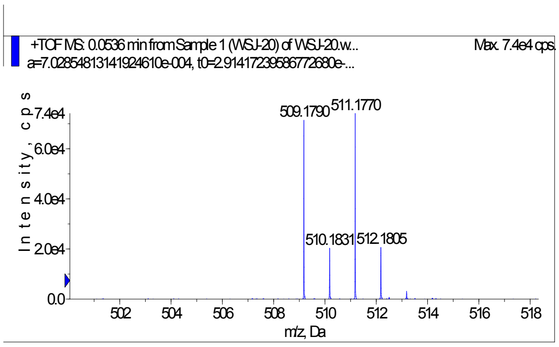

calcd for $[M+H]^+$ 509.1798, found 509.1790

Compound **5e**

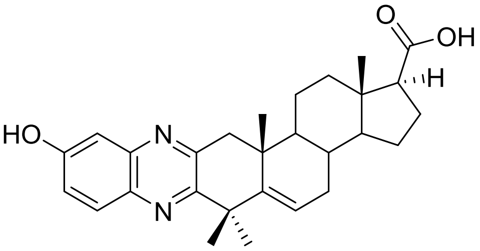

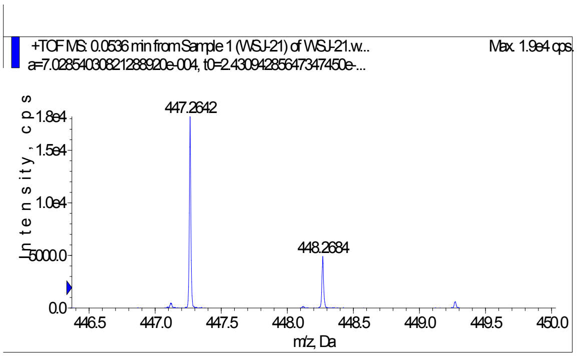

calcd for $[M+H]^+$ 447.2642, found:447.2642

Compound **10a**

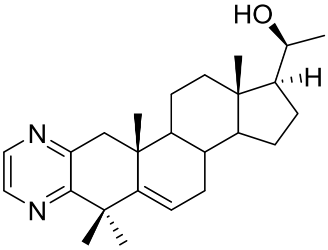

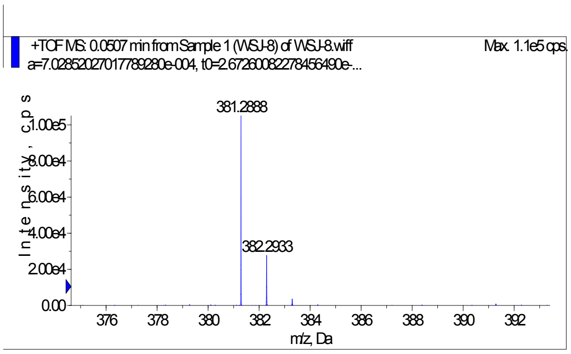

calcd for $[M+H]^+$ 381.2900, found:381.2888

Compound **10b**

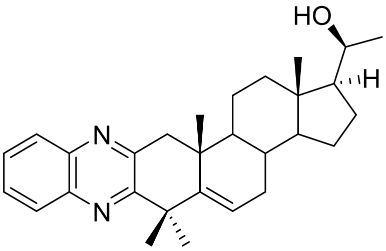

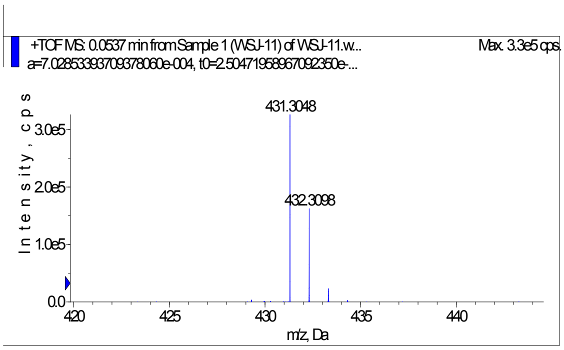

calcd for $[M+H]^+$431.3057, found:431.3048

Compound **10c**

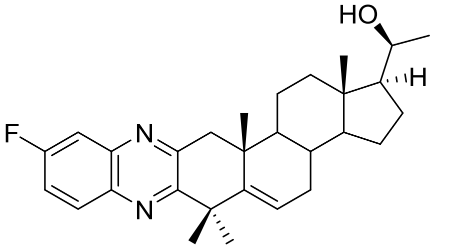

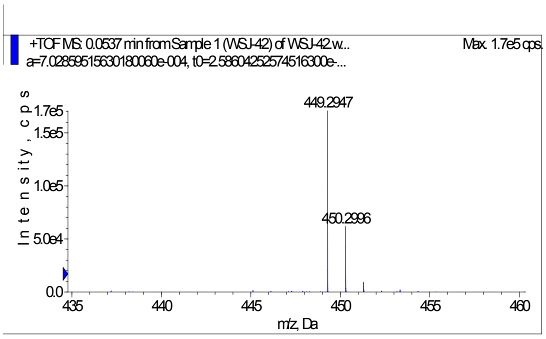

calcd for [M+H]+ 449.2963, found:449.2947

Compound **10e**

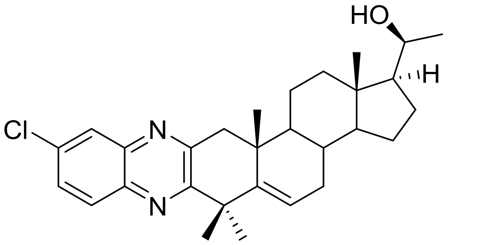

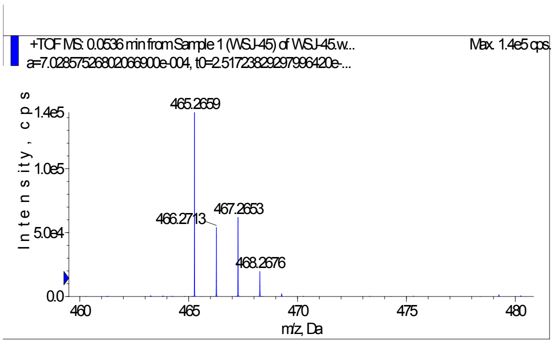

calcd for $[M+H]^{+}$ 465.2667, found:465.2659

Compound **10g**

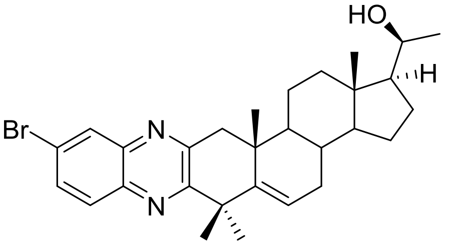

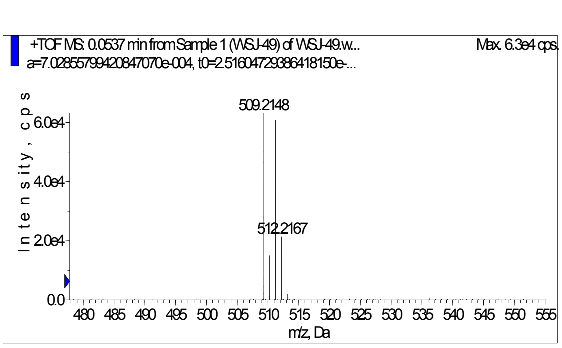

calcd for $[M+H]^{+}$ 509.2162, found:509.2148

Compound **10i**

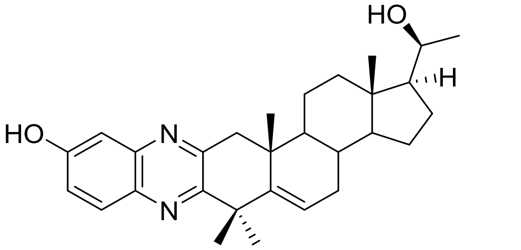

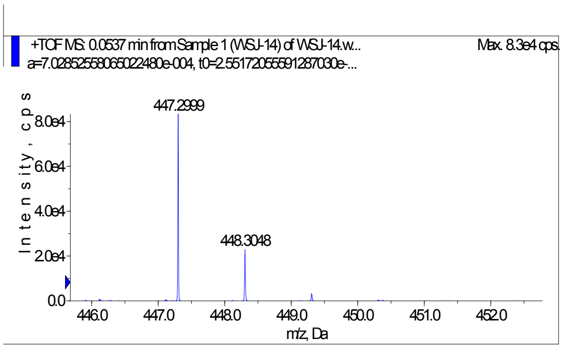

calcd for $[M+H]^{+}$ 447.3006, found:447.2999

Compound **10k**

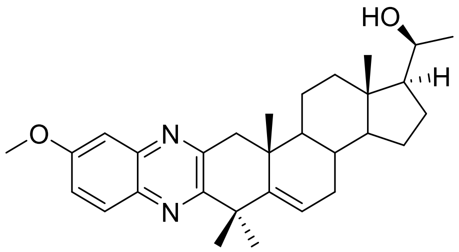

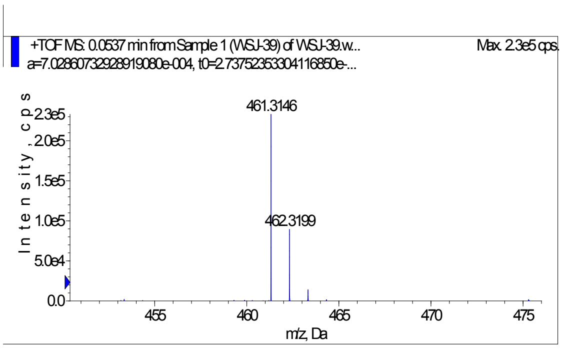

calcd for $[M+H]^+$ 461.3163, found:461.3146

Compound **10m**

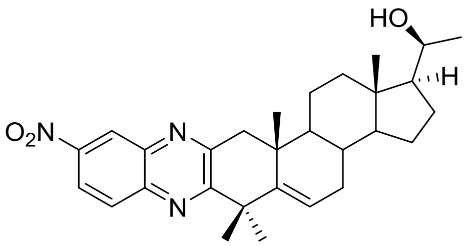

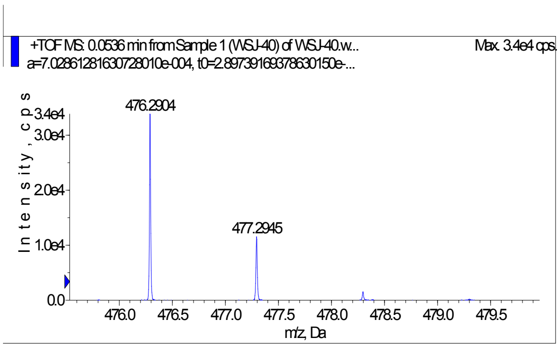

calcd for $[M+H]^+$ 476.2908, found:476.2904

Compound **10o**

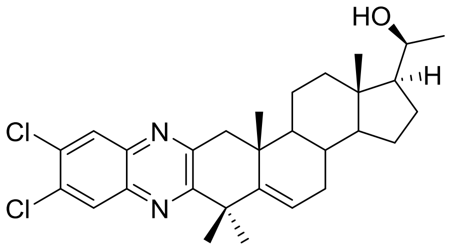

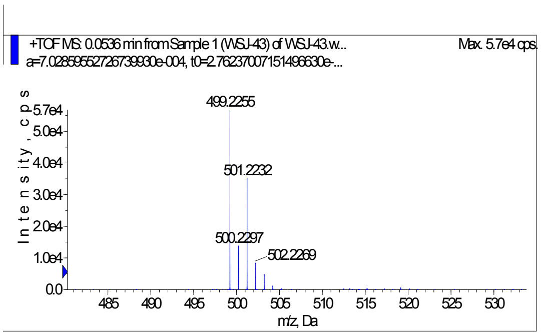

calcd for $[M+H]^+$ 499.2278, found:499.2255

Compound **12a**

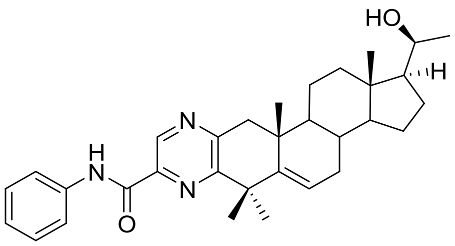

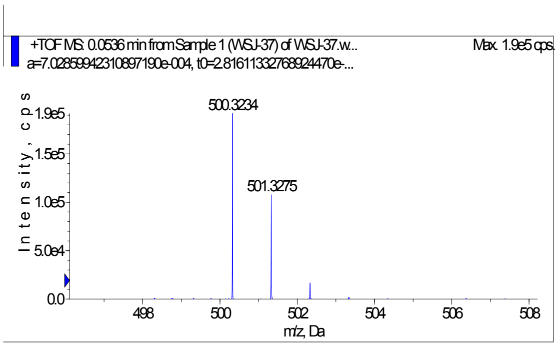

calcd for $[M+H]^+$ 500.3272, found:500.3234

Compound **12b**

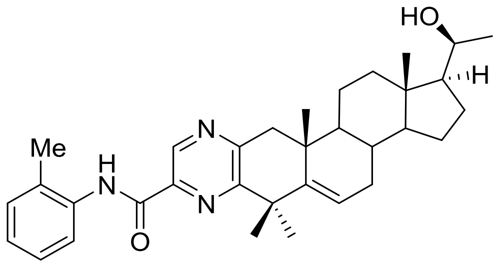

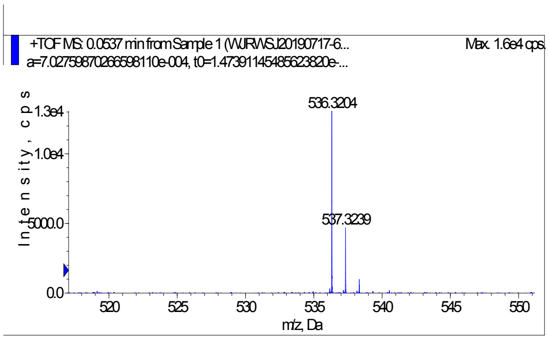

calcd for [M+Na]+ 536.3248, found: 536.3204

Compound **12e**

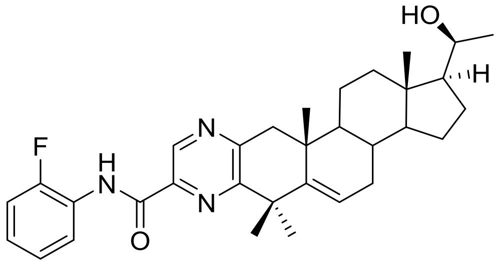

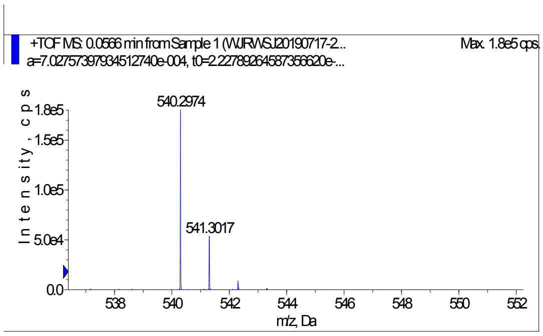

calcd for [M+Na]$^{+}$ 540.2997, found: 540.2974

Compound **12h**

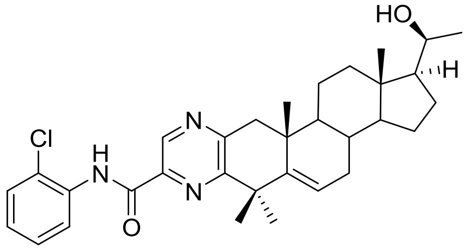

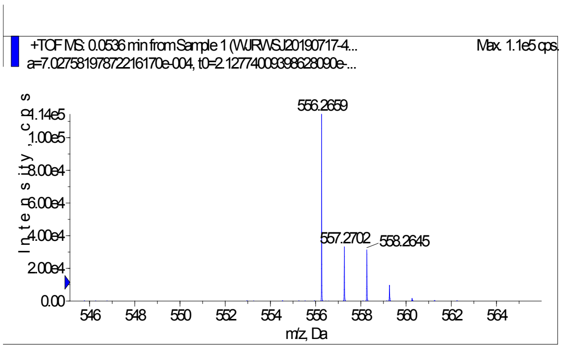

calcd for [M+Na]$^{+}$ 556.2701, found:556.2659

Compound **12k**

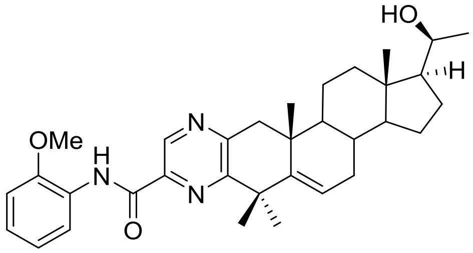

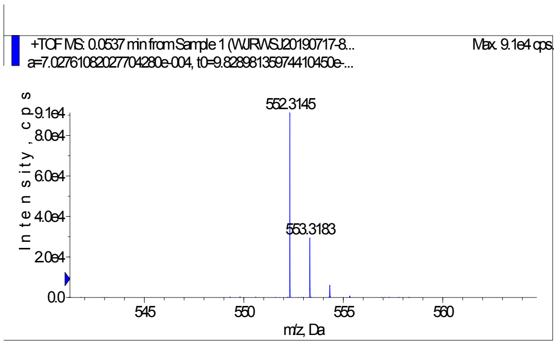

calcd for [M+Na]$^{+}$ 552.3197, found: 552.3145

Compound **12n**

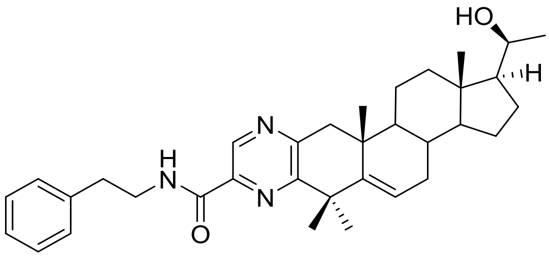

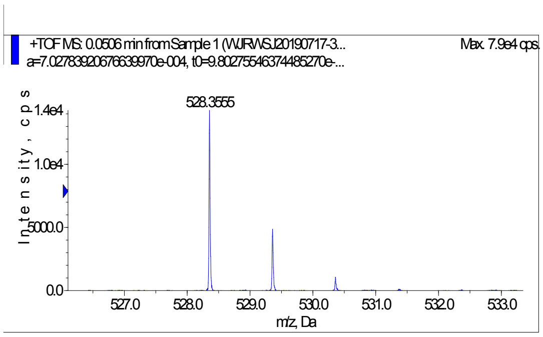

calcd for $[M+H]^+$ 528.3585, found: 528.3555

Compound **12o**

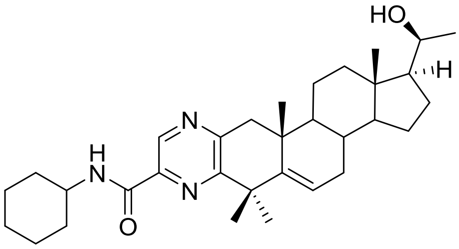

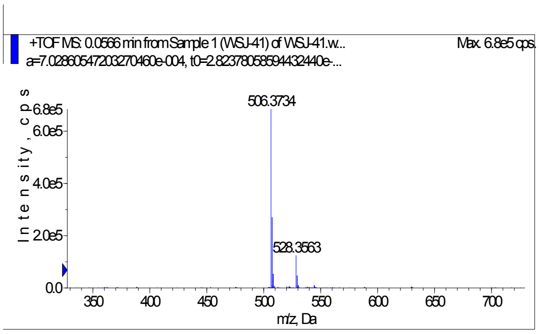

calcd for $[M+H]^+$ 506.3741, found: 506.3734
